# ShenRongGuBenHuanShao Pill Ameliorates Hydrocortisone-Induced Erectile Dysfunction: Insights from Integrated Multi-Omics Analysis

**DOI:** 10.3390/ph19091456

**Published:** 2026-09-14

**Authors:** Yazhou Shao, Xinqian Song, Yichen Jing, Li Dong, Yanping Wang, Ke Sun, Fangdi Hu

**Affiliations:** 1 School of Pharmacy, Lanzhou University, 199 West Donggang Road, Lanzhou 730000, China; shaoyzh22@lzu.edu.cn (Y.S.); 220220944290@lzu.edu.cn (X.S.); jingych2023@lzu.edu.cn (Y.J.); dli2024@lzu.edu.cn (L.D.); ypwang13@lzu.edu.cn (Y.W.); 2 Lanzhou Foci Pharmaceutical Co., Ltd., No. 2289 Huashan Road, Lanzhou New Area, Lanzhou 730000, China; skwind@163.com; 3 Codonopsis Radix Industrial Technology Engineering Research Center, 199 West Donggang Road, Lanzhou 730000, China

**Keywords:** ShenRongGuBenHuanShao Pill, erectile dysfunction, flavonoids, NO/GC/cGMP signaling, PI3K/Akt/mTOR pathway, metabolomics

## Abstract

**Background/Objectives:** ShenRongGuBenHuanShao Pill (SRGBHSP) is a traditional Chinese medicine (TCM) used for the treatment of erectile dysfunction (ED). However, its active constituents and pharmacological mechanisms remain unclear. This study characterized the flavonoid constituents of SRGBHSP and investigated its effects on HC-associated ED-related alterations and potential molecular mechanisms using an integrated multi-omics strategy. **Methods:** SRGBHSP constituents were characterized by UPLC-Q-TOF-MS/MS, and representative flavonoids were quantitatively analyzed by UPLC-MS/MS. Rats exposed to HC received SRGBHSP (1.08, 2.16, or 4.32 g/kg), Yougui Pill, or sildenafil concurrently with HC administration. Pharmacological effects were evaluated using APO-induced erectile responses, histopathology, sperm quality, reproductive hormones, oxidative stress indices, and serum NO/GC/cGMP-related parameters. Integrated 16S rRNA sequencing, untargeted metabolomics, transcriptomics, and DIA-based proteomics were combined with RT-qPCR and Western blotting to investigate potential regulatory mechanisms. **Results:** A total of 131 flavonoids were characterized in SRGBHSP, including flavones, isoflavones, flavonols, flavanones, chalcones, flavans, anthocyanidins, and aurones. Quantitative analysis identified baicalin as the predominant flavonoid constituent. APO-induced erection frequency decreased from 3.50 ± 1.29 in the control group to 0.50 ± 0.58 in the model group and increased to 4.50 ± 1.29 following high-dose SRGBHSP intervention. SRGBHSP also improved reproductive hormone homeostasis, sperm quality, and oxidative stress status in HC-treated rats. SRGBHSP increased serum NOS, NO, GC, and cGMP levels and increased the mRNA and total protein expression of PI3K, Akt, and mTOR in penile tissue. Integrated multi-omics analyses revealed coordinated alterations in gut microbiota, glycerophospholipid metabolism, and penile transcriptomic and proteomic profiles. **Conclusions:** SRGBHSP attenuated multiple ED-related alterations associated with HC exposure, accompanied by improvements in reproductive function, oxidative stress status, and serum NO/GC/cGMP-related parameters. Integrated multi-omics analyses together with expression-level validation revealed coordinated alterations in gut microbiota, glycerophospholipid metabolism, and PI3K/Akt/mTOR-related molecules following SRGBHSP intervention. These findings suggest the potential involvement of PI3K/Akt/mTOR-related signaling in the response to SRGBHSP, although its activation status and causal contribution remain to be established. Flavonoids may represent important bioactive constituents associated with the observed effects of SRGBHSP. Overall, this study provides an integrated pharmacological and multi-omics framework for understanding the effects of SRGBHSP on HC-associated ED-related alterations.

## 1. Introduction

Erectile dysfunction (ED) is a common male sexual disorder characterized by the persistent inability to achieve or maintain an erection sufficient for satisfactory sexual performance [1]. Its prevalence increases markedly with age, affecting up to 50% of men in certain populations [2], with an estimated global burden exceeding 150 million affected individuals [3]. Given its high prevalence and profound impact on physical health, psychological well-being, and quality of life, ED remains a major public health concern worldwide.

The pathophysiology of ED involves multiple interconnected mechanisms, including endocrine dysfunction, neurological impairment, vascular injury, and oxidative stress [4]. Among these, oxidative stress is recognized as a key contributor to endothelial dysfunction and impaired erectile function. Nitric oxide (NO) serves as a principal mediator of penile erection, and activation of the NO/guanylate cyclase (GC)/cyclic guanosine monophosphate (cGMP) signaling pathway is essential for corpus cavernosum smooth muscle relaxation and normal erectile responses [5]. Accumulating evidence indicates that excessive oxidative stress reduces NO bioavailability and disrupts endothelial homeostasis, thereby contributing to the development and progression of ED [6]. Phosphodiesterase type 5 (PDE5) inhibitors are currently regarded as the first-line treatment for ED. However, their clinical application may be limited by adverse effects, contraindications, and unsatisfactory responses in certain patient populations [7]. Therefore, the development of effective and safer alternative therapies remains an important research priority. According to traditional Chinese medicine (TCM) theory, ED belongs to the category of “yangwei” and is primarily associated with kidney-yang deficiency. Accordingly, therapeutic strategies aimed at warming kidney-yang and replenishing essence have been widely employed for the management of ED. Increasing pharmacological evidence has demonstrated that kidney-yang-tonifying medicinal plants and prescriptions possess beneficial effects on erectile function, providing a scientific basis for their traditional clinical application [4,8].

ShenRongGuBenHuanShao Pill (SRGBHSP) is a classic TCM prescription that has been used for nearly a century to treat kidney-yang deficiency-related disorders. Traditionally, it is prescribed to warm kidney-yang, replenish essence, and improve male reproductive function, and has been clinically applied for conditions such as impotence, spermatorrhea, and premature ejaculation [9]. Several constituent medicinal materials of SRGBHSP, including *Panax ginseng* C.A. Mey., *Cistanche deserticola* Y.C. Ma, *Epimedium brevicornu* Maxim., and *Cinnamomum cassia* (L.) J. Presl, have been reported to possess antioxidant, reproductive-protective, and pro-erectile activities [10,11,12,13], providing a pharmacological rationale for investigating SRGBHSP in ED (Table S1). However, evidence derived from individual medicinal materials cannot be directly extrapolated to the efficacy of the complete multi-component formulation. Furthermore, recent clinical observations have suggested that SRGBHSP combined with tadalafil may improve erectile function in patients with ED [14]; nevertheless, the concomitant administration of tadalafil makes it difficult to distinguish the independent contribution of SRGBHSP. Therefore, despite its long-standing traditional use and supportive evidence from its constituent medicinal materials, direct evidence for the efficacy of SRGBHSP as a standalone intervention for ED remains limited, and its associated pharmacological mechanisms have not been systematically characterized.

Emerging evidence suggests that gut microbiota dysbiosis contributes to the pathogenesis of ED through its effects on host metabolism, endothelial function, and reproductive health [15,16]. Consequently, the concept of a gut microbiota–penis axis has attracted increasing attention as a potential regulatory network linking intestinal microbial communities with penile physiological function [17]. Given the multi-component and multi-target characteristics of TCM, integrated multi-omics approaches provide powerful tools for elucidating their complex mechanisms of action. However, whether SRGBHSP exerts therapeutic effects against ED through coordinated regulation of gut microbiota, metabolism, and penile molecular pathways remains unclear.

Building upon our previous studies characterizing the chemical constituents of SRGBHSP and its pharmacological effects on oligoasthenospermia [18,19], the present study extended the investigation to ED, a distinct reproductive disorder with different pathophysiological features. Unlike our previous studies, the present work systematically evaluated the effects of SRGBHSP in a hydrocortisone-induced ED rat model and integrated 16S rRNA sequencing, serum metabolomics, penile transcriptomics, and DIA-based penile proteomics to characterize coordinated changes across the gut microbiota, systemic metabolism, and penile molecular profiles. In addition, the flavonoid constituents of SRGBHSP were characterized and representative compounds were quantitatively analyzed. This integrated strategy was employed to explore the potential molecular mechanisms associated with the effects of SRGBHSP and to provide new insights into the interactions among gut microbiota, metabolism, and penile molecular regulation.

## 2. Results

### 2.1. Chemical Characterization and Quantitative Analysis of Flavonoids in SRGBHSP

Based on our previous phytochemical investigations, 714 compounds were identified in SRGBHSP in vitro, among which 223 compounds were detected in vivo after oral administration [18,19].

**Figure 1:**
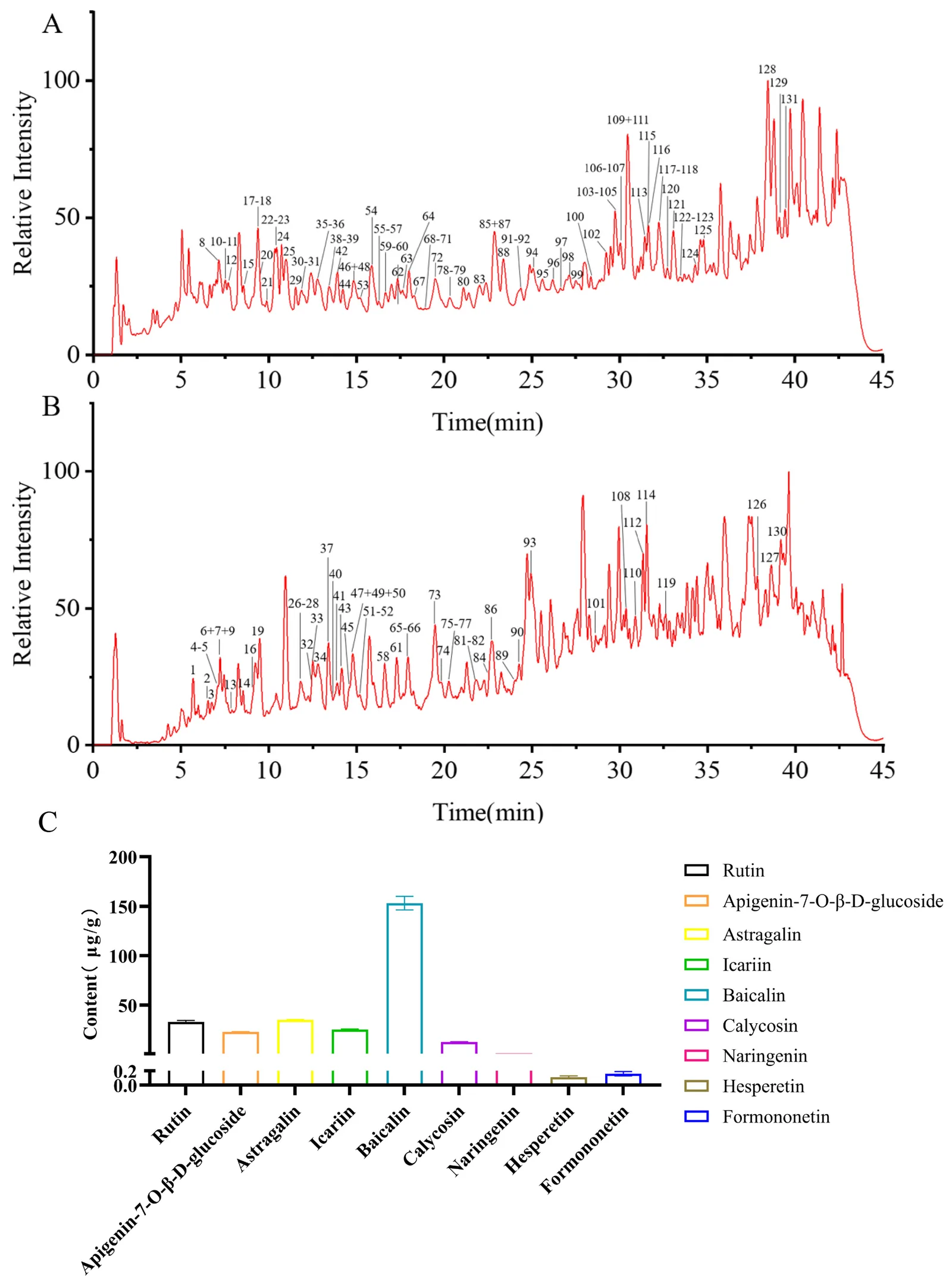
Total ion current (TIC) chromatograms of SRGBHSP. (**A**) Positive ion mode; (**B**) Negative ion mode; (**C**) Quantitative analysis of nine flavonoid constituents in SRGBHSP.

In the present study, UPLC-Q-TOF-MS/MS analysis characterized 131 flavonoids in SRGBHSP, including 58 flavones, 24 isoflavones, 21 flavonols, 16 flavanones, 5 chalcones, 4 flavans, 2 anthocyanidins, and 1 aurone. Representative total ion chromatograms acquired in positive and negative ion modes are shown in Figure 1A,B. Detailed information on retention times, accurate masses, mass errors, and fragmentation patterns is provided in Table S7.

Quantitative analysis showed that baicalin was the most abundant among the representative flavonoids analyzed (185.38 ± 0.71 μg/g), followed by astragalin (39.90 ± 0.35 μg/g), whereas formononetin (0.25 ± 0.03 μg/g) and hesperetin (0.06 ± 0.01 μg/g) were detected at lower concentrations (Figure 1C). Method validation showed satisfactory specificity, linearity, precision, repeatability, stability, and recovery.

### 2.2. Effects of SRGBHSP on APO-Induced Erectile Responses and Reproductive Tissue Alterations in HC-Treated Rats

**Figure 2:**
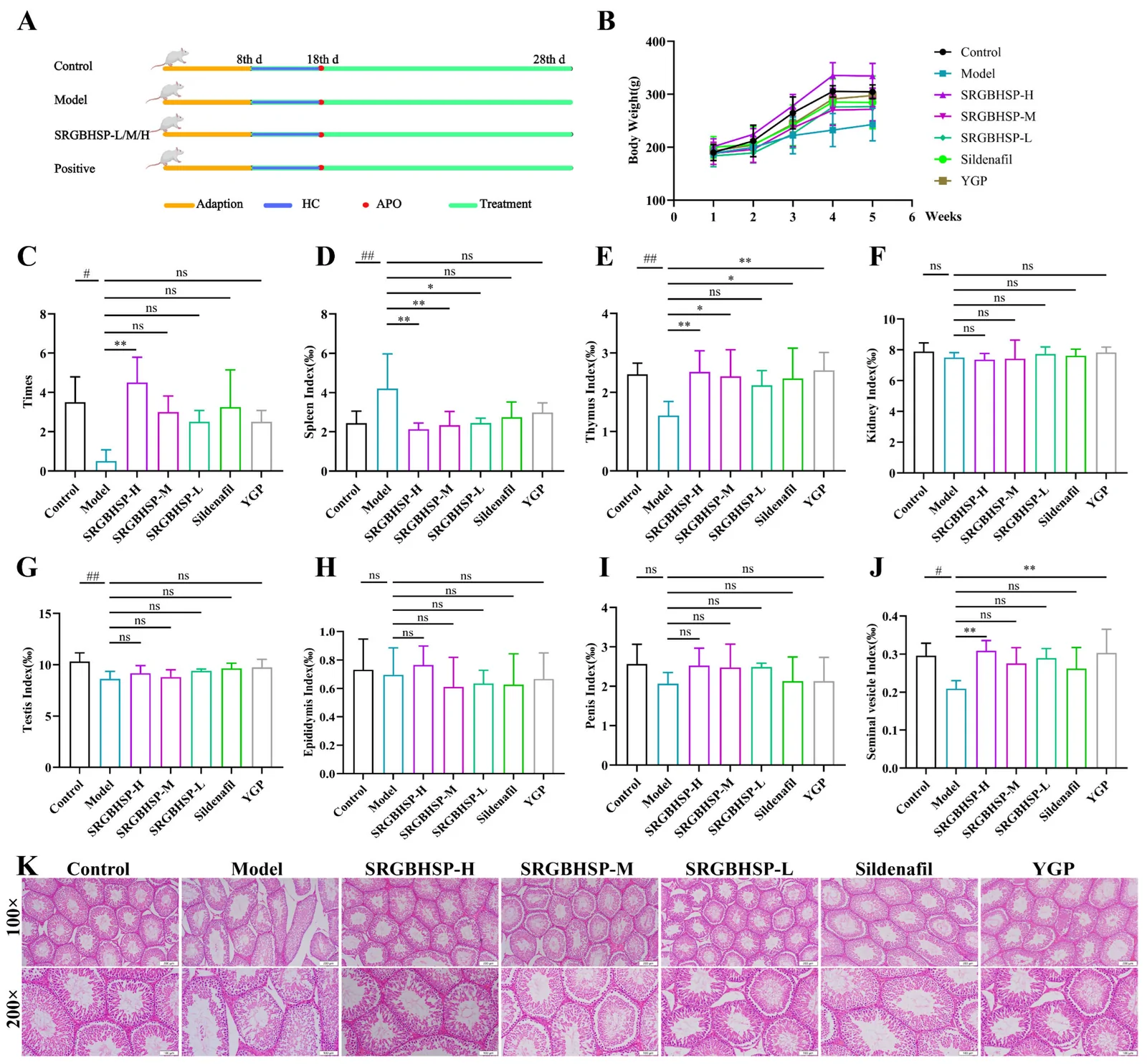
Establishment of the HC-induced ED rat model and effects of SRGBHSP intervention. (**A**) Schematic representation of the animal experimental protocol. (**B**) Changes in body weight during the experimental period (*n* = 6). Body-weight data were analyzed using two-way repeated-measures ANOVA followed by Dunnett’s multiple-comparisons test. (**C**) APO-induced penile erection frequency (*n* = 4). (**D**) Spleen index (*n* = 6). (**E**) Thymus index (*n* = 6). (**F**) Kidney index (*n* = 6). (**G**) Testis index (*n* = 6). (**H**) Epididymis index (*n* = 6). (**I**) Penile index (*n* = 6). (**J**) Seminal vesicle index (*n* = 6). (**K**) Representative hematoxylin and eosin (HE)-stained images of testicular tissue (*n* = 3) (scale bar: 200 μm). Quantitative data are presented as the mean ± SD. Compared with the control group: # *p* < 0.05, ## *p* < 0.01; compared with the model group: * *p* < 0.05, ** *p* < 0.01; ns, *p* > 0.05.

APO-induced erection frequency was significantly lower in the model group than in the control group. SRGBHSP administration increased APO-induced erection frequency, with a significant difference observed between the SRGBHSP-H and model groups (Figure 2C). During the experimental period, body weight gain was reduced in the model group, whereas SRGBHSP administration increased body weight, with the greatest increase observed in the high-dose group (Figure 2B). Two-way repeated-measures ANOVA revealed significant effects of time (*F* (3.641, 127.4) = 141.2, *p* < 0.0001) and treatment (*F* (6, 35) = 11.55, *p* < 0.0001), as well as a significant time × treatment interaction (*F* (24, 140) = 1.723, *p* = 0.0275). Dunnett’s post hoc analysis showed that body weight was significantly higher in the SRGBHSP-H group than in the model group at Weeks 2–5.

Analysis of organ indices showed that SRGBHSP administration significantly decreased the spleen index and increased the thymus index compared with the model group (Figure 2D,E). No significant differences were observed in the kidney, testis, or epididymis indices among the groups (Figure 2F–H).

Histopathological examination of testicular tissues showed degeneration and disorganization of the seminiferous tubules in the model group, accompanied by a reduction in spermatogenic cells. These histological alterations were attenuated following SRGBHSP administration, with more regularly arranged seminiferous tubules and increased spermatogenic cell populations observed in the SRGBHSP-treated groups (Figure 2K). Histological alterations in seminal vesicle tissues were also attenuated following SRGBHSP administration (Figure 2J).

**Figure 3:**
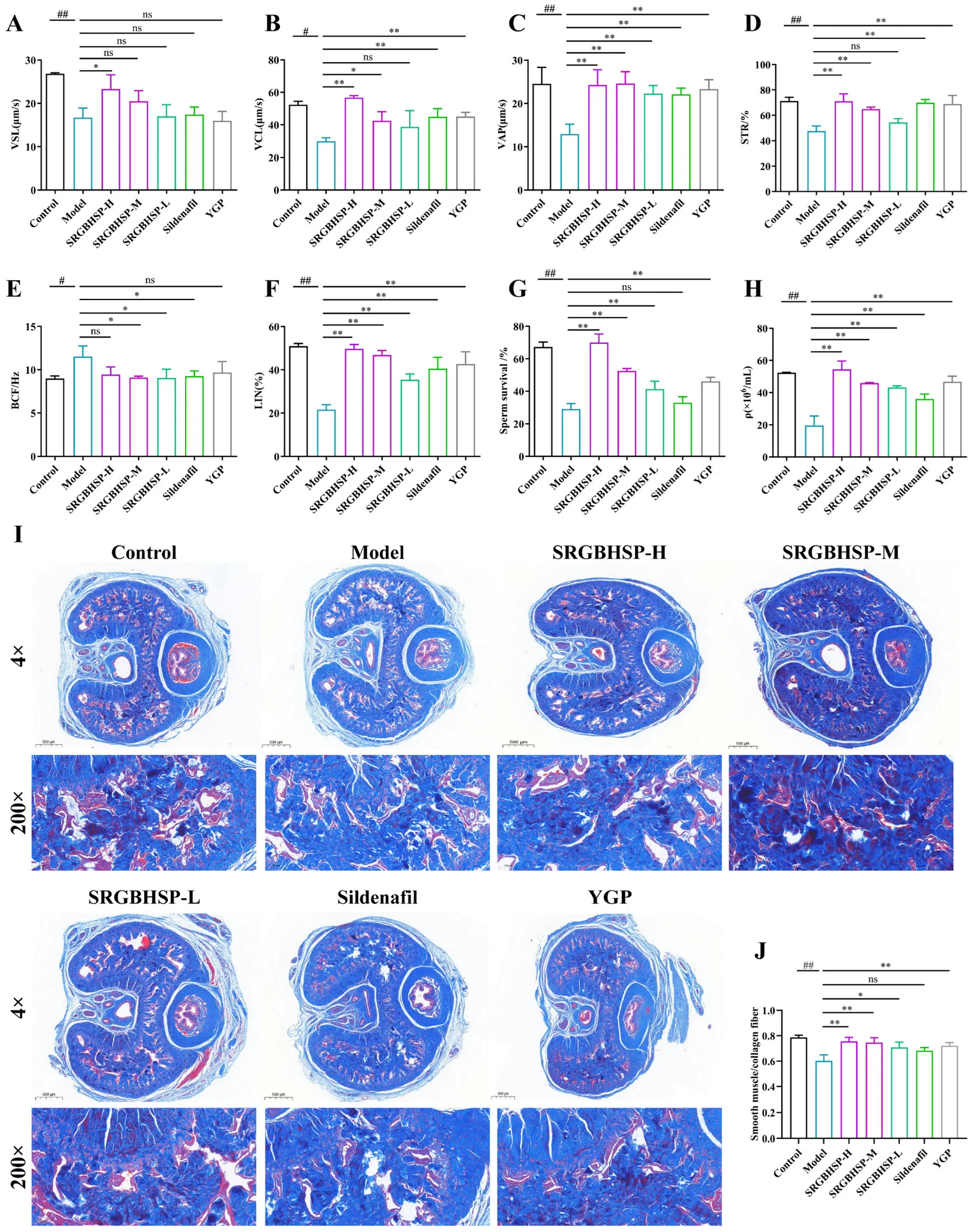
Effects of SRGBHSP on sperm quality and penile corpus cavernosum morphology in HC-induced ED rats. Sperm quality was evaluated by measuring VSL (**A**), VCL (**B**), VAP (**C**), STR (**D**), BCF (**E**), LIN (**F**), sperm viability (**G**), and sperm concentration (**H**) (*n* = 4). (**I**) Representative Masson’s trichrome-stained images of penile corpus cavernosum tissue (*n* = 3). (**J**) Quantitative analysis of the smooth muscle-to-collagen ratio (*n* = 3). Quantitative data are presented as the mean ± SD. Compared with the control group: # *p* < 0.05, ## *p* < 0.01; compared with the model group: * *p* < 0.05, ** *p* < 0.01; ns, *p* > 0.05.

Masson’s trichrome staining showed reduced smooth muscle content and a lower smooth muscle-to-collagen ratio in the corpus cavernosum of the model group compared with the control group. SRGBHSP administration increased both parameters in a dose-dependent manner (Figure 3I,J).

### 2.3. Effects of SRGBHSP on Sperm Quality Parameters in HC-Treated Rats

Compared with the control group, HC administration significantly reduced VSL, VCL, VAP, progressive motility, BCF, LIN, sperm viability, and sperm concentration (Figure 3A–H). SRGBHSP administration significantly increased multiple sperm quality parameters compared with the model group, with particularly marked changes observed in the medium- and high-dose groups (Figure 3A–H).

### 2.4. Effects of SRGBHSP on Serum Biochemical Indices, Renal Histology, and Reproductive Hormone Levels in HC-Treated Rats

**Figure 4:**
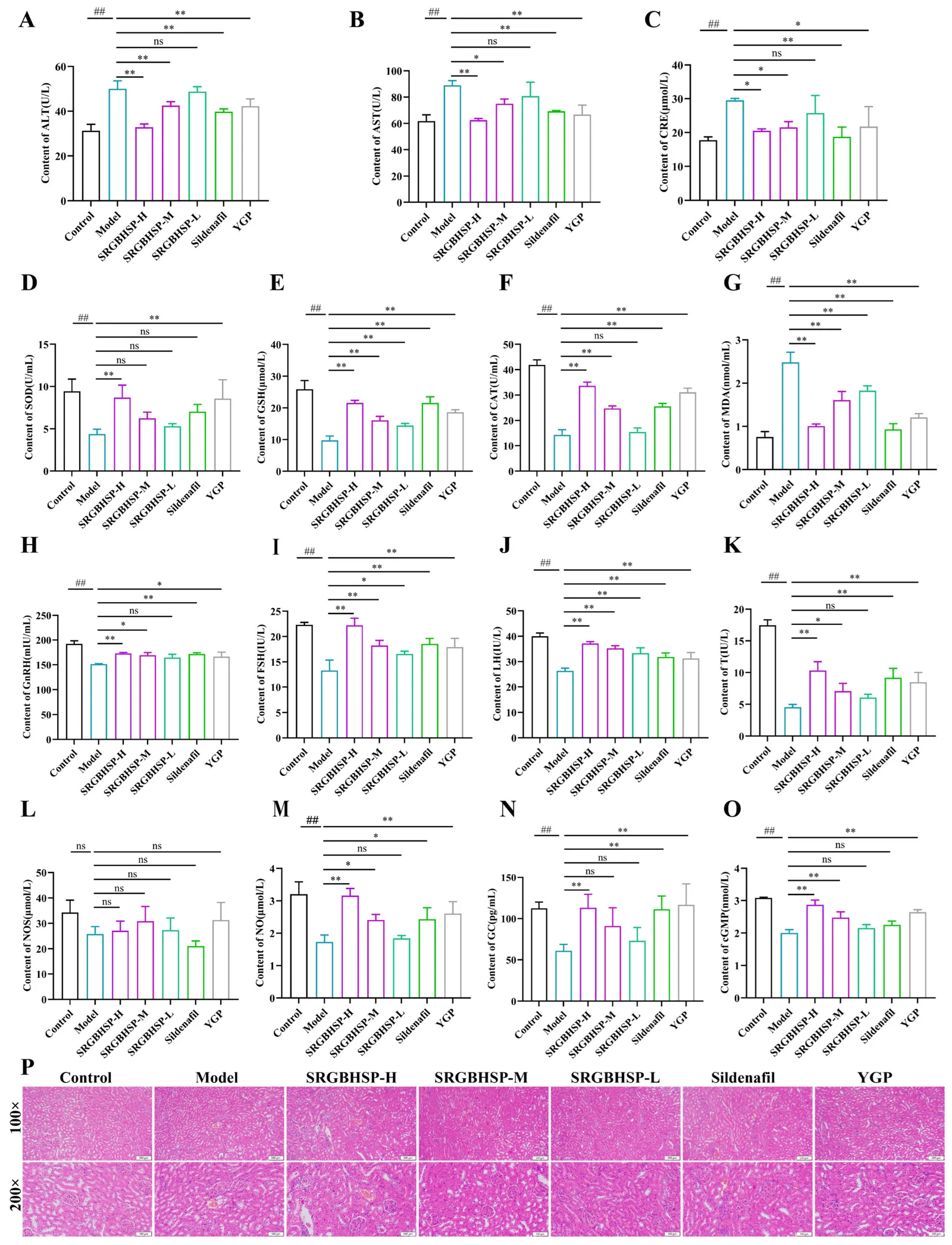
Effects of SRGBHSP on hepatorenal function-related parameters, oxidative stress-related indicators, reproductive hormones, and NO/GC/cGMP-related parameters in HC-induced ED rats. Serum levels of ALT (**A**), AST (**B**), and CRE (**C**) (*n* = 4). Serum levels of SOD (**D**), GSH (**E**), CAT (**F**), and MDA (**G**) (*n* = 4). Serum levels of GnRH (**H**), FSH (**I**), LH (**J**), and T (**K**) (*n* = 4). Serum levels of NOS (**L**), NO (**M**), GC (**N**), and cGMP (**O**) (*n* = 4). (**P**) Representative hematoxylin and eosin (HE)-stained images of kidney tissue (*n* = 3; scale bar: 200 μm). Quantitative data are presented as the mean ± SD. Compared with the control group: ## *p* < 0.01; compared with the model group: * *p* < 0.05, ** *p* < 0.01; ns, *p* > 0.05.

Compared with the control group, HC administration significantly increased serum ALT, AST, and CRE levels (Figure 4A–C). SRGBHSP administration reduced ALT, AST, and CRE levels compared with the model group, with the greatest reductions observed in the high-dose group (Figure 4A–C).

Histopathological examination of renal tissues showed glomerular atrophy and structural disruption in the model group. These histological alterations were attenuated following SRGBHSP administration (Figure 4P).

Serum levels of GnRH, FSH, LH, and T were significantly lower in the model group than in the control group (Figure 4H–K). SRGBHSP administration increased the levels of these reproductive hormones compared with the model group, with significant differences observed for multiple parameters in the medium- and high-dose groups (*p* < 0.01; Figure 4H–K).

### 2.5. Effects of SRGBHSP on Oxidative Stress and NO/GC/cGMP-Related Parameters in HC-Treated Rats

Compared with the control group, HC administration significantly increased MDA levels and decreased SOD, CAT, and GSH levels (Figure 4D–G). SRGBHSP administration decreased MDA levels and increased SOD, CAT, and GSH levels compared with the model group (Figure 4D–G).

NOS, NO, GC, and cGMP levels were significantly lower in the model group than in the control group (Figure 4L–O). SRGBHSP administration significantly increased these parameters compared with the model group, with significant differences observed for multiple parameters in the medium- and high-dose groups (*p* < 0.01; Figure 4L–O).

### 2.6. Effects of SRGBHSP on Gut Microbiota Composition in HC-Treated Rats

**Figure 5:**
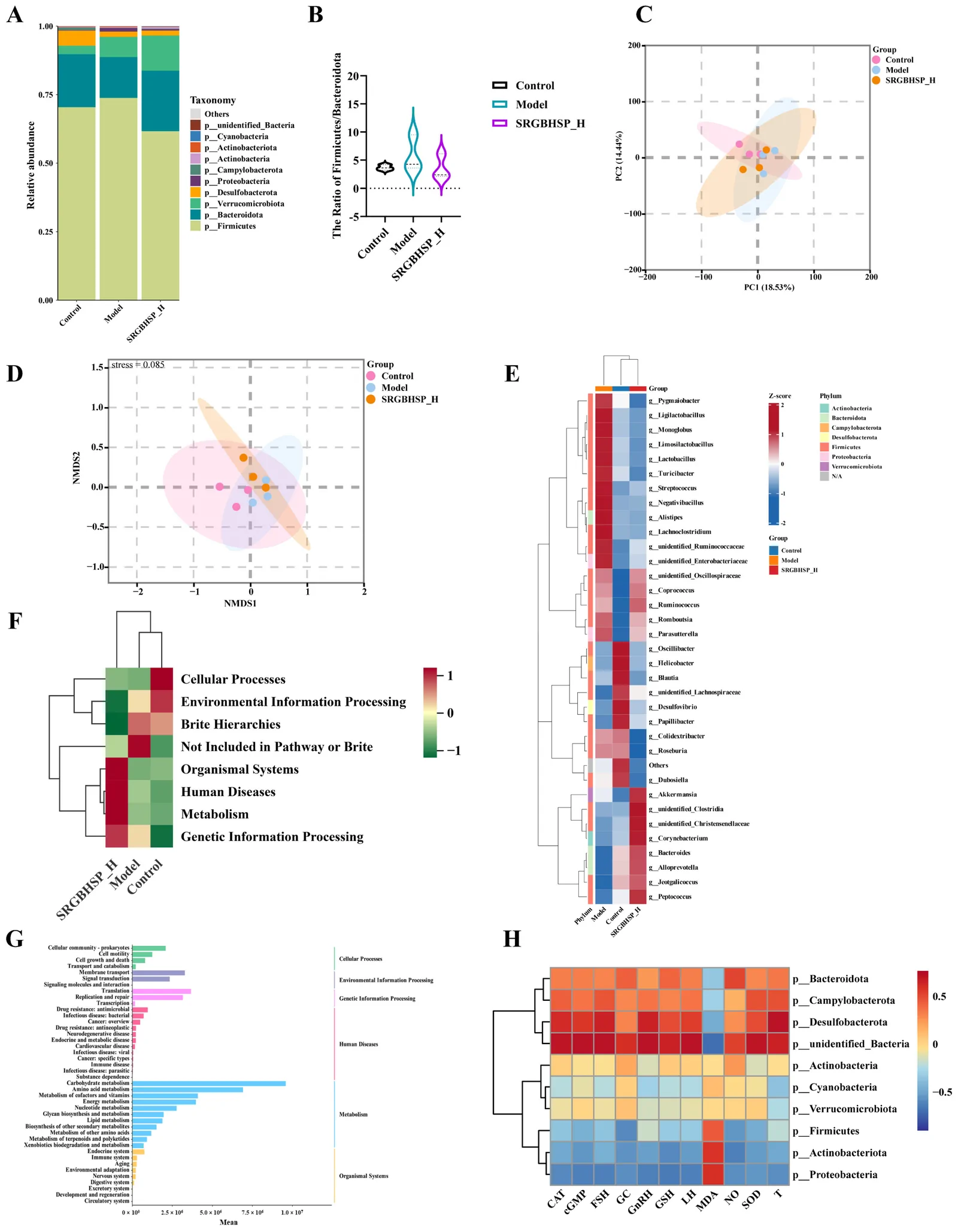
Effects of SRGBHSP on gut microbiota composition in HC-induced ED rats based on 16S rRNA sequencing (*n* = 3). (**A**) Relative abundance of gut microbiota at the phylum level. (**B**) Firmicutes/Bacteroidetes (F/B) ratio among the three experimental groups. The dashed lines indicate the median values. (**C**) PCA of gut microbial communities. (**D**) NMDS analysis. (**E**) Relative abundance of gut microbiota at the genus level. (**F**) Heatmap of PICRUSt2-predicted microbial functional profiles. (**G**) Relative abundance of PICRUSt2-predicted functional categories. (**H**) Correlation analysis between gut microbial taxa at the phylum level and oxidative stress-related parameters or reproductive hormone levels.

16S rRNA sequencing was performed using cecal content samples from the control, model, and SRGBHSP_H groups. PCoA showed distinct clustering of microbial communities among the three groups (Figure 5B,C).

At the phylum level, Firmicutes, Bacteroidota, Verrucomicrobiota, and Desulfobacterota were the dominant taxa across the three groups (Figure 5A), with differences in their relative abundances observed among groups. At the genus level, *Ligilactobacillus*, *Monoglobus*, *Streptococcus*, *Negativibacillus*, *Alistipes*, and *Lachnoclostridium* showed higher relative abundances in the model group than in the control group, whereas their relative abundances were lower in the SRGBHSP_H group than in the model group (Figure 5D).

PICRUSt2-based functional prediction showed differences in predicted microbial functions among the groups, with carbohydrate metabolism, amino acid metabolism, and metabolism of cofactors and vitamins among the major functional categories identified (Figure 5E,F).

Correlation analysis identified significant associations between several microbial taxa and host physiological parameters. An unidentified bacterial taxon was positively correlated with antioxidant-related parameters and reproductive hormone levels, whereas Proteobacteria and Actinobacteriota were negatively correlated with these parameters (Figure 5G).

### 2.7. Effects of SRGBHSP on Serum Metabolic Profiles in HC-Treated Rats

**Figure 6:**
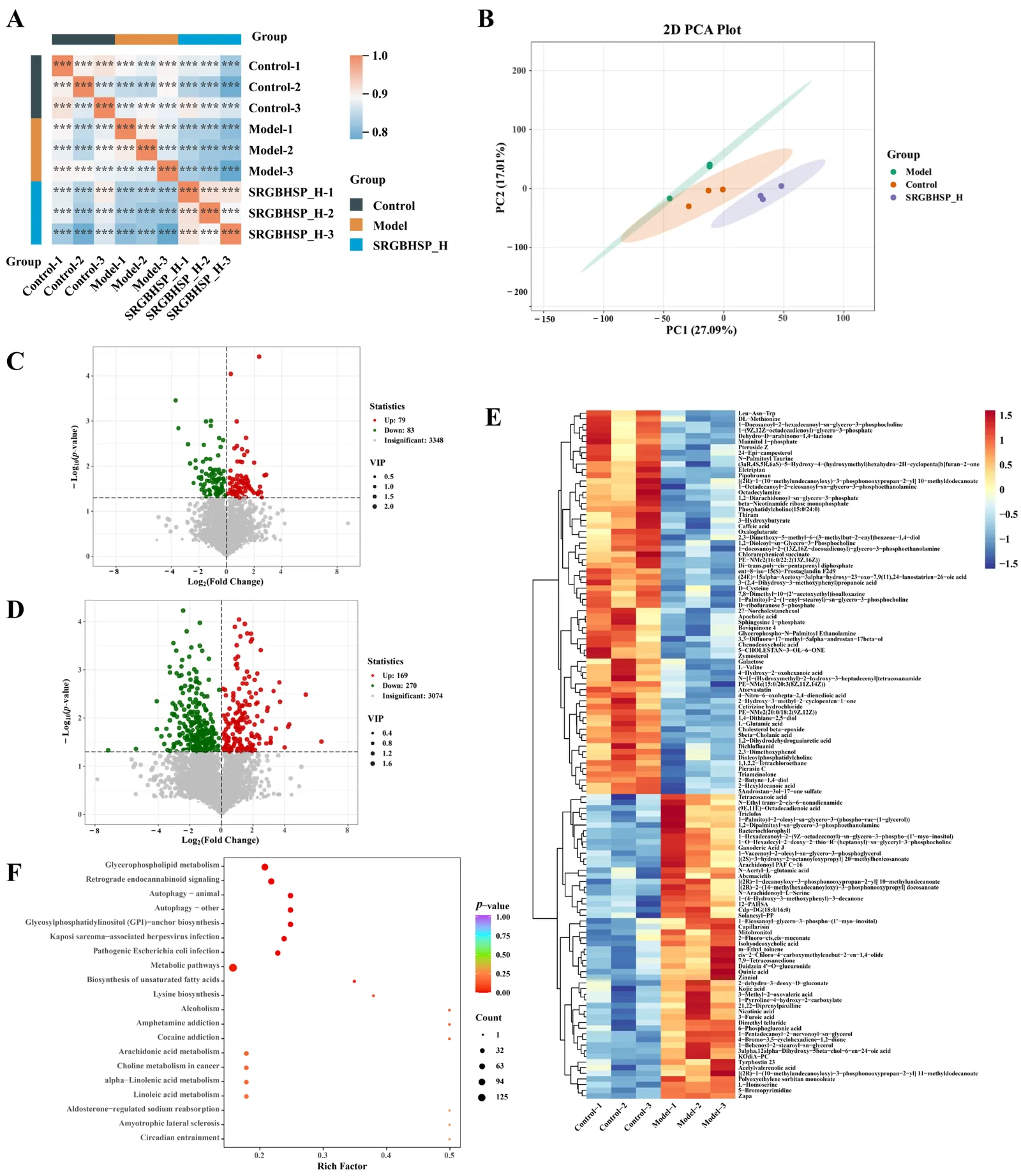
Effects of SRGBHSP on serum metabolic profiles in HC-induced ED rats based on untargeted metabolomic analysis (*n* = 3). (**A**) Inter-sample correlation analysis. The symbols *** indicate statistical significance at *p* < 0.001, respectively. (**B**) PCA. Volcano plots of differential metabolites in the Model versus Control comparison (**C**) and the SRGBHSP_H versus Model comparison (**D**). (**E**) Heatmap of differential metabolites among the Control, Model, and SRGBHSP_H groups. (**F**) KEGG pathway enrichment analysis of differential metabolites in the SRGBHSP_H versus Model comparison (top 20 enriched pathways).

Untargeted serum metabolomic analysis was performed in the control, model, and SRGBHSP_H groups. A total of 3803 metabolite features were detected across positive and negative ionization modes, of which 3513 metabolites were annotated. The identified metabolites were mainly classified as glycerophospholipids, benzene derivatives, and organic acids and their derivatives. Intra-group correlation analysis showed high correlations among biological replicates (Figure 6A).

Compared with the control group, 162 differential metabolites were identified in the model group, including 79 upregulated and 83 downregulated metabolites. In the model versus SRGBHSP_H comparison, 439 differential metabolites were identified, comprising 169 upregulated and 270 downregulated metabolites (Figure 6B–E).

KEGG enrichment analysis of the differential metabolites in the model versus SRGBHSP_H comparison identified several significantly enriched pathways. Glycerophospholipid metabolism showed the highest enrichment significance, with retrograde endocannabinoid signaling and autophagy-related pathways also identified among the enriched pathways (Figure 6F, Table S8).

### 2.8. Effects of SRGBHSP on Penile Proteomic Profiles in HC-Treated Rats

**Figure 7:**
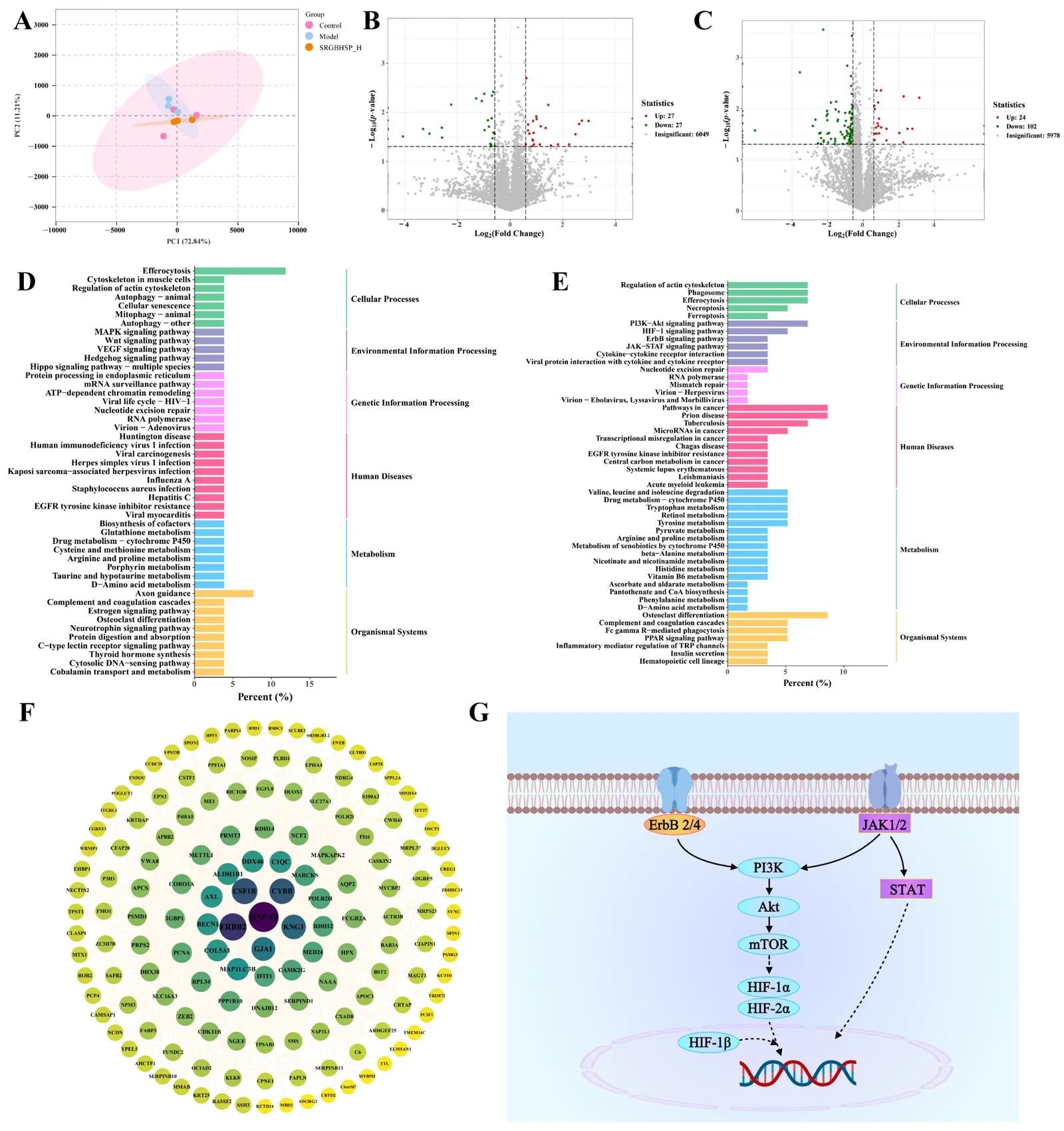
Effects of SRGBHSP on penile proteomic profiles in HC-induced ED rats (*n* = 3). (**A**) PCA of penile proteomic profiles. Volcano plots of DEPs in the Model versus Control comparison (**B**) and the SRGBHSP_H versus Model comparison (**C**), the dashed lines indicate the cutoff values for statistical significance (p value) and fold change (log_2_FC). KEGG pathway enrichment analysis of DEPs in the Model versus Control comparison (**D**) and the SRGBHSP_H versus Model comparison (**E**). (**F**) PPI network analysis of DEPs. The color gradient of nodes represents the degree value, with darker colors indicating higher degree values. (**G**) Schematic summary of proteomics-derived signaling pathways potentially associated with the effects of SRGBHSP in HC-induced ED rats. The dashed line indicates a predicted indirect relationship or potential regulatory interaction. Arrows indicate the direction of the predicted regulatory relationships among components.

DIA-based proteomic analysis was performed using penile tissues from the control, model, and SRGBHSP_H groups. Quality-control analysis showed the distributions of peptide length, peptide identification, and missed cleavage sites (Figure 7A). A total of 59,300 peptides corresponding to 6160 annotated proteins were identified.

Using a threshold of fold change > 1.5 and *p* ≤ 0.05, 54 differentially expressed proteins (DEPs) were identified between the control and model groups, including 27 upregulated and 27 downregulated proteins. In the model versus SRGBHSP_H comparison, 126 DEPs were identified, comprising 24 upregulated and 102 downregulated proteins (Figure 7A–C, Table S9).

KEGG pathway enrichment analysis of the DEPs identified in the model versus SRGBHSP_H comparison showed significant enrichment of multiple signaling pathways. The PI3K/Akt signaling pathway was among the significantly enriched pathways, together with the HIF-1, ErbB, and JAK/STAT signaling pathways (Figure 7D, Table S9).

### 2.9. Effects of SRGBHSP on Penile Transcriptomic Profiles in HC-Treated Rats

**Figure 8:**
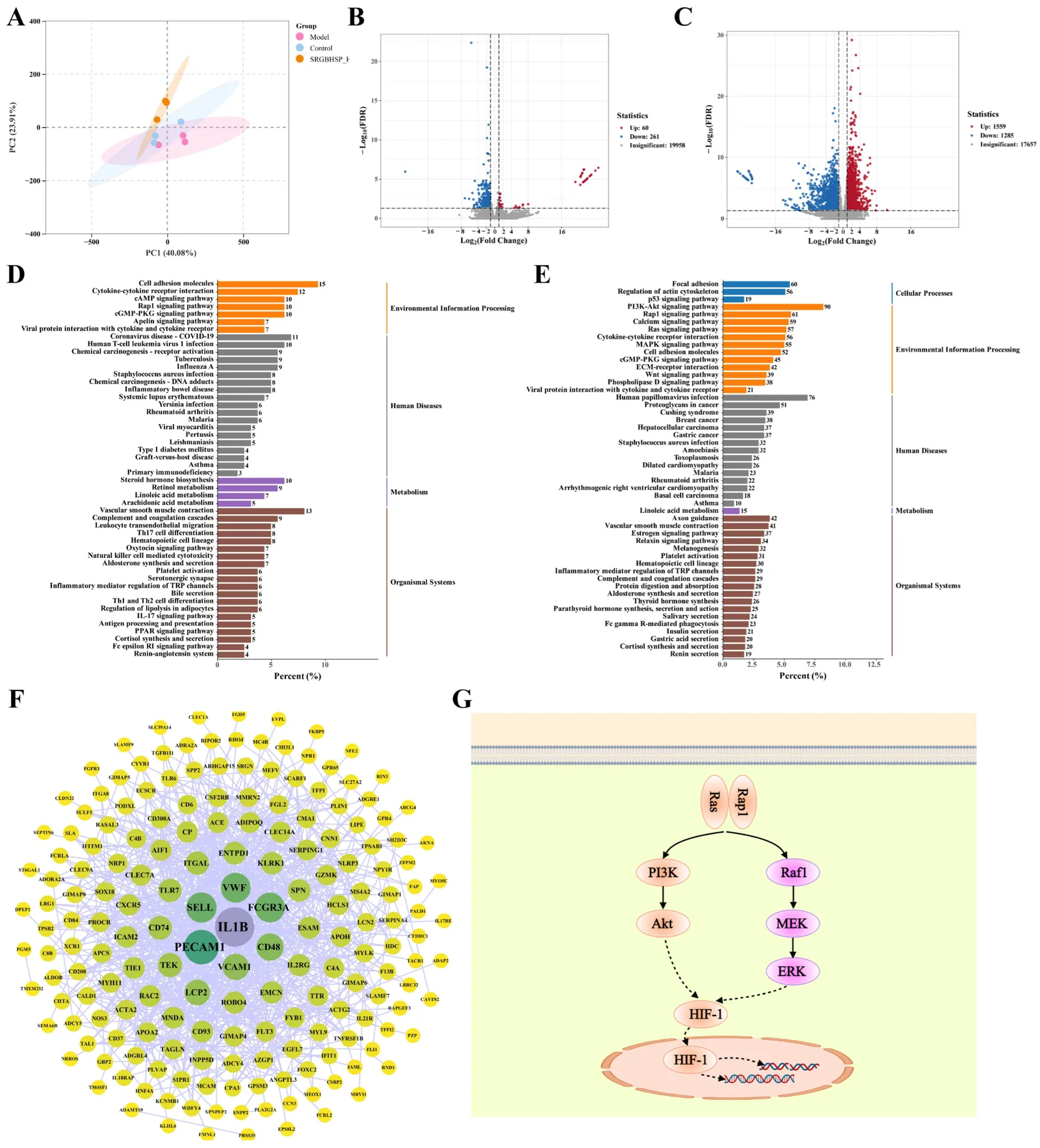
Effects of SRGBHSP on penile transcriptomic profiles in HC-induced ED rats (*n* = 3). (**A**) PCA of penile transcriptomic profiles. Volcano plots of DEGs in the Model versus Control comparison (**B**) and the SRGBHSP_H versus Model comparison (**C**). KEGG pathway enrichment analysis of DEGs in the Model versus Control comparison (**D**) and the SRGBHSP_H versus Model comparison (**E**). (**F**) PPI network analysis based on DEGs. The color gradient of nodes represents the degree value, with higher degree values indicated by darker colors. (**G**) Schematic summary of transcriptomics-derived signaling pathways potentially associated with the effects of SRGBHSP in HC-induced ED rats.

RNA-seq analysis was performed using penile tissues from the control, model, and SRGBHSP_H groups. A total of 33.8 Gb of clean sequencing data were generated, with an average of 3.75 Gb per sample. Q20 and Q30 values exceeded 99.47% and 97.73%, respectively, and the mapping rates ranged from 95.33% to 98.23%. A total of 22,033 genes were detected across all samples, with 283 DEGs shared between the control versus model and model versus SRGBHSP_H comparisons (Figure 8A).

Using the criteria of |log_2_FC| ≥ 1 and FDR < 0.05, 321 differentially expressed genes (DEGs) were identified in the control versus model comparison, including 60 upregulated and 261 downregulated genes (Figure 8B). In the model versus SRGBHSP_H comparison, 2844 DEGs were identified, comprising 1559 upregulated and 1285 downregulated genes (Figure 8C).

KEGG enrichment analysis of the DEGs identified in the model versus SRGBHSP_H comparison showed significant enrichment of multiple signaling pathways, including the PI3K/Akt, Rap1, Ras, and MAPK signaling pathways (Figure 8D,E; Table S10).

### 2.10. Integrative Multi-Omics Analysis Identifies Associations Among Gut Microbiota, Metabolites, Proteins, and Genes

**Figure 9:**
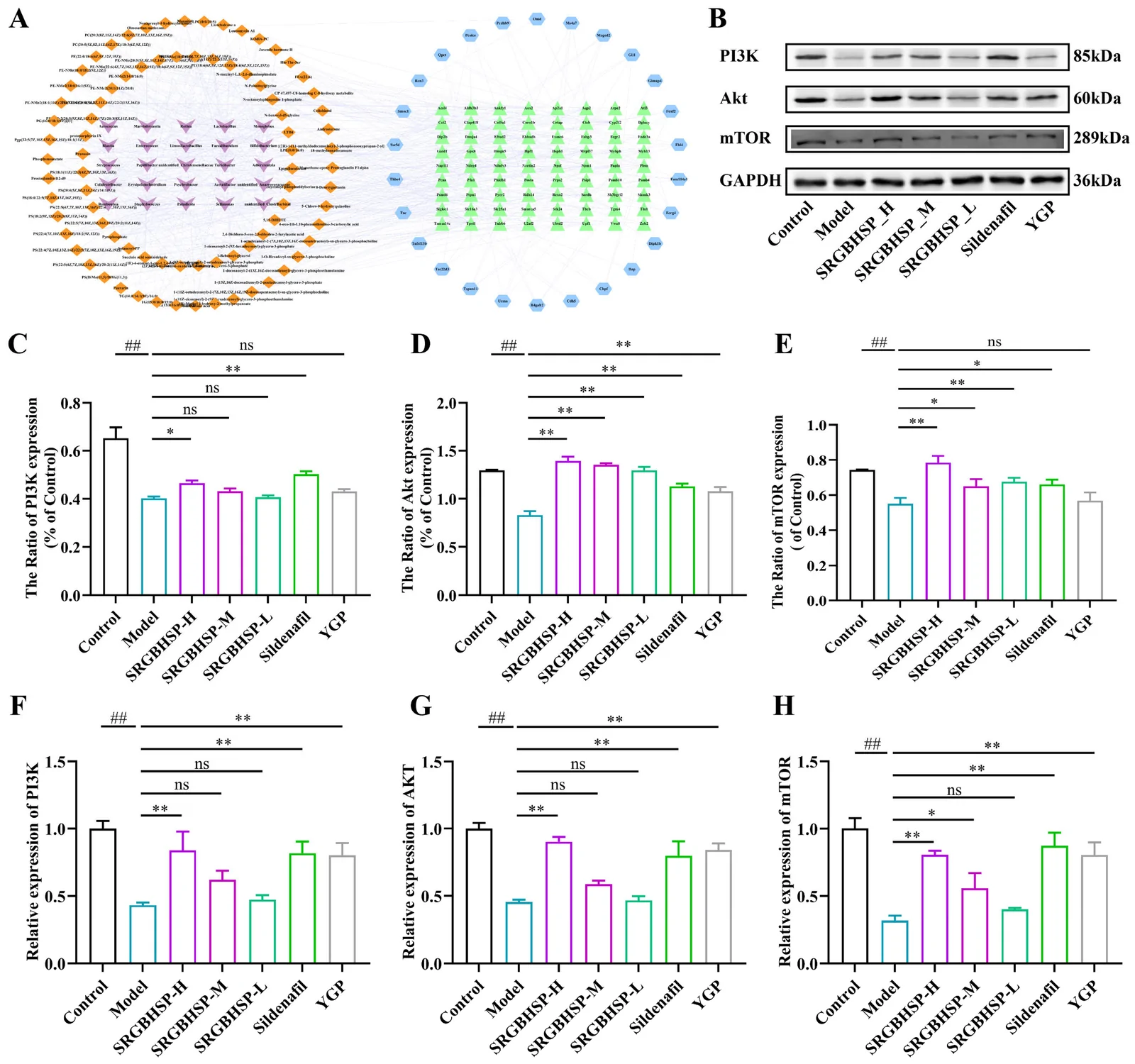
Integrated multi-omics analysis and validation of PI3K/Akt/mTOR-related molecular changes associated with SRGBHSP intervention in HC-induced ED rats. (**A**) Integrative correlation network of significantly altered features identified from the gut microbiome, metabolome, proteome, and transcriptome datasets (purple, gut microbial taxa; orange, metabolites; green, proteins; blue, genes). Different node shapes are used for visual distinction among omics features. (**B**) Representative Western blot images of PI3K, Akt, and mTOR in penile tissues. Relative protein expression levels of PI3K (**C**), Akt (**D**), and mTOR (**E**) quantified by densitometric analysis (*n* = 3 biological samples per group). RT-qPCR analysis of the relative mRNA expression levels of PI3K (**F**), Akt (**G**), and mTOR (**H**) (*n* = 3). Quantitative data are presented as the mean ± SD. Compared with the control group: ## *p* < 0.01; compared with the model group: * *p* < 0.05, ** *p* < 0.01; ns, *p* > 0.05.

Pearson correlation analysis was performed among significantly altered features identified across the multi-omics datasets. Significant correlations were identified among microbial taxa, differential metabolites, proteins, and genes (Figure 9A). Representative examples of these correlated features included metabolites such as puerarin, D-Nmappd, and felbamate, proteins such as SH3BGRL2, LRRC20, and PSMB10, and genes including PMFBP1, COL4A1, and RHOJ (Figure 9A).

### 2.11. Effects of SRGBHSP on PI3K/Akt/mTOR Pathway-Related Gene and Protein Expression in Penile Tissues

**Figure 10:**
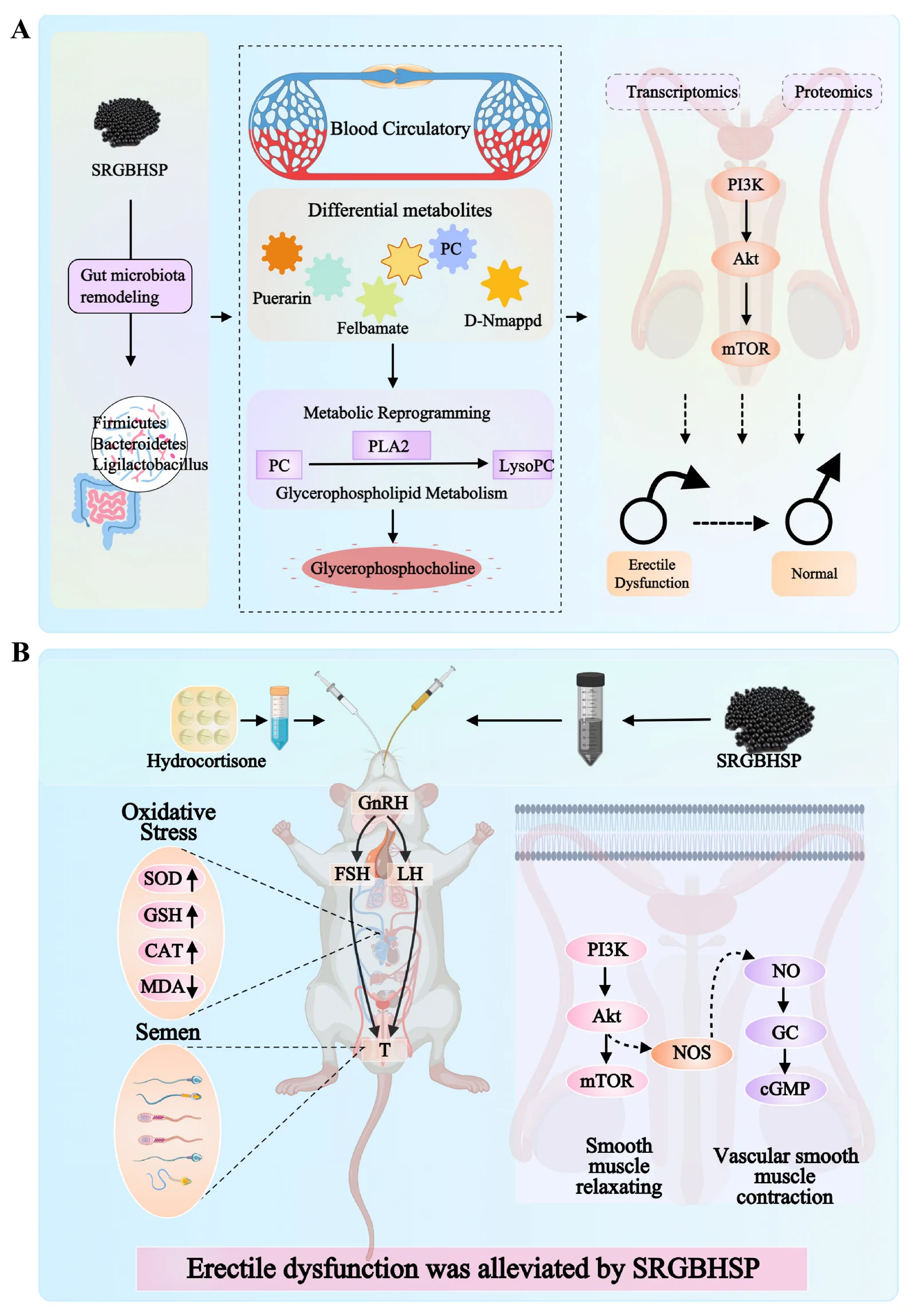
Proposed mechanisms associated with the ameliorative effects of SRGBHSP on ED. (**A**) Integrated analysis of the potential mechanisms associated with SRGBHSP administration based on the multi-omics findings of the present study and previously published evidence. (**B**) Proposed mechanistic model integrating the overall pharmacological and multi-omics findings of the present study with previously reported biological relationships. Solid arrows indicate the proposed relationships among the components based on the integrated pharmacological and multi-omics findings. Dashed arrows indicate proposed regulatory relationships inferred from the present findings and published evidence that were not functionally validated in the present study.

Based on the enrichment of PI3K/Akt signaling in both the transcriptomic and proteomic analyses, the expression of PI3K/Akt/mTOR pathway-related molecules was further examined by Western blotting and RT-qPCR. Western blot analysis showed that the protein expression levels of PI3K, Akt, and mTOR were significantly lower in the model group than in the control group. SRGBHSP administration significantly increased the expression levels of these proteins compared with the model group (Figure 9B–E). Consistently, RT-qPCR analysis showed lower mRNA expression levels of PI3K, Akt, and mTOR in the model group than in the control group, whereas SRGBHSP administration significantly increased their mRNA expression levels compared with the model group (Figure 9F–H, Figure 10A).

## 3. Discussion

Erectile dysfunction (ED) is a multifactorial disorder involving endocrine disturbances, oxidative stress, vascular dysfunction, and impaired regulation of corpus cavernosum smooth muscle tone [4]. Although phosphodiesterase type 5 (PDE5) inhibitors remain the first-line pharmacological treatment for ED, their clinical utility may be limited by contraindications, adverse effects, and inadequate responses in a subset of patients [2,7]. These limitations have encouraged investigation of complementary therapeutic strategies, including traditional Chinese medicine (TCM), whose multi-component nature may allow simultaneous modulation of multiple biological processes relevant to ED. However, the pharmacological effects and molecular basis of many traditional formulations require rigorous experimental evaluation using modern pharmacological approaches.

Against this background, the present study systematically evaluated the effects of SRGBHSP in an HC-induced rat model and characterized the associated biological responses using an integrated multi-omics strategy. SRGBHSP increased APO-induced erectile responses, improved reproductive hormone and sperm parameters, attenuated oxidative stress-related alterations, and increased NOS, NO, GC, and cGMP levels in HC-treated rats. Integrated 16S rRNA sequencing, serum metabolomics, penile transcriptomics, and DIA-based proteomics further identified alterations in gut microbial composition, glycerophospholipid metabolism, and penile molecular profiles following SRGBHSP administration. In particular, PI3K/Akt signaling was enriched in both transcriptomic and proteomic analyses, while subsequent RT-qPCR and Western blot analyses showed increased expression of PI3K, Akt, and mTOR at the mRNA and total protein levels. Collectively, these findings provide multi-level evidence for biological responses associated with SRGBHSP administration in HC-treated rats, while the causal relationships among these molecular alterations and their direct contribution to erectile responses require further investigation.

SRGBHSP is a classical kidney-tonifying TCM formula that has been used clinically for nearly one century for kidney-yang deficiency-related disorders, including impotence, premature ejaculation, and spermatorrhea [9]. Although a recent clinical study reported beneficial outcomes when SRGBHSP was administered in combination with tadalafil to patients with ED [14], this combination therapy does not allow the independent contribution of SRGBHSP to be determined, and direct experimental evidence regarding its effects on ED remains limited. In the present study, 131 flavonoids were characterized in SRGBHSP, encompassing flavones, isoflavones, flavonols, flavanones, chalcones, flavans, anthocyanidins, and aurones. Among the representative flavonoids subjected to quantitative analysis, baicalin showed the highest content, followed by astragalin. Several flavonoids detected in SRGBHSP, including baicalin, icariin, naringenin, rutin, calycosin, and formononetin, have previously been associated with antioxidant, endothelial-protective, or reproductive effects, and some have shown beneficial effects in experimental models of ED [20,21,22,23]. These previous findings provide a pharmacological rationale for considering flavonoids as potentially relevant constituents of SRGBHSP. However, the present chemical profiling does not establish which individual flavonoids contribute directly to the observed effects, and further pharmacokinetic and compound-specific pharmacological studies are required to define their relative contributions.

The HC-induced model was used to evaluate the effects of SRGBHSP on ED-related phenotypes associated with glucocorticoid exposure. HC administration markedly reduced APO-induced erection frequency and was accompanied by alterations in body weight, reproductive hormones, reproductive tissues, and several systemic biochemical parameters. SRGBHSP administration increased APO-induced erection frequency and attenuated several of these HC-associated alterations. APO-induced erection testing has been used in previous experimental studies to evaluate erectile responses in rat models of ED [24,25]. However, APO-induced erection testing primarily reflects pharmacologically evoked erectile responsiveness and does not provide the direct hemodynamic information obtained from cavernous nerve stimulation combined with intracavernosal pressure and mean arterial pressure measurements [26]. Therefore, the increased APO-induced erection frequency observed in the present study should be interpreted as evidence of improved erectile responsiveness rather than definitive restoration of physiological erectile function. In addition, changes in spleen and thymus indices were observed following HC administration and were partially attenuated by SRGBHSP. Given the broad systemic effects of glucocorticoid exposure, these changes should not be interpreted as evidence of a specific immunomodulatory mechanism without further immune-function assessment. Collectively, the present findings indicate that SRGBHSP attenuated multiple ED-related and systemic alterations associated with HC exposure, although the extent to which these effects reflect specific modulation of erectile physiology versus attenuation of broader glucocorticoid-induced disturbances requires further investigation. The HC-induced model differs from other commonly used experimental models of ED in its pathophysiological context. Diabetic ED models reproduce erectile impairment associated with chronic metabolic, endothelial, and neurovascular abnormalities, whereas aging models incorporate age-related vascular, neural, endocrine, and structural alterations. Castration models are particularly useful for investigating androgen-dependent regulation of erectile physiology. Arteriogenic or vascular models directly address impaired penile blood supply or vascular dysfunction, whereas cavernous nerve injury models reproduce neurogenic ED and are particularly relevant to erectile dysfunction following pelvic nerve injury [27,28]. In contrast, HC-induced models have been used to investigate ED-related alterations associated with systemic glucocorticoid exposure [29]. The HC model used in the present study similarly induces ED-related phenotypes in the context of broader systemic glucocorticoid effects involving endocrine, metabolic, oxidative, and tissue alterations. Therefore, this model may be useful for investigating ED-related changes associated with glucocorticoid exposure but does not recapitulate all major etiologies of clinical ED and should be regarded as complementary to, rather than a substitute for, more etiology-specific ED models.

Oxidative stress is an important contributor to both male reproductive dysfunction and erectile impairment. Excessive reactive oxygen species can disrupt testicular steroidogenesis and spermatogenesis while also impairing endothelial function and reducing NO bioavailability, thereby contributing to ED development [30,31]. In the present study, HC administration was associated with impaired sperm quality, decreased circulating reproductive hormone levels, histopathological alterations in testicular tissue, and an unfavorable systemic redox profile. SRGBHSP administration improved multiple sperm motility parameters and testicular histological features and increased circulating levels of GnRH, FSH, LH, and T. In parallel, SRGBHSP increased SOD and CAT activities and GSH levels while decreasing MDA levels. These observations are consistent with previous evidence linking oxidative stress to impaired testicular function and endocrine regulation. Given the close relationship among oxidative stress, steroidogenesis, spermatogenesis, and endothelial function, attenuation of HC-associated oxidative alterations may contribute to the improvements in reproductive parameters observed following SRGBHSP administration. However, the present data do not establish oxidative stress as the causal link between SRGBHSP administration and these reproductive effects.

The NO/GC/cGMP signaling cascade plays a central role in penile erection by regulating corpus cavernosum smooth muscle relaxation, and impairment of NO bioavailability is closely associated with ED [5]. Oxidative stress may further compromise this signaling system by reducing NO production and bioavailability. In the present study, HC administration significantly decreased NOS, NO, GC, and cGMP levels, whereas SRGBHSP administration increased these parameters. These changes occurred in parallel with increased APO-induced erection frequency and a higher smooth muscle-to-collagen ratio in the corpus cavernosum. The concurrent improvement in oxidative stress-related parameters is also consistent with the established relationship between redox homeostasis and NO bioavailability. However, because NOS, NO, GC, and cGMP were measured in serum rather than directly assessing pathway activity in penile tissue, these findings should be interpreted as systemic changes in NO/GC/cGMP-related parameters rather than direct evidence of activation of penile NO/GC/cGMP signaling. Moreover, the present study did not distinguish specific NOS isoforms or assess sGC activity, PDE5, or other downstream components of the pathway. Thus, the observed changes support a potential association between SRGBHSP administration and NO/GC/cGMP-related regulation, but further tissue-specific and functional studies are required to establish its direct contribution to the erectile response.

Given the multi-component and multi-target characteristics of TCM formulations, integrated multi-omics strategies provide a useful approach for characterizing biological responses across multiple molecular levels. In the present study, 16S rRNA sequencing, serum metabolomics, penile transcriptomics, and DIA-based penile proteomics were integrated to characterize the molecular changes associated with SRGBHSP administration in HC-treated rats. These analyses identified alterations in gut microbial composition, systemic metabolic profiles, and penile transcriptomic and proteomic profiles, providing a systems-level view of the biological responses associated with SRGBHSP intervention. However, because these omics analyses are primarily associative, the identified molecular changes and enriched pathways should be considered hypothesis-generating rather than evidence of direct mechanistic or causal relationships.

At the molecular level, transcriptomic and proteomic analyses identified enrichment of several signaling pathways potentially relevant to penile physiology. Notably, PI3K/Akt signaling was enriched in both datasets, while HIF-1, Rap1, Ras, MAPK, and JAK/STAT signaling pathways were also identified in the respective enrichment analyses. Previous studies have implicated these pathways in biological processes relevant to erectile physiology, including endothelial function, angiogenesis, oxidative stress responses, cell survival, and smooth muscle regulation. In addition, differential expression of multiple genes and proteins, including Grik2, Cdk15, Camk2g, and Ddx46, was observed following SRGBHSP administration. The convergence of transcriptomic and proteomic enrichment on PI3K/Akt signaling therefore identifies this pathway as a candidate molecular response associated with SRGBHSP intervention. Nevertheless, pathway enrichment reflects statistical overrepresentation of differentially expressed molecules and does not demonstrate pathway activation or establish that PI3K/Akt signaling is required for the observed effects. Similarly, the other enriched pathways may represent additional molecular responses to SRGBHSP administration, but their functional contributions remain to be experimentally determined.

Metabolomic profiling provides a valuable approach for characterizing biochemical alterations associated with disease states and pharmacological interventions [32]. In parallel, accumulating evidence suggests that gut microbiota dysbiosis may contribute to ED through its associations with systemic metabolism, oxidative stress, endothelial function, and hormonal regulation [16]. In the present study, 16S rRNA sequencing revealed differences in gut microbial community structure among the control, model, and SRGBHSP_H groups. PICRUSt2-based functional prediction further identified differences in predicted microbial functions related to carbohydrate metabolism, amino acid metabolism, and metabolism of cofactors and vitamins. Untargeted serum metabolomics also revealed marked differences in metabolic profiles, with glycerophospholipid metabolism showing the highest enrichment significance among the differential metabolites identified between the Model and SRGBHSP_H groups. Glycerophospholipids are major structural components of biological membranes and also participate in cellular signaling, oxidative stress responses, and vascular homeostasis. Previous studies have linked disturbances in lipid and glycerophospholipid metabolism to endothelial dysfunction and vascular abnormalities relevant to erectile physiology. Thus, the enrichment of glycerophospholipid metabolism observed in the present study identifies this pathway as a potentially relevant metabolic response associated with SRGBHSP administration, rather than demonstrating restoration of this metabolic pathway.

Integrative correlation analysis further identified significant associations among microbial taxa, differential metabolites, proteins, and genes, suggesting potential links among gut microbial alterations, systemic metabolic changes, and penile molecular responses. Importantly, these correlations do not establish the directionality or causality of the observed relationships. Therefore, although the present findings are consistent with the possibility of microbiota–metabolism–penile interactions, they do not establish a functional “gut microbiota–penis axis”. Further studies involving fecal microbiota transplantation, microbiota depletion, and targeted supplementation of candidate microbial metabolites would be required to determine whether gut microbial alterations contribute causally to the effects associated with SRGBHSP administration.

The PI3K/Akt/mTOR pathway regulates diverse cellular processes, including cell survival, metabolism, angiogenesis, and tissue homeostasis, and alterations in this pathway have been implicated in ED-related vascular and cellular dysfunction [2,33]. In the present study, PI3K/Akt signaling was enriched in both the transcriptomic and proteomic datasets following SRGBHSP administration, identifying this pathway as a candidate molecular response associated with the intervention. Subsequent RT-qPCR and Western blot analyses showed that the mRNA and total protein expression levels of PI3K, Akt, and mTOR, which were reduced in the Model group, were increased following SRGBHSP administration. The concordance between pathway enrichment and expression analyses provides additional evidence that PI3K/Akt/mTOR-related molecular alterations are associated with SRGBHSP intervention. However, increased mRNA and total protein abundance does not establish activation of the PI3K/Akt/mTOR pathway, which is substantially regulated through phosphorylation and other post-translational mechanisms. Moreover, the biological consequences of mTOR signaling may be context-dependent. mTOR has been implicated in the regulation of type I collagen production [34], whereas PI3K/Akt/mTOR signaling also participates in angiogenesis and vascular regulation [35]. Therefore, the biological significance of the increased total PI3K, Akt, and mTOR expression observed in the present study cannot be inferred from protein abundance alone. Because phosphorylation states and downstream effectors were not assessed, and no pharmacological inhibition or genetic perturbation was performed, the present findings do not establish pathway activation or demonstrate that PI3K/Akt/mTOR signaling is required for the effects associated with SRGBHSP. Further studies assessing phosphorylated PI3K, Akt, and mTOR, together with downstream targets and pathway-specific functional perturbation, are required to determine the functional contribution of this signaling pathway.

The NO/GC/cGMP signaling cascade plays a central role in penile erection by mediating NO-dependent relaxation of corpus cavernosum smooth muscle [5]. Previous studies have demonstrated biological crosstalk between PI3K/Akt signaling and NO regulation in vascular endothelial cells, in which Akt can phosphorylate eNOS and thereby promote NO production [35]. In the present study, SRGBHSP administration increased serum NOS, NO, GC, and cGMP levels, while PI3K/Akt signaling was concurrently enriched in the transcriptomic and proteomic datasets and the mRNA and total protein expression levels of PI3K, Akt, and mTOR were increased. These parallel changes are consistent with a potential relationship between PI3K/Akt/mTOR-related molecular responses and NO/GC/cGMP-related regulation. However, the present study did not assess phosphorylated Akt or eNOS, specific NOS isoforms, sGC subunits, PDE5, or other molecular components required to establish functional crosstalk between these signaling systems. Furthermore, no pathway-specific perturbation experiments were performed to determine their directional relationship. Therefore, the present data do not establish that PI3K/Akt/mTOR signaling functions upstream of NO/GC/cGMP signaling or mediates the effects associated with SRGBHSP administration. Rather, the concurrent alterations in these signaling-related parameters should be regarded as an association that provides a hypothesis for further mechanistic investigation. Future studies combining pathway-specific inhibition with assessment of p-Akt/Akt, p-eNOS/eNOS, sGC, cGMP, and PDE5 in penile tissue will be required to determine whether and how these signaling systems interact in the response to SRGBHSP.

Taken together, the present findings indicate that SRGBHSP administration was associated with concurrent changes in oxidative stress-related parameters, NO/GC/cGMP-related indicators, gut microbial composition, systemic metabolism, and penile molecular profiles in HC-treated rats. The convergence of transcriptomic and proteomic enrichment on PI3K/Akt signaling, together with altered PI3K, Akt, and mTOR expression, identifies this signaling pathway as a candidate molecular response associated with SRGBHSP intervention. Similarly, the observed changes in serum NO/GC/cGMP-related parameters and their known biological connections with PI3K/Akt signaling provide a rationale for further investigating potential crosstalk between these signaling systems (Figure 10B). However, the current data support an associative rather than causal mechanistic framework, and the directionality and functional relationships among these molecular changes remain to be established.

Several limitations of the present study should be acknowledged. First, erectile responses were evaluated using APO-induced erection testing without direct hemodynamic assessment by intracavernosal pressure and mean arterial pressure. Therefore, the observed increase in APO-induced erection frequency should be interpreted as evidence of improved erectile responsiveness rather than definitive restoration of erectile hemodynamic function. Future studies incorporating cavernous nerve stimulation and direct ICP/MAP measurements are warranted to further validate these findings. Moreover, because SRGBHSP administration was initiated concurrently with HC exposure, the present design primarily reflects attenuation of HC-associated ED-related alterations rather than reversal of established ED. Second, the multi-omics analyses primarily identified associations among gut microbiota, systemic metabolism, and penile molecular responses, and these relationships require functional validation. Third, although PI3K/Akt signaling was consistently identified by transcriptomic and proteomic analyses and changes in PI3K, Akt, and mTOR expression were further examined, phosphorylation states and pathway-specific functional perturbations were not assessed; therefore, pathway activation and causality cannot be established. Finally, further studies incorporating tissue-specific assessment of NO/GC/cGMP signaling and functional characterization of individual bioactive constituents will be necessary to more precisely define the pharmacological mechanisms of SRGBHSP.

## 4. Materials and Methods

### 4.1. Ethics Statement

All animal experimental procedures were approved by the Animal Ethics Committee of Lanzhou University (Approval No. MECI20240028) and performed in accordance with institutional guidelines and the ARRIVE guidelines. Animals were obtained from the Laboratory Animal Center of Lanzhou University (License No. SCXK (Gan) 2024–0002).

### 4.2. Materials

ShenRongGuBenHuanShao Pill (SRGBHSP, batch No. 22091) was provided by Lanzhou Foci Pharmaceutical Co., Ltd. (Lanzhou, China). The botanical composition of SRGBHSP is listed in Supplementary Materials (SRGBHSP Prescription Composition). Acetonitrile, methanol, and formic acid (LC-MS grade) were purchased from Thermo Fisher Scientific (Waltham, MA, USA). Distilled water was obtained from Watsons (Hong Kong, China). Reference standards including hesperetin, baicalin, naringenin, formononetin, calycosin, icariin, rutin, apigenin-7-O-β-D-glucoside, and astragalin (all ≥98% purity) were purchased from the corresponding suppliers. Detailed information regarding reference standards is provided in Table S2.

### 4.3. Sample Preparation of SRGBHSP

An appropriate amount of SRGBHSP powder was accurately weighed and extracted twice with 80% ethanol at a solid-to-liquid ratio of 1:25 under ultrasonic conditions at 70 °C for 60 min each time. The combined filtrates were concentrated under reduced pressure and lyophilized to obtain the total flavonoid extract of SRGBHSP (total flavonoid content: 14.99 mg/g).

### 4.4. Preparation of Sample Solution

A precise amount of the total flavonoid extract (100 mg) was dissolved in 70% acetonitrile to a final concentration of 10 mg/mL. The solution was centrifuged at 12,000 rpm for 10 min, and the supernatant was filtered through a 0.22 μm membrane prior to analysis.

### 4.5. Chromatographic Conditions

Chromatographic separation was performed on an InfinityLab Poroshell 120 EC–C18 column (150 mm × 2.1 mm, 1.9 μm) maintained at 30 °C. The mobile phase consisted of (A) 0.1% formic acid in water and (B) acetonitrile, with a gradient elution program as follows: 0–3 min, 10–20% B; 3–10 min, 20–25% B; 10–20 min, 25–30% B; 20–35 min, 30–58% B; 35–40 min, 58–85% B; 40–45 min, 85–10% B. The flow rate was 0.3 mL/min, and the injection volume was 3 μL.

### 4.6. Mass Spectrometry Conditions

Mass spectrometry was conducted using an electrospray ionization (ESI) source with nitrogen as the drying gas. The drying gas temperature was set at 225 °C with a flow rate of 10 L/min, and the nebulizer gas pressure was 25 psi. Capillary voltages were 3500 V in positive ion mode and 4000 V in negative ion mode. The mass scan range was *m*/*z* 20–1700, with a fragmentation voltage of 80 V. Collision energies for tandem MS (MS^2^) were set at 10, 20, and 40 V in both positive and negative ion modes.

### 4.7. Preparation of Standard Solutions

Appropriate amounts of reference standards of rutin, icariin, formononetin, astragalin, baicalin, calycosin, naringenin, hesperetin, and apigenin-7-O-β-D-glucoside were accurately weighed and dissolved in 50% methanol to prepare individual stock solutions at a concentration of 1000 μg/mL. From each stock solution, 1 mL was precisely transferred into a 10 mL volumetric flask, diluted to volume with 50% methanol, and mixed thoroughly to obtain a mixed standard stock solution containing all nine compounds.

### 4.8. Preparation of Test Solution

A 100 mg aliquot of the total flavonoid extract of SRGBHSP was placed into a 10 mL volumetric flask, dissolved in 50% methanol, and diluted to volume. After thorough mixing, the solution was centrifuged at 12,000 rpm for 10 min and filtered through a 0.22 μm membrane filter prior to analysis.

### 4.9. Chromatographic Conditions for Qualitative Identification

Chromatographic separation was carried out on a CORTECS Premier T3 column (150 mm × 2.1 mm, 1.6 μm) maintained at 30 °C. The mobile phase consisted of (A) 0.1% formic acid in water and (B) acetonitrile, with a gradient elution program as follows: 0–1 min, 50–66% B; 1–3 min, 66% B; 3–5 min, 66–68% B; 5–6 min, 68–70% B; 6–9 min, 70–50% B. The flow rate was 0.2 mL/min, and the injection volume was 4 μL.

### 4.10. Chromatographic Conditions for Quantitative Determination

Mass spectrometry was performed using a Waters XEVO TQ-S triple quadrupole mass spectrometer (Waters Corporation, Milford, MA, USA) equipped with an electrospray ionization source operating in positive ion mode with multiple reaction monitoring (MRM). The ion source voltage was set to 3.5 kV, and the source temperature was 500 °C. The desolvation gas flow rate was 1000 L/h, and the cone gas flow rate was 150 L/h.

### 4.11. Animals and Treatment

After a 7-day acclimatization period, fifty-six male Sprague–Dawley rats (200 ± 20 g) were randomly assigned to seven groups (n = 8 per group): control, model, SRGBHSP-L (1.08 g/kg), SRGBHSP-M (2.16 g/kg), SRGBHSP-H (4.32 g/kg), Yougui Pill (YGP, 0.19 g/kg), and sildenafil (5.0 mg/kg) [36]. The dose levels of SRGBHSP were selected according to our previous study [18]. The specific sequence-generation method and a formal allocation-concealment procedure were not prospectively documented. For downstream analyses using subsets, animals or biological samples were randomly selected from the corresponding experimental groups without reference to the observed outcomes. No formal blinding procedure was implemented during experimental assessment or data analysis, and no predefined exclusion criteria were established.

Except for the control group, rats received hydrocortisone (HC, 60 mg/kg; Shanghai Aladdin Biochemical Technology Co., Ltd, China) by oral gavage once daily for 10 consecutive days to induce ED-related alterations. The HC dose and duration used in the present study were selected based on preliminary experiments. Administration of SRGBHSP, YGP, or sildenafil was initiated concurrently with HC administration and continued throughout the experimental period. Rats in the control and model groups received an equivalent volume of physiological saline. Body condition and behavioral changes were monitored daily throughout the study. Two animals in the SRGBHSP-L group died unexpectedly during the experimental period, whereas no deaths occurred in the other groups. No animals were excluded after randomization on the basis of the observed experimental outcomes.

To evaluate erectile responsiveness, an apomorphine (APO)-induced erection test was performed on Day 11 after 10 consecutive days of HC administration. APO (80 μg/kg, subcutaneous injection) was administered into the loose skin of the neck, and the number of penile erections within 30 min was recorded as previously described [26].

Following the final administration and overnight fasting, rats were anesthetized with sodium pentobarbital. Blood, fecal samples, testes, epididymides, kidneys, and penile tissues were collected for subsequent analyses. Samples designated for biochemical assays and multi-omics analyses were immediately stored at −80 °C until use.

### 4.12. Histopathological Examination (H&E Staining)

The left kidneys and testes from three rats in each group were fixed in 4% paraformaldehyde, embedded in paraffin, sectioned, and stained with hematoxylin and eosin (H&E) according to standard procedures. Histopathological changes were observed and photographed using a light microscope (Leica Microsystems, Wetzlar, Germany).

### 4.13. Masson’s Trichrome Staining

Penile tissues from three rats in each group were fixed in 4% paraformaldehyde, paraffin-embedded, sectioned, and stained using a Masson’s trichrome staining kit (Beyotime Biotechnology, Shanghai, China) according to the manufacturer’s instructions. Stained sections were examined under a light microscope (Leica Microsystems, Wetzlar, Germany). The relative areas of smooth muscle and collagen fibers within the corpus cavernosum were quantitatively analyzed using Image-Pro Plus software (version 7.0, Media Cybernetics, Rockville, MD, USA), and the smooth muscle-to-collagen ratio was calculated.

### 4.14. Evaluation of the Sperm Quality

Four rats from each group were randomly selected for sperm quality assessment. The right epididymis was excised and placed in 2 mL of pre-warmed PBS at 37 °C. After incubation for 5 min, sperm suspension was prepared and analyzed using a computer-assisted sperm analysis system (CASA; WLIJY-9000, Weili New Century Science & Tech Development Co., Ltd., Beijing, China). Straight-line velocity (VSL), curvilinear velocity (VCL), average path velocity (VAP), and related kinetic parameters were determined according to the manufacturer’s instructions.

### 4.15. Assessment of Liver and Kidney Function and Reproductive Hormones

Serum samples were collected after centrifugation of whole blood. Biochemical parameters associated with hepatic and renal function were measured using an automated biochemical analyzer (CA-200Vet, URIT Medical Electronic Co., Ltd., Guilin, China). Serum concentrations of GnRH, FSH, LH, and T were determined using commercial ELISA kits according to the manufacturers’ instructions. The GnRH assay kit (F3396-B) was obtained from Shanghai Fankewi Biotechnology Co., Ltd. (Shanghai, China), whereas the FSH (MM-70867R1), LH (MM-0624R1), and T (MM-0577R1) assay kits were obtained from Jiangsu Meimian Biotechnology Co., Ltd. (Yancheng, China).

### 4.16. Assessment of Oxidative Stress Parameters and NO/GC/cGMP-Related Indicators

Serum SOD and CAT activities and the levels of MDA and GSH were determined using commercial assay kits according to the manufacturers’ instructions. Serum concentrations of NOS, NO, GC, and cGMP were determined using commercial assay kits according to the manufacturers’ instructions. The SOD and CAT assay kits, as well as the MDA (A003-1-2), GSH (A006-2-1), and NO (A013-2-1) assay kits, were obtained from Nanjing Jiancheng Bioengineering Institute (Nanjing, China). The NOS (MM-0451R1), GC (MM-71740R1), and cGMP (MM-0027R1) assay kits were obtained from Jiangsu Meimian Biotechnology Co., Ltd. (Yancheng, China). The NOS assay did not distinguish among eNOS, nNOS, and iNOS, while the GC assay measured GC concentration rather than enzymatic activity and did not specifically distinguish sGC.

### 4.17. Multi-Omics Investigation of the Mechanisms Underlying the Anti-ED Effects of SRGBHSP

#### 4.17.1. Gut Microbiota Analysis by 16S rRNA Sequencing

Fecal samples were collected aseptically and stored at −80 °C until analysis. Microbial genomic DNA was extracted using a commercial kit according to the manufacturer’s instructions. The V3–V4 region of the bacterial 16S rRNA gene was amplified and sequenced on the Illumina NovaSeq 6000 platform (Illumina Inc., San Diego, CA, USA). Subsequent bioinformatic analyses were performed by Wuhan Metware Biotechnology Co., Ltd. (Wuhan, China).

#### 4.17.2. Untargeted Metabolomic Analysis

Serum samples were subjected to untargeted metabolomic profiling using LC-MS/MS system (Thermo Fisher Scientific, Waltham, MA, USA). Metabolite extraction, chromatographic separation, mass spectrometric acquisition, and data processing were performed by Wuhan Metware Biotechnology Co., Ltd. Detailed analytical conditions are provided in Tables S3 and S4.

#### 4.17.3. DIA-Based Proteomic Analysis

Penile tissues were subjected to DIA-based proteomic analysis to identify differentially expressed proteins associated with SRGBHSP treatment. Protein extraction, enzymatic digestion, LC-MS/MS acquisition, and bioinformatic analyses were performed by Wuhan Metware Biotechnology Co., Ltd. Detailed experimental procedures are provided in Table S5.

#### 4.17.4. Transcriptomic Analysis

Total RNA was extracted from penile tissues using TRIzol reagent. RNA quality was assessed prior to library construction. Sequencing libraries were generated and sequenced on the Illumina platform by Wuhan Metware Biotechnology Co., Ltd. Differentially expressed genes were identified using standard bioinformatic pipelines.

### 4.18. Integrative Multi-Omics Analysis

Pearson correlation analysis was performed to investigate associations among gut microbial genera, differential metabolites, differentially expressed proteins, differentially expressed genes, and oxidative stress indicators. Correlations with |r| ≥ 0.8 and *p* < 0.05 were considered significant. Correlation networks were visualized using Cytoscape software (version 3.7.1, Cytoscape Consortium, San Diego, CA, USA).

### 4.19. Western Blot

Protein lysates were prepared from rat penile tissues. Equal amounts of protein were subjected to SDS-PAGE and subsequently electrotransferred onto PVDF membranes. Following blocking with 5% skim milk for 1 h, the membranes were probed overnight at 4 °C with specific primary antibodies obtained from Cell Signaling Technology (USA). And the membranes were incubated with HRP-conjugated secondary antibodies (Immunoway, Suzhou, China) for 1 h. Immunoreactive bands were visualized using an ECL detection system (TANON, Shanghai, China), and band intensities were quantified densitometrically using ImageJ software (version 1.53).

### 4.20. RNA Extraction and RT-qPCR

Total RNA was isolated from rat penile with TRIzol reagent according to the manufacturer’s instructions. cDNA was synthesized via reverse transcription, and quantitative PCR was performed using SYBR Green detection. Relative mRNA expression levels of target genes were calculated using the 2^−ΔΔCt^ method, with GAPDH employed as the endogenous control for normalization. For each experimental group, three biological replicates were analyzed, each with three technical replicates (Table S6).

### 4.21. Statistical Analysis

Data are presented as the mean ± SD. Statistical analyses were performed using SPSS 19.0 and GraphPad Prism 10.1.2. Normality was assessed using the Shapiro–Wilk test, and homogeneity of variance was evaluated using Levene’s test. For normally distributed data with homogeneous variances, comparisons among multiple groups were performed using one-way analysis of variance (ANOVA), followed by Tukey’s post hoc test. APO-induced erection frequency was analyzed using the Kruskal–Wallis test followed by Dunn’s multiple-comparisons test, with the model group used as the reference group. Longitudinal body-weight data were analyzed using two-way repeated-measures ANOVA, with time as the within-subject factor and treatment as the between-subject factor. When the assumption of sphericity was not met, the Geisser–Greenhouse correction was applied. Dunnett’s multiple-comparisons test was subsequently used to compare each group with the model group at individual time points. A two-sided *p* < 0.05 was considered statistically significant.

## 5. Conclusions

In conclusion, SRGBHSP significantly ameliorated HC-induced ED and improved reproductive function, oxidative stress status, and NO/GC/cGMP-related parameters. Chemical profiling identified 131 flavonoid compounds in SRGBHSP, with baicalin representing the predominant constituent. Integrated analyses of gut microbiota, metabolomics, transcriptomics, and DIA-based proteomics revealed coordinated alterations in gut microbial composition, glycerophospholipid metabolism, and penile molecular profiles, accompanied by changes in PI3K/Akt/mTOR-related gene and protein expression. These findings suggest that gut microbiota remodeling and metabolic reprogramming may participate in the therapeutic effects of SRGBHSP and indicate the potential involvement of PI3K/Akt/mTOR-related signaling and a gut microbiota–penis regulatory axis. Overall, this study provides a comprehensive multi-omics framework for understanding the anti-ED effects of SRGBHSP and offers scientific evidence supporting its traditional clinical application.

## Data Availability

The original contributions presented in this study are included in the article/Supplementary Materials. Further inquiries can be directed to the corresponding author.

## Supplementary Materials

The following supporting information can be downloaded at: https://www.mdpi.com/article/10.3390/ph19091456/s1, Table S1: The SRGBHSP formula comprises medicinal herbs with therapeutic potential for ED; Table S2: Detailed information regarding reference standards; Table S3: Elution process; Table S4: Q Exactive HF-X mass spectrometry conditions; Table S5: Elution process; Table S6: Primer sequence; Table S7: Identification of flavonoid constituents in SRGBHSP by UHPLC-Q-TOF-MS/MS; Table S8: Differential metabolites; Table S9: Differentially expressed proteins; Table S10: Differentially expressed genes [11,12,13,37,38,39,40,41,42,43,44,45,46,47,48,49,50,51,52,53,54,55,56,57,58,59,60,61,62].

## Abbreviations

The following abbreviations are used in this manuscript:

SRGBHSP	ShenRongGuBenHuanShao Pill
ED	Erectile dysfunction
TCM	Traditional Chinese medicine
PI3K	Phosphoinositide 3-kinase
Akt	Protein kinase B
mTOR	Mammalian target of rapamycin
HC	Hydrocortisone
UPLC-Q-TOF-MS/MS	Ultra-performance liquid chromatography coupled with quadrupole time-of-flight tandem mass spectrometry
UPLC-MS/MS	Ultra-performance liquid chromatography–tandem mass spectrometry
NO	Nitric oxide
GC	Guanylate cyclase
cGMP	Cyclic guanosine monophosphate
NOS	Nitric oxide synthase
GSH	Glutathione
ALT	Alanine aminotransferase
AST	Aspartate aminotransferase
CRE	Creatinine
GnRH	Gonadotropin-releasing hormone
HPG axis	Hypothalamic-pituitary-gonadal axis
MDA	Malondialdehyde
SOD	Superoxide dismutase
CAT	Catalase
YGP	Yougui Pill
FSH	Follicle-stimulating hormone
LH	Luteinizing hormone
T	Testosterone
VSL	Straight-line velocity
VCL	Curvilinear velocity
VAP	Average path velocity
STR	Straightness
BCF	Beat-cross frequency
LIN	Linear index
RT-PCR	Real-time quantitative polymerase chain reaction
HE	Hematoxylin & Eosin stain
