# Supplementary material for: ShenRongGuBenHuanShao Pill Ameliorates Hydrocortisone-Induced Erectile Dysfunction: Insights from Integrated Multi-Omics Analysis
Source: Pharmaceuticals (Basel). 2026 Sep 14;19(9):1456. doi: 10.3390/ph19091456 (PMC13610974; doi:10.3390/ph19091456)
Supplement: Supplementary file 1 [file pharmaceuticals-19-01456-s001.zip › pharmaceuticals-4493824-supplementary.pdf]

**Supplementary Material**

# ShenRongGuBenHuanShao Pill Ameliorates Hydrocortisone-Induced Erectile Dysfunction through Modulation of the PI3K/Akt/mTOR and NO/GC/cGMP Signaling Pathways

**Contents:**

**SRGBHSP Prescription Composition**

**Table S1** The SRGBHSP formula comprises medicinal herbs with therapeutic potential for ED

**Table S2** Detailed information regarding reference standards

**Table S3** Elution process

**Table S4** Q Exactive HF-X mass spectrometry conditions

**Table S5** Elution process

**Table S6** Primer sequence

**Table S7** Identification of flavonoid constituents in SRGBHSP by UHPLC-Q-TOF-MS/MS

**Table S8** Differential metabolites

**Table S9** Differentially expressed proteins

**Table S10** Differentially expressed genes

**SRGBHSP Prescription Composition:**

SRGBHSP is composed of the following 65 TCMs: *Rehmannia glutinosa* (Gaertn.) Libosch. ex DC., *Glycine max* (L.) Merr., *Colla Corii Asini, Cartilaginous core* of velvet antler, *Tribulus terrestris* L., *Achyranthes bidentata* Blume., *Aconitum carmichaelii* Debx., *Cuscuta chinensis* Lam., *Dioscorea oppositifolia* L., W*olfiporia cocos*, *Fallopia multiflora* (Thunb.) Haraldson., *Lycium barbarum* L., *Angelica sinensis* (Oliv.) Diels., *Crataegus pinnatifida* Bunge., *Schisandra chinensis* (Turcz.) Baill., *Amomum villosum* Lour., *Chaenomeles speciosa* (Sweet) Nakai., *Foeniculum vulgare* Mill., *Cinnamomum cassia* Presl., *Epimedium brevicornu* Maxim., *Cistanche deserticola* Y.C.Ma., *Astragalus membranaceus* (Fisch.) Bunge., *Codonopsis pilosula* (Franch.) Nannf., *Atractylodes macrocephala* Koidz., *Rehmannia glutinosa* (Libosch.) DC., *Nelumbo nucifera* Gaertn., Os Draconis (Long-Gu), *Chrysanthemum morifolium* Ramat., *Hordeum vulgare* L., Liu Shen Qu, Fish Maw, *Fritillaria thunbergii* Miq., *Pheretima aspergillum* (E. Perrier)., *Mylabris phalerata* Pallas., *Scutellaria baicalensis* Georgi., *Eriocheir*

*sinensis* H. Milne-Edwards., *Glycyrrhiza uralensis* Fisch., *Eucommia ulmoides* Oliv., *Psoralea corylifolia* L., *Conioselinum anthriscoides* 'Chuanxiong', *Curculigo orchioides* Gaertn., *Morinda officinalis* F.C. How., *Cyathula officinalis* K.C.Kuan., *Cornus officinalis* Siebold & Zucc., *Paeonia lactiflora* Pall., *Panax ginseng* C.A.Mey., *Cervus nippon.,* Actinolite (Yang-Qi-Shi), Actinolite (Yin-Qi-Shi), Tortoise Carapace and Plastron, Testudinis Carapax et Plastrum Colla, *Ophiopogon japonicus* (L.f.) Ker Gawl., *Asparagus cochinchinensis* (Lour.) Merr., *Eclipta prostrata* (L.) L.*, Platycladus orientalis* (L.) Franco., *Polygala tenuifolia* Willd., *Aucklandia costus* Falc., *Citrus reticulata* Blanco., *Zanthoxylum bungeanum* Maxim., *Syzygium aromaticum* (L.) Merr. & L.M.Perry., *Sinapis alba* L., *Pinellia ternata* (Thunb.) Makino., Borax, Cinnabar, *Hippocampus japonicus* Kaup.

**Statement on Plant Name Curation:** The plant name has been checked with http://mpns.kew.org, mentioning the date of accessing that website.

**Table S1.** The SRGBHSP formula comprises medicinal herbs with therapeutic potential for ED

| NO. | Herb | References |
|---|---|---|
| 1 | *Colla Corii Asini* | 37 |
| 2 | *Psoralea corylifolia* L. | 38 |
| 3 | *Ligusticum chuanxiong* hort. | 39 |
| 4 | *Ligusticum chuanxiong* hort.+ *Paeonia lactiflora* Pall. | 40 |
| 5 | *Angelica sinensis* (Oliv.) Diels. | 41 |
| 6 | *Rehmannia glutinosa* (Libosch.) DC. | 42 |
| 7 | *Pheretima aspergillum* (E. Perrier) | 43 |
| 8 | *Aconitum carmichaelii* Debx. | 44 |
| 9 | *Hippocampus japonicus* Kaup. | 45 |
| 10 | *Fallopia multiflora* (Thunb.) Haraldson. | 46 |
| 11 | *Zanthoxylum bungeanum* Maxim. | 47 |
| 12 | *Astragalus membranaceus* (Fisch.) Bunge. | 48 |
| 13 | *Tribulus terrestris* L. | 49 |
| 14 | Os Draconis (Long-Gu) | 50 |
| 15 | *Cervus nippon* | 51 |
| 16 | *Syzygium aromaticum* (L.) Merr. & L.M.Perry. | 52 |
| 17 | *Achyranthes bidentata* Blume. | 53 |
| 18 | *Panax ginseng* C.A.Mey. | 54 |
| 19 | *Cistanche deserticola* Y.C.Ma. | 13 |
| 20 | *Cinnamomum cassia* Presl. | 11 |
| 21 | *Dioscorea opposite* Thunb. + *Crataegus pinnatifida* Bge. | 55 |
| 22 | *Cornus officinalis* Siebold & Zucc. + *Rehmannia glutinosa* (Gaertn.) Libosch. ex DC. | 56 |

| | | |
|---|---|---|
| 23 | *Curculigo orchioides* Gaertn. | 57 |
| 24 | *Curculigo orchioides* Gaertn. + *Epimedium brevicornu* Maxim. | 58 |
| 25 | *Foeniculum vulgare* Mill. | 59 |
| 26 | Actinolite (Yang-Qi-Shi) | 60 |
| 27 | *Epimedium brevicornu* Maxim. | 12 |
| 28 | *Epimedium brevicornu* Maxim. + *Morinda officinalis* F.C. How. | 61 |
| 29 | *Polygala tenuifolia* Willd. | 62 |

**Table S2.** Detailed information regarding reference standards

| Name | Batch NO. | Purity/≥ | Source |
|---|---|---|---|
| Hesperetin | 130816 | 98% | Chengdu Purifa Technology Co., Ltd. |
| Baicalin | 131109 | 98% | Chengdu Purifa Technology Co., Ltd. |
| Naringenin | 130330 | 98% | Chengdu Purifa Technology Co., Ltd. |
| Formononetin | 20160519 | 98% | Baoji Chenguang Biotechnology Co., Ltd. |
| Calycosin | HC012084198 | 98% | Baoji Chenguang Biotechnology Co., Ltd. |
| Icariin | WP23080108 | 98% | Sichuan Weikeqi Biotechnology Co., Ltd. |
| Rutin | 153-18-4 | 98% | Sichuan Weikeqi Biotechnology Co., Ltd. |
| Apigenin-7-O-β-D-glucoside | | 98% | self-prepared |
| Astragalin | 14-2009 | 98% | Shanghai Research Center for Standardization of Traditional Chinese Medicine |

**Table S3.** Elution process

| Time (min) | A (%) | B (%) |
|---|---|---|
| 0.0 | 95 | 5 |
| 2.0 | 80 | 20 |
| 5.0 | 40 | 60 |
| 6.0 | 1 | 99 |
| 7.5 | 1 | 99 |
| 7.6 | 95 | 5 |
| 10.0 | 95 | 5 |

**Table S4.** Q Exactive HF-X mass spectrometry conditions

| Name | ESI+ | ESI- |
|---|---|---|
| Spray Voltage | 3500 | 3200 |
| Sheath gas (Arb) | 30 | 30 |
| Aux gas (Arb) | 5 | 5 |
| Ion transfer tube temperature | 320 | 320 |
| Vaporizer temperature | 300 | 300 |
| Scan Range | 75-1000 | 75-1000 |
| Resolution | 35000 | 35000 |
| AGC target | $1\times10^{6}$ | $1\times10^{6}$ |
| RF lens (%) | 50 | 50 |
| Scan Range | 75-1000 | 75-1000 |

**Table S5.** Elution process

| Time (min) | A (%) | B (%) |
|---|---|---|
| 0.0 | 95 | 5 |
| 4.0 | 80 | 20 |
| 5.8 | 40 | 60 |
| 6.2 | 1 | 99 |
| 6.9 | 1 | 99 |

**Table S6.** Primer sequence

| Name | Forward primer | Reverse primer |
|---|---|---|
| PI3K | GAAACCCAGTCACCTAGGGC | GGTGGGCAGTACGAACTCAA |
| Akt | TCACCTCTGAGACCGACACC | ACTGGCTGAGTAGGAGAACTGG |
| mTOR | AGAAACTGCACGTCAGCACCA | CCATTCCAGCCAGTCATCTTTG |
| GAPDH | GGGTCCCAGCTTAGGTTCATC | TGAGGTCAATGAAGGGGTCG |

**TableS7.** Identification of flavonoid constituents in SRGBHSP by UHPLC-Q-TOF-MS/MS

| NO. | $t_R$/min | Compounds | Molecular formula | Quasi-molecular ion peak | Theoretical value | Measured value | Mass accuracy (ppm) | MS/MS （*m/z*） |
|---|---|---|---|---|---|---|---|---|
| 1 | 5.827 | Apiin | $C_{26}H_{28}O_{14}$ | $[M-H]^-$ | 563.1406 | 563.1397 | 1.65 | 563.1397,269.0457 |
| 2 | 6.485 | Peltatoside | $C_{26}H_{28}O_{16}$ | $[M-H]^-$ | 595.1305 | 595.1280 | 4.12 | 300.0270,301.0350,271.0230 |
| 3 | 6.856 | Rutin | $C_{27}H_{30}O_{16}$ | $[M-H]^-$ | 609.1461 | 609.1455 | 1.00 | 609.1455,300.0266,178.9982 |
| 4 | 6.981 | Luteolin-7-O-β-D-glucuronide | $C_{21}H_{18}O_{12}$ | $[M-H]^-$ | 461.0725 | 461.0723 | 0.54 | 285.0405,286.0433,287.0550 |
| 5 | 7.010 | Liquiritin apioside | $C_{26}H_{30}O_{13}$ | $[M-H]^-$ | 549.1614 | 549.1595 | 3.39 | 417.1173,255.0567,135.0081 |
| 6 | 7.195 | Hyperoside | $C_{21}H_{20}O_{12}$ | $[M-H]^-$ | 463.0882 | 463.0878 | 0.86 | 465.0904,464.0884,463.0878,303.0372,302.0371,301.0342,300.0269 |
| 7 | 7.197 | Isoquercitrin | $C_{21}H_{20}O_{12}$ | $[M-H]^-$ | 463.0882 | 463.0878 | 0.86 | 151.0051,178.9993,300.0269 |
| 8 | 7.205 | Quercetin | $C_{15}H_{10}O_7$ | $[M+H]^+$ | 303.0499 | 303.0502 | -0.90 | 285.0335,229.0513,153.0187 |
| 9 | 7.218 | Liquiritin | $C_{21}H_{22}O_9$ | $[M-H]^-$ | 417.1191 | 417.1196 | -1.18 | 255.0656,135.0087,119.0497 |
| 10 | 7.458 | Dihydrobaicalin | $C_{21}H_{20}O_{11}$ | $[M+H]^+$ | 449.1078 | 449.1080 | -0.36 | 287.0558 |
| 11 | 7.463 | Astragalin | $C_{21}H_{20}O_{11}$ | $[M+H]^+$ | 449.1078 | 449.1080 | -0.36 | 287.0558,288.0586 |
| 12 | 7.629 | Chrysin 6-C-glucoside 8-C-arabinoside | $C_{26}H_{28}O_{13}$ | $[M+H]^+$ | 549.1603 | 549.1597 | 1.04 | 531.1504,513.1394,495.1286,411.1068 |
| 13 | 8.034 | Luteolin-7-glucoside | $C_{21}H_{20}O_{11}$ | $[M-H]^-$ | 447.0933 | 447.0922 | 2.42 | 285.0390,284.0306,255.0281,227.0334 |
| 14 | 8.729 | Isorhamnetin-3-O-beta-D-Glucoside | $C_{22}H_{22}O_{12}$ | $[M-H]^-$ | 477.1038 | 477.1034 | 0.94 | 314.0403,300.0303,270.3483 |
| 15 | 8.737 | Kaempferol | $C_{15}H_{10}O_6$ | $[M+H]^+$ | 287.0550 | 287.0552 | -0.65 | 121.0306,153.0180 |
| 16 | 9.017 | Puerarin | $C_{21}H_{20}O_9$ | $[M-H]^-$ | 415.1035 | 415.1016 | 4.46 | 295.0604,267.0658 |
| 17 | 9.186 | Apigenin-7-O-glucoside | $C_{21}H_{20}O_{10}$ | $[M+H]^+$ | 433.1129 | 433.1141 | -2.72 | 271.0608,272.0638,273.0659 |
| 18 | 9.203 | Galangin | $C_{15}H_{10}O_5$ | $[M+H]^+$ | 271.0601 | 271.0603 | -0.74 | 153.0188,119.0501,229.0491 |
| 19 | 9.399 | Hesperidin | $C_{28}H_{34}O_{15}$ | $[M-H]^-$ | 609.1825 | 609.1805 | 3.27 | 301.0713,325.0695609.1805 |

| | | | | | | | | |
|---|---|---|---|---|---|---|---|---|
| 20 | 9.514 | Homoeriodictyol | $C_{16}H_{14}O_6$ | $[M+H]^+$ | 303.0863 | 303.0864 | -0.28 | 177.0551,153.0185,145.0294,285.0792 |
| 21 | 9.932 | 6''-O-Acetyldaidzin | $C_{23}H_{22}O_{10}$ | $[M+H]^+$ | 459.1286 | 459.1273 | 2.78 | 255.0658 |
| 22 | 10.189 | Peonidin-3-O-beta-galactoside | $C_{22}H_{23}O_{11}^+$ | $[M]^+$ | 463.1235 | 463.1222 | 2.79 | 301.0711,201.0532 |
| 23 | 10.284 | Diosmetin-7-O-β-D-glucopyranoside | $C_{22}H_{22}O_{11}$ | $[M+H]^+$ | 463.1235 | 463.1222 | 2.79 | 301.0711 |
| 24 | 10.674 | Artocarpanone | $C_{16}H_{14}O_6$ | $[M+H]^+$ | 303.0863 | 303.0867 | -1.28 | 285.0784,257.0808,229.0860 |
| 25 | 10.977 | Baicalin | $C_{21}H_{18}O_{11}$ | $[M+H]^+$ | 447.0922 | 447.0927 | -1.15 | 271.0603,447.0927 |
| 26 | 11.636 | Scutellarein | $C_{15}H_{10}O_6$ | $[M-H]^-$ | 285.0405 | 285.0396 | 3.01 | 117.0343,119.0498,136.9898,139.0028 |
| 27 | 11.661 | 7,2'-Dihydroxyflavone | $C_{15}H_{10}O_4$ | $[M-H]^-$ | 253.0506 | 253.0497 | 3.67 | 117.0338,91.0184,135.0088 |
| 28 | 11.764 | Isoliquiritoside | $C_{21}H_{22}O_9$ | $[M-H]^-$ | 417.1185 | 417.1191 | 1.45 | 255.0654,135.0079,119.0495 |
| 29 | 11.773 | Isoflavone base + 1O, 1MeO, O-Hex+$C_7H_{12}NO$ | $C_{29}H_{33}NO_{10}$ | $[M+H]^+$ | 556.2177 | 556.2176 | 0.22 | 54.0352,269.0810,288.1443 |
| 30 | 11.815 | Tectochrysin | $C_{16}H_{12}O_4$ | $[M+H]^+$ | 269.0808 | 269.0809 | -0.24 | 254.0570,237.0551,213.0913,226.0629 |
| 31 | 11.857 | Ononin | $C_{22}H_{22}O_9$ | $[M+H]^+$ | 431.1337 | 431.1336 | 0.14 | 269.0815 |
| 32 | 12.401 | Dihydrobaicalein-7-O-glucuronide | $C_{21}H_{20}O_{11}$ | $[M-H]^-$ | 447.0933 | 447.0901 | 7.11 | 271.0604,243.0623,113.0235 |
| 33 | 12.542 | Luteolin 7-(6''-acetylglucoside) | $C_{23}H_{22}O_{12}$ | $[M-H]^-$ | 489.1038 | 489.1028 | 2.14 | 285.0403,162.8379 |
| 34 | 12.709 | Licochalcone B | $C_{16}H_{14}O_5$ | $[M-H]^-$ | 285.0768 | 285.0762 | 2.26 | 150.0315,270.0522,177.0182 |
| 35 | 12.826 | Daidzein | $C_{15}H_{10}O_4$ | $[M+H]^+$ | 255.0652 | 255.0654 | -0.85 | 255.0654,237.0546,227.0707,209.0601,199.0758 |
| 36 | 12.934 | Isosakuranin | $C_{16}H_{14}O_5$ | $[M+H]^+$ | 287.0914 | 287.0913 | 0.35 | 269.0815,254.0475,209.0595 |
| 37 | 13.396 | Liquiritigenin | $C_{15}H_{12}O_4$ | [M-H]- | 255.0663 | 255.0658 | 1.88 | 119.0500,135.0084 |
| 38 | 13.477 | Viscidulin III | $C_{17}H_{14}O_8$ | $[M+H]^+$ | 347.0761 | 347.0770 | -2.47 | 332.0539,314.0408,169.0121 |
| 39 | 13.490 | Axillarin | $C_{17}H_{14}O_8$ | $[M+H]^+$ | 347.0761 | 347.0768 | -1.90 | 301.0344,332.0536,273.0386 |
| 40 | 13.679 | Sissotrin | $C_{22}H_{22}O_{10}$ | $[M-H]^-$ | 445.1140 | 445.1150 | -2.20 | 267.0294,283.0604,430.0877 |
| 41 | 13.935 | Hispidulin 7-glucuronide | $C_{22}H_{20}O_{12}$ | $[M-H]^-$ | 475.0882 | 475.0867 | 3.15 | 299.0552,284.0321,113.0240 |
| 42 | 13.966 | 6''-O-Acetylgenistin | $C_{23}H_{22}O_{11}$ | $[M+H]^+$ | 475.1235 | 475.1236 | -0.24 | 271.0605,187.0598 |
| 43 | 14.091 | Chrysin-7-O-Beta-D-glucoronide | $C_{21}H_{18}O_{10}$ | $[M-H]^-$ | 429.0827 | 429.0811 | 3.77 | 253.0496,113.0240,85.0292,59.0144 |

| | | | | | | | | |
|---|---|---|---|---|---|---|---|---|
| 44 | 14.241 | Baicalein-7-O-β-D- methyl glucopyrano siduronic acid | $C_{22}H_{20}O_{11}$ | $[M+Na]^{+}$ | 483.0898 | 483.0905 | -1.56 | 285.0767,270.0530 |
| 45 | 14.525 | Diosmetin 7-glucuronide | $C_{22}H_{20}O_{12}$ | $[M-H]^{-}$ | 475.0882 | 475.0855 | 5.67 | 284.0321,299.0551,71.0131 |
| 46 | 14.840 | Acacetin | $C_{16}H_{12}O_{5}$ | $[M+H]^{+}$ | 285.0757 | 285.0763 | -1.94 | 153.0189,213.0547,242.0574,270.0530 |
| 47 | 14.853 | 6-Methoxyluteolin | $C_{16}H_{12}O_{7}$ | $[M-H]^{-}$ | 315.0510 | 315.0502 | 2.61 | 201.0180,133.0288,227.0338173.0239 |
| 48 | 14.857 | Jaceidin | $C_{18}H_{16}O_{8}$ | $[M+H]^{+}$ | 361.0918 | 361.0922 | -1.13 | 346.0680,331.0458,328.0552 |
| 49 | 14.885 | Luteolin | $C_{15}H_{10}O_{6}$ | $[M-H]^{-}$ | 285.0405 | 285.0399 | 2.66 | 257.0450,241.0484,151.0030,133.0288 |
| 50 | 14.911 | Isorhamnetin | $C_{16}H_{12}O_{7}$ | $[M-H]^{-}$ | 315.0510 | 315.0505 | 1.67 | 315.0505,300.0265 |
| 51 | 15.025 | Oroxylin A-7-O-glucuronide | $C_{22}H_{20}O_{11}$ | $[M-H]^{-}$ | 459.0933 | 459.0932 | 0.18 | 283.0606,268.0359,113.0239 |
| 52 | 15.107 | Isosakuranetin-7-rutinoside | $C_{28}H_{34}O_{14}$ | $[M-H]^{-}$ | 593.1876 | 593.1853 | 3.84 | 285.0765,147.0442,547.2185 |
| 53 | 15.355 | Calycosin | $C_{16}H_{12}O_{5}$ | $[M+H]^{+}$ | 285.0757 | 285.0759 | -0.18 | 137.0237,225.0533,270.0527 |
| 54 | 15.864 | Wogonoside | $C_{22}H_{20}O_{11}$ | $[M+Na]^{+}$ | 483.0898 | 483.0905 | -1.56 | 285.0768,270.0529,252.0375 |
| 55 | 16.023 | Apigenin 7-(6”-O-acetyl)-glucoside | $C_{23}H_{22}O_{11}$ | $[M+H]^{+}$ | 475.1235 | 475.1237 | -0.45 | 271.0609,188.0703,129.0717 |
| 56 | 16.208 | Apigenin-7-arabinopyranoside | $C_{20}H_{18}O_{9}$ | $[M+H]^{+}$ | 403.1024 | 403.1020 | 0.89 | 271.06,123.0070 |
| 57 | 16.228 | Wogonin-5-O-D-glucoside | $C_{22}H_{22}O_{10}$ | $[M+H]^{+}$ | 447.1286 | 447.1294 | -1.85 | 242.0588,271.0598,285.0761 |
| 58 | 16.576 | Genistin | $C_{21}H_{20}O_{10}$ | $[M-H]^{-}$ | 431.0984 | 431.0976 | 1.78 | 269.0454,225.0536,311.0550 |
| 59 | 16.596 | Epmedin B | $C_{38}H_{48}O_{19}$ | $[M+H]^{+}$ | 809.2863 | 809.2866 | -0.43 | 677.2438,531.1862,369.1339 |
| 60 | 16.633 | Acacetin 7-O-glucoside | $C_{22}H_{22}O_{10}$ | $[M+H]^{+}$ | 447.1286 | 447.1291 | -1.18 | 285.0766 |
| 61 | 17.262 | Anhydroicaritin 3-rhamnosyl-(1->2)-rhamnoside | $C_{33}H_{40}O_{14}$ | $[M-H]^{-}$ | 659.2345 | 659.2332 | 2.01 | 366.1104,323.0916 |
| 62 | 17.461 | Cirsimaritin | $C_{17}H_{14}O_{6}$ | $[M+H]^{+}$ | 315.0863 | 315.0875 | -3.77 | 300.0619,285.0411,282.0548,195.0317 |
| 63 | 17.486 | Epmedin C | $C_{39}H_{50}O_{19}$ | $[M+H]^{+}$ | 823.3019 | 823.3014 | 0.62 | 677.2442,531.1869,515.1900,369.1339,313.0717 |
| 64 | 17.948 | Icariin | $C_{33}H_{40}O_{15}$ | $[M+H]^{+}$ | 677.2440 | 677.2438 | 0.29 | 531.1877,369.1342,313.0718 |
| 65 | 18.194 | Luteolin 4’-methyl ether 7-glucoside | $C_{22}H_{22}O_{11}$ | $[M-H]^{-}$ | 461.1089 | 461.1087 | 0.51 | 299.0566,284.0320,255.0334 |
| 66 | 18.374 | Apigenin | $C_{15}H_{10}O_{5}$ | $[M-H]^{-}$ | 269.0455 | 269.0454 | 0.54 | 117.0345,151.0037,225.0552 |

| | | | | | | | | |
|---|---|---|---|---|---|---|---|---|
| 67 | 18.452 | Baicalin methyl ester | $C_{22}H_{20}O_{11}$ | $[M+H]^+$ | 461.1078 | 461.1093 | -3.18 | 271.0608,225.0552,169.0133 |
| 68 | 19.032 | Hesperetin | $C_{16}H_{14}O_6$ | $[M+H]^+$ | 303.0863 | 303.0864 | -0.28 | 153.0186,177.0547,261.0701,285.0738 |
| 69 | 19.045 | Naringenin | $C_{15}H_{12}O_5$ | $[M+H]^+$ | 273.0757 | 273.0765 | -2.76 | 147.045 |
| 70 | 19.111 | Icariside I | $C_{27}H_{30}O_{11}$ | $[M+H]^+$ | 531.1861 | 531.1856 | 0.92 | 313.0708,369.1338 |
| 71 | 19.146 | Genistein | $C_{15}H_{10}O_5$ | $[M+H]^+$ | 271.0601 | 271.0611 | -3.70 | 153.0188,215.0731,243.0659 |
| 72 | 19.527 | Scutellarein 4'-methyl ether | $C_{16}H_{12}O_6$ | $[M+H]^+$ | 301.0707 | 301.0711 | -1.45 | 168.0053,84.0210 |
| 73 | 19.670 | Hydrate Isobavachin Hydrate | $C_{20}H_{22}O_5$ | $[M-H]^-$ | 341.1394 | 341.1374 | 5.98 | 221.0809,119.0499 |
| 74 | 19.858 | Baicalein | $C_{15}H_{10}O_5$ | $[M-H]^-$ | 269.0455 | 269.0454 | 0.54 | 117.0345,151.0037,225.0556 |
| 75 | 20.025 | Dehydroapigenin | $C_{15}H_8O_5$ | $[M-H]^-$ | 267.0299 | 267.0290 | 3.35 | 117.0347,151.0029,211.0399 |
| 76 | 20.216 | Sorbifolin | $C_{16}H_{12}O_6$ | $[M-H]^-$ | 299.0561 | 299.0549 | 4.04 | 284.0322,301.0626,136.9880 |
| 77 | 20.326 | Biochanin A | $C_{16}H_{12}O_5$ | $[M-H]^-$ | 283.0612 | 283.0609 | 1.05 | 211.0396,195.0454,167.0509,239.0326 |
| 78 | 20.373 | Formononetin 7-O-(6”-acetylglcoside) | $C_{24}H_{24}O_{10}$ | $[M+H]^+$ | 473.1442 | 473.1458 | -3.34 | 269.0819 |
| 79 | 20.509 | 6”-O-Acetylglycitin | $C_{24}H_{24}O_{11}$ | $[M+H]^+$ | 489.1391 | 489.1388 | 0.69 | 270.0521,285.0761 |
| 80 | 21.093 | Natsudaidain-3-O-(3-hydroxy-3-methylglutaric acid)-Glu | $C_{33}H_{40}O_{18}$ | $[M+H]^+$ | 725.2287 | 725.2289 | -0.22 | 419.1350,389.0873 |
| 81 | 21.804 | Glycitein | $C_{16}H_{12}O_5$ | $[M-H]^-$ | 283.0612 | 283.0616 | -1.42 | 240.0429,211.0381,268.0384,196.0517 |
| 82 | 21.821 | Chrysoeriol | $C_{16}H_{12}O_6$ | $[M-H]^-$ | 299.0561 | 299.0567 | -1.96 | 284.0327,285.0361,256.0372 |
| 83 | 22.132 | Apigenin 7-(6”-crotonylglucoside) | $C_{25}H_{24}O_{11}$ | $[M+H]^+$ | 501.1391 | 501.1387 | 0.88 | 271.0607,154.0499 |
| 84 | 22.575 | Baicalein-7-O-β-D-ethylglucuronide | $C_{23}H_{22}O_{11}$ | $[M-H]^-$ | 473.1089 | 473.1088 | 0.28 | 269.0459,243.1210 |
| 85 | 22.600 | Baicalein 7-O-beta-D-ethylglucuronide | $C_{23}H_{22}O_{11}$ | $[M+H]^+$ | 475.1235 | 475.1234 | 0.19 | 271.0608,159.0292,113.0237 |
| 86 | 22.637 | Eurycarpin A | $C_{20}H_{18}O_5$ | $[M-H]^-$ | 337.1081 | 337.1086 | -1.34 | 265.0499,266.0547,277.1847 |
| 87 | 22.929 | Apigenin 6,8-di-C-alpha-L-arabinopyranoside | $C_{25}H_{26}O_{13}$ | $[M+H]^+$ | 535.1446 | 535.1450 | -0.72 | 331.0817,316.0578 |
| 88 | 23.347 | Quercetin 3,7-dimethyl ether | $C_{17}H_{14}O_7$ | $[M+H]^+$ | 331.0812 | 331.0814 | -0.52 | 316.0578,301.0343 |
| 89 | 23.962 | Wogonin | $C_{16}H_{12}O_5$ | $[M-H]^-$ | 283.0612 | 283.0616 | -1.42 | 240.0429,268.0384 |

| | | | | | | | | |
|---|---|---|---|---|---|---|---|---|
| 90 | 24.065 | Hispidulin | $C_{16}H_{12}O_6$ | $[M-H]^-$ | 299.0561 | 299.0565 | -1.29 | 284.0327,165.9909,137.9963,211.0373 |
| 91 | 24.307 | Isosinensetin | $C_{20}H_{20}O_7$ | $[M+H]^+$ | 343.1282 | 343.1284 | -0.59 | 343.0820,358.1047,315.0868,181.0139 |
| 92 | 24.601 | Tricin | $C_{17}H_{14}O_7$ | $[M+H]^+$ | 331.0812 | 331.0811 | 0.39 | 301.0343,298.0472 |
| 93 | 25.003 | Isoliquiritigenin | $C_{15}H_{12}O_4$ | [M-H]- | 255.0663 | 255.0664 | -0.46 | 119.0500,135.0084 |
| 94 | 25.037 | 5, 7-Dihydroxyflavanone | $C_{15}H_{12}O_4$ | $[M+H]^+$ | 257.0808 | 257.0810 | -0.64 | 137.0239,147.0445,119.0499 |
| 95 | 25.420 | Formononetin | $C_{16}H_{12}O_4$ | $[M+H]^+$ | 269.0808 | 269.0815 | -2.48 | 118.0410,213.0906 |
| 96 | 26.369 | 5,7-Dihydroxy-3’,4’,5’-trimethoxyflavanone | $C_{18}H_{18}O_7$ | $[M+H]^+$ | 347.1125 | 347.1131 | -1.65 | 317.0660,286.0835,218.0582 |
| 97 | 26.733 | Trimethoxyflavone | $C_{18}H_{16}O_5$ | $[M+H]^+$ | 313.1071 | 313.1069 | 0.48 | 283.0600,255.0636,181.0134 |
| 98 | 27.147 | Sinensetin | $C_{20}H_{20}O_7$ | $[M+H]^+$ | 373.1282 | 373.1281 | 0.21 | 373.1281,343.0820,312.0996,329.1025,358.1046 |
| 99 | 27.480 | tetramethoxyflavone | $C_{19}H_{18}O_6$ | $[M+H]^+$ | 343.1176 | 343.1180 | -1.13 | 313.0710,285.0750,181.0130,153.0192 |
| 100 | 28.474 | Irigenin | $C_{18}H_{16}O_8$ | $[M+H]^+$ | 361.0918 | 361.0925 | -1.96 | 331.0448,303.0484,285.0353 |
| 101 | 28.819 | Sagittatoside A | $C_{33}H_{40}O_{15}$ | $[M-H]^-$ | 675.2294 | 675.2285 | 1.40 | 352.0932,367.1165,323.0914 |
| 102 | 29.228 | 7-methylapigeninidin | $C_{16}H_{12}O_5$ | $[M+H]^+$ | 285.0757 | 285.0764 | -2.29 | 270.0526 |
| 103 | 29.750 | Dihydroxy-dimethoxyflavone | $C_{17}H_{14}O_6$ | $[M+H]^+$ | 315.0863 | 315.0872 | -2.82 | 300.0644,254.0587,197.0602, |
| 104 | 29.803 | Nobiletin | $C_{21}H_{22}O_8$ | $[M+H]^+$ | 403.1387 | 403.1388 | -0.14 | 387.1077,373.0917,355.0803,327.0843 |
| 105 | 29.867 | Sagittatosdie B | $C_{32}H_{38}O_{14}$ | $[M+H]^+$ | 647.2334 | 647.2348 | -2.12 | 369.1337,325.0716,313.0713,405.0962 |
| 106 | 29.953 | Tetramethyl-o-scutellarein | $C_{19}H_{18}O_6$ | $[M+H]^+$ | 343.1176 | 343.1178 | -0.54 | 327.0873,313.0710,309.0761,299.0916,285.0757,267.0654,181.0131,153.0189,135.0450 |
| 107 | 30.089 | 2’-O-rhamnosylicariside II | $C_{33}H_{40}O_{14}$ | $[M+H]^+$ | 661.2491 | 661.2499 | -1.24 | 313.0725,369.1339,411.1435,515.1917 |
| 108 | 30.275 | Genkwanin | $C_{16}H_{12}O_5$ | $[M-H]^-$ | 283.0612 | 283.0611 | 0.34 | 268.0374,269.0409,239.0253 |
| 109 | 30.312 | Skullcapflavone II | $C_{19}H_{18}O_8$ | $[M+H]^+$ | 375.1074 | 375.1084 | -2.56 | 169.0145,197.0088,227.0557,327.0506,345.0612 |
| 110 | 30.676 | Licochalcone D | $C_{21}H_{22}O_5$ | $[M-H]^-$ | 353.1394 | 353.1379 | 4.37 | 150.0325,338.1136,177.0194 |
| 111 | 30.813 | Persicogenin | $C_{17}H_{16}O_6$ | $[M+H]^+$ | 317.1020 | 317.1026 | -2.01 | 169.1072,287.0554,302.0788 |

| | | | | | | | | |
|---|---|---|---|---|---|---|---|---|
| 112 | 31.373 | Baohuoside I | $C_{27}H_{30}O_{10}$ | $[M-H]^-$ | 513.1766 | 513.1760 | 1.21 | 513.1760,366.1103 |
| 113 | 31.427 | heptamethoxyflavone | $C_{22}H_{24}O_9$ | $[M+H]^+$ | 433.1493 | 433.1508 | -3.45 | 403.1033,385.0921,357.0971 |
| 114 | 31.507 | Neobavaisoflavone | $C_{20}H_{18}O_4$ | $[M-H]^-$ | 321.1132 | 321.1127 | 1.65 | 265.0604,277.0501,252.0423 |
| 115 | 31.560 | Gancaonin N | $C_{21}H_{20}O_6$ | $[M+H]^+$ | 369.1333 | 369.1336 | -0.91 | 313.0717 |
| 116 | 31.726 | Licoflavone A | $C_{20}H_{18}O_4$ | $[M+H]^+$ | 323.1278 | 323.1289 | -3.46 | 267.0662,255.0661,239.0710 |
| 117 | 32.079 | Licocoumarone | $C_{20}H_{20}O_5$ | $[M+H]^+$ | 341.1384 | 341.1393 | -2.79 | 285.0767,149.0243 |
| 118 | 32.242 | Tangeretin | $C_{20}H_{20}O_7$ | $[M+H]^+$ | 373.1282 | 373.1287 | -1.40 | 358.1077,344.0865,343.0812,325.0696,297.0775 |
| 119 | 32.528 | Bavachin | $C_{20}H_{20}O_4$ | $[M-H]^-$ | 323.1289 | 323.1287 | 0.56 | 119.0501,203.0710 |
| 120 | 32.571 | Glyasperin C | $C_{21}H_{24}O_5$ | $[M+H]^+$ | 357.1697 | 357.1704 | -2.10 | 301.1080,221.1177,165.0553,123.0450 |
| 121 | 33.075 | Kanzonol-M | $C_{23}H_{26}O_6$ | $[M+H]^+$ | 399.1802 | 399.1804 | -0.46 | 384.1606,368.1635,353.1387,337.1440,330.1106 |
| 122 | 33.594 | Licoisoflavone A | $C_{20}H_{18}O_6$ | $[M+H]^+$ | 355.1176 | 355.1177 | -0.24 | 299.0566,287.0563,243.1572 |
| 123 | 33.613 | Glabridin | $C_{20}H_{20}O_4$ | $[M+H]^+$ | 325.1434 | 325.1436 | -0.51 | 149.0242,205.0862,269.0816 |
| 124 | 34.302 | Demethylnobiletin | $C_{20}H_{20}O_8$ | $[M+H]^+$ | 389.1231 | 389.1243 | -3.11 | 169.0133,197.0077,359.0766,341.0663 |
| 125 | 34.906 | Licoflavone C | $C_{20}H_{18}O_5$ | $[M+H]^+$ | 339.1227 | 339.1220 | 2.07 | 283.0611,271.0608,284.0635 |
| 126 | 37.822 | Isobavachin | $C_{20}H_{20}O_4$ | $[M-H]^-$ | 323.1289 | 323.1284 | 1.49 | 119.0502,203.0714 |
| 127 | 38.558 | Licochalcone A | $C_{21}H_{22}O_4$ | $[M-H]^-$ | 337.1445 | 337.1437 | 2.46 | 337.1437,322.1184,305.1157,93.0350 |
| 128 | 38.728 | Bavachinin | $C_{21}H_{22}O_4$ | $[M+H]^+$ | 339.1591 | 339.1598 | -2.11 | 256.0716,219.1018,147.0450,119.0497 |
| 129 | 39.222 | Isoangustone A | $C_{25}H_{26}O_6$ | $[M+H]^+$ | 423.1802 | 423.1802 | 0.04 | 367.1175,311.0565 |
| 130 | 39.342 | Corylifol A | $C_{25}H_{26}O_4$ | $[M-H]^-$ | 389.1758 | 389.1761 | -0.68 | 265.0507,277.0511,320.1040 |
| 131 | 39.478 | Licoflavone B | $C_{25}H_{26}O_4$ | $[M+H]^+$ | 391.1904 | 391.1905 | -0.29 | 267.0658,239.0708,137.0238 |

**Table S8.** Differential metabolites

| Index | Compounds | Formula | score | CAS | VIP | P-value | FDR |
|---|---|---|---|---|---|---|---|
| MEDN0006 | L-Tyrosine | $C_9H_{11}NO_3$ | 0.966 | 60-18-4 | 1.5464846 | 0.0159939 | 0.2866659 |
| MW0006930 | Dopamine | $C_8H_{11}NO_2$ | 0.9556 | 62-31-7 | 1.4787881 | 0.026005 | 0.3434421 |

| | | | | | | | |
|---|---|---|---|---|---|---|---|
| MW0117082 | Indole-3-propionic acid | $C_{11}H_{11}NO_2$ | 0.9551 | 830-96-6 | 1.3616418 | 0.0236801 | 0.3328885 |
| MW0114590 | Hexaethylene glycol | $C_{12}H_{26}O_7$ | 0.9397 | 2615-15-8 | 1.4822977 | 0.0463745 | 0.3886256 |
| MW0012998 | LPC(18:0/0:0) | $C_{26}H_{54}NO_7P$ | 0.9289 | 19420-57-6 | 1.6091036 | 0.0030335 | 0.1612552 |
| MW0169297 | Indole-3-carbinol | $C_9H_9NO$ | 0.9243 | 700-06-1 | 1.3465453 | 0.0233745 | 0.3328885 |
| MW0150045 | Glu-Leu | $C_{11}H_{20}N_2O_5$ | 0.9077 | 5969-52-8 | 1.5625131 | 0.005105 | 0.2008321 |
| MW0126267 | protoporphyrin IX | $C_{34}H_{34}N_4O_4$ | 0.8873 | 553-12-8 | 1.5417876 | 0.0344223 | 0.37436 |
| MW0104770 | 2-Hydroxycinnamic acid | $C_9H_8O_3$ | 0.8715 | 614-60-8 | 1.5249376 | 0.0183065 | 0.3019276 |
| MW0012353 | 13-Docosenamide | $C_{22}H_{43}NO$ | 0.7801 | 112-84-5 | 1.5361241 | 0.0109538 | 0.2617735 |
| MEDP0588 | Ritalinic acid | $C_{13}H_{17}NO_2$ | 0.7676 | 19395-41-6 | 1.5237295 | 0.0128874 | 0.2632176 |
| MEDN1011 | Mannitol | $C_6H_{14}O_6$ | 0.7634 | 69-65-8 | 1.5376486 | 0.0118131 | 0.2623196 |
| MEDP0685 | Methylcysteine | $C_4H_9NO_2S$ | 0.7373 | 1187-84-4 | 1.5124786 | 0.0218897 | 0.3269138 |
| MW0003876 | 5-Bromo-4-hydroxy-[1,1'-biphenyl]-3-carboxylic acid | $C_{13}H_9BrO_3$ | 0.6823 | 4906-68-7 | 1.5754638 | 0.0171286 | 0.2964168 |
| MW0109260 | Phosphonoacetate | $C_2H_5O_5P$ | 0.6641 | 4408-78-0 | 1.6572371 | 0.0001749 | 0.0778407 |
| MW0169547 | Nicotinic acid | $C_6H_5NO_2$ | 0.5849 | 59-67-6 | 1.5395706 | 0.0105208 | 0.2582548 |
| MW0160313 | Sulfolithocholic acid | $C_{24}H_{40}O_6S$ | 0.5845 | 34669-57-3 | 1.3456309 | 0.0348671 | 0.3745809 |
| MW0106090 | Caffeic acid | $C_9H_8O_4$ | 0.517 | 501-16-6 | 1.5641682 | 0.0019406 | 0.1391314 |
| MW0056955 | PC(18:0/18:2) | $C_{44}H_{84}NO_8P$ | 0.9752 | 27098-24-4 | 1.6371944 | 0.0003899 | 0.0783406 |
| MW0062160 | Pregnanetriol | $C_{21}H_{36}O_3$ | 0.943 | 1098-45-9 \| 13933-75-0 | 1.4247297 | 0.0273143 | 0.348928 |
| MW0111235 | Octadecylamine | $C_{18}H_{39}N$ | 0.9261 | 124-30-1 | 1.6620791 | 0.0005869 | 0.0940775 |
| MW0049807 | 1-Palmitoyl-2-oleoyl-sn-glycerol | $C_{37}H_{70}O_5$ | 0.9177 | 29541-66-0 | 1.6217596 | 0.0021477 | 0.1508997 |
| MW0141122 | Dioleoylphosphatidylcholine | $C_{44}H_{84}NO_8P$ | 0.9027 | 52088-89-8 | 1.2272981 | 0.0429397 | 0.3886256 |
| MEDL01912 | Succinic acid semialdehyde | $C_4H_6O_3$ | 0.9429 | 692-29-5 | 1.4798872 | 0.0439299 | 0.3886256 |
| MW0161297 | Isocryptoxanthin | $C_{40}H_{56}O$ | 0.8843 | 472-62-8 | 1.6306346 | 0.0035569 | 0.1711678 |
| MW0169873 | Spirilloxanthin | $C_{42}H_{60}O_2$ | 0.8773 | 34255-08-8 | 1.6073424 | 0.0178988 | 0.2994213 |
| MW0055186 | Tetradecanoylcarnitine | $C_{21}H_{41}NO_4$ | 0.8702 | 25597-07-3 | 1.4221053 | 0.0428789 | 0.3886256 |
| MW0141837 | 1-Stearoyl-2-Arachidonoyl PC-d8 | $C_{46}H_{76}D_8NO_8P$ | 0.8602 | 161922-50-5 | 1.6230222 | 0.0180762 | 0.2995365 |
| FDATN00789 | Ondansetron hydrochloride dihydrate | $C_{18}H_{24}ClN_3O_3$ | 0.829 | 103639-04-9 \| 99614-01-4 | 1.6684384 | 5.863E-05 | 0.0778407 |
| MW0115598 | 1,2-Dichloroethane | $C_2H_4Cl_2$ | 0.8058 | 107-06-2 | 1.5216657 | 0.0268109 | 0.3450058 |
| MW0108132 | Melphalan | $C_{13}H_{18}Cl_2N_2O_2$ | 0.7966 | 148-82-3 | 1.3419762 | 0.0340179 | 0.37436 |
| MW0062208 | Prosapogenin A | $C_{39}H_{62}O_{12}$ | 0.7924 | 19057-67-1 | 1.4657849 | 0.0206526 | 0.3234737 |

| | | | | | | | |
|---|---|---|---|---|---|---|---|
| MW0168484 | Ethanolamine | $C_2H_7NO$ | 0.7849 | 141-43-5 | 1.5286284 | 0.0129631 | 0.2632328 |
| MW0054217 | KOdiA-PC | $C_{32}H_{58}NO_{11}P$ | 0.784 | 439904-33-3 | 1.6221075 | 0.0111124 | 0.2623196 |
| MW0114723 | Leucomycin A1 | $C_{40}H_{67}NO_{14}$ | 0.7835 | 16846-34-7 | 1.5723612 | 0.0219618 | 0.3269138 |
| MEDP1448 | MG(18:0/0:0/0:0) | $C_{21}H_{42}O_4$ | 0.7613 | 22610-61-3 | 1.605859 | 0.0009748 | 0.1180852 |
| MW0125030 | Methyl 1-methylpyrrole-2-carboxylate | $C_7H_9NO_2$ | 0.7559 | 37619-24-2 | 1.5760302 | 0.0475559 | 0.3894264 |
| MW0124660 | Leucodopachrome | $C_9H_9NO_4$ | 0.7399 | 18766-67-1 | 1.4292094 | 0.0409764 | 0.3886256 |
| MW0013819 | 3,4-Dihydrocoumarin | $C_9H_8O_2$ | 0.7381 | 119-84-6 | 1.5254648 | 0.0407617 | 0.3886256 |
| MW0119160 | 3',4'-(Methylenedioxy)acetophenone | $C_9H_8O_3$ | 0.7222 | 3162-29-6 | 1.6332845 | 0.0104182 | 0.2577398 |
| MW0012825 | 1-O-Hexadecyl-sn-glycero-3-phosphocholine | $C_{24}H_{52}NO_6P$ | 0.7221 | 52691-62-0 \| 97288-09-0 | 1.5798092 | 0.0054577 | 0.2008321 |
| MW0108118 | Malic acid | $C_4H_6O_5$ | 0.7177 | 97-67-6 | 1.6726237 | 0.0048905 | 0.1997729 |
| MW0012928 | PC(O-16:0/20:5(5Z,8Z,11Z,14Z,17Z)) | $C_{44}H_{80}NO_7P$ | 0.7164 | 132196-28-2 | 1.5647487 | 0.0125168 | 0.2632176 |
| FDATN00732 | 5-Chloro-8-hydroxyquinoline | $C_9H_6ClNO$ | 0.7109 | 130-16-5 | 1.6205659 | 0.0084583 | 0.2377131 |
| MW0154638 | N-Phosphotaurocyamine | $C_3H_{10}N_3O_6PS$ | 0.6991 | 4189-99-5 | 1.4557054 | 0.0440261 | 0.3886256 |
| MW0111297 | Tetrabutylammonium | $C_{16}H_{36}N+$ | 0.6985 | 10549-76-5 | 1.4418718 | 0.0347399 | 0.37436 |
| MW0125681 | Nb-Stearoyltryptamine | $C_{28}H_{46}N_2O$ | 0.6974 | 21469-14-7 | 1.4568536 | 0.0146318 | 0.2793565 |
| MW0050035 | 1-Stearoyl-2-arachidonoylglycerol | $C_{41}H_{72}O_5$ | 0.6971 | 65914-84-3 | 1.5921964 | 0.0453738 | 0.3886256 |
| MW0147515 | (2Z,4E)-4-hydroxy-6-oxohexa-2,4-dienoic acid | $C_6H_6O_4$ | 0.687 | 114828-85-2 | 1.5353756 | 0.0248781 | 0.3400647 |
| MW0169533 | N-benzoyl-d5-glycine | $C_9H_9NO_3$ | 0.6868 | 53518-98-2 | 1.6126318 | 0.0289297 | 0.3589703 |
| MW0169721 | Puromycin | $C_{22}H_{29}N_7O_5$ | 0.6746 | 53-79-2 \| 58-58-2 | 1.4487842 | 0.015834 | 0.2861593 |
| MW0157527 | Thio-THIP | $C_6H_8N_2OS$ | 0.6586 | 97164-95-9 | 1.3863399 | 0.04456 | 0.3886256 |
| MW0055335 | N-octanoylsphingosine 1-phosphate | $C_{26}H_{52}NO_6P$ | 0.6558 | 158983-53-0 | 1.6648688 | 0.0004014 | 0.0783406 |
| MEDP1658 | LPC(O-18:1) | $C_{26}H_{54}NO_6P$ | 0.6449 | 87907-66-2 | 1.6313863 | 0.0010982 | 0.1195787 |
| MW0144117 | 9,11-methane-epoxy Prostaglandin F1alpha | $C_{21}H_{36}O_4$ | 0.6382 | 72517-81-8 | 1.5302183 | 0.0177694 | 0.2994213 |
| MW0010294 | 1-Hexacosene | $C_{26}H_{52}$ | 0.6361 | 18835-33-1 | 1.4777522 | 0.0210896 | 0.3249457 |
| MEDL02249 | Chalcone | $C_{15}H_{12}O$ | 0.634 | 94-41-7 | 1.3398115 | 0.0204878 | 0.3234737 |
| MEDN0659 | Pyrophosphate | $H_4O_7P_2$ | 0.6322 | 206975 | 1.6313443 | 0.0025862 | 0.1519189 |
| MEDTP02857 | Thiram | $C_6H_{12}N_2S_4$ | 0.6295 | 137-26-8 | 1.6711166 | 0.0001282 | 0.0778407 |
| MW0110845 | 4-Hydroxycyclophosphamide | $C_7H_{15}Cl_2N_2O_3P$ | 0.609 | 40277-05-2 | 1.4446355 | 0.0318511 | 0.3680688 |
| MW0122860 | Anagrelide hydrochloride | $C_{10}H_8Cl_3N_3O$ | 0.5882 | 58579-51-4 | 1.4992606 | 0.0081599 | 0.2377131 |
| MW0010388 | Cyclotetracosane | $C_{24}H_{48}$ | 0.5822 | 297-03-0 | 1.6243494 | 0.0025984 | 0.1519189 |
| MW0007107 | Felbamate | $C_{11}H_{14}N_2O_4$ | 0.5705 | 25451-15-4 | 1.5837751 | 0.0358385 | 0.3769482 |

| | | | | | | | |
|---|---|---|---|---|---|---|---|
| FDATP00111 | Naftopidil dihydrochloride | $C_{24}H_{30}Cl_2N_2O_3$ | 0.5664 | 57149-08-3 | 1.5238542 | 0.0275217 | 0.3490383 |
| ZINC967836 | Nereistoxin | $C_5H_{11}NS_2$ | 0.5575 | 1631-58-9 | 1.4741084 | 0.0414184 | 0.3886256 |
| MW0009682 | Ritanserin | $C_{27}H_{25}F_2N_3OS$ | 0.5524 | 87051-43-2 | 1.4700827 | 0.0248396 | 0.3400647 |
| MW0110515 | Trifluoromethanesulfonic acid | $CHF_3O_3S$ | 0.5483 | 1493-13-6 | 1.5795442 | 0.0064518 | 0.2158577 |
| MEDP1139 | Linoleylethanolamide | $C_{20}H_{37}NO_2$ | 0.528 | 68171-52-8 | 1.5872779 | 0.0021937 | 0.1511069 |
| MW0169690 | Prazosin | $C_{19}H_{21}N_5O_4$ | 0.5113 | 19216-56-9 | 1.5163347 | 0.0436996 | 0.3886256 |
| MW0069011 | TG(15:0/15:0/15:0) | $C_{48}H_{92}O_6$ | 0.8749 | 184777-22-8 | 1.6130145 | 0.001337 | 0.1269402 |
| MW0159666 | Calcium carbonate | $CaCO_3$ | 0.7627 | 471-34-1 | 1.6058912 | 0.0088853 | 0.2438599 |
| MW0052800 | 12-PAHSA | $C_{34}H_{66}O_4$ | 0.6467 | 1997286-65-3 | 1.4453858 | 0.0075007 | 0.2311405 |
| FDATN00689 | Metaraminol bitartrate | $C_{13}H_{19}NO_8$ | 0.61 | 33402-03-8 \| 17171-57-2 | 1.3803263 | 0.0463785 | 0.3886256 |
| MW0106558 | (2R)-piperidin-1-ium-2-carboxylate | $C_6H_{11}NO_2$ | 0.9163 | - | 1.3994795 | 0.0240072 | 0.3346719 |
| MW0122532 | 7-Nitroindazole | $C_7H_5N_3O_2$ | 0.6696 | - | 1.6386429 | 0.0003416 | 0.0783406 |
| MW0057225 | 1,2-di-[(11Z)-eicosenoyl]-sn-glycero-3-phosphocholine | $C_{48}H_{92}NO_8P$ | 0.9569 | - | 1.391641 | 0.0131307 | 0.2651045 |
| MEDP1381 | Carnitine C20:2 | $C_{27}H_{49}NO_4$ | 0.9226 | - | 1.42612 | 0.0333015 | 0.3742381 |
| MEDP1171 | Butenoyl-PAF | $C_{28}H_{56}NO_7P$ | 0.9124 | - | 1.6041481 | 0.0253816 | 0.3421107 |
| MW0054541 | LPC(20:2/0:0) | $C_{28}H_{54}NO_7P$ | 0.8467 | - | 1.533128 | 0.0068649 | 0.2247597 |
| MW0056207 | [(2R)-2-(20-methylhenicosanoyloxy)-3-phosphonooxypropyl] docosanoate | $C_{47}H_{93}O_8P$ | 0.9469 | - | 1.5170475 | 0.0378827 | 0.3868119 |
| MW0056957 | PC(18:0/18:3(9Z,12Z,15Z)) | $C_{44}H_{82}NO_8P$ | 0.9385 | - | 1.6046693 | 0.0026379 | 0.1519189 |
| MW0056986 | 1,2-di-[(11Z)-octadecenoyl]-sn-glycero-3-phosphocholine | $C_{44}H_{84}NO_8P$ | 0.9335 | - | 1.6213356 | 0.0256659 | 0.3421107 |
| MW0056861 | 1-pentadecanoyl-2-(11Z,14Z-eicosadienoyl)-glycero-3-phosphocholine | $C_{43}H_{82}NO_8P$ | 0.9328 | - | 1.5272104 | 0.0165574 | 0.2928525 |
| MW0059999 | PE-NMe(22:6(4Z,7Z,10Z,13Z,16Z,19Z)/18:4(6Z,9Z,12Z,15Z)) | $C_{46}H_{72}NO_8P$ | 0.9128 | - | 1.6679883 | 0.0083848 | 0.2377131 |
| MW0058103 | 1-pentadecanoyl-2-(13Z,16Z-docosadienoyl)-glycero-3-phosphoethanolamine | $C_{42}H_{80}NO_8P$ | 0.9121 | - | 1.6112775 | 0.0342238 | 0.37436 |
| MW0059341 | PE-NMe(16:0/22:0) | $C_{44}H_{88}NO_8P$ | 0.8996 | - | 1.6601461 | 0.0237876 | 0.3329321 |
| MW0012818 | Arachidonoyl PAF C-16 | $C_{44}H_{82}NO_7P$ | 0.8899 | - | 1.6443092 | 0.0464177 | 0.3886256 |

| | | | | | | | |
|---|---|---|---|---|---|---|---|
| MW0150950 | His-Gly-Val-Asp-Lys | $C_{23}H_{38}N_8O_8$ | 0.8731 | - | 1.5140391 | 0.0413309 | 0.3886256 |
| MW0056121 | PA(20:5(5Z,8Z,11Z,14Z,17Z)/22:5(4Z,7Z,10Z,13Z,16Z)) | $C_{45}H_{69}O_8P$ | 0.8615 | - | 1.4254918 | 0.0053616 | 0.2008321 |
| MW0150187 | Glu-Tyr-Asp-Lys | $C_{24}H_{35}N_5O_{10}$ | 0.8392 | - | 1.4665596 | 0.0409949 | 0.3886256 |
| MW0062787 | PS(20:4(5Z,8Z,11Z,14Z)/14:1(9Z)) | $C_{40}H_{68}NO_{10}P$ | 0.8369 | - | 1.6139004 | 0.0386715 | 0.3885685 |
| MW0056193 | [(2R)-2-(12-methyltetradecanoyloxy)-3-phosphonooxypropyl] docosanoate | $C_{40}H_{79}O_8P$ | 0.8336 | - | 1.620797 | 0.0152057 | 0.2833094 |
| MW0062593 | PS(18:3(9Z,12Z,15Z)/18:3(9Z,12Z,15Z)) | $C_{42}H_{70}NO_{10}P$ | 0.825 | - | 1.5278758 | 0.0491657 | 0.3961451 |
| MW0056194 | [(2R)-2-(14-methylhexadecanoyloxy)-3-phosphonooxypropyl] docosanoate | $C_{42}H_{83}O_8P$ | 0.8078 | - | 1.2023965 | 0.0405767 | 0.3886256 |
| MW0147757 | Constanolactone E methyl acetonide | $C_{24}H_{40}O_5$ | 0.8073 | - | 1.278922 | 0.0458054 | 0.3886256 |
| MW0058274 | PE(18:1(9Z)/22:6(4Z,7Z,10Z,13Z,16Z,19Z)) | $C_{45}H_{76}NO_8P$ | 0.7995 | - | 1.6040445 | 0.0407664 | 0.3886256 |
| MEDP1319 | LPC(22:4/0:0) | $C_{30}H_{54}NO_7P$ | 0.7934 | - | 1.2144554 | 0.0297213 | 0.3611021 |
| MW0124797 | Metamitron | $C_{10}H_{10}N_4O$ | 0.7919 | - | 1.5784437 | 0.0361408 | 0.3789928 |
| MW0057157 | PC(18:4(6Z,9Z,12Z,15Z)/18:4(6Z,9Z,12Z,15Z)) | $C_{44}H_{72}NO_8P$ | 0.7915 | - | 1.5905849 | 0.0480113 | 0.3904255 |
| MW0060732 | PE-NMe2(20:5(5Z,8Z,11Z,14Z,17Z)/22:5(7Z,10Z,13Z,16Z,19Z)) | $C_{49}H_{78}NO_8P$ | 0.7875 | - | 1.5989593 | 0.0408877 | 0.3886256 |
| MW0060723 | PE-NMe2(20:5(5Z,8Z,11Z,14Z,17Z)/20:3(8Z,11Z,14Z)) | $C_{47}H_{78}NO_8P$ | 0.764 | - | 1.5991908 | 0.0447635 | 0.3886256 |
| MW0058481 | PE(20:2(11Z,14Z)/14:1(9Z)) | $C_{39}H_{72}NO_8P$ | 0.7521 | - | 1.5755013 | 0.0327136 | 0.3695268 |
| MW0145296 | Arg-Asp-Ile | $C_{16}H_{30}N_6O_6$ | 0.749 | - | 1.5140861 | 0.0120192 | 0.2623196 |
| MW0060576 | PE-NMe2(20:2(11Z,14Z)/20:2(11Z,14Z)) | $C_{47}H_{86}NO_8P$ | 0.7489 | - | 1.5311249 | 0.0379875 | 0.3868119 |
| MW0060159 | PE-NMe2(14:0/16:0) | $C_{37}H_{74}NO_8P$ | 0.7434 | - | 1.5887434 | 0.0492889 | 0.3962161 |
| MW0062292 | Dimyristoyl phosphatidylserine | $C_{34}H_{66}NO_{10}P$ | 0.7268 | - | 1.6392121 | 0.0251534 | 0.3421107 |
| MW0057410 | PC(20:5(5Z,8Z,11Z,14Z,17Z)/14:0) | $C_{42}H_{74}NO_8P$ | 0.7255 | - | 1.5849009 | 0.0445641 | 0.3886256 |
| MW0058463 | PE(20:1(11Z)/20:3(8Z,11Z,14Z)) | $C_{45}H_{82}NO_8P$ | 0.7253 | - | 1.4106206 | 0.0174976 | 0.2994213 |
| MW0011866 | 1,2-Docosahexanoyl-sn-glycero-3-phosphocholine | $C_{52}H_{80}NO_8P$ | 0.722 | - | 1.4585384 | 0.0280808 | 0.3532977 |
| MW0060671 | PE-NMe2(20:4(5Z,8Z,11Z,14Z)/22:2(13Z,16Z)) | $C_{49}H_{86}NO_8P$ | 0.7187 | - | 1.5950988 | 0.0074551 | 0.2311405 |
| MW0015865 | Arachidonoyl-1-thioglycerol | $C_{23}H_{38}O_3S$ | 0.7156 | - | 1.2986191 | 0.0421581 | 0.3886256 |
| MW0013614 | 1-Hexadecanoyl-2-(9Z-octadecenoyl)-sn-glycero-3-phosphoserine | $C_{40}H_{76}NO_{10}P$ | 0.7141 | - | 1.4104125 | 0.0414456 | 0.3886256 |
| MW0060545 | PE-NMe2(20:1(11Z)/20:0) | $C_{47}H_{92}NO_8P$ | 0.7118 | - | 1.5923955 | 0.0451938 | 0.3886256 |

| | | | | | | | |
|---|---|---|---|---|---|---|---|
| MW0057216 | 1-(11Z-eicosenoyl)-2-(9Z-hexadecenoyl)-glycero-3-phosphocholine | $C_{44}H_{84}NO_8P$ | 0.7052 | - | 1.4934488 | 0.026701 | 0.3448557 |
| MW0058391 | PE(18:4(6Z,9Z,12Z,15Z)/18:4(6Z,9Z,12Z,15Z)) | $C_{41}H_{66}NO_8P$ | 0.7048 | - | 1.5525766 | 0.0443124 | 0.3886256 |
| MW0059794 | PE-NMe(20:5(5Z,8Z,11Z,14Z,17Z)/18:3(6Z,9Z,12Z)) | $C_{44}H_{72}NO_8P$ | 0.704 | - | 1.5859206 | 0.0031753 | 0.1640419 |
| MW0055327 | Cer(d18:0/14:0) | $C_{32}H_{65}NO_3$ | 0.6994 | - | 1.6735947 | 0.0040645 | 0.1903804 |
| MW0143472 | 5-CHOLESTAN-3-OL-6-ONE | $C_{27}H_{46}O_2$ | 0.6966 | - | 1.6154736 | 0.0080572 | 0.2377131 |
| MW0151102 | His-Thr-Ser | $C_{13}H_{21}N_5O_6$ | 0.6933 | - | 1.5847359 | 0.0323705 | 0.3695268 |
| MW0155416 | Phe-Val-Trp | $C_{25}H_{30}N_4O_4$ | 0.6894 | - | 1.4102131 | 0.0312435 | 0.3639866 |
| MW0056962 | PC(18:0/20:3(5Z,8Z,11Z)) | $C_{46}H_{86}NO_8P$ | 0.6852 | - | 1.6474192 | 0.0015932 | 0.1314936 |
| MW0057337 | PC(20:3(8Z,11Z,14Z)/22:6(4Z,7Z,10Z,13Z,16Z,19Z)) | $C_{50}H_{82}NO_8P$ | 0.6744 | - | 1.6307512 | 0.0142271 | 0.2760263 |
| MW0011923 | 1,2-Dipentadecanoyl-sn-glycero-3-phosphoethanolamine | $C_{35}H_{70}NO_8P$ | 0.6658 | - | 1.5465653 | 0.0167851 | 0.2933639 |
| MW0058316 | PE(18:3(6Z,9Z,12Z)/15:0) | $C_{38}H_{70}NO_8P$ | 0.6626 | - | 1.48453 | 0.0394901 | 0.3886256 |
| MW0009478 | 4-(5H-Dibenzo[a,d]cyclohepten-5-ylidene)-1-[4-(2H-tetrazol-5-yl)butyl]-piperidine | $C_{25}H_{27}N_5$ | 0.6544 | - | 1.5399741 | 0.0216448 | 0.3269138 |
| MEDTP02779 | [(2R)-3-hexadecoxy-2-hydroperoxypropyl] 2-(trimethylazaniumyl)ethyl phosphate | $C_{24}H_{52}NO_7P$ | 0.6506 | - | 1.4828446 | 0.0177836 | 0.2994213 |
| MW0056936 | PC(16:1(9Z)/22:2(13Z,16Z)) | $C_{46}H_{86}NO_8P$ | 0.6369 | - | 1.1706046 | 0.0218742 | 0.3269138 |
| MW0059747 | PE-NMe(20:4(5Z,8Z,11Z,14Z)/22:0) | $C_{48}H_{88}NO_8P$ | 0.6316 | - | 1.4276861 | 0.0221796 | 0.3285893 |
| MEDN1649 | Barbital | $C_8H_{12}N_2O_3$ | 0.6001 | - | 1.6453587 | 0.0018893 | 0.1391314 |
| MW0152252 | Leu-Asn-Trp | $C_{21}H_{29}N_5O_5$ | 0.5934 | - | 1.5437659 | 0.0072578 | 0.2311405 |
| MW0138483 | Isokaempferide | $C_{16}H_{12}O_6$ | 0.5773 | - | 1.4678314 | 0.0217713 | 0.3269138 |
| MW0143259 | 4'-Apo-beta-carotenal | $C_{35}H_{46}O$ | 0.5717 | - | 1.6551084 | 0.0054362 | 0.2008321 |
| MEDP1429 | Carnitine C5:DC | $C_{12}H_{21}NO_6$ | 0.5491 | - | 1.549359 | 0.045169 | 0.3886256 |
| MW0134891 | 6-(2H-1,3-benzodioxole-5-carbonyloxy)-3,4,5-trihydroxyoxane-2-carboxylic acid | $C_{14}H_{14}O_{10}$ | 0.5464 | - | 1.5344206 | 0.0423743 | 0.3886256 |
| MW0069062 | TG(15:0/16:0/15:0) | $C_{49}H_{94}O_6$ | 0.973 | - | 1.6106886 | 0.0386182 | 0.3885685 |
| MW0050542 | DG(20:2(11Z,14Z)/22:5(4Z,7Z,10Z,13Z,16Z)/0:0) | $C_{45}H_{74}O_5$ | 0.9501 | - | 1.5685739 | 0.0064334 | 0.2158577 |
| MW0069063 | TG(15:0/16:0/16:1(9Z)) | $C_{50}H_{94}O_6$ | 0.9411 | - | 1.624551 | 0.0024316 | 0.1519189 |
| MW0068203 | TG(14:1(9Z)/15:0/20:3(5Z,8Z,11Z)) | $C_{52}H_{92}O_6$ | 0.932 | - | 1.6709401 | 0.0093982 | 0.2479621 |
| MW0069109 | TG(15:0/16:1(9Z)/15:0) | $C_{49}H_{92}O_6$ | 0.897 | - | 1.4577828 | 0.0008497 | 0.112259 |
| MW0168172 | 2-[[(4R)-4-[(3R,5S,7R,9S,10S,12S,13R,14S,17R)-3,7,12-trihydroxy-10,13-dimethyl-2,3,4,5,6,7,8,9,11,12,14,15,16,17-tetradecahydro-1H- | $C_{26}H_{45}NO_7S$ | 0.8822 | - | 1.6182723 | 0.023589 | 0.3328885 |

| | | | | | | | |
|---|---|---|---|---|---|---|---|
| | cyclopenta[a]phenanthren-17-yl]pentanoyl]amino]ethanesulfonic acid | | | | | | |
| MW0063991 | TG(10:0/16:0/13:0) | $C_{42}H_{80}O_6$ | 0.8715 | - | 1.4533425 | 0.0304679 | 0.3611021 |
| MW0066610 | TG(14:0/15:0/20:4(5Z,8Z,11Z,14Z)) | $C_{52}H_{92}O_6$ | 0.8647 | - | 1.3509368 | 0.0008628 | 0.112259 |
| MW0167470 | [(2S)-2,3-bis[[(Z)-tetradec-9-enoyl]oxy]propyl] (Z)-hexadec-9-enoate | $C_{47}H_{84}O_6$ | 0.8334 | - | 1.5955548 | 0.0346637 | 0.37436 |
| MW0075411 | TG(18:1(11Z)/14:0/18:2(9Z,12Z)) | $C_{53}H_{96}O_6$ | 0.8311 | - | 1.4975842 | 0.00807 | 0.2377131 |
| MW0063980 | TG(10:0/15:0/i-14:0) | $C_{42}H_{80}O_6$ | 0.7971 | - | 1.6298503 | 0.0002313 | 0.0783406 |
| MW0142392 | 2-[(1Z,3E)-5-carboxy-1-hydroxy-5-oxopenta-1,3-dienyl]benzoic acid | $C_{13}H_{10}O_6$ | 0.7923 | - | 1.6068736 | 0.0229466 | 0.3328885 |
| MW0167447 | [(2S)-2-pentadecanoyloxy-3-[(Z)-tetradec-9-enoyl]oxypropyl] (6Z,9Z,12Z)-octadeca-6,9,12-trienoate | $C_{50}H_{88}O_6$ | 0.7861 | - | 1.4316399 | 0.0156046 | 0.2855156 |
| MW0069061 | TG(15:0/16:0/14:1(9Z)) | $C_{48}H_{90}O_6$ | 0.7812 | - | 1.3584141 | 0.0313233 | 0.3639866 |
| MW0141803 | 1-O-(cis-9-Octadecenyl)-2-O-acetyl-sn-glycero-3-phosphocholine | $C_{28}H_{57}NO_7P+$ | 0.7749 | - | 1.6507397 | 0.0164951 | 0.2928525 |
| MW0069078 | TG(15:0/16:0/20:4(8Z,11Z,14Z,17Z)) | $C_{54}H_{96}O_6$ | 0.7646 | - | 1.6415934 | 0.0060653 | 0.2109627 |
| MW0155401 | Phe-Val-Asp-Lys | $C_{24}H_{37}N_5O_7$ | 0.7283 | - | 1.5884211 | 0.0117982 | 0.2623196 |
| MW0000251 | Ergotaman-3',6',18-trione, 12'-hydroxy-2'-methyl-5'-(phenylmethyl)-, (5'alpha)- | $C_{33}H_{35}N_5O_5$ | 0.7256 | - | 1.5667151 | 0.0011757 | 0.1195787 |
| MW0146816 | Boc-Phe(NMe)-Pro-Phe(NMe)-Gly-OMe | $C_{33}H_{44}N_4O_7$ | 0.7052 | - | 1.6362337 | 0.000747 | 0.109343 |
| MW0051604 | [(2S)-3-hydroxy-2-(18-methylnonadecanoyloxy)propyl] 22-methyltetracosanoate | $C_{48}H_{94}O_5$ | 0.698 | - | 1.5542866 | 0.0293058 | 0.3611021 |
| MW0146178 | Asp-Leu-Ser-Glu | $C_{18}H_{30}N_4O_{10}$ | 0.6857 | - | 1.5724962 | 0.0150656 | 0.2833094 |
| MW0145971 | Asparagylaspartic acid | $C_8H_{13}N_3O_6$ | 0.6638 | - | 1.4109297 | 0.0232958 | 0.3328885 |
| MW0111049 | 1-O-Hexadecyl-2-deoxy-2-thio-R-(heptanoyl)-sn-glyceryl-3-phosphocholine | $C_{31}H_{64}NO_6PS$ | 0.6458 | - | 1.4845702 | 0.0123849 | 0.2632176 |
| MW0142374 | 2-Hydroxy-3-methyl-2-cyclopenten-1-one | $C_6H_8O_2$ | 0.6017 | - | 1.6240309 | 0.0024725 | 0.1519189 |
| MW0067194 | TG(14:0/20:4(5Z,8Z,11Z,14Z)/18:4(6Z,9Z,12Z,15Z)) | $C_{55}H_{90}O_6$ | 0.5916 | - | 1.5472133 | 0.0446526 | 0.3886256 |
| MEDL00638 | Taurocholic acid | $C_{26}H_{45}NO_7S$ | 0.9635 | 81-24-3 | 1.5178655 | 0.0056025 | 0.2008321 |
| MW0107948 | Pyroglutamic acid | $C_5H_7NO_3$ | 0.9442 | 98-79-3 | 1.5518595 | 0.0172285 | 0.2966853 |
| MW0014242 | 3-Oxocholic acid | $C_{24}H_{38}O_5$ | 0.9423 | 2304-89-4 | 1.4795738 | 0.0443843 | 0.3886256 |
| MEDN1751 | 2-Hexyldecanoic acid | $C_{16}H_{32}O_2$ | 0.9395 | 25354-97-6 | 1.6017 | 0.0015134 | 0.1314936 |

| | | | | | | | |
|---|---|---|---|---|---|---|---|
| MW0119641 | 3-Indoleacrylic acid | $C_{11}H_9NO_2$ | 0.9275 | 29953-71-7 | 1.5677257 | 0.0011888 | 0.1195787 |
| MW0128009 | L-3-Phenyllactic acid | $C_9H_{10}O_3$ | 0.8875 | 20312-36-1 | 1.6046472 | 0.0490226 | 0.3958999 |
| MW0003649 | 3,4-Dimethylbenzoic acid | $C_9H_{10}O_2$ | 0.8754 | 619-04-5 | 1.4070612 | 0.0391098 | 0.3886256 |
| MEDN0202 | α-Ketoglutaric Acid (α-KG) | $C_5H_6O_5$ | 0.8094 | 328-50-7 | 1.5608063 | 0.0107332 | 0.2582577 |
| MW0002691 | 2-Amino-5-nitrobenzoic acid | $C_7H_6N_2O_4$ | 0.8628 | 616-79-5 | 1.6140461 | 0.0256843 | 0.3421107 |
| MW0103485 | beta-Nicotinamide ribose monophosphate | $C_{11}H_{16}N_2O_8P^+$ | 0.8147 | 75414-16-3 | 1.450575 | 0.032456 | 0.3695268 |
| MEDP0827 | Isophorone | $C_9H_{14}O$ | 0.8031 | 78-59-1 | 1.5039322 | 0.0323187 | 0.3695268 |
| MW0108639 | N-Acetyl-L-glutamic acid | $C_7H_{11}NO_5$ | 0.7918 | 1188-37-0 | 1.4982011 | 0.025955 | 0.3434421 |
| MEDN1534 | Tagatose | $C_6H_{12}O_6$ | 0.7781 | 41847-61-4 | 1.4965901 | 0.0190688 | 0.3114651 |
| MEDN0691 | Maltitol | $C_{12}H_{24}O_{11}$ | 0.7661 | 585-88-6 | 1.4153877 | 0.0302952 | 0.3611021 |
| MW0005838 | 6-Phosphogluconic acid | $C_6H_{13}O_{10}P$ | 0.7625 | 921-62-0 | 1.5542332 | 0.0047605 | 0.1997729 |
| MW0014621 | 5,15-DiHETE | $C_{20}H_{32}O_4$ | 0.7593 | 82200-87-1 | 1.6236065 | 0.0400228 | 0.3886256 |
| MW0015222 | 7-Methylxanthine | $C_6H_6N_4O_2$ | 0.687 | 552-62-5 | 1.4349381 | 0.0461239 | 0.3886256 |
| MW0015437 | 9-Decenoic acid | $C_{10}H_{18}O_2$ | 0.6549 | 14436-32-9 | 1.3416761 | 0.0344774 | 0.37436 |
| MEDP0864 | (R)-Methyl 3-hydroxy-2-methylpropanoate | $C_4H_8O_3$ | 0.6028 | 2068-83-9 | 1.4284138 | 0.0194739 | 0.3152614 |
| MW0103590 | Guanosine-5'-monophosphate | $C_{10}H_{14}N_5O_8P$ | 0.5998 | 85-32-5 \| 128952-18-1 \| 29593-02-0 | 1.5684585 | 0.0472107 | 0.3894264 |
| MW0049033 | Cortisone | $C_{21}H_{28}O_5$ | 0.5893 | 19515 | 1.5106001 | 0.00967 | 0.2479621 |
| MW0107902 | L-Leucyl-L-alanine | $C_9H_{18}N_2O_3$ | 0.5772 | 7298-84-2 | 1.4345477 | 0.0403166 | 0.3886256 |
| MW0114223 | D-glucosamine 6-phosphate | $C_6H_{14}NO_8P$ | 0.5454 | 6815-93-6 | 1.4188057 | 0.0301352 | 0.3611021 |
| MW0106158 | cis-3-Hexenyl acetate | $C_8H_{14}O_2$ | 0.9506 | 3681-71-8 | 1.4098375 | 0.0447227 | 0.3886256 |
| MW0103037 | Unoprostone | $C_{22}H_{38}O_5$ | 0.9395 | 120373-36-6 | 1.3362485 | 0.0127218 | 0.2632176 |
| MW0055293 | Nervonic acid | $C_{24}H_{46}O_2$ | 0.932 | 506-37-6 | 1.5539372 | 0.0013777 | 0.1273671 |
| MW0012972 | 1-Palmitoyl-2-oleoyl-sn-glycero-3-(phospho-rac-(1-glycerol)) | $C_{40}H_{77}O_{10}P$ | 0.9309 | 185435-28-3 | 1.5095767 | 0.0011342 | 0.1195787 |
| MW0103271 | Cis-8,11,14,17-Eicosatetraenoic acid | $C_{20}H_{32}O_2$ | 0.9232 | 2091-26-1 | 1.608214 | 0.0015965 | 0.1314936 |
| MW0111680 | 1,1-Dimethoxynonane | $C_{11}H_{24}O_2$ | 0.9167 | 18824-63-0 | 1.4871803 | 0.042734 | 0.3886256 |
| MW0103061 | FAA(18:1) | $C_{18}H_{34}O_2$ | 0.9093 | 506-17-2 | 1.4465135 | 0.0357106 | 0.3767306 |
| MW0103260 | Isohyodeoxycholic acid | $C_{24}H_{40}O_4$ | 0.9083 | 570-84-3 | 1.656688 | 0.0083235 | 0.2377131 |
| MW0109742 | Sodium dodecyl sulfate | $C_{12}H_{25}NaO_4S$ | 0.8891 | 151-21-3 \| 12738-53-3 \| | 1.5339161 | 0.009153 | 0.2458988 |

| | | | | | | | |
|---|---|---|---|---|---|---|---|
| MEDN1156 | FFA(20:4) | $C_{20}H_{32}O_2$ | 0.8717 | 12765-21-8 \| 1334-67-4 82073-91-4 | 1.347986 | 0.040442 | 0.3886256 |
| MW0139450 | Puerarin | $C_{21}H_{20}O_9$ | 0.8092 | 3681-99-0 | 1.2686375 | 0.0176448 | 0.2994213 |
| MW0012975 | 1-Hexadecanoyl-2-octadecanoyl-sn-glycero-3-phosphocholine | $C_{42}H_{84}NO_8P$ | 0.8921 | 59403-51-9 | 1.6604093 | 0.0003085 | 0.0783406 |
| MW0140636 | (2s)-2-(2,4-Dichlorophenoxy)propanoic acid | $C_9H_8Cl_2O_3$ | 0.8779 | 15165-69-2 | 1.6681887 | 9.025E-05 | 0.0778407 |
| MW0116828 | 5-Bromopyrimidine | $C_4H_3BrN_2$ | 0.8748 | 4595-59-9 | 1.5270085 | 0.0212884 | 0.3265769 |
| MW0163576 | 2-dehydro-3-deoxy-D-gluconate | $C_6H_9O_6$- | 0.8644 | 17510-99-5 | 1.588569 | 0.0112444 | 0.2623196 |
| MW0123371 | Citral propylene glycol acetal | $C_{13}H_{22}O_2$ | 0.8605 | 10444-50-5 | 1.3649022 | 0.0207269 | 0.3234737 |
| MW0012308 | Isoacitretin | $C_{21}H_{26}O_3$ | 0.8543 | 69427-46-9 | 1.5918962 | 0.0152438 | 0.2833094 |
| MW0137472 | Baicalin | $C_{21}H_{18}O_{11}$ | 0.8517 | 21967-41-9 \| 31564-28-0 | 1.6126914 | 0.048154 | 0.3906815 |
| MW0012971 | 1-Palmitoyl-2-oleoylglycero-3-phosphoglycerol | $C_{40}H_{77}O_{10}P$ | 0.845 | 81490-05-3 | 1.6635326 | 0.0066796 | 0.2213727 |
| MW0109516 | R-1 Methanandamide Phosphate | $C_{23}H_{40}NO_5P$ | 0.8422 | 649569-33-5 | 1.5651347 | 0.0106842 | 0.2582577 |
| MW0052462 | Docusate | $C_{20}H_{38}O_7S$ | 0.8287 | 10041-19-7 | 1.4332079 | 0.029638 | 0.3611021 |
| MW0129941 | 1-O-trans-Cinnamoyl-beta-D-glucopyranose | $C_{15}H_{18}O_7$ | 0.8251 | 40004-96-4 | 1.552794 | 0.0405457 | 0.3886256 |
| MW0120571 | (R-(R*,R*))-3-(3-Cyclohexyl-3-hydroxypropyl)-2,5-dioxo-imidazolidine-4-heptanoic acid | $C_{19}H_{32}N_2O_5$ | 0.8227 | 78420-15-2 \| 65705-83-1 | 1.4395661 | 0.0166434 | 0.2928525 |
| MW0011902 | 1,2-Dioleoyl-sn-Glycero-3-Phosphocholine | $C_{44}H_{84}NO_8P$ | 0.8225 | 4235-95-4 | 1.4029913 | 0.0027457 | 0.1555734 |
| MW0112757 | 3-Benzoyl-6-phenyl-pyran-2,4-dione | $C_{18}H_{12}O_4$ | 0.8152 | 602-98-2 | 1.5845013 | 0.0467944 | 0.3886256 |
| ZINC19682848 | 3-(4-Hydroxy-3-methoxyphenyl)propanal | $C_{10}H_{12}O_3$ | 0.8137 | 80638-48-8 | 1.1656427 | 0.0448242 | 0.3886256 |
| MW0107617 | Kojic acid | $C_6H_6O_4$ | 0.8128 | 501-30-4 | 1.455292 | 0.0198804 | 0.3189033 |
| MW0142811 | 3-Amino-6H-dibenzo[b,d]pyran-6-one | $C_{13}H_9NO_2$ | 0.8012 | 6967-04-0 | 1.3286843 | 0.0355838 | 0.3765515 |
| MW0141493 | 16-Oxohexadecanoic acid | $C_{16}H_{30}O_3$ | 0.7983 | 69275-06-5 | 1.5816809 | 0.0387131 | 0.3885685 |
| MW0015406 | (-)-9,10-Dihydrojasmonic acid | $C_{12}H_{20}O_3$ | 0.7925 | 76968-33-7 \| 98674-52-3 | 1.5426373 | 0.03254 | 0.3695268 |
| MW0012541 | 16alpha-Hydroxydehydroepiandrosterone | $C_{19}H_{28}O_3$ | 0.7844 | 1232-73-1 | 1.637124 | 0.0035303 | 0.1711678 |
| MW0107187 | Glyoxylic acid | $C_2H_2O_3$ | 0.7835 | 298-12-4 | 1.3711882 | 0.0305363 | 0.3611021 |
| MW0152764 | Lovastatin acid | $C_{24}H_{38}O_6$ | 0.7728 | 75225-51-3 | 1.3784426 | 0.0223264 | 0.3285893 |
| MW0006786 | Dichlorprop-P | $C_9H_8Cl_2O_3$ | 0.7693 | 15165-67-0 | 1.6461352 | 0.0063698 | 0.2158577 |
| MW0010092 | Zinniol | $C_{15}H_{22}O_4$ | 0.7692 | 17811-28-8 | 1.4504517 | 0.0279268 | 0.352902 |
| MW0056887 | 1-Palmitoyl-2-oleoyl-sn-glycero-3-phosphocholine | $C_{42}H_{82}NO_8P$ | 0.7685 | 26853-31-6 | 1.6548268 | 0.0305915 | 0.3611021 |

| | | | | | | | |
|---|---|---|---|---|---|---|---|
| MW0009321 | 4-Acetamidobenzaldehyde | $C_9H_9NO_2$ | 0.7656 | 122-85-0 \| 34-24-2 | 1.2597458 | 0.0266667 | 0.3448557 |
| MW0125809 | Nilutamide | $C_{12}H_{10}F_3N_3O_4$ | 0.7637 | 63612-50-0 | 1.3396118 | 0.0383494 | 0.3885685 |
| MW0017031 | Chamissonolide | $C_{17}H_{24}O_6$ | 0.761 | 78853-98-2 | 1.5647252 | 0.0083912 | 0.2377131 |
| MW0124336 | Hydroxyatrazine | $C_8H_{15}N_5O$ | 0.7608 | 2163-68-0 | 1.5078761 | 0.0373108 | 0.3852945 |
| MEDP1067 | Palmitoyl-N-isopropylamide | $C_{19}H_{39}NO$ | 0.7599 | 189939-61-5 | 1.3998382 | 0.0383956 | 0.3885685 |
| MW0144065 | 7-Chloro-2-dibenzofuranol | $C_{12}H_7ClO_2$ | 0.756 | 73518-07-7 | 1.3773761 | 0.0373998 | 0.3852945 |
| MW0007573 | Mefenamic acid | $C_{15}H_{15}NO_2$ | 0.7504 | 61-68-7 | 1.2684593 | 0.0257094 | 0.3421107 |
| MW0141917 | 3-(2-Hydroxyphenyl)catechol | $C_{12}H_{10}O_3$ | 0.7469 | 91368-55-7 | 1.5180988 | 0.0405089 | 0.3886256 |
| MW0142077 | 2,5-Dichlorobiphenyl | $C_{12}H_8Cl_2$ | 0.7458 | 34883-39-1 | 1.4749906 | 0.0302267 | 0.3611021 |
| MEDL02162 | FFA(22:5) | $C_{22}H_{34}O_2$ | 0.7432 | 24880-45-3 | 1.6045418 | 0.000621 | 0.0948532 |
| MW0009052 | Nepafenac | $C_{15}H_{14}N_2O_2$ | 0.7403 | 78281-72-8 | 1.4601472 | 0.0203775 | 0.3234737 |
| MW0123155 | Brazilin | $C_{16}H_{14}O_5$ | 0.7402 | 474-07-7 | 1.4434204 | 0.0264278 | 0.3447271 |
| MW0007215 | Fosamprenavir | $C_{25}H_{36}N_3O_9PS$ | 0.7322 | 226700-79-4 | 1.6082038 | 0.0333437 | 0.3742381 |
| MW0006527 | Chloramphenicol | $C_{11}H_{12}Cl_2N_2O_5$ | 0.7266 | 56-75-7 | 1.3822756 | 0.040476 | 0.3886256 |
| MW0103204 | Zymosterol | $C_{27}H_{44}O$ | 0.7216 | 128-33-6 | 1.6108208 | 0.0033713 | 0.169189 |
| MW0126740 | Tetrahydrothiophene-2-carboxylic acid | $C_5H_8O_2S$ | 0.7171 | 19418-11-2 | 1.434131 | 0.0214761 | 0.3269138 |
| MW0143450 | Aspartyl phosphate | $C_4H_8NO_7P$ | 0.7166 | 22138-53-0 | 1.3870839 | 0.0422076 | 0.3886256 |
| ZINC1672876 | Heptyl acetate | $C_9H_{18}O_2$ | 0.7135 | 112-06-1 | 1.4528032 | 0.0367364 | 0.3830579 |
| MW0142590 | 3 Arachidonic Acid-d8 | $C_{20}H_{24}D_8O_2$ | 0.7106 | 69254-37-1 | 1.6232575 | 0.0009218 | 0.1156564 |
| MW0006401 | Bissalicyl fumarate | $C_{18}H_{12}O_8$ | 0.7105 | 74134-01-3 | 1.6438469 | 0.009652 | 0.2479621 |
| MW0054376 | Linoleic Acid-d4 | $C_{18}H_{32}O_2$ | 0.7079 | 79050-23-0 | 1.5040022 | 0.0422869 | 0.3886256 |
| MW0004001 | 3-Furoic acid | $C_5H_4O_3$ | 0.7069 | 488-93-7 | 1.5050184 | 0.0096428 | 0.2479621 |
| MW0114215 | D-Glycero-D-gulo-heptose | $C_7H_{14}O_7$ | 0.7043 | 3146-50-7 \| 62475-58-5 | 1.6535451 | 0.0281592 | 0.3532977 |
| MW0111843 | 1-Naphthyl glucuronide | $C_{16}H_{16}O_7$ | 0.7039 | 17238-47-0 \| 27710-10-7 | 1.4294093 | 0.0254545 | 0.3421107 |
| MW0140207 | (1E)-4-oxobut-1-ene-1,2,4-tricarboxylic acid | $C_7H_6O_7$ | 0.7008 | 85179-60-8 | 1.6308564 | 0.0054983 | 0.2008321 |
| MW0146516 | Bacteriochlorophyll | $C_{55}H_{74}MgN_4O_6$ | 0.6984 | 17499-98-8 | 1.5185554 | 0.0473735 | 0.3894264 |
| MW0118564 | 2-heptylquinolin-4(1H)-one | $C_{16}H_{21}NO$ | 0.6969 | 40522-46-1 \| 2503-80-2 | 1.4963865 | 0.0343061 | 0.37436 |

| | | | | | | | |
|---|---|---|---|---|---|---|---|
| MW0053497 | Geranylgeranyl diphosphate | $C_{20}H_{36}O_7P_2$ | 0.6878 | 6699-20-3 | 1.3866674 | 0.0494346 | 0.3962161 |
| MW0138079 | Epigallocatechin | $C_{15}H_{14}O_7$ | 0.6841 | 970-74-1 | 1.6481492 | 0.0025872 | 0.1519189 |
| MW0123351 | cis-2-Chloro-4-carboxymethylenebut-2-en-1,4-olide | $C_6H_3ClO_4$ | 0.6803 | 115793-61-8 \| 18529-78-7 | 1.5953906 | 0.0136602 | 0.2668184 |
| MW0193980 | Sorbitol-6-phosphate | $C_6H_{15}O_9P$ | 0.675 | 20479-58-7 | 1.5571338 | 0.0011914 | 0.1195787 |
| MW0158047 | Triclofos | $C_2H_4Cl_3O_4P$ | 0.674 | 306-52-5 | 1.5454762 | 0.0451541 | 0.3886256 |
| MW0053459 | Ganodermic acid TQ | $C_{32}H_{46}O_5$ | 0.6715 | 112430-66-7 | 1.3948526 | 0.0473379 | 0.3894264 |
| MW0148548 | Dimethyl telluride | $C_2H_6Te$ | 0.6624 | 593-80-6 | 1.5934934 | 0.0002774 | 0.0783406 |
| MW0155496 | Pheophytin a | $C_{55}H_{74}N_4O_5$ | 0.6613 | 603-17-8 | 1.3781771 | 0.0422622 | 0.3886256 |
| MW0109259 | (R)-3-Amino-4-(3-hexylphenylamino)-4-oxobutylphosphonic acid | $C_{16}H_{27}N_2O_4P$ | 0.6524 | 909725-61-7 | 1.4281913 | 0.028695 | 0.3574664 |
| MW0153846 | N-Nitroso-N-methylurea | $C_2H_5N_3O_2$ | 0.6515 | 684-93-5 \| 28606-00-0 | 1.541311 | 0.010586 | 0.2582548 |
| MW0063833 | Tetrahymanol | $C_{30}H_{52}O$ | 0.6493 | 2130-17-8 | 1.372824 | 0.0449953 | 0.3886256 |
| MW0156094 | Propan-2-yl 7-{(1R,2R,3S,5S)-2-[(3R)-3-(2,3-dihydro-1H-inden-2-yl)-3-hydroxyprop-1-en-1-yl]-3-fluoro-5-hydroxycyclopentyl}hept-5-enoate | $C_{27}H_{37}FO_4$ | 0.6491 | 208114-93-6 | 1.6100188 | 0.043263 | 0.3886256 |
| MW0157517 | Thiocysteine | $C_3H_7NO_2S_2$ | 0.6444 | 5652-32-4 | 1.2480027 | 0.046774 | 0.3886256 |
| MW0106668 | Ethyl sulfate | $C_2H_6O_4S$ | 0.6397 | 540-82-9 | 1.5939277 | 0.0197921 | 0.3189033 |
| MW0063587 | Solanesyl-PP | $C_{45}H_{76}O_7P_2$ | 0.6355 | 146340-00-3 | 1.5539656 | 0.001291 | 0.1259755 |
| FDATN01474 | DL-Serine | $C_3H_7NO_3$ | 0.6331 | 302-84-1 \| 6898-95-9 \| 25821-52-7 | 1.6383313 | 0.0077643 | 0.2351385 |
| MW0151849 | Isopenicillin N | $C_{14}H_{21}N_3O_6S$ | 0.6313 | 58678-43-6 | 1.3912048 | 0.0230285 | 0.3328885 |
| MW0141189 | Naphthalene-1,6-disulfonic acid | $C_{10}H_8O_6S_2$ | 0.6153 | 525-37-1 | 1.2653477 | 0.0306169 | 0.3611021 |
| MW0155563 | Picrasin C | $C_{23}H_{34}O_7$ | 0.6092 | 33804-89-6 | 1.574874 | 0.0246611 | 0.3397426 |
| MW0007840 | Monoethyl phthalate | $C_{10}H_{10}O_4$ | 0.6034 | 2306-33-4 | 1.6239041 | 0.0016095 | 0.1314936 |
| MW0106387 | Dexrazoxane | $C_{11}H_{16}N_4O_4$ | 0.603 | 24584-09-6 | 1.3556636 | 0.0457179 | 0.3886256 |
| MW0129533 | 1-(2-Hydroxyphenyl)-3-phenylpropane-1,3-dione | $C_{15}H_{12}O_3$ | 0.6021 | 1469-94-9 | 1.3718881 | 0.0431657 | 0.3886256 |
| MW0155706 | 3alpha-Hydroxy-5beta-pregnane-20-one sulfate | $C_{21}H_{34}O_5S$ | 0.5783 | 1477-67-4 | 1.5048406 | 0.0236898 | 0.3328885 |
| MW0169052 | Dexamethasone | $C_{22}H_{29}FO_5$ | 0.5728 | 23495-06-9 | 1.436847 | 0.0091696 | 0.2458988 |
| ZINC119983 | Cianidanol | $C_{15}H_{14}O_6$ | 0.5657 | 154-23-4 | 1.5685964 | 0.0134628 | 0.2668184 |
| MW0105485 | Ala-Tyr | $C_{12}H_{16}N_2O_4$ | 0.5656 | 3061-88-9 | 1.651414 | 0.0122756 | 0.2632176 |
| MW0125994 | N-Acetyl-a-neuraminic acid | $C_{11}H_{19}NO_9$ | 0.5052 | 21646-00-4 | 1.4579929 | 0.0054989 | 0.2008321 |

| | | | | | | | |
|---|---|---|---|---|---|---|---|
| MW0063789 | Tetracosanoic acid | $C_{24}H_{48}O_2$ | 0.9439 | 557-59-5 | 1.4000881 | 0.0327129 | 0.3695268 |
| MW0103959 | (E)-10-Hydroxy-8-decenoic acid | $C_{10}H_{18}O_3$ | 0.8275 | 106541-97-3 | 1.4523864 | 0.0290201 | 0.3589703 |
| MW0117883 | 2,4,6-Trimethyl-1,3,5-trithiane | $C_6H_{12}S_3$ | 0.6184 | 2765-04-0 | 1.6574901 | 0.0044517 | 0.199493 |
| FDATN01395 | Bactocill | $C_{19}H_{20}N_3NaO_6S$ | 0.5835 | 7240-38-2 \| 1173-88-2 | 1.4676379 | 0.0449279 | 0.3886256 |
| MW0142781 | 3alpha,12alpha-Dihydroxy-5beta-chol-6-en-24-oic acid | $C_{24}H_{38}O_4$ | 0.5764 | 62681-12-3 | 1.5915302 | 0.005818 | 0.2043877 |
| MEDN0381 | FFA(16:0) | $C_{16}H_{32}O_2$ | 0.9612 | - | 1.552695 | 0.010279 | 0.2561004 |
| MW0012811 | 1-Hexadecanoyl-2-(9Z-octadecenoyl)-sn-glycero-3-phospho-(1'-myo-inositol) | $C_{43}H_{81}O_{13}P$ | 0.9449 | - | 1.6355326 | 0.0206271 | 0.3234737 |
| MW0062462 | PS(18:0/22:5(7Z,10Z,13Z,16Z,19Z)) | $C_{46}H_{80}NO_{10}P$ | 0.9717 | - | 1.6367608 | 0.0017557 | 0.1371613 |
| MW0060169 | PE-NMe2(14:0/20:1(11Z)) | $C_{41}H_{80}NO_8P$ | 0.9236 | - | 1.4049795 | 0.0355864 | 0.3765515 |
| MW0058253 | 1-(9Z-octadecenoyl)-2-octadecanoyl-sn-glycero-3-phosphoethanolamine | $C_{41}H_{80}NO_8P$ | 0.9154 | - | 1.345607 | 0.0245026 | 0.3390985 |
| MW0060197 | PE-NMe2(14:1(9Z)/20:0) | $C_{41}H_{80}NO_8P$ | 0.9148 | - | 1.6526416 | 0.0136104 | 0.2668184 |
| MW0142386 | 2-Hydroxy-5-methyl-cis,cis-muconic acid | $C_7H_8O_5$ | 0.8877 | - | 1.5530053 | 0.0047822 | 0.1997729 |
| MW0058679 | 1-docosanoyl-2-(9Z-tetradecenoyl)-glycero-3-phosphoethanolamine | $C_{41}H_{80}NO_8P$ | 0.8815 | - | 1.5610542 | 0.0022515 | 0.1519189 |
| MW0137859 | Daidzein 4'-O-glucuronide | $C_{21}H_{18}O_{10}$ | 0.8702 | - | 1.3554612 | 0.0128276 | 0.2632176 |
| MW0059085 | 1-hexadecyl-2-(9Z-octadecenoyl)-sn-glycero-3-phosphoethanolamine | $C_{39}H_{78}NO_7P$ | 0.8694 | - | 1.6007476 | 0.0355724 | 0.3765515 |
| MW0061090 | 1,2-Dipalmitoyl-phosphatidyl-glycerole | $C_{38}H_{75}O_{10}P$ | 0.8652 | - | 1.6242368 | 0.0049627 | 0.2003897 |
| MW0142794 | 3alpha,7alpha-Dihydroxy-12-oxo-5beta-cholanate | $C_{24}H_{38}O_5$ | 0.862 | - | 1.510729 | 0.045951 | 0.3886256 |
| MW0059306 | PE-NMe(15:0/20:2(11Z,14Z)) | $C_{41}H_{78}NO_8P$ | 0.8521 | - | 1.653849 | 0.0153971 | 0.2833094 |
| MW0140575 | (2R)-3-sulfopropanediol | $C_3H_8O_5S$ | 0.8401 | - | 1.4278894 | 0.0459484 | 0.3886256 |
| MW0056865 | PC(15:0/20:4(8Z,11Z,14Z,17Z)) | $C_{43}H_{78}NO_8P$ | 0.8391 | - | 1.5731105 | 0.00461 | 0.1997729 |
| MW0006282 | Benzoyl phosphate | $C_7H_7O_5P$ | 0.838 | - | 1.5923309 | 0.0076767 | 0.234505 |
| MW0059246 | PE-NMe(14:0/20:0) | $C_{40}H_{80}NO_8P$ | 0.8326 | - | 1.4995774 | 0.0095424 | 0.2479621 |
| MW0152029 | Kikkanol D | $C_{15}H_{26}O_3$ | 0.8285 | - | 1.5424612 | 0.0439136 | 0.3886256 |
| MW0001371 | 1,4-Dihydroxy-2-naphthoic acid | $C_{11}H_8O_4$ | 0.8278 | - | 1.0689649 | 0.0461435 | 0.3886256 |
| MW0060366 | PE-NMe2(18:1(9Z)/18:1(9Z)) | $C_{43}H_{82}NO_8P$ | 0.8261 | - | 1.6514289 | 0.0148702 | 0.2823734 |
| MW0061385 | 1,2-Distearoyl-rac-glycero-3-phospho-(1'-sn-glycerol-3'-phosphate) | $C_{42}H_{84}O_{13}P_2$ | 0.8259 | - | 1.5475531 | 0.0030177 | 0.1612552 |

| | | | | | | | |
|---|---|---|---|---|---|---|---|
| MW0012462 | 5-[(3aR,4S,5S)-5-hydroxy-4-(4-methyl-3-oxooct-1-en-6-ynyl)-3,3a,4,5,6,6a-hexahydro-1H-pentalen-2-ylidene]pentanoic acid | $C_{22}H_{30}O_4$ | 0.8258 | - | 1.3590436 | 0.0478975 | 0.3904255 |
| MW0054484 | Lpa(0:0/18:2(9Z,12Z)) | $C_{21}H_{39}O_7P$ | 0.8253 | - | 1.5335515 | 0.044426 | 0.3886256 |
| MW0058309 | PE(18:2(9Z,12Z)/24:1(15Z)) | $C_{47}H_{88}NO_8P$ | 0.8175 | - | 1.4133751 | 0.0449091 | 0.3886256 |
| MW0058327 | PE(18:3(6Z,9Z,12Z)/20:1(11Z)) | $C_{43}H_{78}NO_8P$ | 0.8171 | - | 1.6401543 | 0.0024713 | 0.1519189 |
| MW0061141 | 1-Vaccenoyl-2-oleoyl-sn-glycero-3-phosphoglycerol | $C_{42}H_{79}O_{10}P$ | 0.8167 | - | 1.5882786 | 0.0044862 | 0.199493 |
| MW0056356 | PA(22:6(4Z,7Z,10Z,13Z,16Z,19Z)/14:1(9Z)) | $C_{39}H_{63}O_8P$ | 0.8015 | - | 1.4997064 | 0.0209019 | 0.3234737 |
| MW0055527 | [(2R)-2-octanoyloxy-3-phosphonooxypropyl] decanoate | $C_{21}H_{41}O_8P$ | 0.7996 | - | 1.4904095 | 0.0444678 | 0.3886256 |
| MW0056236 | PA(22:1(13Z)/22:6(4Z,7Z,10Z,13Z,16Z,19Z)) | $C_{47}H_{79}O_8P$ | 0.798 | - | 1.6282585 | 0.0041573 | 0.1921663 |
| MW0015258 | 9,10-Epoxystearic acid | $C_{18}H_{34}O_3$ | 0.7967 | - | 1.4649284 | 0.0427458 | 0.3886256 |
| MW0013917 | 3-alpha,20-alpha-Dihydroxy-5-beta-pregnane 3-glucuronide | $C_{27}H_{44}O_8$ | 0.7961 | - | 1.4020717 | 0.037606 | 0.3862864 |
| MW0063071 | PS(22:6(4Z,7Z,10Z,13Z,16Z,19Z)/22:5(4Z,7Z,10Z,13Z,16Z)) | $C_{50}H_{76}NO_{10}P$ | 0.7876 | - | 1.4510011 | 0.0030755 | 0.1612552 |
| MW0055672 | PA(18:0/20:3(8Z,11Z,14Z)) | $C_{41}H_{75}O_8P$ | 0.7867 | - | 1.5008988 | 0.0047662 | 0.1997729 |
| MW0054571 | LPE(0:0/18:0) | $C_{23}H_{48}NO_7P$ | 0.785 | - | 1.6532899 | 0.0052721 | 0.2008321 |
| MW0012945 | 1-Oleoylglycerophosphoserine | $C_{24}H_{46}NO_9P$ | 0.7821 | - | 1.5026665 | 0.0434027 | 0.3886256 |
| MW0059314 | PE-NMe(15:0/22:2(13Z,16Z)) | $C_{43}H_{82}NO_8P$ | 0.7759 | - | 1.6204182 | 0.0128157 | 0.2632176 |
| MW0060439 | PE-NMe2(18:3(6Z,9Z,12Z)/22:2(13Z,16Z)) | $C_{47}H_{84}NO_8P$ | 0.7688 | - | 1.3096125 | 0.0120339 | 0.2623196 |
| MW0059582 | PE-NMe(20:0/14:0) | $C_{40}H_{80}NO_8P$ | 0.7685 | - | 1.6131404 | 0.0048347 | 0.1997729 |
| MW0062753 | PS(20:3(5Z,8Z,11Z)/22:5(7Z,10Z,13Z,16Z,19Z)) | $C_{48}H_{78}NO_{10}P$ | 0.7683 | - | 1.6615393 | 0.0002022 | 0.0783406 |
| MW0058194 | 1-Octadecanoyl-2-eicosanoyl-sn-glycero-3-phosphoethanolamine | $C_{43}H_{86}NO_8P$ | 0.764 | - | 1.4956177 | 0.0050459 | 0.2008321 |
| MW0169519 | N-Acetyl-S-farnesyl-L-cysteine | $C_{20}H_{33}NO_3S$ | 0.7639 | - | 1.5567899 | 0.0186381 | 0.3059616 |
| MW0149444 | gamma-Glutamyl-alpha-aminobutyrate | $C_9H_{16}N_2O_5$ | 0.7637 | - | 1.4928026 | 0.0124448 | 0.2632176 |
| MW0142047 | 2,4-Dichloro-5-oxo-2,5-dihydro-2-furylacetic acid | $C_6H_4Cl_2O_4$ | 0.7624 | - | 1.5071488 | 0.0115767 | 0.2623196 |
| MW0058205 | PE(18:0/22:4(7Z,10Z,13Z,16Z)) | $C_{45}H_{82}NO_8P$ | 0.7623 | - | 1.5198582 | 0.0119563 | 0.2623196 |
| MW0063015 | PS(22:5(4Z,7Z,10Z,13Z,16Z)/22:6(4Z,7Z,10Z,13Z,16Z,19Z)) | $C_{50}H_{76}NO_{10}P$ | 0.7608 | - | 1.368649 | 0.0123799 | 0.2632176 |
| MW0058689 | PE(22:0/18:4(6Z,9Z,12Z,15Z)) | $C_{45}H_{82}NO_8P$ | 0.7591 | - | 1.542607 | 0.0306552 | 0.3611021 |
| MW0055627 | 1-hexadecanoyl-2-(13Z,16Z-docosadienoyl)-glycero-3-phosphate | $C_{41}H_{77}O_8P$ | 0.7586 | - | 1.6195913 | 0.0459301 | 0.3886256 |

| | | | | | | | |
|---|---|---|---|---|---|---|---|
| MW0013070 | 2,2,4,4-Tetramethyl-6-(1-oxobutyl)-1,3,5-cyclohexanetrione | $C_{14}H_{20}O_4$ | 0.7569 | - | 1.40477 | 0.0323028 | 0.3695268 |
| MW0059406 | PE-NMe(18:0/24:0) | $C_{48}H_{96}NO_8P$ | 0.7549 | - | 1.6438209 | 0.0274581 | 0.3490383 |
| MW0059407 | PE-NMe(18:0/24:1(15Z)) | $C_{48}H_{94}NO_8P$ | 0.752 | - | 1.3855696 | 0.0234766 | 0.3328885 |
| MEDN1231 | 1-O-vanillyl-β-D-glucose | $C_{14}H_{18}O_9$ | 0.7515 | - | 1.2198811 | 0.0489472 | 0.3958999 |
| MW0063032 | PS(22:5(7Z,10Z,13Z,16Z,19Z)/20:2(11Z,14Z)) | $C_{48}H_{80}NO_{10}P$ | 0.7509 | - | 1.6652851 | 0.0001582 | 0.0778407 |
| MW0054323 | Leukotriene C4-d5 | $C_{30}H_{47}N_3O_9S$ | 0.7499 | - | 1.401618 | 0.044557 | 0.3886256 |
| MW0147486 | (1S,2R,3S,4R,7R,9S,12R,14S,17R,18R,19R,21S,22S)-22-(2-hydroxypropan-2-yl)-3,8,8,17,19-pentamethyl-23,24-dioxaheptacyclo[19.2.1.01,18.03,17.04,14.07,12.012,14]tetracosane-2,9-diol | $C_{30}H_{48}O_5$ | 0.7498 | - | 1.2383512 | 0.0353152 | 0.3765515 |
| MW0062792 | PS(20:4(5Z,8Z,11Z,14Z)/18:1(11Z)) | $C_{44}H_{76}NO_{10}P$ | 0.7497 | - | 1.4880927 | 0.0073694 | 0.2311405 |
| MW0062968 | PS(22:4(7Z,10Z,13Z,16Z)/18:2(9Z,12Z)) | $C_{46}H_{78}NO_{10}P$ | 0.7487 | - | 1.6718187 | 0.0023721 | 0.1519189 |
| MW0062490 | PS(18:1(11Z)/22:5(4Z,7Z,10Z,13Z,16Z)) | $C_{46}H_{78}NO_{10}P$ | 0.7485 | - | 1.6410589 | 0.0014876 | 0.1314936 |
| MW0056242 | 1-(13Z,16Z-docosadienoyl)-2-hexadecanoyl-glycero-3-phosphate | $C_{41}H_{77}O_8P$ | 0.7482 | - | 1.5989626 | 0.0256234 | 0.3421107 |
| MW0061498 | Pgp(20:4(8Z,11Z,14Z,17Z)/20:1(11Z)) | $C_{46}H_{82}O_{13}P_2$ | 0.7471 | - | 1.6331514 | 0.0097474 | 0.2481354 |
| MW0063012 | PS(22:5(4Z,7Z,10Z,13Z,16Z)/22:4(7Z,10Z,13Z,16Z)) | $C_{50}H_{80}NO_{10}P$ | 0.746 | - | 1.6735236 | 0.0064073 | 0.2158577 |
| MW0061517 | Pgp(22:5(7Z,10Z,13Z,16Z,19Z)/18:1(11Z)) | $C_{46}H_{80}O_{13}P_2$ | 0.7454 | - | 1.6238214 | 0.0005331 | 0.0936303 |
| MW0060409 | PE-NMe2(18:2(9Z,12Z)/22:1(13Z)) | $C_{47}H_{88}NO_8P$ | 0.7424 | - | 1.4386574 | 0.0118372 | 0.2623196 |
| MW0150998 | His-Leu-Ser-Lys | $C_{21}H_{37}N_7O_6$ | 0.7407 | - | 1.5894163 | 0.0113424 | 0.2623196 |
| MW0060265 | PE-NMe2(16:0/22:2(13Z,16Z)) | $C_{45}H_{86}NO_8P$ | 0.7397 | - | 1.6650902 | 0.0070197 | 0.2262416 |
| MW0056264 | PA(22:2(13Z,16Z)/22:5(7Z,10Z,13Z,16Z,19Z)) | $C_{47}H_{79}O_8P$ | 0.7394 | - | 1.6605866 | 0.0089988 | 0.2450604 |
| MW0061495 | Pgp(20:4(5Z,8Z,11Z,14Z)/22:5(7Z,10Z,13Z,16Z,19Z)) | $C_{48}H_{78}O_{13}P_2$ | 0.7393 | - | 1.5996751 | 0.000399 | 0.0783406 |
| MW0056367 | PA(22:6(4Z,7Z,10Z,13Z,16Z,19Z)/20:0) | $C_{45}H_{77}O_8P$ | 0.7361 | - | 1.6641018 | 0.0158842 | 0.2861593 |
| MW0061475 | Pgp(20:2(11Z,14Z)/18:2(9Z,12Z)) | $C_{44}H_{80}O_{13}P_2$ | 0.7353 | - | 1.6474144 | 0.0087143 | 0.2410502 |
| MW0059381 | PE-NMe(18:0/15:0) | $C_{39}H_{78}NO_8P$ | 0.7346 | - | 1.6296285 | 0.0191507 | 0.3114651 |
| MW0062548 | PS(18:2(9Z,12Z)/22:4(7Z,10Z,13Z,16Z)) | $C_{46}H_{78}NO_{10}P$ | 0.731 | - | 1.6620227 | 0.0180015 | 0.2995365 |
| MW0057922 | 1-(1Z-octadecenyl)-2-(9Z-octadecenoyl)-sn-glycero-3-phosphocholine | $C_{44}H_{86}NO_7P$ | 0.7299 | - | 1.1405375 | 0.023527 | 0.3328885 |
| MW0063013 | PS(22:5(4Z,7Z,10Z,13Z,16Z)/22:5(4Z,7Z,10Z,13Z,16Z)) | $C_{50}H_{78}NO_{10}P$ | 0.7297 | - | 1.6628105 | 0.0157592 | 0.2861593 |

| | | | | | | | |
|---|---|---|---|---|---|---|---|
| MW0055039 | 1-Behenoyl-glycerol | $C_{25}H_{50}O_4$ | 0.7295 | - | 1.6317466 | 0.0307004 | 0.3611021 |
| MW0055731 | PA(18:1(9Z)/20:4(5Z,8Z,11Z,14Z)) | $C_{41}H_{71}O_8P$ | 0.7288 | - | 1.5143543 | 0.0307343 | 0.3611021 |
| MW0147548 | cis-4,10-13,16-Docosatetraenoic Acid methyl ester | $C_{23}H_{38}O_2$ | 0.7268 | - | 1.6051613 | 0.0346867 | 0.37436 |
| MW0055719 | 1-(9Z-octadecenoyl)-2-octadecanoyl-glycero-3-phosphate | $C_{39}H_{75}O_8P$ | 0.7242 | - | 1.6365262 | 0.0344184 | 0.37436 |
| MW0057446 | 1-Docosanoyl-2-hexadecanoyl-sn-glycero-3-phosphocholine | $C_{46}H_{92}NO_8P$ | 0.724 | - | 1.2755647 | 0.0495129 | 0.3962161 |
| MW0194314 | 5S-Hydroxy-6R-(S-gamma-glutamylcysteinyl)-7E,9E,11Z,14Z-eicosatetraenoic acid | $C_{28}H_{44}N_2O_8S$ | 0.7107 | - | 1.2080865 | 0.0470161 | 0.3894264 |
| MW0060744 | PE-NMe2(22:0/18:2(9Z,12Z)) | $C_{47}H_{90}NO_8P$ | 0.7084 | - | 1.6324929 | 0.0010919 | 0.1195787 |
| MW0059403 | PE-NMe(18:0/22:5(4Z,7Z,10Z,13Z,16Z)) | $C_{46}H_{82}NO_8P$ | 0.708 | - | 1.3981712 | 0.0263628 | 0.3447271 |
| MW0132477 | 3,4,5-trihydroxy-6-[4-[(E)-2-[2,3,5-trihydroxy-4-[(E)-3-methylbut-1-enyl]phenyl]ethenyl]phenoxy]oxane-2-carboxylic acid | $C_{25}H_{28}O_{10}$ | 0.7074 | - | 1.4779564 | 0.0055921 | 0.2008321 |
| MW0060305 | PE-NMe2(18:0/16:1(9Z)) | $C_{41}H_{80}NO_8P$ | 0.7073 | - | 1.5920348 | 0.022808 | 0.3328885 |
| MW0143414 | 4-Nitro-6-oxohepta-2,4-dienedioic acid | $C_7H_5NO_7$ | 0.6992 | - | 1.5570716 | 0.0297895 | 0.3611021 |
| MW0168221 | Contignasterol | $C_{29}H_{48}O_7$ | 0.6983 | - | 1.5512886 | 0.0166725 | 0.2928525 |
| MW0154653 | 2-Propanamine, 2-methyl-N-(phenylmethylene)-, N-oxide | $C_{11}H_{15}NO$ | 0.6973 | - | 1.654016 | 0.0003156 | 0.0783406 |
| MW0062927 | PS(22:1(13Z)/22:5(7Z,10Z,13Z,16Z,19Z)) | $C_{50}H_{86}NO_{10}P$ | 0.6964 | - | 1.6750469 | 0.0001054 | 0.0778407 |
| MW0142181 | 24-Epi-campesterol | $C_{28}H_{48}O$ | 0.6879 | - | 1.618139 | 0.0117717 | 0.2623196 |
| MW0012755 | 1-Acetoxy-2-hydroxy-5,12,15-heneicosatrien-4-one | $C_{23}H_{38}O_4$ | 0.6874 | - | 1.4137096 | 0.0282871 | 0.3536388 |
| MW0056911 | 1-Palmitoyl-2-(1-enyl-stearoyl)-sn-glycero-3-phosphocholine | $C_{42}H_{84}NO_7P$ | 0.6855 | - | 1.6515392 | 0.0007882 | 0.110753 |
| MW0125295 | N'-(1-Hexyl-2-oxoindolin-3-ylidene)benzohydrazide | $C_{21}H_{23}N_3O_2$ | 0.683 | - | 1.6628661 | 0.0005892 | 0.0940775 |
| MW0060277 | PE-NMe2(16:1(9Z)/18:0) | $C_{41}H_{80}NO_8P$ | 0.6816 | - | 1.4921669 | 0.0143003 | 0.2760263 |
| MEDN1262 | LPS(18:2) | $C_{24}H_{44}NO_9P$ | 0.6805 | - | 1.2802063 | 0.035459 | 0.3765515 |
| MW0062458 | (2S)-2-amino-3-[[(2R)-2-[(Z)-docos-13-enoyl]oxy-3-octadecanoyloxypropoxy]-hydroxyphosphoryl]oxypropanoic acid | $C_{46}H_{88}NO_{10}P$ | 0.6786 | - | 1.6207016 | 0.0030646 | 0.1612552 |
| MW0060353 | PE-NMe2(18:1(11Z)/22:4(7Z,10Z,13Z,16Z)) | $C_{47}H_{84}NO_8P$ | 0.6782 | - | 1.3170902 | 0.0144957 | 0.2782695 |
| MW0062982 | PS(22:4(7Z,10Z,13Z,16Z)/22:2(13Z,16Z)) | $C_{50}H_{86}NO_{10}P$ | 0.6778 | - | 1.5775824 | 0.001757 | 0.1371613 |
| MW0056108 | PA(20:5(5Z,8Z,11Z,14Z,17Z)/18:4(6Z,9Z,12Z,15Z)) | $C_{41}H_{63}O_8P$ | 0.67 | - | 1.2341873 | 0.0455974 | 0.3886256 |
| MW0062176 | Prieuranin acetate | $C_{40}H_{52}O_{17}$ | 0.6681 | - | 1.6214084 | 0.0152571 | 0.2833094 |

| | | | | | | | |
|---|---|---|---|---|---|---|---|
| MW0059304 | PE-NMe(15:0/20:0) | $C_{41}H_{82}NO_8P$ | 0.6628 | - | 1.5519416 | 0.0474483 | 0.3894264 |
| MW0111860 | 1-Phenyl-1,3-heptadecanedione | $C_{23}H_{36}O_2$ | 0.6619 | - | 1.5408677 | 0.0467507 | 0.3886256 |
| MW0062985 | PS(22:4(7Z,10Z,13Z,16Z)/22:5(7Z,10Z,13Z,16Z,19Z)) | $C_{50}H_{80}NO_{10}P$ | 0.6557 | - | 1.6656499 | 0.0086896 | 0.2410502 |
| MW0060270 | PE-NMe2(16:0/24:0) | $C_{47}H_{94}NO_8P$ | 0.655 | - | 1.3535287 | 0.046629 | 0.3886256 |
| MW0062539 | PS(18:2(9Z,12Z)/20:2(11Z,14Z)) | $C_{44}H_{78}NO_{10}P$ | 0.6501 | - | 1.550011 | 0.0229249 | 0.3328885 |
| MW0108751 | N-docosahexaenoyl phenylalanine | $C_{31}H_{41}NO_3$ | 0.6497 | - | 1.3215201 | 0.0298034 | 0.3611021 |
| MW0056551 | [(2R)-1-(10-methylundecanoyloxy)-3-phosphonooxypropan-2-yl] 10-methyldodecanoate | $C_{28}H_{55}O_8P$ | 0.6452 | - | 1.6506111 | 0.0001773 | 0.0778407 |
| MW0060301 | PE-NMe2(18:0/14:0) | $C_{39}H_{78}NO_8P$ | 0.6434 | - | 1.4689397 | 0.005807 | 0.2043877 |
| MW0062513 | PS(18:1(9Z)/20:4(5Z,8Z,11Z,14Z)) | $C_{44}H_{76}NO_{10}P$ | 0.6422 | - | 1.4055972 | 0.039517 | 0.3886256 |
| MW0049041 | 1-Octadecanoyl-cyclophosphatidic acid | $C_{21}H_{41}O_6P$ | 0.637 | - | 1.4046572 | 0.0136713 | 0.2668184 |
| MW0003458 | 3-(3,5-dihydroxyphenyl)-1-propanoic acid sulphate | $C_9H_{10}O_7S$ | 0.6354 | - | 1.4277941 | 0.0343276 | 0.37436 |
| MW0062486 | (2S)-2-amino-3-[[(2R)-2-docosanoyloxy-3-[(Z)-octadec-11-enoyl]oxypropoxy]-hydroxyphosphoryl]oxypropanoic acid | $C_{46}H_{88}NO_{10}P$ | 0.6305 | - | 1.3916254 | 0.0115197 | 0.2623196 |
| MW0056874 | Phosphatidylcholine(15:0/24:0) | $C_{47}H_{94}NO_8P$ | 0.6303 | - | 1.4086534 | 0.001818 | 0.1388419 |
| MW0154797 | Oxaloglutarate | $C_7H_8O_7$ | 0.629 | - | 1.6642764 | 0.0001113 | 0.0778407 |
| MW0060880 | PE-NMe2(22:5(4Z,7Z,10Z,13Z,16Z)/24:1(15Z)) | $C_{53}H_{94}NO_8P$ | 0.6243 | - | 1.5685978 | 0.0099946 | 0.2522046 |
| MW0012810 | 1-hexadecanoyl-2-(9Z,12Z-octadecadienoyl)-sn-glycero-3-phospho-D-myo-inositol | $C_{43}H_{79}O_{13}P$ | 0.6207 | - | 1.4235735 | 0.0223549 | 0.3285893 |
| MW0123212 | Carazolol | $C_{18}H_{22}N_2O_2$ | 0.6188 | - | 1.3475251 | 0.0373446 | 0.3852945 |
| MW0125613 | N-Coumaroyl serotonin | $C_{19}H_{18}N_2O_3$ | 0.6099 | - | 1.6006139 | 0.0436289 | 0.3886256 |
| MW0109998 | Trandolaprilat | $C_{22}H_{30}N_2O_5$ | 0.6079 | - | 1.4221098 | 0.0100509 | 0.2522046 |
| MW0061621 | Physagulin F | $C_{30}H_{40}O_9$ | 0.6055 | - | 1.3720767 | 0.0264948 | 0.3447271 |
| MW0062888 | PS(22:0/20:3(5Z,8Z,11Z)) | $C_{48}H_{88}NO_{10}P$ | 0.6027 | - | 1.3811902 | 0.0073293 | 0.2311405 |
| MW0055946 | PA(20:1(11Z)/22:5(4Z,7Z,10Z,13Z,16Z)) | $C_{45}H_{77}O_8P$ | 0.6005 | - | 1.657483 | 0.0095376 | 0.2479621 |
| MW0110954 | Chlorsulfuron | $C_{12}H_{12}ClN_5O_4S$ | 0.596 | - | 1.4284092 | 0.0346918 | 0.37436 |
| MW0159719 | Di-trans,poly-cis-pentaprenyl diphosphate | $C_{25}H_{44}O_7P_2$ | 0.595 | - | 1.5394804 | 0.0135206 | 0.2668184 |
| MW0002199 | 2,4,6-Trichlorophenol | $C_6H_3Cl_3O$ | 0.5889 | - | 1.5383182 | 0.0069098 | 0.2247597 |
| MW0058301 | 1-(9Z,12Z-octadecadienoyl)-2-docosanoyl-glycero-3-phosphoethanolamine | $C_{45}H_{86}NO_8P$ | 0.5882 | - | 1.6383877 | 0.0047477 | 0.1997729 |
| MW0057430 | PC(20:5(5Z,8Z,11Z,14Z,17Z)/22:0) | $C_{50}H_{90}NO_8P$ | 0.5824 | - | 1.6254449 | 0.0032508 | 0.1655091 |

| | | | | | | | | |
|---|---|---|---|---|---|---|---|---|
| MW0003662 | 3,5,7-trihydroxy-2-(3-hydroxyphenyl)-5H-chromen-5-yl | $C_{15}H_{11}O_5$ | 0.5813 | - | 1.4003619 | 0.0335488 | 0.37436 |
| MW0055727 | 1-(9Z-octadecenoyl)-2-(11Z-eicosenoyl)-glycero-3-phosphate | $C_{41}H_{77}O_8P$ | 0.5787 | - | 1.6497496 | 0.0406648 | 0.3886256 |
| MW0139218 | Osthole | $C_{15}H_{16}O_3$ | 0.5779 | - | 1.5693446 | 0.0024467 | 0.1519189 |
| MW0056445 | [(2R)-1-octanoyloxy-3-phosphonooxypropan-2-yl] tetradecanoate | $C_{25}H_{49}O_8P$ | 0.5751 | - | 1.4675898 | 0.0313942 | 0.3639866 |
| MW0012803 | 1-Heneicosanoyl-glycero-3-phosphate | $C_{24}H_{49}O_7P$ | 0.5746 | - | 1.581562 | 0.0125893 | 0.2632176 |
| MW0127618 | (-)-Gallocatechin | $C_{15}H_{14}O_7$ | 0.5692 | - | 1.6376967 | 0.0002577 | 0.0783406 |
| MW0060325 | PE-NMe2(18:0/22:5(4Z,7Z,10Z,13Z,16Z)) | $C_{47}H_{84}NO_8P$ | 0.5671 | - | 1.6072582 | 0.0042212 | 0.1925875 |
| MW0005886 | 8-hydroxy-2-(2-hydroxy-4-methylpent-3-en-1-yl)-2-methyl-2H-chromene-5-carboxylic acid | $C_{17}H_{20}O_5$ | 0.5503 | - | 1.4371385 | 0.0408788 | 0.3886256 |
| MW0140217 | (1R,2R)-3-[(1,2-Dihydro-2-hydroxy-1-naphthalenyl)thio]-2-oxopropanoic acid | $C_{13}H_{12}O_4S$ | 0.5461 | - | 1.6682094 | 0.0005293 | 0.0936303 |
| MW0113762 | 6"-O-Acetylholocalin | $C_{16}H_{19}NO_8$ | 0.545 | - | 1.6189361 | 0.0113146 | 0.2623196 |
| MW0115362 | 2-(Ethylamino)-5-(hydroxymethyl)-5,6,7,7a-tetrahydro-3aH-pyrano[3,2-d][1,3]thiazole-6,7-diol | $C_9H_{16}N_2O_4S$ | 0.5389 | - | 1.5844717 | 0.0208543 | 0.3234737 |
| MW0058702 | PE(22:0/22:5(4Z,7Z,10Z,13Z,16Z)) | $C_{49}H_{88}NO_8P$ | 0.5211 | - | 1.4360679 | 0.0338028 | 0.37436 |
| MW0053421 | Ganoderic Acid J | $C_{30}H_{42}O_7$ | 0.5024 | - | 1.606314 | 0.0035449 | 0.1711678 |
| MW0060358 | PE-NMe2(18:1(11Z)/24:1(15Z)) | $C_{49}H_{94}NO_8P$ | 0.5004 | - | 1.3915593 | 0.0367465 | 0.3830579 |
| MW0152904 | Lys-Asp-Leu-Glu | $C_{21}H_{37}N_5O_9$ | 0.9048 | - | 1.5552501 | 0.0479034 | 0.3904255 |
| MW0066564 | TG(14:0/14:1(9Z)/16:0) | $C_{47}H_{88}O_6$ | 0.8635 | - | 1.5305605 | 0.0434717 | 0.3886256 |
| MW0049724 | 1-Pentadecanoyl-2-nervonoyl-sn-glycerol | $C_{42}H_{80}O_5$ | 0.8535 | - | 1.6496443 | 0.0201973 | 0.3225146 |
| MW0050886 | 1-docosanoyl-2-(7Z,10Z,13Z,16Z,19Z-docosapentaenoyl)-sn-glycerol | $C_{47}H_{82}O_5$ | 0.8336 | - | 1.2247431 | 0.0245178 | 0.3390985 |
| MW0050884 | 1-docosanoyl-2-(7Z,10Z,13Z,16Z-docosatetraenoyl)-sn-glycerol | $C_{47}H_{84}O_5$ | 0.8138 | - | 1.5035797 | 0.0083332 | 0.2377131 |
| MW0145898 | Asn-Thr-Asp-Arg | $C_{18}H_{32}N_8O_9$ | 0.7815 | - | 1.3663364 | 0.0120967 | 0.2623196 |
| MW0011479 | (9S,10S)-10-hydroxy-9-(phosphonooxy)octadecanoic acid | $C_{18}H_{37}O_7P$ | 0.7809 | - | 1.4866736 | 0.02711 | 0.347582 |
| MW0157845 | Thr-Tyr-Leu-Glu | $C_{24}H_{36}N_4O_9$ | 0.7535 | - | 1.3714283 | 0.0455213 | 0.3886256 |
| MW0063143 | PS(DiMe(11,5)/DiMe(11,3)) | $C_{48}H_{82}NO_{12}P$ | 0.7408 | - | 1.5858602 | 0.0019133 | 0.1391314 |
| MW0151553 | Ile-Phe-Val-Lys | $C_{26}H_{43}N_5O_5$ | 0.6976 | - | 1.4399309 | 0.0379331 | 0.3868119 |
| MW0151470 | Ile-Ile-Glu-Glu-Val | $C_{27}H_{47}N_5O_{10}$ | 0.6904 | - | 1.4640976 | 0.0309161 | 0.3620271 |

| | | | | | | | |
|---|---|---|---|---|---|---|---|
| MW0050864 | 1-Behenoyl-2-stearoyl-sn-glycerol | $C_{43}H_{84}O_5$ | 0.6887 | - | 1.6058758 | 0.0216555 | 0.3269138 |
| MW0049643 | 1-Pentadecanoyl-3-palmitoyl-sn-glycerol | $C_{34}H_{66}O_5$ | 0.6867 | - | 1.4313938 | 0.0120544 | 0.2623196 |
| MW0143565 | 5Androstan-3ol-17-one sulfate | $C_{19}H_{30}O_5S$ | 0.6762 | - | 1.6285254 | 0.0029478 | 0.1612552 |
| MW0049825 | 1-hexadecanoyl-2-(7Z,10Z,13Z,16Z-docosatetraenoyl)-sn-glycerol | $C_{41}H_{72}O_5$ | 0.6745 | - | 1.4905817 | 0.0154034 | 0.2833094 |
| MW0052240 | [(2S)-3-hydroxy-2-octanoyloxypropyl] 20-methylhenicosanoate | $C_{33}H_{64}O_5$ | 0.6739 | - | 1.6020252 | 0.016905 | 0.2939969 |
| MW0068275 | TG(14:1(9Z)/18:0/20:4(8Z,11Z,14Z,17Z)) | $C_{55}H_{96}O_6$ | 0.6675 | - | 1.4482065 | 0.0264783 | 0.3447271 |
| MW0141840 | 1-Stearoyl-2-Docosahexaenoyl-sn-Glycero-3-[Phospho-rac-(1-glycerol)] | $C_{46}H_{79}O_{10}P$ | 0.6339 | - | 1.6683014 | 0.0036894 | 0.1751465 |
| MW0062464 | PS(18:0/24:0) | $C_{48}H_{94}NO_{10}P$ | 0.6254 | - | 1.4769532 | 0.0135431 | 0.2668184 |
| MW0061618 | [(1S,2R,6S,7R,9R,11R,12S,13S,14S,16R,17S)-16-[(1R)-1-[(2R)-4,5-dimethyl-6-oxo-2,3-dihydropyran-2-yl]ethyl]-6,12-dihydroxy-2,17-dimethyl-3-oxo-8,15-dioxahexacyclo[9.8.0.02,7.07,9.012,17.014,16]nonadec-4-en-13-yl] acetate | $C_{30}H_{38}O_9$ | 0.6156 | - | 1.4272649 | 0.0178692 | 0.2994213 |
| MW0016444 | Cdp-DG(18:0/16:0) | $C_{46}H_{85}N_3O_{15}P_2$ | 0.5836 | - | 1.5354517 | 0.0054514 | 0.2008321 |
| MW0063197 | PS(MonoMe(13,5)/DiMe(11,3)) | $C_{49}H_{84}NO_{12}P$ | 0.5611 | - | 1.6715558 | 0.0081554 | 0.2377131 |
| MW0063150 | PS(DiMe(11,5)/MonoMe(13,5)) | $C_{51}H_{88}NO_{12}P$ | 0.5498 | - | 1.3249157 | 0.0397286 | 0.3886256 |
| MW0165467 | (3S,4aR,6aR,6aR,6bR,8aS,12aS,14aR,14bR)-4,4,6a,6b,9,9,11,12a,14b-nonamethyl-1,2,3,4a,5,6,6a,7,8,8a,10,11,12,13,14,14a-hexadecahydropicen-3-ol | $C_{31}H_{54}O$ | 0.5293 | - | 1.3718345 | 0.0368745 | 0.3832543 |

**Table S9.** Differentially expressed proteins

| Accession | Gene | P-value | FDR | Accession | Gene | P-value | FDR |
|---|---|---|---|---|---|---|---|
| A0A096MK61 | Crtap | 0.0450602 | 0.8488054 | O08836 | Igbp1 | 0.0154684 | 0.8488054 |
| A0A0B4J2J0 | Fcgr1a | 0.0371946 | 0.8488054 | O35293 | Cyp2f2 | 0.0147279 | 0.8488054 |
| A0A0G2JSS9 | Atl3 | 0.0205738 | 0.8488054 | O35547 | Acsl4 | 0.0330519 | 0.8488054 |
| A0A0G2JTR4 | Abr | 0.0461349 | 0.8488054 | O35777 | Magt1 | 0.0030052 | 0.7679417 |

| | | | | | | | |
|---|---|---|---|---|---|---|---|
| A0A0G2JUP3 | Obscn | 0.0269004 | 0.8488054 | O35910 | Slc16a3 | 0.0027625 | 0.7679417 |
| A0A0G2JVB2 | Scube2 | 0.0012156 | 0.67774 | O54747 | Pold1 | 0.0433094 | 0.8488054 |
| A0A0G2JXD9 | Dop1b | 0.0325978 | 0.8488054 | O54748 | Stk3 | 0.042021 | 0.8488054 |
| A0A0G2JXY9 | Epn2 | 0.0257183 | 0.8488054 | O54922 | Exoc7 | 0.0405494 | 0.8488054 |
| A0A0G2JY22 | Dip2b | 3.93E-005 | 0.1574955 | O55000 | Ppp1r10 | 0.0101189 | 0.8488054 |
| A0A0G2JZ33 | Lnpk | 0.0456999 | 0.8488054 | O70467 | Prmt3 | 0.0341885 | 0.8488054 |
| A0A0G2K047 | Acss3 | 0.0057975 | 0.8488054 | O70593 | Sgta | 0.0104868 | 0.8488054 |
| A0A0G2K0I6 | Cdh5 | 0.0240285 | 0.8488054 | O88280 | Slit3 | 0.0275709 | 0.8488054 |
| A0A0G2K1D2 | Rap1gds1 | 0.0463005 | 0.8488054 | O88600 | Hspa4 | 0.0482024 | 0.8488054 |
| A0A0G2K1L2 | Phykpl | 0.0323578 | 0.8488054 | O88664 | Taok1 | 0.0458883 | 0.8488054 |
| A0A0G2K1M7 | Ano6 | 0.049653 | 0.8516885 | O88780 | Klk8 | 0.0263261 | 0.8488054 |
| A0A0G2K214 | Zc3h7b | 0.0454325 | 0.8488054 | O89046 | Coro1b | 0.0060068 | 0.8488054 |
| A0A0G2K278 | Gpx2 | 0.0289159 | 0.8488054 | P04182 | Oat | 0.0378499 | 0.8488054 |
| A0A0G2K2H0 | Axl | 0.0224168 | 0.8488054 | P04276 | Gc | 0.0478055 | 0.8488054 |
| A0A0G2K320 | Siglec1 | 0.0072212 | 0.8488054 | P04961 | Pcna | 0.0287607 | 0.8488054 |
| A0A0G2K363 | Zeb2 | 0.0007566 | 0.6684691 | P05712 | Rab2a | 0.0455866 | 0.8488054 |
| A0A0G2K3W1 | Vwa8 | 0.0175979 | 0.8488054 | P07483 | Fabp3 | 0.0245677 | 0.8488054 |
| A0A0G2K6A7 | Zdhhc13 | 0.0367003 | 0.8488054 | P07896 | Ehhadh | 0.0248251 | 0.8488054 |
| A0A0G2K6J6 | Ipo8 | 0.0079502 | 0.8488054 | P07936 | Gap43 | 0.0192462 | 0.8488054 |
| A0A0G2K719 | Ddx3x | 0.0482887 | 0.8488054 | P08050 | Gja1 | 0.0265686 | 0.8488054 |
| A0A0G2K8D6 | Dcun1d1 | 0.0408447 | 0.8488054 | P08934 | Kng1 | 0.0304017 | 0.8488054 |
| A0A0G2K8I0 | Sh3bgrl2 | 0.0141111 | 0.8488054 | P09215 | Prkcd | 0.0211524 | 0.8488054 |
| A0A0G2K9J1 | Syncrip | 0.008774 | 0.8488054 | P09330 | Prps2 | 0.0061141 | 0.8488054 |
| A0A0G2K9Y5 | Hrg | 0.0480816 | 0.8488054 | P09527 | Rab7a | 0.0134268 | 0.8488054 |
| A0A0G2KA71 | Nectin2 | 0.0098975 | 0.8488054 | P10759 | Ampd1 | 0.0447518 | 0.8488054 |
| A0A0G2KBC4 | Csf1r | 0.036749 | 0.8488054 | P10860 | Glud1 | 0.045984 | 0.8488054 |
| A0A0H2UI05 | Cfap20 | 0.0223122 | 0.8488054 | P11505 | Atp2b1 | 0.0102896 | 0.8488054 |
| A0A0H2UI39 | Apoc3 | 0.0196101 | 0.8488054 | P12346 | Tf | 0.0222778 | 0.8488054 |
| A0A0U1RS25 | Upf1 | 0.0063344 | 0.8488054 | P12368 | Prkar2a | 0.0359633 | 0.8488054 |
| A0A140TAF0 | Tpm3 | 0.0399861 | 0.8488054 | P13084 | Npm1 | 0.0324712 | 0.8488054 |
| A0A1W2Q642 | Pi16 | 0.0078683 | 0.8488054 | P13286 | Phkg1 | 0.0444448 | 0.8488054 |
| A0A1W2Q6E6 | Cyp4f39 | 0.0175339 | 0.8488054 | P13601 | Aldh1a7 | 0.0189436 | 0.8488054 |
| A0A8I5XVM7 | Ociad2 | 0.0035508 | 0.8375748 | P13676 | Apeh | 0.0299537 | 0.8488054 |
| A0A8I5XVS2 | Mrc1 | 0.0315678 | 0.8488054 | P13852 | Prnp | 0.0173395 | 0.8488054 |

| | | | | | | | |
|---|---|---|---|---|---|---|---|
| A0A8I5XW36 | Wtap | 0.017311 | 0.8488054 | P14659 | Hspa2 | 0.0406974 | 0.8488054 |
| A0A8I5Y087 | Mnda | 0.0267298 | 0.8488054 | P15337 | Creb1 | 0.0043998 | 0.8488054 |
| A0A8I5Y1A6 | Ctnnal1 | 0.036974 | 0.8488054 | P15399 | Pbsn | 0.0148819 | 0.8488054 |
| A0A8I5Y1J2 | ENSRNOG00000067128 | 0.0431523 | 0.8488054 | P17988 | Sult1a1 | 0.0266624 | 0.8488054 |
| A0A8I5Y6L2 | Postn | 0.0276737 | 0.8488054 | P19999 | Mbl1 | 0.0108685 | 0.8488054 |
| A0A8I5Y6U5 | Srp14 | 0.0168059 | 0.8488054 | P20417 | Ptpn1 | 0.0370183 | 0.8488054 |
| A0A8I5Y708 | Stk4 | 0.0261624 | 0.8488054 | P20611 | Acp2 | 0.003229 | 0.7921378 |
| A0A8I5Y735 | ENSRNOG00000066904 | 0.0361096 | 0.8488054 | P20761 | Igh-1a | 0.0163079 | 0.8488054 |
| A0A8I5ZJE5 | Nexn | 0.0461876 | 0.8488054 | P21533 | Rpl6 | 0.0254255 | 0.8488054 |
| A0A8I5ZJM6 | LOC100365438 | 0.0427747 | 0.8488054 | P27435 | Tpsab1 | 0.0312731 | 0.8488054 |
| A0A8I5ZKB4 | Ehbp1 | 0.0118429 | 0.8488054 | P27605 | Hprt1 | 0.0376921 | 0.8488054 |
| A0A8I5ZLN6 | Larp4 | 0.0163536 | 0.8488054 | P30009 | Marcks | 0.0151036 | 0.8488054 |
| A0A8I5ZLW9 | ENSRNOG00000063672 | 0.0123025 | 0.8488054 | P31722 | C1qc | 0.0374875 | 0.8488054 |
| A0A8I5ZM48 | ENSRNOG00000062392 | 0.0301515 | 0.8488054 | P32089 | Slc25a1 | 0.0114813 | 0.8488054 |
| A0A8I5ZM61 | Rnasek | 0.0192105 | 0.8488054 | P32577 | Csk | 0.0217346 | 0.8488054 |
| A0A8I5ZMT9 | Ppih | 0.0417811 | 0.8488054 | P34080 | Aqp2 | 0.0060295 | 0.8488054 |
| A0A8I5ZNF3 | Ncf4 | 0.0263774 | 0.8488054 | P35213 | Ywhab | 0.0342359 | 0.8488054 |
| A0A8I5ZQL5 | Acad8 | 0.0276383 | 0.8488054 | P35280 | Rab8a | 0.0450108 | 0.8488054 |
| A0A8I5ZQQ3 | Pdpr | 0.0404872 | 0.8488054 | P35704 | Prdx2 | 0.0352182 | 0.8488054 |
| A0A8I5ZQQ5 | Smarca5 | 0.024223 | 0.8488054 | P36365 | Fmo1 | 0.0424306 | 0.8488054 |
| A0A8I5ZR52 | Hspa9 | 0.0457471 | 0.8488054 | P36860 | Ralb | 0.0155161 | 0.8488054 |
| A0A8I5ZRU2 | ENSRNOG00000063550 | 0.0190191 | 0.8488054 | P47967 | Lgals5 | 0.0260007 | 0.8488054 |
| A0A8I5ZRY8 | Papln | 0.0128382 | 0.8488054 | P48004 | Psma7 | 0.0338006 | 0.8488054 |
| A0A8I5ZTN7 | Gbp5 | 0.0343201 | 0.8488054 | P50237 | Sult1c1 | 0.0247075 | 0.8488054 |
| A0A8I5ZU77 | Dglucy | 0.020678 | 0.8488054 | P53813 | Pros1 | 0.0241154 | 0.8488054 |
| A0A8I5ZUH5 | Plekha6 | 0.0024733 | 0.7679417 | P60123 | Ruvbl1 | 0.0214195 | 0.8488054 |
| A0A8I5ZVC3 | Ap2a1 | 0.016389 | 0.8488054 | P62329 | Tmsb4x | 0.0295053 | 0.8488054 |
| A0A8I5ZVT2 | Fndc3a | 0.0484399 | 0.8488054 | P62630 | Eef1a1 | 0.0280839 | 0.8488054 |
| A0A8I5ZW20 | Ubtd2 | 0.000175 | 0.3578201 | P62819 | S100a3 | 0.026833 | 0.8488054 |
| A0A8I5ZXD6 | Fam114a1 | 0.0134505 | 0.8488054 | P62828 | Ran | 0.0339556 | 0.8488054 |
| A0A8I5ZYC7 | Apbb2 | 0.0237044 | 0.8488054 | P62864 | Fau | 0.0419002 | 0.8488054 |
| A0A8I6A183 | Ccar1 | 0.0456252 | 0.8488054 | P62963 | Pfn1 | 0.0486781 | 0.8505497 |
| A0A8I6A1E0 | Usp8 | 0.0263352 | 0.8488054 | P63012 | Rab3a | 0.0225432 | 0.8488054 |
| A0A8I6A3C4 | Vps39 | 0.0158702 | 0.8488054 | P63025 | Vamp3 | 0.0223986 | 0.8488054 |

| | | | | | | | |
|---|---|---|---|---|---|---|---|
| A0A8I6A4A4 | Lman2l | 0.0354017 | 0.8488054 | P67999 | Rps6kb1 | 0.0318841 | 0.8488054 |
| A0A8I6A4K3 | Nap1l1 | 0.0270369 | 0.8488054 | P81799 | Nagk | 0.0257079 | 0.8488054 |
| A0A8I6A583 | ENSRNOG00000064207 | 0.0393623 | 0.8488054 | P83732 | Rpl24 | 0.0039894 | 0.8488054 |
| A0A8I6A5E1 | Smarcc2 | 0.0437588 | 0.8488054 | P85970 | Arpc2 | 0.008475 | 0.8488054 |
| A0A8I6A6S3 | Psmd1 | 0.0301406 | 0.8488054 | P97603 | Neo1 | 0.0446909 | 0.8488054 |
| A0A8I6A7C9 | Actr3b | 0.0322719 | 0.8488054 | Q01579 | Gstt1 | 0.0226209 | 0.8488054 |
| A0A8I6A9G4 | ENSRNOG00000068610 | 0.0059505 | 0.8488054 | Q01714 | Sp1 | 0.0231276 | 0.8488054 |
| A0A8I6A9V7 | Vps26c | 0.0114086 | 0.8488054 | Q02293 | Fntb | 0.013435 | 0.8488054 |
| A0A8I6A9Y1 | U2af2 | 0.0107201 | 0.8488054 | Q04631 | Fnta | 0.0489203 | 0.8510227 |
| A0A8I6AB22 | ENSRNOG00000067079 | 0.0256749 | 0.8488054 | Q05982 | Nme1 | 0.0395735 | 0.8488054 |
| A0A8I6ABA8 | Rabgef1 | 0.0493599 | 0.8516885 | Q07266 | Dbn1 | 0.043273 | 0.8488054 |
| A0A8I6ABG6 | Tln1 | 0.013179 | 0.8488054 | Q08163 | Cap1 | 0.0279925 | 0.8488054 |
| A0A8I6AC62 | Slc43a1 | 0.0143926 | 0.8488054 | Q0PMD2 | Antxr1 | 0.0149708 | 0.8488054 |
| A0A8I6AD83 | Gmfg | 0.0465024 | 0.8488054 | Q1RP74 | Tbcb | 0.0148568 | 0.8488054 |
| A0A8I6AEG4 | Ndufv3 | 0.0041397 | 0.8488054 | Q2HWF0 | Fnbp1l | 0.0226362 | 0.8488054 |
| A0A8I6AEL3 | Hdlbp | 0.0192778 | 0.8488054 | Q32KJ5 | Gns | 0.0346029 | 0.8488054 |
| A0A8I6AEV8 | Bola2l2 | 0.0262231 | 0.8488054 | Q3B8Q2 | Eif4a3 | 0.0242464 | 0.8488054 |
| A0A8I6AFC5 | Hpf1 | 0.0052311 | 0.8488054 | Q3KR92 | Tpst1 | 0.0010814 | 0.6684691 |
| A0A8I6AG33 | Tnpo1 | 0.0383871 | 0.8488054 | Q3KRE8 | Tubb2b | 0.0014975 | 0.7064639 |
| A0A8I6AG83 | Rpp30 | 0.0375401 | 0.8488054 | Q3ZAV4 | Gimap9 | 0.0443894 | 0.8488054 |
| A0A8I6AHV2 | ENSRNOG00000062608 | 0.0255801 | 0.8488054 | Q498M4 | Wdr5 | 0.0102492 | 0.8488054 |
| A0A8I6AI35 | Eftud2 | 0.0195354 | 0.8488054 | Q4KLH5 | Agfg1 | 0.0169887 | 0.8488054 |
| A0A8I6AIQ9 | Myh13 | 0.0302458 | 0.8488054 | Q4KM35 | Psmb10 | 0.0224788 | 0.8488054 |
| A0A8I6AJV7 | Safb2 | 0.0237872 | 0.8488054 | Q4QR73 | Dnaja4 | 0.0380138 | 0.8488054 |
| A0A8I6AKK7 | Pitpnc1 | 0.0330939 | 0.8488054 | Q4V7E8 | Lrrfip2 | 0.0481191 | 0.8488054 |
| A0A8I6ALC2 | Baz1b | 0.0037884 | 0.8488054 | Q4V8C7 | Prkra | 0.0382475 | 0.8488054 |
| A0A8I6ALW9 | ENSRNOG00000065952 | 0.0294999 | 0.8488054 | Q4V8K1 | Steap4 | 0.0404677 | 0.8488054 |
| A0A8I6AM24 | Ap3d1 | 0.0277476 | 0.8488054 | Q5BJU0 | Rras2 | 0.0351765 | 0.8488054 |
| A0A8I6AM26 | Srrt | 0.0073396 | 0.8488054 | Q5BKC9 | Ngef | 0.010517 | 0.8488054 |
| A0A8I6ANN1 | ENSRNOG00000067233 | 0.0458823 | 0.8488054 | Q5EGZ1 | Ace2 | 0.0219764 | 0.8488054 |
| A0A8I6APC0 | Ighm | 0.0191634 | 0.8488054 | Q5FWT9 | Sike1 | 0.0025498 | 0.7679417 |
| A0A8I6AQI7 | Serpinf2 | 0.0304252 | 0.8488054 | Q5HZE4 | Mri1 | 0.0112676 | 0.8488054 |
| A0A8I6AR59 | Aldh3b3 | 0.0021389 | 0.7679417 | Q5HZV9 | Ppp1r7 | 0.011686 | 0.8488054 |
| A0A8I6ATZ0 | Cfhr2 | 0.009822 | 0.8488054 | Q5I034 | Custos | 0.0391587 | 0.8488054 |

| | | | | | | | |
|---|---|---|---|---|---|---|---|
| A0A8I6AV37 | Prtfdc1 | 0.0461574 | 0.8488054 | Q5QE78 | Aox2 | 0.0266262 | 0.8488054 |
| A0A8I6AV84 | Zyx | 0.0016444 | 0.7203686 | Q5QE79 | Aox4 | 0.0207215 | 0.8488054 |
| A0A8I6G1P7 | ENSRNOG00000062657 | 0.025088 | 0.8488054 | Q5RJK5 | Cbx3 | 0.0301084 | 0.8488054 |
| A0A8I6G5T6 | Pkm | 0.0167593 | 0.8488054 | Q5U1X1 | Rexo2 | 0.0014396 | 0.7064639 |
| A0A8I6G7L6 | Nfix | 0.0085642 | 0.8488054 | Q5U204 | Lamtor3 | 0.0459764 | 0.8488054 |
| A0A8I6G9C9 | Srl | 0.0411655 | 0.8488054 | Q5U317 | Fip1l1 | 0.0336618 | 0.8488054 |
| A0A8I6GDH0 | ENSRNOG00000069405 | 0.0326487 | 0.8488054 | Q5U3Z7 | Shmt2 | 0.0372172 | 0.8488054 |
| A0A8I6GJ67 | Pin1 | 0.0221515 | 0.8488054 | Q5XHY7 | Stam2 | 0.0330113 | 0.8488054 |
| A0A8I6GJI9 | Fam185a | 0.0008982 | 0.6684691 | Q5XI68 | Dr1 | 0.0466872 | 0.8488054 |
| A0A8I6GLJ8 | ENSRNOG00000071091 | 5.14E-005 | 0.1574955 | Q5XID1 | Ciapin1 | 0.043642 | 0.8488054 |
| A0A8I6GMR3 | Anapc7 | 0.0375175 | 0.8488054 | Q5XIM9 | Cct2 | 0.0263404 | 0.8488054 |
| A0A8J8XF56 | Dazap1 | 0.0064845 | 0.8488054 | Q5XIU5 | Psmf1 | 0.000385 | 0.5903548 |
| A0A8J8XVH8 | ENSRNOG00000068221 | 0.0067211 | 0.8488054 | Q62625 | Map1lc3b | 0.0187687 | 0.8488054 |
| A0A8J8YNR6 | Fxr2 | 0.00109 | 0.6684691 | Q62780 | Ddx46 | 0.0065714 | 0.8488054 |
| A6HVR9 | Rpl34 | 0.0245975 | 0.8488054 | Q63041 | A1m | 0.0063123 | 0.8488054 |
| A6IPG1 | Eef1b2 | 0.0133266 | 0.8488054 | Q63203 | Fcgr2 | 0.0010012 | 0.6684691 |
| A6IXU7 | Tubb6 | 0.026535 | 0.8488054 | Q63312 | Phldb1 | 0.0088236 | 0.8488054 |
| A6J603 | Cd5l | 0.011962 | 0.8488054 | Q63357 | Myo1d | 0.0483986 | 0.8488054 |
| A6J9C6 | Blvrb | 0.0453443 | 0.8488054 | Q63396 | Sub1 | 0.0292521 | 0.8488054 |
| B0BNJ9 | Tmem14c | 0.0407632 | 0.8488054 | Q63560 | Map6 | 0.0335694 | 0.8488054 |
| B0LT89 | Stk24 | 0.0069263 | 0.8488054 | Q63616 | Vps33b | 0.0206716 | 0.8488054 |
| B2GUZ3 | Mthfd1l | 0.046899 | 0.8488054 | Q63632 | Slc12a4 | 0.0191736 | 0.8488054 |
| B4F777 | Hmgn5 | 0.0456027 | 0.8488054 | Q64380 | Sardh | 0.0355879 | 0.8488054 |
| B4F7C7 | Hebp1 | 0.0029484 | 0.7679417 | Q66HA8 | Hsph1 | 0.0204207 | 0.8488054 |
| C6JUQ2 | Gab1 | 0.0446791 | 0.8488054 | Q66HR2 | Mapre1 | 0.0466941 | 0.8488054 |
| D3Z8E6 | Camsap1 | 0.0121926 | 0.8488054 | Q68FQ0 | Cct5 | 0.0403739 | 0.8488054 |
| D3ZBP3 | Exosc2 | 0.0179695 | 0.8488054 | Q68FV2 | Arl6ip6 | 0.0029587 | 0.7679417 |
| D3ZBT9 | Ppp6r3 | 0.0147776 | 0.8488054 | Q6AXT0 | Mrpl37 | 0.0060041 | 0.8488054 |
| D3ZG07 | Lsm6 | 0.0158095 | 0.8488054 | Q6AY23 | Pycr2 | 0.016053 | 0.8488054 |
| D3ZJ96 | Usp28 | 0.0110931 | 0.8488054 | Q6AY41 | Tmem30a | 0.013564 | 0.8488054 |
| D3ZKE1 | Uvrag | 0.0344493 | 0.8488054 | Q6AY71 | Cfap418 | 0.0444739 | 0.8488054 |
| D3ZML3 | Cdk11b | 0.0273713 | 0.8488054 | Q6AYF6 | Glt8d1 | 0.0155115 | 0.8488054 |
| D3ZNF4 | Tbl1xr1 | 0.0321259 | 0.8488054 | Q6AYT7 | Abhd12 | 0.0433578 | 0.8488054 |
| D3ZPW7 | Gpx8 | 0.0471339 | 0.8488054 | Q6AZ50 | Atg3 | 0.0443366 | 0.8488054 |

| | | | | | | | |
|---|---|---|---|---|---|---|---|
| D3ZQG6 | Trim2 | 0.0100831 | 0.8488054 | Q6DGG0 | Ppid | 0.0384327 | 0.8488054 |
| D3ZQR7 | Plod2 | 0.0294405 | 0.8488054 | Q6IN22 | Ctsb | 0.0200606 | 0.8488054 |
| D3ZU54 | Denr | 0.0309088 | 0.8488054 | Q6IRE3 | Gimap4 | 0.012846 | 0.8488054 |
| D3ZUY0 | Rdh14 | 0.0377743 | 0.8488054 | Q6IUR5 | Nenf | 0.002628 | 0.7679417 |
| D3ZV82 | Gbp7 | 0.0089535 | 0.8488054 | Q6MGC4 | Pfdn6 | 0.0238934 | 0.8488054 |
| D3ZW38 | Exosc6 | 0.047077 | 0.8488054 | Q6P686 | Ostf1 | 0.0360678 | 0.8488054 |
| D3ZYE2 | Igkvl15 | 0.0414518 | 0.8488054 | Q6P720 | Arhgef25 | 0.0473079 | 0.8488054 |
| D3ZYK9 | Npm3 | 0.034052 | 0.8488054 | Q6P7C7 | Gpnmb | 0.0282117 | 0.8488054 |
| D4A183 | Vnn3 | 0.0117603 | 0.8488054 | Q6XVN8 | Map1lc3a | 0.0496343 | 0.8516885 |
| D4A1J6 | Ankfy1 | 0.0084131 | 0.8488054 | Q7TP47 | Syncrip | 0.0378892 | 0.8488054 |
| D4A321 | Dhx38 | 0.0390415 | 0.8488054 | Q7TSA0 | Rhot2 | 0.0402064 | 0.8488054 |
| D4A3X3 | Isg15 | 0.0337603 | 0.8488054 | Q7TSE9 | Hax1 | 0.0361541 | 0.8488054 |
| D4A7I6 | Washc4 | 0.0175246 | 0.8488054 | Q811A2 | Bst2 | 0.0018576 | 0.7595054 |
| D4A997 | Htatsf1 | 0.0184773 | 0.8488054 | Q812D1 | Psip1 | 0.0217469 | 0.8488054 |
| D4AAU6 | Ccdc25 | 0.0065274 | 0.8488054 | Q8CG07 | Wrnip1 | 0.0126829 | 0.8488054 |
| D4ACA6 | Mideas | 0.038872 | 0.8488054 | Q8CG09 | Abcc1 | 0.0364447 | 0.8488054 |
| D4ADL7 | Xxylt1 | 0.0208899 | 0.8488054 | Q8CIY2 | Duox1 | 0.0254533 | 0.8488054 |
| E9PU10 | Ifi47 | 0.010775 | 0.8488054 | Q8R511 | Fnbp1 | 0.0121501 | 0.8488054 |
| E9PU28 | Impdh2 | 0.0091028 | 0.8488054 | Q8R5G4 | Cdkn2c | 0.0155741 | 0.8488054 |
| F1LPS6 | Ifit1 | 0.0109213 | 0.8488054 | Q91Y81 | Septin2 | 0.031288 | 0.8488054 |
| F1LRT9 | Dync1h1 | 0.0457223 | 0.8488054 | Q925Q9 | Sh3kbp1 | 0.0438755 | 0.8488054 |
| F1LTN6 | AABR07060872.1 | 0.0467388 | 0.8488054 | Q99P77 | Gtpbp4 | 0.0338554 | 0.8488054 |
| F1LUD9 | Lrrc20 | 0.0457018 | 0.8488054 | Q9JJ54 | Hnrnpd | 0.0203622 | 0.8488054 |
| F1M0C4 | Kctd14 | 0.016515 | 0.8488054 | Q9JLU4 | Shank3 | 0.0226148 | 0.8488054 |
| F1MA98 | Tpr | 0.0191185 | 0.8488054 | Q9QUL6 | Nsf | 0.0360327 | 0.8488054 |
| F7F7A6 | Eif4a1 | 0.0441255 | 0.8488054 | Q9QVC8 | Fkbp4 | 0.0459452 | 0.8488054 |
| F7FBG3 | Commd7 | 0.0343845 | 0.8488054 | Q9QXJ0 | Ttl | 0.0299431 | 0.8488054 |
| F7FE18 | Arsl | 0.039611 | 0.8488054 | Q9R050 | Ssbp3 | 0.0475397 | 0.8488054 |
| F7FGV7 | Psmd6 | 0.0010714 | 0.6684691 | Q9R1T3 | Ctsz | 0.0474207 | 0.8488054 |
| G3V6F1 | Mybph | 0.0220154 | 0.8488054 | Q9WUS0 | Ak4 | 0.0252237 | 0.8488054 |
| G3V6G1 | Jchain | 0.0080088 | 0.8488054 | Q9WUW3 | Cfi | 0.0489827 | 0.8510227 |
| G3V763 | Col5a1 | 0.0029671 | 0.7679417 | Q9WV78 | Plvap | 0.0206486 | 0.8488054 |
| G3V7P1 | Stx12 | 0.0412759 | 0.8488054 | Q9WVT0 | Adgrf5 | 0.0269123 | 0.8488054 |
| G3V828 | Cnpy3 | 0.0139004 | 0.8488054 | Q9Z1K9 | Adam17 | 0.0154983 | 0.8488054 |

| | | | | | | | |
|---|---|---|---|---|---|---|---|
| G3V8U9 | Psmb4 | 0.0150474 | 0.8488054 | Q9Z1S7 | Omd | 0.0344481 | 0.8488054 |
| M0R4Z8 | Gatd1 | 0.0331846 | 0.8488054 | Q9Z2L9 | Ndrg4 | 0.014584 | 0.8488054 |
| M0R637 | Mettl1 | 0.0402692 | 0.8488054 | R4I3Z7 | Ncf2 | 0.0469047 | 0.8488054 |
| M0RCX8 | Srm | 0.0238079 | 0.8488054 | Z4YP11 | Tgm4 | 0.0154466 | 0.8488054 |

**Table S10.** Differentially expressed genes

| all_ID | Model vs Control | SRGBHSP_H vs Model | gene_name | chr | start | end | strand | gene_biotype | TF_Family |
|---|---|---|---|---|---|---|---|---|---|
| ENSRNOG00000019050 | TRUE | TRUE | Ifit1 | 1 | 2.32E+08 | 2.32E+08 | + | protein_coding | NA |
| ENSRNOG00000022910 | TRUE | TRUE | Emcn | 2 | 2.26E+08 | 2.26E+08 | + | protein_coding | NA |
| ENSRNOG00000031743 | TRUE | TRUE | Gbp2 | 2 | 2.31E+08 | 2.31E+08 | + | protein_coding | NA |
| ENSRNOG00000016538 | TRUE | TRUE | Itga8 | 17 | 75304008 | 75501510 | - | protein_coding | NA |
| ENSRNOG00000003209 | TRUE | TRUE | Pcp4l1 | 13 | 83588992 | 83609966 | - | protein_coding | NA |
| ENSRNOG00000015812 | TRUE | TRUE | Tm4sf1 | 2 | 1.41E+08 | 1.41E+08 | - | protein_coding | NA |
| ENSRNOG00000008369 | TRUE | TRUE | Gimap4 | 4 | 77636401 | 77643306 | + | protein_coding | NA |
| ENSRNOG00000005700 | TRUE | TRUE | Nsg1 | 14 | 72648741 | 72670514 | + | protein_coding | NA |
| ENSRNOG00000056714 | TRUE | TRUE | Sla | 7 | 98535368 | 98584648 | - | protein_coding | NA |
| ENSRNOG00000065852 | TRUE | FALSE | ENSRNOG00000065852 | 8 | 35750482 | 35754071 | - | protein_coding | NA |
| ENSRNOG00000009086 | TRUE | TRUE | Apcs | 13 | 85373220 | 85374298 | - | protein_coding | NA |
| ENSRNOG00000019417 | TRUE | TRUE | Hsd3b5 | 2 | 1.86E+08 | 1.86E+08 | - | protein_coding | NA |
| ENSRNOG00000052604 | TRUE | FALSE | ENSRNOG00000052604 | 13 | 51262633 | 51296723 | - | protein_coding | NA |
| ENSRNOG00000067685 | TRUE | TRUE | Mug1 | 4 | 1.55E+08 | 1.55E+08 | + | protein_coding | NA |
| ENSRNOG00000058664 | TRUE | FALSE | Usp9y | Y | 1156135 | 1300943 | - | protein_coding | NA |
| ENSRNOG00000007639 | TRUE | TRUE | C8b | 5 | 1.2E+08 | 1.2E+08 | + | protein_coding | NA |
| ENSRNOG00000008638 | TRUE | TRUE | Angptl3 | 5 | 1.14E+08 | 1.14E+08 | + | protein_coding | NA |
| ENSRNOG00000005620 | TRUE | TRUE | Lcp2 | 10 | 18642658 | 18689559 | + | protein_coding | NA |
| ENSRNOG00000030538 | TRUE | TRUE | Slco1b2 | 4 | 1.75E+08 | 1.75E+08 | + | protein_coding | NA |
| ENSRNOG00000067532 | TRUE | TRUE | Cyp3a23-3a1 | 12 | 9254475 | 9285030 | - | protein_coding | NA |
| ENSRNOG00000030869 | TRUE | FALSE | Aldoart2 | 6 | 72939788 | 72941709 | + | protein_coding | NA |
| ENSRNOG00000051912 | TRUE | TRUE | Acnat2 | 5 | 63753270 | 63757521 | - | protein_coding | NA |
| ENSRNOG00000000969 | TRUE | TRUE | Cyp3a18 | 12 | 8880528 | 8930381 | + | protein_coding | NA |
| ENSRNOG00000043131 | TRUE | FALSE | ENSRNOG00000043131 | 8 | 34672534 | 34676388 | - | protein_coding | NA |

| | | | | | | | | | |
|---|---|---|---|---|---|---|---|---|---|
| ENSRNOG00000012613 | TRUE | TRUE | F13b | 13 | 51130920 | 51156381 | + | protein_coding | NA |
| ENSRNOG00000020817 | TRUE | TRUE | Cyp2a1 | 1 | 82231611 | 82244887 | + | protein_coding | NA |
| ENSRNOG00000043404 | TRUE | TRUE | Uroc1 | 4 | 1.23E+08 | 1.23E+08 | + | protein_coding | NA |
| ENSRNOG00000070559 | TRUE | TRUE | ENSRNOG00000070559 | 11 | 78039376 | 78049353 | - | protein_coding | NA |
| ENSRNOG00000021405 | TRUE | TRUE | Cyp2c7 | 1 | 2.37E+08 | 2.37E+08 | + | protein_coding | NA |
| ENSRNOG00000024016 | TRUE | TRUE | Cyp2c6v1 | 1 | 2.38E+08 | 2.38E+08 | + | protein_coding | NA |
| ENSRNOG00000009788 | TRUE | TRUE | Serpina4 | 6 | 1.23E+08 | 1.23E+08 | + | protein_coding | NA |
| ENSRNOG00000015406 | TRUE | TRUE | Pgm5 | 1 | 2.22E+08 | 2.23E+08 | - | protein_coding | NA |
| ENSRNOG00000020993 | TRUE | TRUE | Ms4a2 | 1 | 2.08E+08 | 2.08E+08 | - | protein_coding | NA |
| ENSRNOG00000029861 | TRUE | FALSE | Gsta2 | 8 | 79184322 | 79196798 | - | protein_coding | NA |
| ENSRNOG00000034038 | TRUE | TRUE | AABR07005844.1 | 1 | 1.86E+08 | 1.86E+08 | + | protein_coding | NA |
| ENSRNOG00000047986 | TRUE | TRUE | Sult2a2 | 1 | 74911100 | 75508134 | - | protein_coding | NA |
| ENSRNOG00000001980 | TRUE | FALSE | Ugt2b35 | 14 | 20853587 | 20891037 | + | protein_coding | NA |
| ENSRNOG00000064682 | TRUE | TRUE | ENSRNOG00000064682 | 13 | 82486637 | 82523851 | + | lncRNA | NA |
| ENSRNOG00000008895 | TRUE | TRUE | Hnf4a | 3 | 1.52E+08 | 1.52E+08 | + | protein_coding | RXR-like |
| ENSRNOG00000033164 | TRUE | FALSE | Cyp2b3 | 1 | 81652787 | 81732143 | + | protein_coding | NA |
| ENSRNOG00000032560 | TRUE | FALSE | Cyp3a23-3a1 | 12 | 9015383 | 9285008 | - | protein_coding | NA |
| ENSRNOG00000015773 | TRUE | TRUE | Il21r | 1 | 1.8E+08 | 1.8E+08 | + | protein_coding | NA |
| ENSRNOG00000054764 | TRUE | TRUE | Flt3 | 12 | 7623930 | 7699474 | + | protein_coding | NA |
| ENSRNOG00000009037 | TRUE | TRUE | Sulf1 | 5 | 6362911 | 6525584 | - | protein_coding | NA |
| ENSRNOG00000036711 | TRUE | TRUE | Spn | 1 | 1.82E+08 | 1.82E+08 | - | protein_coding | NA |
| ENSRNOG00000039593 | TRUE | TRUE | Ecscr | 18 | 27309718 | 27319032 | - | protein_coding | NA |
| ENSRNOG00000070397 | TRUE | TRUE | C4b | 20 | 4197366 | 4211681 | + | protein_coding | NA |
| ENSRNOG00000009342 | TRUE | TRUE | Fcnb | 3 | 11393739 | 11402151 | - | protein_coding | NA |
| ENSRNOG00000047446 | TRUE | TRUE | Foxc2 | 19 | 49185662 | 49188737 | + | protein_coding | Fork_head |
| ENSRNOG00000032240 | TRUE | FALSE | Gbp5 | 2 | 2.31E+08 | 2.31E+08 | + | protein_coding | NA |
| ENSRNOG00000049464 | TRUE | TRUE | Cyp2c13 | 1 | 2.39E+08 | 2.39E+08 | - | protein_coding | NA |
| ENSRNOG00000017980 | TRUE | TRUE | Itgal | 1 | 1.82E+08 | 1.82E+08 | + | protein_coding | NA |
| ENSRNOG00000062101 | TRUE | TRUE | Ace | 10 | 90910316 | 90931131 | + | protein_coding | NA |
| ENSRNOG00000022242 | TRUE | FALSE | Cxcl9 | 14 | 15722908 | 15728435 | + | protein_coding | NA |
| ENSRNOG00000018692 | TRUE | TRUE | Mc4r | 18 | 60419832 | 60421719 | - | protein_coding | NA |
| ENSRNOG00000004273 | TRUE | TRUE | Ifitm1 | 1 | 1.96E+08 | 1.96E+08 | + | protein_coding | NA |
| ENSRNOG00000062480 | TRUE | FALSE | ENSRNOG00000062480 | 1 | 69429269 | 69447438 | + | protein_coding | NA |
| ENSRNOG00000011557 | TRUE | FALSE | S100a8 | 2 | 1.76E+08 | 1.76E+08 | + | protein_coding | NA |

| | | | | | | | | | |
|---|---|---|---|---|---|---|---|---|---|
| ENSRNOG00000001544 | TRUE | TRUE | Cyyr1 | 11 | 24515316 | 24663961 | - | protein_coding | NA |
| ENSRNOG00000060687 | TRUE | TRUE | Slc24a3 | 3 | 1.33E+08 | 1.33E+08 | + | protein_coding | NA |
| ENSRNOG00000016491 | TRUE | TRUE | Fam110d | 5 | 1.46E+08 | 1.46E+08 | - | protein_coding | NA |
| ENSRNOG00000071002 | TRUE | TRUE | Kcne4 | 9 | 80247379 | 80250979 | + | protein_coding | NA |
| ENSRNOG00000043486 | TRUE | TRUE | Tnfrsf26 | 1 | 1.99E+08 | 1.99E+08 | - | protein_coding | NA |
| ENSRNOG00000006167 | TRUE | TRUE | Rasal3 | 7 | 11396090 | 11411465 | - | protein_coding | NA |
| ENSRNOG00000017020 | TRUE | TRUE | Inpp5d | 9 | 88287677 | 88392746 | + | protein_coding | NA |
| ENSRNOG00000033261 | TRUE | TRUE | Fam107a | 15 | 16607205 | 16630396 | + | protein_coding | NA |
| ENSRNOG00000002659 | TRUE | TRUE | Ciita | 10 | 5140178 | 5187440 | - | protein_coding | NA |
| ENSRNOG00000054251 | TRUE | TRUE | Clec7a | 4 | 1.63E+08 | 1.63E+08 | - | protein_coding | NA |
| ENSRNOG00000010262 | TRUE | TRUE | Hdc | 3 | 1.14E+08 | 1.14E+08 | - | protein_coding | NA |
| ENSRNOG00000053661 | TRUE | FALSE | Klra17 | 4 | 1.63E+08 | 1.63E+08 | - | protein_coding | NA |
| ENSRNOG00000000187 | TRUE | TRUE | Csf2rb | 7 | 1.1E+08 | 1.1E+08 | + | protein_coding | NA |
| ENSRNOG00000059857 | TRUE | TRUE | Rnd1 | 7 | 1.3E+08 | 1.3E+08 | - | protein_coding | NA |
| ENSRNOG00000022523 | TRUE | TRUE | Fkbp5 | 20 | 6457216 | 6541674 | - | protein_coding | NA |
| ENSRNOG00000042771 | TRUE | TRUE | Apol3 | 7 | 1.09E+08 | 1.09E+08 | + | protein_coding | NA |
| ENSRNOG00000010233 | TRUE | TRUE | Cald1 | 4 | 63265942 | 63446932 | + | protein_coding | NA |
| ENSRNOG00000068701 | TRUE | TRUE | ENSRNOG00000068701 | 14 | 4224146 | 4226886 | + | lncRNA | NA |
| ENSRNOG00000001821 | TRUE | TRUE | Adipoq | 11 | 77721912 | 77735564 | - | protein_coding | NA |
| ENSRNOG00000002776 | TRUE | TRUE | Sell | 13 | 76416915 | 76436456 | + | protein_coding | NA |
| ENSRNOG00000064497 | TRUE | TRUE | Olr1585 | 13 | 86009008 | 86073347 | + | protein_coding | NA |
| ENSRNOG00000004972 | TRUE | TRUE | Upp1 | 14 | 83809960 | 83829462 | + | protein_coding | NA |
| ENSRNOG00000049334 | TRUE | TRUE | Atp2c2 | 19 | 47754120 | 47811368 | + | protein_coding | NA |
| ENSRNOG00000020169 | TRUE | TRUE | Gimap8 | 4 | 77600186 | 77611590 | + | protein_coding | NA |
| ENSRNOG00000066264 | TRUE | TRUE | RGD1563400 | 12 | 18566837 | 18582202 | - | protein_coding | NA |
| ENSRNOG00000019662 | TRUE | TRUE | Tm6sf1 | 1 | 1.36E+08 | 1.36E+08 | + | protein_coding | NA |
| ENSRNOG00000023257 | TRUE | TRUE | Adamts9 | 4 | 1.25E+08 | 1.25E+08 | - | protein_coding | NA |
| ENSRNOG00000045998 | TRUE | TRUE | Sema6b | 9 | 950939 | 961521 | - | protein_coding | NA |
| ENSRNOG00000009343 | TRUE | TRUE | Evpl | 10 | 1.01E+08 | 1.01E+08 | - | protein_coding | NA |
| ENSRNOG00000022697 | TRUE | TRUE | Clec14a | 6 | 75881473 | 75884612 | - | protein_coding | NA |
| ENSRNOG00000007350 | TRUE | TRUE | Rac2 | 7 | 1.1E+08 | 1.1E+08 | - | protein_coding | NA |
| ENSRNOG00000016069 | TRUE | FALSE | Cd3e | 8 | 45303852 | 45315022 | - | protein_coding | NA |
| ENSRNOG00000020803 | TRUE | TRUE | Meox1 | 10 | 86818478 | 86837660 | - | protein_coding | Homeobox |
| ENSRNOG00000047414 | TRUE | TRUE | AABR07058479.1 | 7 | 1.09E+08 | 1.09E+08 | - | protein_coding | NA |

| | | | | | | | | | |
|---|---|---|---|---|---|---|---|---|---|
| ENSRNOG00000005530 | TRUE | FALSE | Rps6kl1 | 6 | 1.05E+08 | 1.05E+08 | - | protein_coding | NA |
| ENSRNOG00000002141 | TRUE | TRUE | Cd200 | 11 | 55410196 | 55501648 | + | protein_coding | NA |
| ENSRNOG00000002820 | TRUE | FALSE | ENSRNOG00000002820 | 10 | 42615555 | 42623784 | + | protein_coding | NA |
| ENSRNOG00000008005 | TRUE | TRUE | Akna | 5 | 76777415 | 76816017 | - | protein_coding | Others |
| ENSRNOG00000046254 | TRUE | TRUE | Adgre1 | 9 | 2242474 | 2398000 | + | protein_coding | NA |
| ENSRNOG00000057058 | TRUE | TRUE | Cd300a | 10 | 99929801 | 99942674 | + | protein_coding | NA |
| ENSRNOG00000019388 | TRUE | TRUE | Egfl7 | 3 | 9407520 | 9416879 | + | protein_coding | NA |
| ENSRNOG00000003954 | TRUE | TRUE | Il2rg | X | 66392542 | 66399823 | - | protein_coding | NA |
| ENSRNOG00000014333 | TRUE | TRUE | Vcam1 | 2 | 2.04E+08 | 2.04E+08 | - | protein_coding | NA |
| ENSRNOG00000012495 | TRUE | TRUE | Podxl | 4 | 60135109 | 60181899 | - | protein_coding | NA |
| ENSRNOG00000005853 | TRUE | TRUE | Tacr1 | 4 | 1.15E+08 | 1.15E+08 | + | protein_coding | NA |
| ENSRNOG00000025143 | TRUE | TRUE | Icam2 | 10 | 91308538 | 91315293 | - | protein_coding | NA |
| ENSRNOG00000010832 | TRUE | TRUE | Pdgfrl | 16 | 51347948 | 51407850 | - | protein_coding | NA |
| ENSRNOG00000011774 | TRUE | TRUE | Fblim1 | 5 | 1.54E+08 | 1.54E+08 | - | protein_coding | NA |
| ENSRNOG00000051977 | TRUE | TRUE | Mmrn2 | 16 | 9705892 | 9727405 | + | protein_coding | NA |
| ENSRNOG00000058039 | TRUE | TRUE | Acta2 | 1 | 2.32E+08 | 2.32E+08 | - | protein_coding | NA |
| ENSRNOG00000008045 | TRUE | TRUE | Slamf9 | 13 | 84941982 | 84944748 | + | protein_coding | NA |
| ENSRNOG00000023209 | TRUE | TRUE | Slamf7 | 13 | 84136899 | 84154105 | - | protein_coding | NA |
| ENSRNOG00000024382 | TRUE | TRUE | Fcgr3a | 13 | 83249872 | 83259921 | + | protein_coding | NA |
| ENSRNOG00000004200 | TRUE | TRUE | Sybu | 7 | 75847003 | 75948828 | - | protein_coding | NA |
| ENSRNOG00000008416 | TRUE | TRUE | Gimap5 | 4 | 77687564 | 77703086 | + | protein_coding | NA |
| ENSRNOG00000061739 | TRUE | TRUE | Klrk1 | 4 | 1.63E+08 | 1.63E+08 | - | protein_coding | NA |
| ENSRNOG00000003094 | TRUE | TRUE | Cmahp | 17 | 40583667 | 40642275 | - | protein_coding | NA |
| ENSRNOG00000033338 | TRUE | TRUE | Gimap6 | 4 | 77652610 | 77659116 | - | protein_coding | NA |
| ENSRNOG00000001752 | TRUE | TRUE | Nrros | 11 | 68628712 | 68646142 | + | protein_coding | NA |
| ENSRNOG00000008592 | TRUE | TRUE | Barx2 | 8 | 30251132 | 30319013 | - | protein_coding | Homeobox |
| ENSRNOG00000011913 | TRUE | TRUE | Cp | 2 | 1.02E+08 | 1.02E+08 | + | protein_coding | NA |
| ENSRNOG00000060460 | TRUE | TRUE | Clec1a | 4 | 1.63E+08 | 1.63E+08 | - | protein_coding | NA |
| ENSRNOG00000010744 | TRUE | TRUE | Nrp1 | 19 | 56359455 | 56513633 | + | protein_coding | NA |
| ENSRNOG00000025476 | TRUE | TRUE | Tmem252 | 1 | 2.22E+08 | 2.22E+08 | + | protein_coding | NA |
| ENSRNOG00000031168 | TRUE | TRUE | Arhgap15 | 3 | 27989633 | 28600265 | + | protein_coding | NA |
| ENSRNOG00000002907 | TRUE | TRUE | Plekha6 | 13 | 44615011 | 44754673 | + | protein_coding | NA |
| ENSRNOG00000026702 | TRUE | TRUE | Jaml | 8 | 45383495 | 45416565 | + | protein_coding | NA |
| ENSRNOG00000002229 | TRUE | TRUE | Adcy5 | 11 | 65471612 | 65618974 | - | protein_coding | NA |

| | | | | | | | | | |
|---|---|---|---|---|---|---|---|---|---|
| ENSRNOG00000020173 | TRUE | TRUE | Tie1 | 5 | 1.32E+08 | 1.32E+08 | - | protein_coding | NA |
| ENSRNOG00000059961 | TRUE | TRUE | Rapgef3 | 7 | 1.29E+08 | 1.29E+08 | - | protein_coding | NA |
| ENSRNOG00000013776 | TRUE | FALSE | Semg1 | 3 | 1.53E+08 | 1.53E+08 | - | protein_coding | NA |
| ENSRNOG00000029662 | TRUE | TRUE | Wdfy4 | 16 | 8221719 | 8450491 | - | protein_coding | NA |
| ENSRNOG00000069485 | TRUE | TRUE | ENSRNOG00000069485 | 20 | 6549166 | 6575490 | - | lncRNA | NA |
| ENSRNOG00000008862 | TRUE | TRUE | Abcg4 | 8 | 44611187 | 44626881 | - | protein_coding | NA |
| ENSRNOG00000037199 | TRUE | TRUE | Spink8 | 8 | 1.1E+08 | 1.1E+08 | + | protein_coding | NA |
| ENSRNOG00000003170 | TRUE | TRUE | Nlrp3 | 10 | 44328566 | 44352811 | + | protein_coding | NA |
| ENSRNOG00000019043 | TRUE | FALSE | Pdcd1 | 9 | 94418791 | 94431937 | - | protein_coding | NA |
| ENSRNOG00000025051 | TRUE | TRUE | Tal1 | 5 | 1.29E+08 | 1.29E+08 | + | protein_coding | bHLH |
| ENSRNOG00000005964 | TRUE | TRUE | Nr4a3 | 5 | 62361822 | 62402733 | + | protein_coding | NGFIB-like |
| ENSRNOG00000053272 | TRUE | TRUE | Chi3l1 | 13 | 45641802 | 45649787 | + | protein_coding | NA |
| ENSRNOG00000001652 | TRUE | TRUE | Erg | 11 | 34678618 | 34845871 | - | protein_coding | ETS |
| ENSRNOG00000007584 | TRUE | TRUE | Ehd4 | 3 | 1.07E+08 | 1.07E+08 | - | protein_coding | NA |
| ENSRNOG00000020563 | TRUE | TRUE | Cma1 | 15 | 29417451 | 29420233 | - | protein_coding | NA |
| ENSRNOG00000017628 | TRUE | TRUE | Tagln | 8 | 46222472 | 46230668 | - | protein_coding | NA |
| ENSRNOG00000004009 | TRUE | TRUE | Xpnpep2 | X | 1.27E+08 | 1.27E+08 | + | protein_coding | NA |
| ENSRNOG00000043182 | TRUE | TRUE | Septin6 | X | 1.16E+08 | 1.16E+08 | - | protein_coding | NA |
| ENSRNOG00000009370 | TRUE | TRUE | Tbkbp1 | 10 | 82120564 | 82134352 | - | protein_coding | NA |
| ENSRNOG00000016640 | TRUE | TRUE | Dner | 9 | 85586987 | 85902637 | - | protein_coding | NA |
| ENSRNOG00000008759 | TRUE | FALSE | Csf3r | 5 | 1.38E+08 | 1.38E+08 | + | protein_coding | NA |
| ENSRNOG00000016575 | TRUE | TRUE | Tnfrsf1b | 5 | 1.57E+08 | 1.57E+08 | - | protein_coding | NA |
| ENSRNOG00000029401 | TRUE | TRUE | Actg2 | 4 | 1.16E+08 | 1.16E+08 | - | protein_coding | NA |
| ENSRNOG00000032844 | TRUE | TRUE | RT1-Da | 20 | 4512911 | 4518455 | + | protein_coding | NA |
| ENSRNOG00000013683 | TRUE | TRUE | S1pr1 | 2 | 2.04E+08 | 2.04E+08 | - | protein_coding | NA |
| ENSRNOG00000066008 | TRUE | TRUE | Pecam1 | 10 | 91590521 | 91652116 | - | protein_coding | NA |
| ENSRNOG00000009907 | TRUE | FALSE | Mmp8 | 8 | 4724029 | 4733520 | + | protein_coding | NA |
| ENSRNOG00000046143 | TRUE | TRUE | Pgap3 | 10 | 83387113 | 83398628 | - | protein_coding | NA |
| ENSRNOG00000005787 | TRUE | FALSE | Erp27 | 4 | 1.7E+08 | 1.7E+08 | - | protein_coding | NA |
| ENSRNOG00000018735 | TRUE | TRUE | Cd74 | 18 | 54256778 | 54266003 | + | protein_coding | NA |
| ENSRNOG00000007062 | TRUE | TRUE | Rin3 | 6 | 1.21E+08 | 1.22E+08 | + | protein_coding | NA |
| ENSRNOG00000008134 | TRUE | TRUE | Mefv | 10 | 11787422 | 11796973 | + | protein_coding | NA |
| ENSRNOG00000020546 | TRUE | TRUE | Lipe | 1 | 80965627 | 80984310 | - | protein_coding | NA |

| | | | | | | | | | |
|---|---|---|---|---|---|---|---|---|---|
| ENSRNOG00000022110 | TRUE | TRUE | Gcsam | 11 | 55196712 | 55206992 | - | protein_coding | NA |
| ENSRNOG00000001216 | TRUE | TRUE | Trpm2 | 20 | 10707014 | 10753181 | + | protein_coding | NA |
| ENSRNOG00000045560 | TRUE | TRUE | Gvin1 | 1 | 1.6E+08 | 1.6E+08 | - | protein_coding | NA |
| ENSRNOG00000014574 | TRUE | TRUE | Entpd1 | 1 | 2.39E+08 | 2.4E+08 | + | protein_coding | NA |
| ENSRNOG00000016818 | TRUE | TRUE | Fgfr3 | 14 | 76987993 | 77003341 | - | protein_coding | NA |
| ENSRNOG00000020246 | TRUE | TRUE | Myl9 | 3 | 1.45E+08 | 1.45E+08 | + | protein_coding | NA |
| ENSRNOG00000002161 | TRUE | TRUE | Tlr6 | 14 | 43362164 | 43375685 | + | protein_coding | NA |
| ENSRNOG00000051690 | TRUE | TRUE | Clec9a | 4 | 1.63E+08 | 1.63E+08 | + | protein_coding | NA |
| ENSRNOG00000008697 | TRUE | TRUE | Ccn3 | 7 | 86094000 | 86101019 | + | protein_coding | NA |
| ENSRNOG00000017684 | TRUE | TRUE | Fbxl22 | 8 | 67069998 | 67076685 | - | protein_coding | NA |
| ENSRNOG00000033940 | TRUE | TRUE | Adgrl4 | 2 | 2.4E+08 | 2.4E+08 | + | protein_coding | NA |
| ENSRNOG00000013886 | TRUE | TRUE | Fyb1 | 2 | 55632698 | 55779629 | + | protein_coding | NA |
| ENSRNOG00000017676 | TRUE | TRUE | Plvap | 16 | 18184975 | 18197301 | - | protein_coding | NA |
| ENSRNOG00000017767 | TRUE | TRUE | Irag1 | 1 | 1.65E+08 | 1.65E+08 | - | protein_coding | NA |
| ENSRNOG00000002215 | TRUE | TRUE | Mylk | 11 | 65783008 | 66030261 | - | protein_coding | NA |
| ENSRNOG00000019689 | TRUE | TRUE | Vwf | 4 | 1.58E+08 | 1.58E+08 | + | protein_coding | NA |
| ENSRNOG00000005679 | TRUE | TRUE | Fap | 3 | 47138813 | 47225545 | - | protein_coding | NA |
| ENSRNOG00000056219 | TRUE | FALSE | Olr1 | 4 | 1.63E+08 | 1.63E+08 | - | protein_coding | NA |
| ENSRNOG00000006620 | TRUE | TRUE | Xcr1 | 8 | 1.23E+08 | 1.23E+08 | - | protein_coding | NA |
| ENSRNOG00000049918 | TRUE | TRUE | Lrg1 | 9 | 947516 | 949813 | - | protein_coding | NA |
| ENSRNOG00000000441 | TRUE | TRUE | Gpsm3 | 20 | 4157123 | 4159035 | - | protein_coding | NA |
| ENSRNOG00000033256 | TRUE | FALSE | AB124611 | 8 | 20078639 | 20095696 | + | protein_coding | NA |
| ENSRNOG00000003136 | TRUE | TRUE | Fcrla | 13 | 83160398 | 83170762 | - | protein_coding | NA |
| ENSRNOG00000011181 | TRUE | TRUE | Cpa3 | 2 | 1.03E+08 | 1.03E+08 | - | protein_coding | NA |
| ENSRNOG00000001302 | TRUE | TRUE | Adora2a | 20 | 13315853 | 13333386 | - | protein_coding | NA |
| ENSRNOG00000020699 | TRUE | TRUE | Cd37 | 1 | 95719190 | 95724648 | - | protein_coding | NA |
| ENSRNOG00000048053 | TRUE | TRUE | AC128859.3 | 10 | 67953273 | 67983840 | + | protein_coding | NA |
| ENSRNOG00000029980 | TRUE | FALSE | Zbtb16 | 8 | 48994566 | 49177011 | - | protein_coding | ZBTB |
| ENSRNOG00000020884 | TRUE | TRUE | Cd6 | 1 | 2.07E+08 | 2.07E+08 | - | protein_coding | NA |
| ENSRNOG00000027730 | TRUE | TRUE | Nxpe1 | 8 | 48751014 | 48785353 | + | protein_coding | NA |
| ENSRNOG00000003486 | TRUE | TRUE | Mnda | 13 | 86017892 | 86034201 | - | protein_coding | NA |
| ENSRNOG00000009348 | TRUE | TRUE | Nos3 | 4 | 10793834 | 10814166 | - | protein_coding | NA |
| ENSRNOG00000059618 | TRUE | TRUE | Scarf1 | 10 | 60359197 | 60371516 | + | protein_coding | NA |
| ENSRNOG00000016945 | TRUE | TRUE | Pla2g2a | 5 | 1.51E+08 | 1.51E+08 | + | protein_coding | NA |

| | | | | | | | | | |
|---|---|---|---|---|---|---|---|---|---|
| ENSRNOG00000027736 | TRUE | TRUE | Cnn1 | 8 | 20632338 | 20641098 | + | protein_coding | NA |
| ENSRNOG00000030889 | TRUE | TRUE | Cldn22 | 16 | 44422345 | 44423354 | - | protein_coding | NA |
| ENSRNOG00000001915 | TRUE | TRUE | Chodl | 11 | 17654206 | 17676441 | + | protein_coding | NA |
| ENSRNOG00000004029 | TRUE | TRUE | Nectin4 | 13 | 83803184 | 83821709 | + | protein_coding | NA |
| ENSRNOG00000019330 | TRUE | TRUE | Procr | 3 | 1.44E+08 | 1.44E+08 | + | protein_coding | NA |
| ENSRNOG00000042445 | TRUE | TRUE | AC130970.1 | 10 | 1.01E+08 | 1.01E+08 | + | protein_coding | NA |
| ENSRNOG00000008904 | TRUE | TRUE | Fli1 | 8 | 30832753 | 30950433 | - | protein_coding | ETS |
| ENSRNOG00000054560 | TRUE | TRUE | Sh2d3c | 3 | 16010625 | 16046484 | + | protein_coding | NA |
| ENSRNOG00000003500 | TRUE | TRUE | Apoa2 | 13 | 83644470 | 83646355 | + | protein_coding | NA |
| ENSRNOG00000024569 | TRUE | TRUE | Gimap9 | 4 | 77626138 | 77629497 | + | protein_coding | NA |
| ENSRNOG00000003207 | TRUE | TRUE | Fmnl1 | 10 | 88116009 | 88143357 | + | protein_coding | NA |
| ENSRNOG00000033215 | TRUE | TRUE | RT1-Db1 | 20 | 4548666 | 4558258 | - | protein_coding | NA |
| ENSRNOG00000024181 | TRUE | TRUE | Tpsab1 | 10 | 14360396 | 14362811 | - | protein_coding | NA |
| ENSRNOG00000023493 | TRUE | TRUE | Creb3l4 | 2 | 1.76E+08 | 1.76E+08 | - | protein_coding | TF_bZIP |
| ENSRNOG00000009427 | TRUE | FALSE | Tbx21 | 10 | 82082322 | 82098831 | - | protein_coding | T-box |
| ENSRNOG00000005465 | TRUE | TRUE | Kcnmb1 | 10 | 18557904 | 18565798 | + | protein_coding | NA |
| ENSRNOG00000014149 | TRUE | TRUE | Npy1r | 16 | 23037789 | 23046759 | - | protein_coding | NA |
| ENSRNOG00000000394 | TRUE | TRUE | Srgn | 20 | 30442813 | 30457406 | - | protein_coding | NA |
| ENSRNOG00000003566 | TRUE | TRUE | Apoh | 10 | 93310977 | 93356329 | + | protein_coding | NA |
| ENSRNOG00000020401 | TRUE | TRUE | Adcy4 | 15 | 29266287 | 29282108 | - | protein_coding | NA |
| ENSRNOG00000071032 | TRUE | TRUE | Mmp25 | 10 | 12661208 | 12675871 | - | protein_coding | NA |
| ENSRNOG00000068084 | TRUE | TRUE | ENSRNOG00000068084 | 5 | 1.5E+08 | 1.5E+08 | + | lncRNA | NA |
| ENSRNOG00000015880 | TRUE | TRUE | Dpep1 | 19 | 51219660 | 51235257 | + | protein_coding | NA |
| ENSRNOG00000065499 | TRUE | TRUE | ENSRNOG00000065499 | 10 | 1.05E+08 | 1.05E+08 | - | lncRNA | NA |
| ENSRNOG00000018611 | TRUE | TRUE | Tpsb2 | 10 | 14382013 | 14383569 | + | protein_coding | NA |
| ENSRNOG00000016403 | TRUE | TRUE | Olah | 17 | 74877655 | 74902518 | + | protein_coding | NA |
| ENSRNOG00000010898 | TRUE | FALSE | Prok2 | 4 | 1.32E+08 | 1.32E+08 | - | protein_coding | NA |
| ENSRNOG00000011483 | TRUE | FALSE | S100a9 | 2 | 1.76E+08 | 1.76E+08 | - | protein_coding | NA |
| ENSRNOG00000010213 | TRUE | TRUE | Fgd5 | 4 | 1.24E+08 | 1.25E+08 | + | protein_coding | NA |
| ENSRNOG00000019965 | TRUE | TRUE | Tgfb1i1 | 1 | 1.83E+08 | 1.83E+08 | + | protein_coding | NA |
| ENSRNOG00000070606 | TRUE | FALSE | ENSRNOG00000070606 | 4 | 70001317 | 70014600 | + | protein_coding | NA |
| ENSRNOG00000019718 | TRUE | TRUE | Galnt15 | 16 | 7227271 | 7267014 | + | protein_coding | NA |
| ENSRNOG00000004737 | TRUE | TRUE | Cd48 | 13 | 84185532 | 84209142 | + | protein_coding | NA |
| ENSRNOG00000001333 | TRUE | TRUE | Azgp1 | 12 | 16931024 | 16939091 | + | protein_coding | NA |

| | | | | | | | | | |
|---|---|---|---|---|---|---|---|---|---|
| ENSRNOG00000057880 | TRUE | TRUE | Myh11 | 10 | 743685 | 838459 | + | protein_coding | NA |
| ENSRNOG00000010661 | TRUE | TRUE | Gzmk | 2 | 44840811 | 44848894 | - | protein_coding | NA |
| ENSRNOG00000015259 | TRUE | TRUE | Pth2r | 9 | 66706050 | 66810036 | + | protein_coding | NA |
| ENSRNOG00000002794 | TRUE | FALSE | Selp | 13 | 76476295 | 76511845 | + | protein_coding | NA |
| ENSRNOG00000015913 | TRUE | TRUE | Tspan5 | 2 | 2.27E+08 | 2.27E+08 | + | protein_coding | NA |
| ENSRNOG00000018336 | TRUE | TRUE | Eps8l2 | 1 | 1.96E+08 | 1.96E+08 | + | protein_coding | NA |
| ENSRNOG00000008054 | TRUE | TRUE | Igsf9 | 13 | 84948619 | 84965607 | + | protein_coding | NA |
| ENSRNOG00000036865 | TRUE | FALSE | Krt77 | 7 | 1.33E+08 | 1.33E+08 | - | protein_coding | NA |
| ENSRNOG00000015310 | TRUE | TRUE | Lrrc32 | 1 | 1.53E+08 | 1.53E+08 | + | protein_coding | NA |
| ENSRNOG00000016362 | TRUE | TRUE | Gpr4 | 1 | 78865409 | 78876599 | + | protein_coding | NA |
| ENSRNOG00000031706 | TRUE | FALSE | RGD1563601 | 17 | 27101431 | 27102809 | - | protein_coding | NA |
| ENSRNOG00000008988 | TRUE | TRUE | Cyp2d2 | 7 | 1.14E+08 | 1.14E+08 | - | protein_coding | NA |
| ENSRNOG00000069032 | TRUE | TRUE | ENSRNOG00000069032 | 7 | 1.09E+08 | 1.09E+08 | - | protein_coding | NA |
| ENSRNOG00000004249 | TRUE | TRUE | Tlr7 | X | 27027425 | 27054754 | + | protein_coding | NA |
| ENSRNOG00000058586 | TRUE | TRUE | Tyro3 | 3 | 1.07E+08 | 1.07E+08 | + | protein_coding | NA |
| ENSRNOG00000065590 | TRUE | TRUE | Gpr65 | 6 | 1.18E+08 | 1.18E+08 | + | protein_coding | NA |
| ENSRNOG00000019854 | TRUE | TRUE | Napsa | 1 | 95064900 | 95077094 | + | protein_coding | NA |
| ENSRNOG00000023485 | TRUE | FALSE | Ushbp1 | 16 | 18057326 | 18067877 | - | protein_coding | NA |
| ENSRNOG00000004649 | TRUE | TRUE | Il1b | 3 | 1.17E+08 | 1.17E+08 | - | protein_coding | NA |
| ENSRNOG00000015318 | TRUE | TRUE | Heyl | 5 | 1.36E+08 | 1.36E+08 | + | protein_coding | bHLH |
| ENSRNOG00000026762 | TRUE | TRUE | RT1-DOa | 20 | 4753598 | 4756577 | - | protein_coding | NA |
| ENSRNOG00000024620 | TRUE | TRUE | Mamdc2 | 1 | 2.21E+08 | 2.21E+08 | - | protein_coding | NA |
| ENSRNOG00000033217 | TRUE | TRUE | Esam | 8 | 37238287 | 37249215 | + | protein_coding | NA |
| ENSRNOG00000059750 | TRUE | TRUE | AC141102.1 | 2 | 1.83E+08 | 1.83E+08 | - | protein_coding | NA |
| ENSRNOG00000065000 | TRUE | TRUE | Lrrc8c | 14 | 4227832 | 4315249 | - | protein_coding | NA |
| ENSRNOG00000005039 | TRUE | TRUE | Tfpi | 3 | 69533156 | 69576880 | - | protein_coding | NA |
| ENSRNOG00000012742 | TRUE | TRUE | Irx2 | 1 | 30660960 | 30666378 | - | protein_coding | Homeobox |
| ENSRNOG00000008587 | TRUE | TRUE | Tek | 5 | 1.1E+08 | 1.1E+08 | + | protein_coding | NA |
| ENSRNOG00000000561 | TRUE | TRUE | Pald1 | 20 | 29270193 | 29334858 | - | protein_coding | NA |
| ENSRNOG00000019138 | TRUE | TRUE | Clec11a | 1 | 94801496 | 94804633 | - | protein_coding | NA |
| ENSRNOG00000000853 | TRUE | TRUE | Aif1 | 20 | 3646777 | 3652668 | + | protein_coding | NA |
| ENSRNOG00000009204 | TRUE | TRUE | Il17re | 4 | 1.47E+08 | 1.47E+08 | + | protein_coding | NA |
| ENSRNOG00000005998 | TRUE | TRUE | Smoc1 | 6 | 1.01E+08 | 1.01E+08 | + | protein_coding | NA |
| ENSRNOG00000054218 | TRUE | TRUE | Il18rap | 9 | 42768220 | 42800959 | + | protein_coding | NA |

| | | | | | | | | | |
|---|---|---|---|---|---|---|---|---|---|
| ENSRNOG00000004109 | TRUE | TRUE | Zfpm2 | 7 | 71678880 | 72116205 | + | protein_coding | zf-C2H2 |
| ENSRNOG00000013851 | TRUE | TRUE | Spry4 | 18 | 30436443 | 30453004 | - | protein_coding | NA |
| ENSRNOG00000000956 | TRUE | FALSE | Rasl11a | 12 | 8258159 | 8262576 | - | protein_coding | NA |
| ENSRNOG00000015086 | TRUE | TRUE | Plin1 | 1 | 1.34E+08 | 1.34E+08 | - | protein_coding | NA |
| ENSRNOG00000018804 | TRUE | TRUE | Ripor2 | 17 | 40323867 | 40548092 | - | protein_coding | NA |
| ENSRNOG00000010513 | TRUE | TRUE | Tfpi2 | 4 | 31982178 | 31986600 | - | protein_coding | NA |
| ENSRNOG00000016874 | TRUE | TRUE | Zfp521 | 18 | 4787295 | 5068458 | - | protein_coding | zf-C2H2 |
| ENSRNOG00000020424 | TRUE | FALSE | Plpp4 | 1 | 1.84E+08 | 1.84E+08 | + | protein_coding | NA |
| ENSRNOG00000025184 | TRUE | TRUE | Prss35 | 8 | 87714966 | 87731009 | + | protein_coding | NA |
| ENSRNOG00000016164 | TRUE | TRUE | Fcrl2 | 2 | 1.73E+08 | 1.73E+08 | - | protein_coding | NA |
| ENSRNOG00000016275 | TRUE | TRUE | Ttr | 18 | 11943789 | 11951008 | + | protein_coding | NA |
| ENSRNOG00000017412 | TRUE | TRUE | AABR07001512.1 | 1 | 48369044 | 48369595 | - | protein_coding | NA |
| ENSRNOG00000025895 | TRUE | TRUE | Cavin2 | 9 | 50301206 | 50314147 | - | protein_coding | NA |
| ENSRNOG00000038881 | TRUE | TRUE | Hcls1 | 11 | 63817476 | 63841014 | - | protein_coding | NA |
| ENSRNOG00000047545 | TRUE | TRUE | Adra2a | 1 | 2.53E+08 | 2.53E+08 | + | protein_coding | NA |
| ENSRNOG00000049289 | TRUE | TRUE | Robo4 | 8 | 37119988 | 37132519 | + | protein_coding | NA |
| ENSRNOG00000007457 | TRUE | TRUE | Serping1 | 3 | 69842739 | 69852034 | - | protein_coding | NA |
| ENSRNOG00000036837 | TRUE | TRUE | Nfe2 | 7 | 1.34E+08 | 1.34E+08 | - | protein_coding | TF_bZIP |
| ENSRNOG00000021261 | TRUE | TRUE | Rassf2 | 3 | 1.19E+08 | 1.19E+08 | - | protein_coding | NA |
| ENSRNOG00000001823 | TRUE | TRUE | St6gal1 | 11 | 77526837 | 77653310 | - | protein_coding | NA |
| ENSRNOG00000066573 | TRUE | TRUE | C4a | 20 | 4005731 | 4020080 | + | protein_coding | NA |
| ENSRNOG00000055564 | TRUE | TRUE | RGD1564664 | 3 | 79920129 | 79921523 | - | protein_coding | NA |
| ENSRNOG00000071066 | TRUE | TRUE | Stum | 13 | 92173544 | 92227213 | - | protein_coding | NA |
| ENSRNOG00000009832 | TRUE | TRUE | Slc39a14 | 15 | 45376917 | 45423524 | - | protein_coding | NA |
| ENSRNOG00000022884 | TRUE | TRUE | Cd84 | 13 | 84327553 | 84362076 | + | protein_coding | NA |
| ENSRNOG00000037148 | TRUE | TRUE | Adap2 | 10 | 65138020 | 65165604 | + | protein_coding | NA |
| ENSRNOG00000053494 | TRUE | TRUE | Mcpt1l1 | 15 | 29903980 | 29906645 | - | protein_coding | NA |
| ENSRNOG00000010128 | TRUE | TRUE | Slc27a2 | 3 | 1.14E+08 | 1.14E+08 | + | protein_coding | NA |
| ENSRNOG00000050325 | TRUE | TRUE | Adgre4 | 9 | 9760355 | 9900760 | + | protein_coding | NA |
| ENSRNOG00000005177 | TRUE | FALSE | Tp53i3 | 13 | 69620792 | 69652475 | + | protein_coding | NA |
| ENSRNOG00000020072 | TRUE | TRUE | Pcdhb9 | 18 | 29105298 | 29108468 | + | protein_coding | NA |
| ENSRNOG00000033588 | TRUE | TRUE | Siglec15 | 18 | 71505399 | 71521881 | - | protein_coding | NA |
| ENSRNOG00000008012 | TRUE | TRUE | Abcb1b | 4 | 25158362 | 25442709 | - | protein_coding | NA |
| ENSRNOG00000066894 | TRUE | TRUE | Tcaf2 | 4 | 71553704 | 71568433 | - | protein_coding | NA |

| | | | | | | | | | |
|---|---|---|---|---|---|---|---|---|---|
| ENSRNOG00000006807 | TRUE | TRUE | Aldob | 5 | 63889046 | 63902116 | - | protein_coding | NA |
| ENSRNOG00000012430 | TRUE | TRUE | Cxcr5 | 8 | 44843413 | 44857893 | - | protein_coding | NA |
| ENSRNOG00000008356 | TRUE | TRUE | Myo5c | 8 | 75989528 | 76066483 | + | protein_coding | NA |
| ENSRNOG00000004578 | TRUE | TRUE | Cthrc1 | 7 | 70122474 | 70132756 | + | protein_coding | NA |
| ENSRNOG00000024799 | TRUE | TRUE | Cd93 | 3 | 1.36E+08 | 1.36E+08 | - | protein_coding | NA |
| ENSRNOG00000013973 | TRUE | TRUE | Lcn2 | 3 | 15680687 | 15684095 | - | protein_coding | NA |
| ENSRNOG00000021919 | TRUE | TRUE | Rhoj | 6 | 94035483 | 94118275 | + | protein_coding | NA |
| ENSRNOG00000004089 | TRUE | TRUE | Enpp2 | 7 | 86202350 | 86324827 | - | protein_coding | NA |
| ENSRNOG00000021004 | TRUE | FALSE | Rasip1 | 1 | 96091074 | 96109562 | + | protein_coding | NA |
| ENSRNOG00000049115 | TRUE | FALSE | Ccr5 | 8 | 1.24E+08 | 1.24E+08 | + | protein_coding | NA |
| ENSRNOG00000057245 | TRUE | TRUE | Rbbp8nl | 3 | 1.67E+08 | 1.67E+08 | - | protein_coding | NA |
| ENSRNOG00000042229 | TRUE | TRUE | Gimap1 | 4 | 77682171 | 77691866 | + | protein_coding | NA |
| ENSRNOG00000003772 | TRUE | TRUE | Csrp2 | 7 | 46348686 | 46367745 | + | protein_coding | NA |
| ENSRNOG00000006709 | TRUE | TRUE | Pzp | 4 | 1.62E+08 | 1.62E+08 | - | protein_coding | NA |
| ENSRNOG00000001908 | TRUE | TRUE | Klhl6 | 11 | 80970917 | 81009677 | + | protein_coding | NA |
| ENSRNOG00000069663 | TRUE | TRUE | Arl4c | 9 | 89298005 | 89304513 | - | protein_coding | NA |
| ENSRNOG00000013179 | TRUE | TRUE | Tinagl1 | 5 | 1.43E+08 | 1.43E+08 | - | protein_coding | NA |
| ENSRNOG00000012881 | TRUE | TRUE | Fgl2 | 4 | 13710575 | 13716207 | - | protein_coding | NA |
| ENSRNOG00000007726 | TRUE | TRUE | Mcam | 8 | 44479376 | 44487571 | + | protein_coding | NA |
| ENSRNOG00000010478 | TRUE | TRUE | Serpina3n | 6 | 1.23E+08 | 1.23E+08 | + | protein_coding | NA |
| ENSRNOG00000053244 | TRUE | TRUE | Spp2 | 9 | 89007175 | 89026688 | + | protein_coding | NA |
| ENSRNOG00000005215 | TRUE | TRUE | Klhl4 | X | 79622113 | 79719480 | + | protein_coding | NA |
| ENSRNOG00000014684 | TRUE | TRUE | Npr1 | 2 | 1.76E+08 | 1.76E+08 | - | protein_coding | NA |
| ENSRNOG00000016248 | TRUE | TRUE | Sox18 | 3 | 1.69E+08 | 1.69E+08 | - | protein_coding | HMG |
| ENSRNOG00000005109 | TRUE | TRUE | Rprm | 3 | 38539581 | 38540998 | - | protein_coding | NA |
| ENSRNOG00000009790 | TRUE | TRUE | Kcnk3 | 6 | 25763228 | 25799153 | - | protein_coding | NA |
| ENSRNOG00000015902 | FALSE | TRUE | Cpxm2 | 1 | 1.87E+08 | 1.87E+08 | - | protein_coding | NA |
| ENSRNOG00000054957 | FALSE | TRUE | Sfrp4 | 17 | 45234097 | 45330736 | - | protein_coding | NA |
| ENSRNOG00000013484 | FALSE | TRUE | Gsta1 | 9 | 23703477 | 23720121 | - | protein_coding | NA |
| ENSRNOG00000020132 | FALSE | TRUE | Ttll10 | 5 | 1.67E+08 | 1.67E+08 | - | protein_coding | NA |
| ENSRNOG00000001766 | FALSE | TRUE | Tfrc | 11 | 68163413 | 68185257 | - | protein_coding | NA |
| ENSRNOG00000011250 | FALSE | TRUE | Inmt | 4 | 84318197 | 84322493 | - | protein_coding | NA |
| ENSRNOG00000062503 | FALSE | TRUE | ENSRNOG00000062503 | 15 | 95024031 | 95027881 | + | lncRNA | NA |
| ENSRNOG00000000515 | FALSE | TRUE | Mapk13 | 20 | 6835320 | 6844222 | + | protein_coding | NA |

| | | | | | | | | | |
|---|---|---|---|---|---|---|---|---|---|
| ENSRNOG00000025670 | FALSE | TRUE | Shisa3 | 14 | 40563673 | 40568244 | - | protein_coding | NA |
| ENSRNOG00000005931 | FALSE | TRUE | Cpq | 7 | 64264081 | 64724546 | + | protein_coding | NA |
| ENSRNOG00000026953 | FALSE | TRUE | Gpr88 | 2 | 2.04E+08 | 2.04E+08 | - | protein_coding | NA |
| ENSRNOG00000006623 | FALSE | TRUE | Cd302 | 3 | 44722325 | 44756045 | - | protein_coding | NA |
| ENSRNOG00000017976 | FALSE | TRUE | Slco2b1 | 1 | 1.54E+08 | 1.54E+08 | - | protein_coding | NA |
| ENSRNOG00000015695 | FALSE | TRUE | Sult5a1 | 19 | 51189287 | 51204362 | - | protein_coding | NA |
| ENSRNOG00000058891 | FALSE | TRUE | Cys1 | 6 | 41300836 | 41318071 | - | protein_coding | NA |
| ENSRNOG00000009450 | FALSE | TRUE | Hcn4 | 8 | 59221653 | 59259639 | + | protein_coding | NA |
| ENSRNOG00000018254 | FALSE | TRUE | Sncaip | 18 | 46207152 | 46343929 | + | protein_coding | NA |
| ENSRNOG00000069246 | FALSE | TRUE | Abcg3l3 | 14 | 4844218 | 4892194 | + | protein_coding | NA |
| ENSRNOG00000016544 | FALSE | TRUE | Arhgef28 | 2 | 29263886 | 29560672 | - | protein_coding | NA |
| ENSRNOG00000005307 | FALSE | TRUE | Slc4a10 | 3 | 46665265 | 46957268 | + | protein_coding | NA |
| ENSRNOG00000045829 | FALSE | TRUE | Thbs1 | 3 | 1.05E+08 | 1.05E+08 | + | protein_coding | NA |
| ENSRNOG00000010183 | FALSE | TRUE | Gask1b | 2 | 1.65E+08 | 1.65E+08 | + | protein_coding | NA |
| ENSRNOG00000054360 | FALSE | TRUE | Tspan11 | 4 | 1.61E+08 | 1.61E+08 | + | protein_coding | NA |
| ENSRNOG00000046007 | FALSE | TRUE | Cldn3 | 12 | 21708398 | 21711001 | - | protein_coding | NA |
| ENSRNOG00000016734 | FALSE | TRUE | Emilin3 | 3 | 1.5E+08 | 1.5E+08 | - | protein_coding | NA |
| ENSRNOG00000013141 | FALSE | TRUE | Eno2 | 4 | 1.58E+08 | 1.58E+08 | - | protein_coding | NA |
| ENSRNOG00000058842 | FALSE | TRUE | Sptbn2 | 1 | 2.02E+08 | 2.02E+08 | + | protein_coding | NA |
| ENSRNOG00000025209 | FALSE | TRUE | Plxnd1 | 4 | 1.49E+08 | 1.49E+08 | - | protein_coding | NA |
| ENSRNOG00000015075 | FALSE | TRUE | Stc1 | 15 | 44299621 | 44310264 | + | protein_coding | NA |
| ENSRNOG00000014060 | FALSE | TRUE | Mras | 8 | 99944036 | 99996408 | - | protein_coding | NA |
| ENSRNOG00000046905 | FALSE | TRUE | Sgce | 4 | 32771477 | 32842254 | - | protein_coding | NA |
| ENSRNOG00000052880 | FALSE | TRUE | Prph | 7 | 1.3E+08 | 1.3E+08 | + | protein_coding | NA |
| ENSRNOG00000066065 | FALSE | TRUE | Basp1 | 2 | 75816122 | 75864043 | - | protein_coding | NA |
| ENSRNOG00000006850 | FALSE | TRUE | Ovol2 | 3 | 1.32E+08 | 1.32E+08 | - | protein_coding | zf-C2H2 |
| ENSRNOG00000027582 | FALSE | TRUE | Dpysl4 | 1 | 1.94E+08 | 1.94E+08 | + | protein_coding | NA |
| ENSRNOG00000057125 | FALSE | TRUE | Ddr1 | 20 | 3044320 | 3064468 | + | protein_coding | NA |
| ENSRNOG00000046700 | FALSE | TRUE | Mettl27 | 12 | 21757329 | 21766685 | - | protein_coding | NA |
| ENSRNOG00000010997 | FALSE | TRUE | Ednrb | 15 | 80643043 | 80672115 | - | protein_coding | NA |
| ENSRNOG00000014226 | FALSE | TRUE | Plch2 | 5 | 1.66E+08 | 1.66E+08 | - | protein_coding | NA |
| ENSRNOG00000026910 | FALSE | TRUE | Gjb4 | 5 | 1.4E+08 | 1.4E+08 | - | protein_coding | NA |
| ENSRNOG00000012155 | FALSE | TRUE | Sox15 | 10 | 54372403 | 54376591 | + | protein_coding | HMG |
| ENSRNOG00000009385 | FALSE | TRUE | Pik3cg | 6 | 48766864 | 48802043 | - | protein_coding | NA |

| | | | | | | | | | |
|---|---|---|---|---|---|---|---|---|---|
| ENSRNOG00000005289 | FALSE | TRUE | Prkd3 | 6 | 16032585 | 16108443 | + | protein_coding | NA |
| ENSRNOG00000001769 | FALSE | TRUE | Tnk2 | 11 | 68114726 | 68154009 | - | protein_coding | NA |
| ENSRNOG00000012286 | FALSE | TRUE | Il20ra | 1 | 14451228 | 14493602 | + | protein_coding | NA |
| ENSRNOG00000031716 | FALSE | TRUE | Ecm2 | 17 | 15120196 | 15152516 | - | protein_coding | NA |
| ENSRNOG00000043445 | FALSE | TRUE | Them5 | 2 | 1.82E+08 | 1.82E+08 | + | protein_coding | NA |
| ENSRNOG00000056376 | FALSE | TRUE | Dusp22 | 17 | 33780981 | 33831321 | - | protein_coding | NA |
| ENSRNOG00000021027 | FALSE | TRUE | Dbp | 1 | 96175440 | 96180745 | + | protein_coding | TF_bZIP |
| ENSRNOG00000002997 | FALSE | TRUE | Slc9a3r2 | 10 | 13662461 | 13673049 | - | protein_coding | NA |
| ENSRNOG00000034191 | FALSE | TRUE | Fmo1 | 13 | 75182176 | 75214647 | - | protein_coding | NA |
| ENSRNOG00000008600 | FALSE | TRUE | Sytl1 | 5 | 1.45E+08 | 1.45E+08 | - | protein_coding | NA |
| ENSRNOG00000010260 | FALSE | TRUE | Dixdc1 | 8 | 51007838 | 51081090 | - | protein_coding | NA |
| ENSRNOG00000003120 | FALSE | TRUE | Prelp | 13 | 45370533 | 45380270 | - | protein_coding | NA |
| ENSRNOG00000012259 | FALSE | TRUE | Il22ra2 | 1 | 14364489 | 14377844 | + | protein_coding | NA |
| ENSRNOG00000065519 | FALSE | TRUE | ENSRNOG00000065519 | 6 | 48960883 | 48964139 | + | lncRNA | NA |
| ENSRNOG00000042524 | FALSE | TRUE | Milr1 | 10 | 91687831 | 91705282 | + | protein_coding | NA |
| ENSRNOG00000061910 | FALSE | TRUE | Igfbp3 | 14 | 82056347 | 82064083 | - | protein_coding | NA |
| ENSRNOG00000005854 | FALSE | TRUE | Angpt1 | 7 | 73531486 | 73784067 | - | protein_coding | NA |
| ENSRNOG00000069413 | FALSE | TRUE | S100a14 | 2 | 1.76E+08 | 1.76E+08 | + | protein_coding | NA |
| ENSRNOG00000011541 | FALSE | TRUE | Cygb | 10 | 1.02E+08 | 1.02E+08 | - | protein_coding | NA |
| ENSRNOG00000007455 | FALSE | TRUE | Tmem132e | 10 | 67330751 | 67386789 | + | protein_coding | NA |
| ENSRNOG00000019931 | FALSE | TRUE | Map3k21 | 19 | 53880238 | 53913576 | + | protein_coding | NA |
| ENSRNOG00000065287 | FALSE | TRUE | ENSRNOG00000065287 | 5 | 46569132 | 46586923 | - | lncRNA | NA |
| ENSRNOG00000020480 | FALSE | TRUE | Fads1 | 1 | 2.07E+08 | 2.07E+08 | + | protein_coding | NA |
| ENSRNOG00000030281 | FALSE | TRUE | Klk10 | 1 | 94235096 | 94239376 | + | protein_coding | NA |
| ENSRNOG00000056955 | FALSE | TRUE | Bicdl1 | 12 | 40876884 | 40966130 | + | protein_coding | NA |
| ENSRNOG00000009802 | FALSE | TRUE | Fam124a | 15 | 36799877 | 36853683 | + | protein_coding | NA |
| ENSRNOG00000002052 | FALSE | TRUE | Ccdc80 | 11 | 55685872 | 55719137 | - | protein_coding | NA |
| ENSRNOG00000007091 | FALSE | TRUE | Ly6e | 7 | 1.07E+08 | 1.07E+08 | + | protein_coding | NA |
| ENSRNOG00000006025 | FALSE | TRUE | Lamb3 | 13 | 1.05E+08 | 1.05E+08 | + | protein_coding | NA |
| ENSRNOG00000000800 | FALSE | TRUE | Man1a1 | 20 | 33202517 | 33385824 | - | protein_coding | NA |
| ENSRNOG00000013720 | FALSE | TRUE | Aebp1 | 14 | 80738892 | 80748877 | + | protein_coding | NA |
| ENSRNOG00000016397 | FALSE | TRUE | Tmprss13 | 8 | 45625626 | 45653938 | + | protein_coding | NA |
| ENSRNOG00000008819 | FALSE | TRUE | Krt84 | 7 | 1.33E+08 | 1.33E+08 | - | protein_coding | NA |
| ENSRNOG00000031162 | FALSE | TRUE | Ccl24 | 12 | 21100835 | 21104893 | + | protein_coding | NA |

| | | | | | | | | | |
|---|---|---|---|---|---|---|---|---|---|
| ENSRNOG00000012701 | FALSE | TRUE | Map7 | 1 | 14910551 | 15037574 | + | protein_coding | NA |
| ENSRNOG00000017109 | FALSE | TRUE | Rassf7 | 1 | 1.96E+08 | 1.96E+08 | + | protein_coding | NA |
| ENSRNOG00000010731 | FALSE | TRUE | Gpm6a | 16 | 36641284 | 36973475 | - | protein_coding | NA |
| ENSRNOG00000008965 | FALSE | TRUE | Socs2 | 7 | 30008578 | 30043548 | - | protein_coding | NA |
| ENSRNOG00000003682 | FALSE | TRUE | Hook2 | 19 | 23151870 | 23164181 | - | protein_coding | NA |
| ENSRNOG00000034198 | FALSE | TRUE | Tceal9 | X | 99228458 | 99247763 | + | protein_coding | NA |
| ENSRNOG00000010666 | FALSE | TRUE | Ccn5 | 3 | 1.52E+08 | 1.53E+08 | + | protein_coding | NA |
| ENSRNOG00000013872 | FALSE | TRUE | P2ry14 | 2 | 1.43E+08 | 1.43E+08 | - | protein_coding | NA |
| ENSRNOG00000024259 | FALSE | TRUE | Tmem54 | 5 | 1.41E+08 | 1.41E+08 | + | protein_coding | NA |
| ENSRNOG00000062599 | FALSE | TRUE | ENSRNOG00000062599 | X | 1.12E+08 | 1.12E+08 | - | lncRNA | NA |
| ENSRNOG00000028335 | FALSE | TRUE | Fat4 | 2 | 1.22E+08 | 1.22E+08 | + | protein_coding | NA |
| ENSRNOG00000045658 | FALSE | TRUE | AABR07044301.1 | 20 | 1407866 | 1408970 | + | protein_coding | NA |
| ENSRNOG00000019414 | FALSE | TRUE | Tmem79 | 2 | 1.74E+08 | 1.74E+08 | - | protein_coding | NA |
| ENSRNOG00000020038 | FALSE | TRUE | Chpf | 9 | 76963184 | 76967878 | - | protein_coding | NA |
| ENSRNOG00000000994 | FALSE | TRUE | Stxbp2 | 12 | 1689410 | 1700458 | + | protein_coding | NA |
| ENSRNOG00000018716 | FALSE | TRUE | Dennd2c | 2 | 1.91E+08 | 1.91E+08 | + | protein_coding | NA |
| ENSRNOG00000004498 | FALSE | TRUE | Scin | 6 | 57109403 | 57188242 | - | protein_coding | NA |
| ENSRNOG00000006889 | FALSE | TRUE | Ambp | 5 | 76568094 | 76578331 | - | protein_coding | NA |
| ENSRNOG00000042901 | FALSE | TRUE | Cfhr1 | 13 | 51369211 | 51410592 | - | protein_coding | NA |
| ENSRNOG00000003669 | FALSE | TRUE | Myocd | 10 | 49836641 | 49927627 | - | protein_coding | NA |
| ENSRNOG00000000177 | FALSE | TRUE | Plpp2 | 7 | 10175948 | 10184071 | + | protein_coding | NA |
| ENSRNOG00000013917 | FALSE | TRUE | Igsf10 | 2 | 1.44E+08 | 1.44E+08 | - | protein_coding | NA |
| ENSRNOG00000015519 | FALSE | TRUE | Ces1d | 19 | 22560770 | 22592351 | + | protein_coding | NA |
| ENSRNOG00000019244 | FALSE | TRUE | Mxra8 | 5 | 1.66E+08 | 1.66E+08 | + | protein_coding | NA |
| ENSRNOG00000036669 | FALSE | TRUE | Uts2r | 10 | 1.06E+08 | 1.06E+08 | + | protein_coding | NA |
| ENSRNOG00000009114 | FALSE | TRUE | Fam110b | 5 | 18789389 | 18929828 | + | protein_coding | NA |
| ENSRNOG00000020440 | FALSE | TRUE | Fads2 | 1 | 2.07E+08 | 2.07E+08 | - | protein_coding | NA |
| ENSRNOG00000014490 | FALSE | TRUE | Bdh2 | 2 | 2.24E+08 | 2.24E+08 | + | protein_coding | NA |
| ENSRNOG00000011820 | FALSE | TRUE | Acp3 | 8 | 1.05E+08 | 1.05E+08 | - | protein_coding | NA |
| ENSRNOG00000017159 | FALSE | TRUE | Dsc3 | 18 | 11377347 | 11413797 | - | protein_coding | NA |
| ENSRNOG00000046862 | FALSE | TRUE | Armcx1 | X | 97898883 | 97903299 | + | protein_coding | NA |
| ENSRNOG00000016632 | FALSE | TRUE | Dsg3 | 18 | 11798900 | 11830818 | + | protein_coding | NA |
| ENSRNOG00000033772 | FALSE | TRUE | Serpinb9 | 17 | 31420125 | 31443230 | - | protein_coding | NA |
| ENSRNOG00000008346 | FALSE | TRUE | Itgb6 | 3 | 45048044 | 45121671 | - | protein_coding | NA |

| | | | | | | | | | |
|---|---|---|---|---|---|---|---|---|---|
| ENSRNOG00000004516 | FALSE | TRUE | Itgbl1 | 15 | 1.01E+08 | 1.01E+08 | + | protein_coding | NA |
| ENSRNOG00000051235 | FALSE | TRUE | Cfb | 20 | 3951474 | 3976505 | + | protein_coding | NA |
| ENSRNOG00000001809 | FALSE | TRUE | Hrg | 11 | 78054498 | 78069389 | - | protein_coding | NA |
| ENSRNOG00000010430 | FALSE | TRUE | Tmem40 | 4 | 1.49E+08 | 1.49E+08 | - | protein_coding | NA |
| ENSRNOG00000005574 | FALSE | TRUE | Adamts8 | 8 | 29349114 | 29368404 | + | protein_coding | NA |
| ENSRNOG00000029597 | FALSE | TRUE | AABR07030544.1 | 10 | 89564967 | 89572321 | - | protein_coding | NA |
| ENSRNOG00000016636 | FALSE | TRUE | Lpin3 | 3 | 1.5E+08 | 1.5E+08 | + | protein_coding | NA |
| ENSRNOG00000008323 | FALSE | TRUE | Pitpnm3 | 10 | 56676261 | 56766584 | - | protein_coding | NA |
| ENSRNOG00000015522 | FALSE | TRUE | Tfap2a | 17 | 24024432 | 24047507 | + | protein_coding | AP-2 |
| ENSRNOG00000050420 | FALSE | TRUE | Krt5 | 7 | 1.33E+08 | 1.33E+08 | - | protein_coding | NA |
| ENSRNOG00000010017 | FALSE | TRUE | Wee1 | 1 | 1.64E+08 | 1.64E+08 | + | protein_coding | NA |
| ENSRNOG00000055025 | FALSE | TRUE | Fam25a | 16 | 9680668 | 9684474 | - | protein_coding | NA |
| ENSRNOG00000029778 | FALSE | TRUE | Maob | X | 5907266 | 6011003 | + | protein_coding | NA |
| ENSRNOG00000046791 | FALSE | TRUE | Sh3rf3 | 20 | 26765015 | 27099257 | + | protein_coding | NA |
| ENSRNOG00000055909 | FALSE | TRUE | Apoa4 | 8 | 46539082 | 46541469 | + | protein_coding | NA |
| ENSRNOG00000028845 | FALSE | TRUE | Ebf1 | 10 | 29099933 | 29487665 | + | protein_coding | COE |
| ENSRNOG00000030387 | FALSE | TRUE | Kng1 | 11 | 77812752 | 77835555 | - | protein_coding | NA |
| ENSRNOG00000029792 | FALSE | TRUE | NEWGENE_1308171 | 17 | 15032069 | 15052739 | - | protein_coding | NA |
| ENSRNOG00000018287 | FALSE | TRUE | RGD1307603 | 1 | 2.01E+08 | 2.01E+08 | - | protein_coding | NA |
| ENSRNOG00000008061 | FALSE | TRUE | Nuak1 | 7 | 19329933 | 19401913 | + | protein_coding | NA |
| ENSRNOG00000025120 | FALSE | TRUE | Gli1 | 7 | 63156926 | 63169251 | - | protein_coding | zf-C2H2 |
| ENSRNOG00000050104 | FALSE | TRUE | Clip3 | 1 | 85547206 | 85563184 | + | protein_coding | NA |
| ENSRNOG00000019667 | FALSE | TRUE | Ppfibp2 | 1 | 1.62E+08 | 1.62E+08 | + | protein_coding | NA |
| ENSRNOG00000008082 | FALSE | TRUE | Rgs6 | 6 | 1.02E+08 | 1.03E+08 | + | protein_coding | NA |
| ENSRNOG00000008184 | FALSE | TRUE | Esrp1 | 5 | 24428717 | 24482062 | - | protein_coding | NA |
| ENSRNOG00000009687 | FALSE | TRUE | Ccdc120 | X | 14753696 | 14772743 | + | protein_coding | NA |
| ENSRNOG00000020129 | FALSE | TRUE | Cdh3 | 19 | 34393727 | 34444084 | + | protein_coding | NA |
| ENSRNOG00000010616 | FALSE | TRUE | Ndor1 | 3 | 8062630 | 8070860 | - | protein_coding | NA |
| ENSRNOG00000003762 | FALSE | TRUE | Smarca1 | X | 1.27E+08 | 1.27E+08 | - | protein_coding | NA |
| ENSRNOG00000012346 | FALSE | TRUE | Epb41l2 | 1 | 19863009 | 20037721 | - | protein_coding | NA |
| ENSRNOG00000030170 | FALSE | TRUE | Krt10 | 10 | 84338706 | 84343701 | - | protein_coding | NA |
| ENSRNOG00000042717 | FALSE | TRUE | Ciart | 2 | 1.83E+08 | 1.83E+08 | - | protein_coding | NA |
| ENSRNOG00000011202 | FALSE | TRUE | Chrna4 | 3 | 1.68E+08 | 1.68E+08 | - | protein_coding | NA |
| ENSRNOG00000021274 | FALSE | TRUE | Fermt1 | 3 | 1.2E+08 | 1.2E+08 | - | protein_coding | NA |

| | | | | | | | | | |
|---|---|---|---|---|---|---|---|---|---|
| ENSRNOG00000037097 | FALSE | TRUE | Wfdc18 | 10 | 68588818 | 68589671 | + | protein_coding | NA |
| ENSRNOG00000008917 | FALSE | TRUE | Ehbp1 | 14 | 96093327 | 96345332 | - | protein_coding | NA |
| ENSRNOG00000018346 | FALSE | TRUE | Agtr1a | 17 | 34174429 | 34226946 | - | protein_coding | NA |
| ENSRNOG00000032202 | FALSE | TRUE | Creb3l3 | 7 | 8622614 | 8631048 | + | protein_coding | TF_bZIP |
| ENSRNOG00000019743 | FALSE | TRUE | Tmem63b | 9 | 15320432 | 15340720 | + | protein_coding | NA |
| ENSRNOG00000049471 | FALSE | TRUE | Steap3 | 13 | 31351954 | 31396519 | - | protein_coding | NA |
| ENSRNOG00000061768 | FALSE | TRUE | Slc43a3 | 3 | 70009313 | 70029749 | + | protein_coding | NA |
| ENSRNOG00000015152 | FALSE | TRUE | Pkp3 | 1 | 1.96E+08 | 1.96E+08 | + | protein_coding | NA |
| ENSRNOG00000001924 | FALSE | TRUE | Tp63 | 11 | 74838859 | 75049398 | - | protein_coding | P53 |
| ENSRNOG00000014448 | FALSE | TRUE | Arntl | 1 | 1.67E+08 | 1.67E+08 | + | protein_coding | bHLH |
| ENSRNOG00000034229 | FALSE | TRUE | Serpinb1b | 17 | 31320556 | 31327935 | - | protein_coding | NA |
| ENSRNOG00000007352 | FALSE | TRUE | Casp14 | 7 | 10926725 | 10933405 | + | protein_coding | NA |
| ENSRNOG00000017050 | FALSE | TRUE | Lmntd2 | 1 | 1.96E+08 | 1.96E+08 | - | protein_coding | NA |
| ENSRNOG00000064381 | FALSE | TRUE | Abcg3l2 | 14 | 4493262 | 4533306 | - | protein_coding | NA |
| ENSRNOG00000033434 | FALSE | TRUE | Casp12 | 8 | 2642434 | 2674037 | + | protein_coding | NA |
| ENSRNOG00000055259 | FALSE | TRUE | Phldb3 | 1 | 80201578 | 80219534 | + | protein_coding | NA |
| ENSRNOG00000014524 | FALSE | TRUE | S1pr3 | 17 | 13781050 | 13794505 | - | protein_coding | NA |
| ENSRNOG00000069320 | FALSE | TRUE | Cyp2a2 | 1 | 82263833 | 82289487 | + | protein_coding | NA |
| ENSRNOG00000069507 | FALSE | TRUE | Prr5 | 7 | 1.16E+08 | 1.16E+08 | + | protein_coding | NA |
| ENSRNOG00000004834 | FALSE | TRUE | Llgl2 | 10 | 1.01E+08 | 1.01E+08 | + | protein_coding | NA |
| ENSRNOG00000009291 | FALSE | TRUE | Dnase1l3 | 15 | 16922335 | 16948317 | + | protein_coding | NA |
| ENSRNOG00000013072 | FALSE | TRUE | Plxna4 | 4 | 60720255 | 61162206 | - | protein_coding | NA |
| ENSRNOG00000003634 | FALSE | TRUE | Zfp354a | 10 | 35396231 | 35408068 | + | protein_coding | zf-C2H2 |
| ENSRNOG00000002881 | FALSE | TRUE | Ddr2 | 13 | 82195463 | 82317363 | - | protein_coding | NA |
| ENSRNOG00000004367 | FALSE | TRUE | Elk3 | 7 | 27768599 | 27804084 | - | protein_coding | ETS |
| ENSRNOG00000018101 | FALSE | TRUE | Klc3 | 1 | 79045844 | 79055416 | - | protein_coding | NA |
| ENSRNOG00000019000 | FALSE | TRUE | Limk2 | 14 | 78218063 | 78286007 | - | protein_coding | NA |
| ENSRNOG00000000614 | FALSE | TRUE | Bicc1 | 20 | 17449560 | 17686776 | + | protein_coding | NA |
| ENSRNOG00000014573 | FALSE | TRUE | Ckmt1 | 3 | 1.08E+08 | 1.08E+08 | + | protein_coding | NA |
| ENSRNOG00000017117 | FALSE | TRUE | Ybx2 | 10 | 54659719 | 54665371 | + | protein_coding | CSD |
| ENSRNOG00000059705 | FALSE | TRUE | Elmo1 | 17 | 44286485 | 44822788 | - | protein_coding | NA |
| ENSRNOG00000005621 | FALSE | TRUE | Gxylt2 | 4 | 1.33E+08 | 1.33E+08 | + | protein_coding | NA |
| ENSRNOG00000013116 | FALSE | TRUE | Spink5 | 18 | 36264452 | 36332185 | + | protein_coding | NA |
| ENSRNOG00000015357 | FALSE | TRUE | Bmper | 8 | 21732225 | 21977256 | + | protein_coding | NA |

| | | | | | | | | | |
|---|---|---|---|---|---|---|---|---|---|
| ENSRNOG00000050106 | FALSE | TRUE | Rcc1 | 5 | 1.45E+08 | 1.45E+08 | - | protein_coding | NA |
| ENSRNOG00000001082 | FALSE | TRUE | Abcb9 | 12 | 32499225 | 32531931 | + | protein_coding | NA |
| ENSRNOG00000014443 | FALSE | TRUE | Pde5a | 2 | 2.11E+08 | 2.11E+08 | + | protein_coding | NA |
| ENSRNOG00000018330 | FALSE | TRUE | Fam83f | 7 | 1.12E+08 | 1.12E+08 | + | protein_coding | NA |
| ENSRNOG00000020238 | FALSE | TRUE | Plekhh3 | 10 | 86098469 | 86106880 | - | protein_coding | NA |
| ENSRNOG00000023551 | FALSE | TRUE | Sp6 | 10 | 82005294 | 82011013 | + | protein_coding | zf-C2H2 |
| ENSRNOG00000014153 | FALSE | TRUE | Lhfpl6 | 2 | 1.37E+08 | 1.37E+08 | + | protein_coding | NA |
| ENSRNOG00000009949 | FALSE | TRUE | Pcdh18 | 2 | 1.34E+08 | 1.34E+08 | - | protein_coding | NA |
| ENSRNOG00000067151 | FALSE | TRUE | ENSRNOG00000067151 | 10 | 49833365 | 49835784 | - | lncRNA | NA |
| ENSRNOG00000069489 | FALSE | TRUE | ENSRNOG00000069489 | 1 | 1.64E+08 | 1.64E+08 | + | lncRNA | NA |
| ENSRNOG00000038784 | FALSE | TRUE | Piezo2 | 18 | 56469680 | 56844216 | - | protein_coding | NA |
| ENSRNOG00000003088 | FALSE | TRUE | Arhgap31 | 11 | 62038635 | 62151564 | + | protein_coding | NA |
| ENSRNOG00000026371 | FALSE | TRUE | Krt17 | 10 | 85178675 | 85183392 | - | protein_coding | NA |
| ENSRNOG00000065303 | FALSE | TRUE | ENSRNOG00000065303 | 15 | 64319958 | 64321500 | + | protein_coding | NA |
| ENSRNOG00000012840 | FALSE | TRUE | Sparc | 10 | 39516406 | 39538396 | - | protein_coding | NA |
| ENSRNOG00000019477 | FALSE | TRUE | Zmynd15 | 10 | 55171198 | 55177812 | + | protein_coding | NA |
| ENSRNOG00000016670 | FALSE | TRUE | AABR07028769.1 | 17 | 81496670 | 81668029 | - | protein_coding | NA |
| ENSRNOG00000046660 | FALSE | TRUE | Gpc6 | 15 | 94029884 | 95024006 | + | protein_coding | NA |
| ENSRNOG00000013928 | FALSE | TRUE | Dsp | 17 | 26623588 | 26671800 | - | protein_coding | NA |
| ENSRNOG00000000986 | FALSE | TRUE | Camsap3 | 12 | 1643251 | 1666685 | + | protein_coding | NA |
| ENSRNOG00000019211 | FALSE | TRUE | Olfml3 | 2 | 1.91E+08 | 1.91E+08 | - | protein_coding | NA |
| ENSRNOG00000022321 | FALSE | TRUE | Hapln3 | 1 | 1.33E+08 | 1.33E+08 | - | protein_coding | NA |
| ENSRNOG00000003430 | FALSE | TRUE | F9 | X | 1.38E+08 | 1.38E+08 | + | protein_coding | NA |
| ENSRNOG00000013265 | FALSE | TRUE | Tgfbr2 | 8 | 1.16E+08 | 1.16E+08 | - | protein_coding | NA |
| ENSRNOG00000025998 | FALSE | TRUE | Crybg1 | 20 | 47426183 | 47621392 | - | protein_coding | NA |
| ENSRNOG00000047306 | FALSE | TRUE | Stap2 | 9 | 828372 | 837219 | - | protein_coding | NA |
| ENSRNOG00000017731 | FALSE | TRUE | Agpat4 | 1 | 48527323 | 48633345 | - | protein_coding | NA |
| ENSRNOG00000007697 | FALSE | TRUE | C8a | 5 | 1.2E+08 | 1.2E+08 | - | protein_coding | NA |
| ENSRNOG00000007049 | FALSE | TRUE | Bcl11a | 14 | 98030461 | 98124180 | + | protein_coding | zf-C2H2 |
| ENSRNOG00000021474 | FALSE | TRUE | Siglec5 | 1 | 93768319 | 93776694 | + | protein_coding | NA |
| ENSRNOG00000031852 | FALSE | TRUE | Lsamp | 11 | 58554805 | 60717029 | - | protein_coding | NA |
| ENSRNOG00000021015 | FALSE | TRUE | Sbsn | 1 | 86001567 | 86006034 | + | protein_coding | NA |
| ENSRNOG00000002097 | FALSE | TRUE | Rasl11b | 14 | 34014509 | 34019152 | - | protein_coding | NA |
| ENSRNOG00000019977 | FALSE | TRUE | Ptprf | 5 | 1.32E+08 | 1.32E+08 | - | protein_coding | NA |

| | | | | | | | | | |
|---|---|---|---|---|---|---|---|---|---|
| ENSRNOG00000026133 | FALSE | TRUE | Ly6d | 7 | 1.07E+08 | 1.07E+08 | - | protein_coding | NA |
| ENSRNOG00000027456 | FALSE | TRUE | Cdc42bpg | 1 | 2.04E+08 | 2.04E+08 | + | protein_coding | NA |
| ENSRNOG00000037909 | FALSE | TRUE | Ppm1f | 11 | 84064420 | 84094340 | - | protein_coding | NA |
| ENSRNOG00000012110 | FALSE | TRUE | Col17a1 | 1 | 2.47E+08 | 2.47E+08 | - | protein_coding | NA |
| ENSRNOG00000026661 | FALSE | TRUE | Hcar1 | 12 | 32711027 | 32712082 | + | protein_coding | NA |
| ENSRNOG00000037951 | FALSE | TRUE | AABR07039210.1 | X | 66970151 | 67200911 | + | protein_coding | NA |
| ENSRNOG00000004870 | FALSE | TRUE | Dyrk3 | 13 | 42594121 | 42604778 | - | protein_coding | NA |
| ENSRNOG00000053200 | FALSE | TRUE | Dock10 | 9 | 81843604 | 82101525 | - | protein_coding | NA |
| ENSRNOG00000069375 | FALSE | TRUE | Kctd12b | X | 17643615 | 17654820 | + | protein_coding | NA |
| ENSRNOG00000062944 | FALSE | TRUE | Kcnj15 | 11 | 34577363 | 34621952 | + | protein_coding | NA |
| ENSRNOG00000039336 | FALSE | TRUE | Hrct1 | 5 | 57982470 | 57982790 | + | protein_coding | NA |
| ENSRNOG00000005246 | FALSE | TRUE | Tfap2c | 3 | 1.61E+08 | 1.61E+08 | + | protein_coding | AP-2 |
| ENSRNOG00000024707 | FALSE | TRUE | Tp73 | 5 | 1.65E+08 | 1.65E+08 | - | protein_coding | P53 |
| ENSRNOG00000020380 | FALSE | TRUE | Lgals7 | 1 | 84150252 | 84152015 | + | protein_coding | NA |
| ENSRNOG00000021220 | FALSE | TRUE | Cpxm1 | 3 | 1.18E+08 | 1.18E+08 | - | protein_coding | NA |
| ENSRNOG00000050469 | FALSE | TRUE | Gng11 | 4 | 32023003 | 32028442 | + | protein_coding | NA |
| ENSRNOG00000014464 | FALSE | TRUE | Tnfsf13b | 16 | 79462402 | 79492693 | - | protein_coding | NA |
| ENSRNOG00000047967 | FALSE | TRUE | Arhgef19 | 5 | 1.54E+08 | 1.54E+08 | + | protein_coding | NA |
| ENSRNOG00000027871 | FALSE | TRUE | Rapgefl1 | 10 | 83793694 | 83810371 | + | protein_coding | NA |
| ENSRNOG00000007765 | FALSE | TRUE | Frzb | 3 | 65332277 | 65365208 | - | protein_coding | NA |
| ENSRNOG00000005705 | FALSE | TRUE | Qpct | 6 | 15964657 | 15996711 | - | protein_coding | NA |
| ENSRNOG00000014080 | FALSE | TRUE | Kif23 | 8 | 62397948 | 62425072 | - | protein_coding | NA |
| ENSRNOG00000012568 | FALSE | TRUE | Madd | 3 | 77114314 | 77157701 | - | protein_coding | NA |
| ENSRNOG00000023148 | FALSE | TRUE | Col11a1 | 2 | 2.02E+08 | 2.02E+08 | + | protein_coding | NA |
| ENSRNOG00000000781 | FALSE | TRUE | Rnf39 | 20 | 1604794 | 1610283 | - | protein_coding | NA |
| ENSRNOG00000070011 | FALSE | TRUE | ENSRNOG00000070011 | 18 | 55095230 | 55118825 | - | lncRNA | NA |
| ENSRNOG00000008947 | FALSE | TRUE | Cpz | 14 | 75223605 | 75246945 | + | protein_coding | NA |
| ENSRNOG00000012060 | FALSE | TRUE | Gucy1b1 | 2 | 1.67E+08 | 1.67E+08 | - | protein_coding | NA |
| ENSRNOG00000046195 | FALSE | TRUE | AABR07029596.1 | 10 | 38133322 | 38179720 | - | protein_coding | NA |
| ENSRNOG00000046829 | FALSE | TRUE | Kdr | 14 | 32217871 | 32261018 | + | protein_coding | NA |
| ENSRNOG00000013589 | FALSE | TRUE | Cxcl12 | 4 | 1.5E+08 | 1.5E+08 | + | protein_coding | NA |
| ENSRNOG00000043378 | FALSE | TRUE | Nkpd1 | 1 | 79167130 | 79174234 | + | protein_coding | NA |
| ENSRNOG00000069539 | FALSE | TRUE | Zfp74 | 1 | 85158980 | 85183522 | - | protein_coding | zf-C2H2 |
| ENSRNOG00000001217 | FALSE | TRUE | Lrrc3 | 20 | 10758955 | 10762067 | + | protein_coding | NA |

| | | | | | | | | | |
|---|---|---|---|---|---|---|---|---|---|
| ENSRNOG00000019648 | FALSE | TRUE | Col6a3 | 9 | 91361583 | 91439471 | - | protein_coding | NA |
| ENSRNOG00000008758 | FALSE | TRUE | Tspan18 | 3 | 79161662 | 79299852 | - | protein_coding | NA |
| ENSRNOG00000019862 | FALSE | TRUE | Pinlyp | 1 | 80134643 | 80139996 | - | protein_coding | NA |
| ENSRNOG00000011750 | FALSE | TRUE | Fam180a | 4 | 63992867 | 64008047 | - | protein_coding | NA |
| ENSRNOG00000004706 | FALSE | TRUE | Vit | 6 | 16445955 | 16565665 | - | protein_coding | NA |
| ENSRNOG00000065213 | FALSE | TRUE | Ccdc85c | 6 | 1.27E+08 | 1.27E+08 | - | protein_coding | NA |
| ENSRNOG00000027767 | FALSE | TRUE | Slc38a5 | X | 14213729 | 14222498 | - | protein_coding | NA |
| ENSRNOG00000033921 | FALSE | TRUE | Als2cl | 8 | 1.11E+08 | 1.11E+08 | + | protein_coding | NA |
| ENSRNOG00000065208 | FALSE | TRUE | ENSRNOG00000065208 | 13 | 85912044 | 85929007 | - | protein_coding | NA |
| ENSRNOG00000006689 | FALSE | TRUE | Chd7 | 5 | 21812070 | 21995358 | + | protein_coding | NA |
| ENSRNOG00000042289 | FALSE | TRUE | Plcxd2 | 11 | 54789477 | 54853344 | + | protein_coding | NA |
| ENSRNOG00000017525 | FALSE | TRUE | Epha1 | 4 | 71246409 | 71260846 | - | protein_coding | NA |
| ENSRNOG00000004165 | FALSE | TRUE | Prkd1 | 6 | 67725905 | 68039042 | - | protein_coding | NA |
| ENSRNOG00000057040 | FALSE | TRUE | Spns2 | 10 | 57066897 | 57105957 | - | protein_coding | NA |
| ENSRNOG00000013452 | FALSE | TRUE | Rcn1 | 3 | 91841052 | 91855295 | - | protein_coding | NA |
| ENSRNOG00000055933 | FALSE | TRUE | Kdf1 | 5 | 1.46E+08 | 1.46E+08 | + | protein_coding | NA |
| ENSRNOG00000004448 | FALSE | TRUE | Acss3 | 7 | 42242652 | 42450230 | - | protein_coding | NA |
| ENSRNOG00000066703 | FALSE | TRUE | Lhfpl3 | 4 | 11837994 | 12379102 | - | protein_coding | NA |
| ENSRNOG00000020300 | FALSE | TRUE | Lsp1 | 1 | 1.98E+08 | 1.98E+08 | + | protein_coding | NA |
| ENSRNOG00000020579 | FALSE | TRUE | Col7a1 | 8 | 1.1E+08 | 1.1E+08 | + | protein_coding | NA |
| ENSRNOG00000002061 | FALSE | TRUE | Ptpn13 | 14 | 6108211 | 6282563 | - | protein_coding | NA |
| ENSRNOG00000055608 | FALSE | TRUE | ENSRNOG00000055608 | 1 | 79884945 | 79900149 | - | protein_coding | zf-C2H2 |
| ENSRNOG00000064828 | FALSE | TRUE | ENSRNOG00000064828 | 2 | 1.91E+08 | 1.91E+08 | - | lncRNA | NA |
| ENSRNOG00000005434 | FALSE | TRUE | Sptbn1 | 14 | 1.04E+08 | 1.04E+08 | - | protein_coding | NA |
| ENSRNOG00000027350 | FALSE | TRUE | Tns4 | 10 | 84021966 | 84041346 | - | protein_coding | NA |
| ENSRNOG00000001275 | FALSE | TRUE | Tmem184a | 12 | 14822898 | 14832065 | + | protein_coding | NA |
| ENSRNOG00000053430 | FALSE | TRUE | Slco4a1 | 3 | 1.68E+08 | 1.68E+08 | + | protein_coding | NA |
| ENSRNOG00000014035 | FALSE | TRUE | Arhgef4 | 9 | 36861835 | 37005075 | + | protein_coding | NA |
| ENSRNOG00000022983 | FALSE | TRUE | Trim17 | 10 | 43740604 | 43750919 | + | protein_coding | NA |
| ENSRNOG00000012175 | FALSE | TRUE | Nfatc2 | 3 | 1.57E+08 | 1.57E+08 | - | protein_coding | RHD |
| ENSRNOG00000010870 | FALSE | TRUE | Foxn1 | 10 | 63251400 | 63273710 | - | protein_coding | Fork_head |
| ENSRNOG00000004687 | FALSE | TRUE | Thbd | 3 | 1.36E+08 | 1.36E+08 | - | protein_coding | NA |
| ENSRNOG00000066913 | FALSE | TRUE | Serpinb3a | 13 | 23236972 | 23246985 | - | protein_coding | NA |
| ENSRNOG00000018949 | FALSE | TRUE | Dlk2 | 9 | 14676562 | 14681594 | - | protein_coding | NA |

| | | | | | | | | | |
|---|---|---|---|---|---|---|---|---|---|
| ENSRNOG00000027924 | FALSE | TRUE | Fam83g | 10 | 46363051 | 46388019 | + | protein_coding | NA |
| ENSRNOG00000039560 | FALSE | TRUE | Omd | 17 | 15060217 | 15068441 | - | protein_coding | NA |
| ENSRNOG00000063796 | FALSE | TRUE | Foxf2 | 17 | 32836777 | 32842176 | - | protein_coding | Fork_head |
| ENSRNOG00000016381 | FALSE | TRUE | Ust | 1 | 2669592 | 2962044 | - | protein_coding | NA |
| ENSRNOG00000022255 | FALSE | TRUE | Tmprss11e | 14 | 21282823 | 21327184 | + | protein_coding | NA |
| ENSRNOG00000030019 | FALSE | TRUE | Atp1a1 | 2 | 1.89E+08 | 1.89E+08 | - | protein_coding | NA |
| ENSRNOG00000015368 | FALSE | TRUE | Pdilt | 1 | 1.74E+08 | 1.74E+08 | - | protein_coding | NA |
| ENSRNOG00000019549 | FALSE | TRUE | Akap12 | 1 | 40730123 | 40819886 | + | protein_coding | NA |
| ENSRNOG00000043342 | FALSE | TRUE | ENSRNOG00000043342 | 16 | 12868270 | 12875876 | - | protein_coding | NA |
| ENSRNOG00000055567 | FALSE | TRUE | Fmnl2 | 3 | 37348574 | 37624754 | + | protein_coding | NA |
| ENSRNOG00000049929 | FALSE | TRUE | AABR07030494.1 | 10 | 87574943 | 87575317 | - | protein_coding | NA |
| ENSRNOG00000015896 | FALSE | TRUE | Rbpms2 | 8 | 66153593 | 66184268 | + | protein_coding | NA |
| ENSRNOG00000063458 | FALSE | TRUE | ENSRNOG00000063458 | 8 | 889638 | 899464 | + | lncRNA | NA |
| ENSRNOG00000023640 | FALSE | TRUE | Rab25 | 2 | 1.74E+08 | 1.74E+08 | - | protein_coding | NA |
| ENSRNOG00000010630 | FALSE | TRUE | Prcp | 1 | 1.47E+08 | 1.47E+08 | + | protein_coding | NA |
| ENSRNOG00000017704 | FALSE | TRUE | Sema3f | 8 | 1.08E+08 | 1.08E+08 | - | protein_coding | NA |
| ENSRNOG00000025151 | FALSE | TRUE | Ildr2 | 13 | 78518802 | 78576924 | + | protein_coding | NA |
| ENSRNOG00000002347 | FALSE | TRUE | Rgl1 | 13 | 64635246 | 64904191 | - | protein_coding | NA |
| ENSRNOG00000011559 | FALSE | TRUE | Cnn3 | 2 | 2.1E+08 | 2.1E+08 | + | protein_coding | NA |
| ENSRNOG00000029510 | FALSE | TRUE | Plxnb1 | 8 | 1.1E+08 | 1.1E+08 | + | protein_coding | NA |
| ENSRNOG00000008765 | FALSE | TRUE | Zfhx4 | 2 | 96224767 | 96408228 | - | protein_coding | Homeobox |
| ENSRNOG00000025705 | FALSE | TRUE | Armcx2 | X | 97980660 | 97985552 | - | protein_coding | NA |
| ENSRNOG00000060979 | FALSE | TRUE | Hspa13 | 11 | 14373275 | 14389895 | - | protein_coding | NA |
| ENSRNOG00000020901 | FALSE | TRUE | S1pr5 | 8 | 19786663 | 19791795 | - | protein_coding | NA |
| ENSRNOG00000039357 | FALSE | TRUE | Niban3 | 16 | 18307221 | 18318585 | + | protein_coding | NA |
| ENSRNOG00000007027 | FALSE | TRUE | Hgf | 4 | 18677101 | 18745409 | - | protein_coding | NA |
| ENSRNOG00000002926 | FALSE | TRUE | Uap1 | 13 | 82342946 | 82377469 | - | protein_coding | NA |
| ENSRNOG00000043203 | FALSE | TRUE | Krt75 | 7 | 1.33E+08 | 1.33E+08 | - | protein_coding | NA |
| ENSRNOG00000046947 | FALSE | TRUE | Ak5 | 2 | 2.41E+08 | 2.42E+08 | - | protein_coding | NA |
| ENSRNOG00000021771 | FALSE | TRUE | Trim29 | 8 | 43682221 | 43706992 | + | protein_coding | NA |
| ENSRNOG00000018525 | FALSE | TRUE | Slc4a9 | 18 | 28128740 | 28141543 | + | protein_coding | NA |
| ENSRNOG00000018580 | FALSE | TRUE | Klk8 | 1 | 94251803 | 94258646 | + | protein_coding | NA |
| ENSRNOG00000019883 | FALSE | TRUE | Pak4 | 1 | 83849904 | 83859413 | - | protein_coding | NA |
| ENSRNOG00000014481 | FALSE | TRUE | Adtrp | 17 | 22930740 | 22993826 | + | protein_coding | NA |

| | | | | | | | | | |
|---|---|---|---|---|---|---|---|---|---|
| ENSRNOG00000015461 | FALSE | TRUE | Serpine2 | 9 | 81124804 | 81188826 | - | protein_coding | NA |
| ENSRNOG00000012478 | FALSE | TRUE | Shox2 | 2 | 1.51E+08 | 1.51E+08 | - | protein_coding | Homeobox |
| ENSRNOG00000014672 | FALSE | TRUE | Hs3st6 | 10 | 13781993 | 13788133 | + | protein_coding | NA |
| ENSRNOG00000064984 | FALSE | TRUE | Plscr2 | 8 | 92808398 | 92845932 | + | protein_coding | NA |
| ENSRNOG00000046912 | FALSE | TRUE | Nr1d2 | 15 | 7524257 | 7550553 | + | protein_coding | THR-like |
| ENSRNOG00000015526 | FALSE | TRUE | Rhcg | 1 | 1.34E+08 | 1.34E+08 | - | protein_coding | NA |
| ENSRNOG00000014137 | FALSE | TRUE | Fbln1 | 7 | 1.16E+08 | 1.16E+08 | + | protein_coding | NA |
| ENSRNOG00000021116 | FALSE | TRUE | Bnipl | 2 | 1.83E+08 | 1.83E+08 | - | protein_coding | NA |
| ENSRNOG00000046699 | FALSE | TRUE | Slpi | 3 | 1.53E+08 | 1.53E+08 | - | protein_coding | NA |
| ENSRNOG00000006079 | FALSE | TRUE | Psd4 | 3 | 7137155 | 7173571 | + | protein_coding | NA |
| ENSRNOG00000002238 | FALSE | TRUE | Sema5b | 11 | 65102031 | 65225311 | - | protein_coding | NA |
| ENSRNOG00000018500 | FALSE | TRUE | Frmd4a | 17 | 73667789 | 74258687 | - | protein_coding | NA |
| ENSRNOG00000014710 | FALSE | TRUE | Prom2 | 3 | 1.15E+08 | 1.15E+08 | - | protein_coding | NA |
| ENSRNOG00000015068 | FALSE | TRUE | Il11ra1 | 5 | 56935516 | 56941408 | + | protein_coding | NA |
| ENSRNOG00000011070 | FALSE | TRUE | Gnb4 | 2 | 1.15E+08 | 1.15E+08 | - | protein_coding | NA |
| ENSRNOG00000063360 | FALSE | TRUE | Iqank1 | 7 | 1.08E+08 | 1.08E+08 | + | protein_coding | NA |
| ENSRNOG00000037839 | FALSE | TRUE | P2ry10 | X | 72111264 | 72212265 | + | protein_coding | NA |
| ENSRNOG00000005176 | FALSE | TRUE | Map7d2 | X | 35372700 | 35488091 | - | protein_coding | NA |
| ENSRNOG00000011459 | FALSE | TRUE | Rhbdf2 | 10 | 1.02E+08 | 1.02E+08 | - | protein_coding | NA |
| ENSRNOG00000016581 | FALSE | TRUE | Serpinb1a | 17 | 31590677 | 31599102 | + | protein_coding | NA |
| ENSRNOG00000025042 | FALSE | TRUE | Pde3a | 4 | 1.74E+08 | 1.74E+08 | + | protein_coding | NA |
| ENSRNOG00000012663 | FALSE | TRUE | Mfsd6 | 9 | 48773414 | 48858465 | + | protein_coding | NA |
| ENSRNOG00000017428 | FALSE | TRUE | Map1b | 2 | 30817261 | 30910317 | - | protein_coding | NA |
| ENSRNOG00000011068 | FALSE | TRUE | Papss2 | 1 | 2.3E+08 | 2.31E+08 | + | protein_coding | NA |
| ENSRNOG00000004517 | FALSE | TRUE | Igf1 | 7 | 22282930 | 22359296 | + | protein_coding | NA |
| ENSRNOG00000025843 | FALSE | TRUE | Ccdc102a | 19 | 10103361 | 10118657 | + | protein_coding | NA |
| ENSRNOG00000004730 | FALSE | TRUE | Meis2 | 3 | 1.03E+08 | 1.03E+08 | - | protein_coding | Homeobox |
| ENSRNOG00000003899 | FALSE | TRUE | Krt15 | 10 | 85066802 | 85171799 | - | protein_coding | NA |
| ENSRNOG00000006353 | FALSE | TRUE | Large2 | 3 | 78336056 | 78342184 | - | protein_coding | NA |
| ENSRNOG00000069372 | FALSE | TRUE | Fam83b | 8 | 77287351 | 77363795 | - | protein_coding | NA |
| ENSRNOG00000004614 | FALSE | TRUE | Lypd6b | 3 | 34199599 | 34360770 | + | protein_coding | NA |
| ENSRNOG00000026050 | FALSE | TRUE | Epb41l4a | 18 | 25406057 | 25615149 | - | protein_coding | NA |
| ENSRNOG00000054212 | FALSE | TRUE | Pde1a | 3 | 64747269 | 65024874 | - | protein_coding | NA |
| ENSRNOG00000012424 | FALSE | TRUE | Adam23 | 9 | 64862878 | 65006515 | + | protein_coding | NA |

| | | | | | | | | | |
|---|---|---|---|---|---|---|---|---|---|
| ENSRNOG00000012535 | FALSE | TRUE | Adgrf4 | 9 | 18272050 | 18303217 | + | protein_coding | NA |
| ENSRNOG00000056184 | FALSE | TRUE | Lrp2 | 3 | 54189308 | 54346708 | - | protein_coding | NA |
| ENSRNOG00000032941 | FALSE | TRUE | Mab21l1 | 2 | 1.4E+08 | 1.4E+08 | + | protein_coding | NA |
| ENSRNOG00000002667 | FALSE | TRUE | Lamc2 | 13 | 65284664 | 65344200 | - | protein_coding | NA |
| ENSRNOG00000010098 | FALSE | TRUE | Plek2 | 6 | 97701106 | 97719326 | - | protein_coding | NA |
| ENSRNOG00000010529 | FALSE | TRUE | Thbs2 | 1 | 55670394 | 55699789 | - | protein_coding | NA |
| ENSRNOG00000031801 | FALSE | TRUE | Ephb3 | 11 | 79840668 | 79859370 | - | protein_coding | NA |
| ENSRNOG00000008602 | FALSE | TRUE | Steap4 | 4 | 26054671 | 26099859 | - | protein_coding | NA |
| ENSRNOG00000028460 | FALSE | TRUE | Lrrc8e | 12 | 2578315 | 2589412 | + | protein_coding | NA |
| ENSRNOG00000025948 | FALSE | TRUE | Inava | 13 | 47666447 | 47686729 | - | protein_coding | NA |
| ENSRNOG00000020342 | FALSE | TRUE | Samd11 | 5 | 1.67E+08 | 1.67E+08 | - | protein_coding | SAND |
| ENSRNOG00000002832 | FALSE | TRUE | Slc16a2 | X | 68723261 | 68848771 | - | protein_coding | NA |
| ENSRNOG00000064516 | FALSE | TRUE | Rtl6 | 7 | 1.16E+08 | 1.16E+08 | - | protein_coding | NA |
| ENSRNOG00000012108 | FALSE | TRUE | Thsd1 | 16 | 69771408 | 69801504 | - | protein_coding | NA |
| ENSRNOG00000020455 | FALSE | TRUE | Cst6 | 1 | 2.03E+08 | 2.03E+08 | - | protein_coding | NA |
| ENSRNOG00000010076 | FALSE | TRUE | Pkp1 | 13 | 47309614 | 47357465 | - | protein_coding | NA |
| ENSRNOG00000011994 | FALSE | TRUE | Perp | 1 | 13542067 | 13554511 | + | protein_coding | NA |
| ENSRNOG00000065794 | FALSE | TRUE | Mafk | 12 | 14832249 | 14858888 | - | lncRNA | NA |
| ENSRNOG00000004889 | FALSE | TRUE | Vangl2 | 13 | 84465527 | 84489378 | - | protein_coding | NA |
| ENSRNOG00000020904 | FALSE | TRUE | Cdc42ep2 | 1 | 2.03E+08 | 2.03E+08 | - | protein_coding | NA |
| ENSRNOG00000010780 | FALSE | TRUE | Dlc1 | 16 | 55291584 | 55671439 | + | protein_coding | NA |
| ENSRNOG00000024557 | FALSE | TRUE | Cep112 | 10 | 93366136 | 93833131 | + | protein_coding | NA |
| ENSRNOG00000018109 | FALSE | TRUE | Clic4 | 5 | 1.47E+08 | 1.48E+08 | - | protein_coding | NA |
| ENSRNOG00000025608 | FALSE | TRUE | Lrat | 2 | 1.68E+08 | 1.68E+08 | - | protein_coding | NA |
| ENSRNOG00000050828 | FALSE | TRUE | Vkorc1 | 1 | 1.83E+08 | 1.83E+08 | - | protein_coding | NA |
| ENSRNOG00000053086 | FALSE | TRUE | Selenop | 2 | 52498339 | 52508852 | + | protein_coding | NA |
| ENSRNOG00000009980 | FALSE | TRUE | Plpp1 | 2 | 44438994 | 44501268 | + | protein_coding | NA |
| ENSRNOG00000003732 | FALSE | TRUE | Flrt2 | 6 | 1.15E+08 | 1.15E+08 | + | protein_coding | NA |
| ENSRNOG00000004330 | FALSE | TRUE | Chrdl1 | X | 1.07E+08 | 1.07E+08 | - | protein_coding | NA |
| ENSRNOG00000069729 | FALSE | TRUE | ENSRNOG00000069729 | 1 | 1.81E+08 | 1.81E+08 | - | lncRNA | NA |
| ENSRNOG00000010600 | FALSE | TRUE | Cysrt1 | 3 | 8053482 | 8059721 | - | protein_coding | NA |
| ENSRNOG00000058423 | FALSE | TRUE | Pnma5 | X | 1.51E+08 | 1.51E+08 | - | protein_coding | NA |
| ENSRNOG00000004013 | FALSE | TRUE | Grip1 | 7 | 54934250 | 55592273 | + | protein_coding | NA |
| ENSRNOG00000023109 | FALSE | TRUE | Icoslg | 20 | 10600420 | 10610703 | - | protein_coding | NA |

| | | | | | | | | | |
|---|---|---|---|---|---|---|---|---|---|
| ENSRNOG00000012616 | FALSE | TRUE | Ppt1 | 5 | 1.35E+08 | 1.35E+08 | + | protein_coding | NA |
| ENSRNOG00000010555 | FALSE | TRUE | Phyhip | 15 | 45533974 | 45545221 | + | protein_coding | NA |
| ENSRNOG00000016374 | FALSE | TRUE | Fgfr2 | 1 | 1.85E+08 | 1.85E+08 | - | protein_coding | NA |
| ENSRNOG00000042848 | FALSE | TRUE | Jam2 | 11 | 23831106 | 23880063 | + | protein_coding | NA |
| ENSRNOG00000065273 | FALSE | TRUE | ENSRNOG00000065273 | 1 | 1.3E+08 | 1.3E+08 | - | protein_coding | NA |
| ENSRNOG00000012575 | FALSE | TRUE | Fat2 | 10 | 39364073 | 39456216 | - | protein_coding | NA |
| ENSRNOG00000028677 | FALSE | TRUE | 4930503L19Rik | 18 | 64065740 | 64089415 | - | protein_coding | NA |
| ENSRNOG00000054695 | FALSE | TRUE | Calcrl | 3 | 69430120 | 69525910 | - | protein_coding | NA |
| ENSRNOG00000020676 | FALSE | TRUE | Ppp1r14a | 1 | 84586627 | 84590671 | + | protein_coding | NA |
| ENSRNOG00000006255 | FALSE | TRUE | Nipal4 | 10 | 30583926 | 30600640 | - | protein_coding | NA |
| ENSRNOG00000066224 | FALSE | TRUE | ENSRNOG00000066224 | 1 | 61696583 | 61723282 | + | protein_coding | zf-C2H2 |
| ENSRNOG00000017477 | FALSE | TRUE | Mmp23 | 5 | 1.66E+08 | 1.66E+08 | - | protein_coding | NA |
| ENSRNOG00000057322 | FALSE | TRUE | Efna3 | 2 | 1.75E+08 | 1.75E+08 | - | protein_coding | NA |
| ENSRNOG00000007081 | FALSE | TRUE | Xdh | 6 | 21530113 | 21592268 | + | protein_coding | NA |
| ENSRNOG00000010691 | FALSE | TRUE | Cmtm3 | 19 | 604458 | 629790 | - | protein_coding | NA |
| ENSRNOG00000046764 | FALSE | TRUE | Tmprss11a | 14 | 21606547 | 21665279 | + | protein_coding | NA |
| ENSRNOG00000015830 | FALSE | TRUE | Ano9 | 1 | 1.96E+08 | 1.96E+08 | - | protein_coding | NA |
| ENSRNOG00000004743 | FALSE | TRUE | Cdcp1 | 8 | 1.23E+08 | 1.23E+08 | - | protein_coding | NA |
| ENSRNOG00000007606 | FALSE | TRUE | Rnf43 | 10 | 72464348 | 72536977 | + | protein_coding | NA |
| ENSRNOG00000013771 | FALSE | TRUE | Clca2 | 2 | 2.34E+08 | 2.34E+08 | - | protein_coding | NA |
| ENSRNOG00000023177 | FALSE | TRUE | Esrp2 | 19 | 34034559 | 34041726 | - | protein_coding | NA |
| ENSRNOG00000069758 | FALSE | TRUE | ENSRNOG00000069758 | 5 | 75890644 | 75897478 | - | lncRNA | NA |
| ENSRNOG00000013474 | FALSE | TRUE | Casz1 | 5 | 1.59E+08 | 1.59E+08 | + | protein_coding | Others |
| ENSRNOG00000016687 | FALSE | TRUE | Ssc5d | 1 | 68891949 | 68909743 | - | protein_coding | NA |
| ENSRNOG00000018679 | FALSE | TRUE | Rptn | 2 | 1.79E+08 | 1.79E+08 | + | protein_coding | NA |
| ENSRNOG00000054964 | FALSE | TRUE | Aoah | 17 | 43809638 | 44049148 | - | protein_coding | NA |
| ENSRNOG00000007284 | FALSE | TRUE | Slc2a1 | 5 | 1.33E+08 | 1.33E+08 | + | protein_coding | NA |
| ENSRNOG00000025001 | FALSE | TRUE | Pcolce | 12 | 19084210 | 19090508 | + | protein_coding | NA |
| ENSRNOG00000001519 | FALSE | TRUE | Dlx2 | 3 | 56370483 | 56373597 | - | protein_coding | Homeobox |
| ENSRNOG00000011800 | FALSE | TRUE | F3 | 2 | 2.1E+08 | 2.1E+08 | + | protein_coding | NA |
| ENSRNOG00000015321 | FALSE | TRUE | Moxd1 | 1 | 21075122 | 21161779 | - | protein_coding | NA |
| ENSRNOG00000009399 | FALSE | TRUE | Fads2b | 3 | 70535459 | 70575442 | - | protein_coding | NA |
| ENSRNOG00000070605 | FALSE | TRUE | Ly6g6c | 20 | 3753632 | 3758867 | - | protein_coding | NA |
| ENSRNOG00000004303 | FALSE | TRUE | Timp3 | 7 | 17521919 | 17571839 | - | protein_coding | NA |

| | | | | | | | | | |
|---|---|---|---|---|---|---|---|---|---|
| ENSRNOG00000011107 | FALSE | TRUE | Pcsk1 | 2 | 4395543 | 4442434 | + | protein_coding | NA |
| ENSRNOG00000007189 | FALSE | TRUE | Ttc22 | 5 | 1.21E+08 | 1.21E+08 | + | protein_coding | NA |
| ENSRNOG00000000940 | FALSE | TRUE | Flt1 | 12 | 7297292 | 7468626 | + | protein_coding | NA |
| ENSRNOG00000050209 | FALSE | TRUE | Fam240b | 17 | 657303 | 663206 | + | protein_coding | NA |
| ENSRNOG00000068138 | FALSE | TRUE | Aldh3b2 | 1 | 2.01E+08 | 2.01E+08 | + | protein_coding | NA |
| ENSRNOG00000060753 | FALSE | TRUE | Esyt1 | 7 | 928848 | 946199 | - | protein_coding | NA |
| ENSRNOG00000009970 | FALSE | TRUE | Gsdme | 4 | 79257804 | 79320806 | - | protein_coding | NA |
| ENSRNOG00000061883 | FALSE | TRUE | Aqp9 | 8 | 71797234 | 71837395 | - | protein_coding | NA |
| ENSRNOG00000010681 | FALSE | TRUE | Pitx2 | 2 | 2.18E+08 | 2.18E+08 | + | protein_coding | Homeobox |
| ENSRNOG00000021447 | FALSE | TRUE | Prr7 | 17 | 9165269 | 9172536 | - | protein_coding | NA |
| ENSRNOG00000018865 | FALSE | TRUE | Adamts12 | 2 | 60032873 | 60327629 | + | protein_coding | NA |
| ENSRNOG00000013172 | FALSE | TRUE | Nr1h3 | 3 | 77158808 | 77168722 | - | protein_coding | THR-like |
| ENSRNOG00000039858 | FALSE | TRUE | Mfsd12 | 7 | 8347314 | 8353988 | - | protein_coding | NA |
| ENSRNOG00000027988 | FALSE | TRUE | Zhx3 | 3 | 1.49E+08 | 1.5E+08 | - | protein_coding | Homeobox |
| ENSRNOG00000020150 | FALSE | TRUE | Il18bp | 1 | 1.56E+08 | 1.56E+08 | - | protein_coding | NA |
| ENSRNOG00000022845 | FALSE | TRUE | Cep70 | 8 | 99810367 | 99862855 | + | protein_coding | NA |
| ENSRNOG00000055293 | FALSE | TRUE | Ptprb | 7 | 51930015 | 52034748 | + | protein_coding | NA |
| ENSRNOG00000005776 | FALSE | TRUE | Bcl11b | 6 | 1.27E+08 | 1.27E+08 | - | protein_coding | zf-C2H2 |
| ENSRNOG00000001031 | FALSE | TRUE | Ocm | 12 | 10586897 | 10596707 | + | protein_coding | NA |
| ENSRNOG00000013069 | FALSE | TRUE | Sapcd2 | 3 | 8187266 | 8192546 | + | protein_coding | NA |
| ENSRNOG00000051970 | FALSE | TRUE | Aqp5 | 7 | 1.31E+08 | 1.31E+08 | + | protein_coding | NA |
| ENSRNOG00000004641 | FALSE | TRUE | Sstr4 | 3 | 1.36E+08 | 1.36E+08 | + | protein_coding | NA |
| ENSRNOG00000067971 | FALSE | TRUE | Sprn | 1 | 1.95E+08 | 1.95E+08 | - | protein_coding | NA |
| ENSRNOG00000016219 | FALSE | TRUE | Vnn1 | 1 | 21537094 | 21547395 | - | protein_coding | NA |
| ENSRNOG00000071073 | FALSE | TRUE | Sost | 10 | 86911517 | 86915561 | - | protein_coding | NA |
| ENSRNOG00000015072 | FALSE | TRUE | Ptgr1 | 5 | 73784009 | 73802666 | - | protein_coding | NA |
| ENSRNOG00000022313 | FALSE | TRUE | Klk9 | 1 | 94246339 | 94250631 | + | protein_coding | NA |
| ENSRNOG00000022911 | FALSE | TRUE | Hjurp | 9 | 88853386 | 88867728 | - | protein_coding | NA |
| ENSRNOG00000020655 | FALSE | TRUE | Ucn2 | 8 | 1.1E+08 | 1.1E+08 | + | protein_coding | NA |
| ENSRNOG00000027747 | FALSE | TRUE | Ninl | 3 | 1.4E+08 | 1.4E+08 | - | protein_coding | NA |
| ENSRNOG00000030285 | FALSE | TRUE | Epha3 | 11 | 1140985 | 1473900 | - | protein_coding | NA |
| ENSRNOG00000048449 | FALSE | TRUE | Itgb3 | 10 | 89509989 | 89564679 | + | protein_coding | NA |
| ENSRNOG00000012363 | FALSE | TRUE | Peli2 | 15 | 21393262 | 21535106 | + | protein_coding | NA |
| ENSRNOG00000030264 | FALSE | TRUE | Fam83h | 7 | 1.08E+08 | 1.08E+08 | - | protein_coding | NA |

| | | | | | | | | | |
|---|---|---|---|---|---|---|---|---|---|
| ENSRNOG00000025603 | FALSE | TRUE | Adgrf2 | 9 | 18219714 | 18269038 | + | protein_coding | NA |
| ENSRNOG00000025350 | FALSE | TRUE | Ppp1r13l | 1 | 79011745 | 79030712 | + | protein_coding | NA |
| ENSRNOG00000006302 | FALSE | TRUE | Gclc | 8 | 78630127 | 78668544 | + | protein_coding | NA |
| ENSRNOG00000004717 | FALSE | TRUE | Epyc | 7 | 32436620 | 32474135 | + | protein_coding | NA |
| ENSRNOG00000005034 | FALSE | TRUE | Arhgef25 | 7 | 63005940 | 63013719 | - | protein_coding | NA |
| ENSRNOG00000004642 | FALSE | TRUE | Lmx1a | 13 | 79835019 | 79978253 | + | protein_coding | Homeobox |
| ENSRNOG00000048389 | FALSE | TRUE | Fgf18 | 10 | 17706174 | 17736818 | - | protein_coding | NA |
| ENSRNOG00000012471 | FALSE | TRUE | Thbs4 | 2 | 23983158 | 24026313 | - | protein_coding | NA |
| ENSRNOG00000001195 | FALSE | TRUE | Trpv4 | 12 | 41938560 | 41977517 | + | protein_coding | NA |
| ENSRNOG00000010917 | FALSE | TRUE | Bmp5 | 8 | 76517164 | 76639925 | + | protein_coding | NA |
| ENSRNOG00000005602 | FALSE | TRUE | Mthfd1 | 6 | 94977862 | 95045372 | + | protein_coding | NA |
| ENSRNOG00000032231 | FALSE | TRUE | Mroh6 | 7 | 1.08E+08 | 1.08E+08 | - | protein_coding | NA |
| ENSRNOG00000033734 | FALSE | TRUE | Tnnt2 | 13 | 47267204 | 47285388 | + | protein_coding | NA |
| ENSRNOG00000009117 | FALSE | TRUE | Otub2 | 6 | 1.23E+08 | 1.23E+08 | + | protein_coding | NA |
| ENSRNOG00000062276 | FALSE | TRUE | AABR07044631.2 | 20 | 13043257 | 13071450 | - | protein_coding | NA |
| ENSRNOG00000019685 | FALSE | TRUE | Gdpd3 | 1 | 1.81E+08 | 1.81E+08 | + | protein_coding | NA |
| ENSRNOG00000007447 | FALSE | TRUE | Pla2g4b | 3 | 1.07E+08 | 1.07E+08 | + | protein_coding | NA |
| ENSRNOG00000011823 | FALSE | TRUE | Tfap2b | 9 | 21786258 | 21814520 | + | protein_coding | AP-2 |
| ENSRNOG00000010170 | FALSE | TRUE | Tubb4b | 3 | 8037799 | 8040296 | - | protein_coding | NA |
| ENSRNOG00000013894 | FALSE | TRUE | Gpr87 | 2 | 1.43E+08 | 1.43E+08 | - | protein_coding | NA |
| ENSRNOG00000060949 | FALSE | TRUE | Anxa8 | 16 | 9397113 | 9412043 | + | protein_coding | NA |
| ENSRNOG00000013177 | FALSE | TRUE | Map3k1 | 2 | 43350098 | 43414463 | - | protein_coding | NA |
| ENSRNOG00000027869 | FALSE | TRUE | Sox5 | 4 | 1.77E+08 | 1.78E+08 | - | protein_coding | HMG |
| ENSRNOG00000002176 | FALSE | TRUE | Nectin3 | 11 | 54364487 | 54462519 | + | protein_coding | NA |
| ENSRNOG00000028185 | FALSE | TRUE | Tstd3 | 5 | 35294645 | 35304101 | - | protein_coding | NA |
| ENSRNOG00000017954 | FALSE | TRUE | Prelid3a | 18 | 61017764 | 61031883 | + | protein_coding | NA |
| ENSRNOG00000005008 | FALSE | TRUE | Angpt4 | 3 | 1.4E+08 | 1.4E+08 | + | protein_coding | NA |
| ENSRNOG00000017707 | FALSE | TRUE | Abi2 | 9 | 61827139 | 61905699 | + | protein_coding | NA |
| ENSRNOG00000009691 | FALSE | TRUE | Lrrn2 | 13 | 44321364 | 44382482 | + | protein_coding | NA |
| ENSRNOG00000019999 | FALSE | TRUE | Lypd3 | 1 | 80226449 | 80230901 | + | protein_coding | NA |
| ENSRNOG00000069349 | FALSE | TRUE | ENSRNOG00000069349 | 3 | 1.57E+08 | 1.57E+08 | + | lncRNA | NA |
| ENSRNOG00000002680 | FALSE | TRUE | Lamc1 | 13 | 65374372 | 65501492 | - | protein_coding | NA |
| ENSRNOG00000000924 | FALSE | TRUE | Slc7a1 | 12 | 6628007 | 6703849 | + | protein_coding | NA |
| ENSRNOG00000010658 | FALSE | TRUE | Cth | 2 | 2.47E+08 | 2.47E+08 | - | protein_coding | NA |

| | | | | | | | | | |
|---|---|---|---|---|---|---|---|---|---|
| ENSRNOG00000008716 | FALSE | TRUE | Nefh | 14 | 79830362 | 79840351 | - | protein_coding | NA |
| ENSRNOG00000001520 | FALSE | TRUE | Dlx1 | 3 | 56356190 | 56360780 | + | protein_coding | Homeobox |
| ENSRNOG00000018266 | FALSE | TRUE | Slc23a3 | 9 | 76622800 | 76631366 | - | protein_coding | NA |
| ENSRNOG00000058618 | FALSE | TRUE | Dcst2 | 2 | 1.75E+08 | 1.75E+08 | + | protein_coding | NA |
| ENSRNOG00000005695 | FALSE | TRUE | Mgp | 4 | 1.7E+08 | 1.7E+08 | - | protein_coding | NA |
| ENSRNOG00000002461 | FALSE | TRUE | Nid1 | 17 | 86085077 | 86158267 | + | protein_coding | NA |
| ENSRNOG00000022772 | FALSE | TRUE | Prickle1 | 7 | 1.25E+08 | 1.25E+08 | - | protein_coding | NA |
| ENSRNOG00000020797 | FALSE | TRUE | She | 2 | 1.75E+08 | 1.75E+08 | + | protein_coding | NA |
| ENSRNOG00000017403 | FALSE | TRUE | Apobr | 1 | 1.81E+08 | 1.81E+08 | + | protein_coding | NA |
| ENSRNOG00000011292 | FALSE | TRUE | Col1a2 | 4 | 32563381 | 32598867 | + | protein_coding | NA |
| ENSRNOG00000027026 | FALSE | TRUE | Jazf1 | 4 | 81881061 | 82183100 | - | protein_coding | zf-C2H2 |
| ENSRNOG00000049351 | FALSE | TRUE | Nat8l | 14 | 76756077 | 76763411 | - | protein_coding | NA |
| ENSRNOG00000062811 | FALSE | TRUE | ENSRNOG00000062811 | 12 | 2189872 | 2196271 | + | protein_coding | NA |
| ENSRNOG00000033402 | FALSE | TRUE | Gsta6 | 9 | 23744288 | 23801131 | - | protein_coding | NA |
| ENSRNOG00000004936 | FALSE | TRUE | Sdc2 | 7 | 64127110 | 64241081 | + | protein_coding | NA |
| ENSRNOG00000016611 | FALSE | TRUE | Hras | 1 | 1.96E+08 | 1.96E+08 | - | protein_coding | NA |
| ENSRNOG00000001736 | FALSE | TRUE | Bdh1 | 11 | 69302534 | 69337671 | - | protein_coding | NA |
| ENSRNOG00000012206 | FALSE | TRUE | Dennd5a | 1 | 1.64E+08 | 1.64E+08 | - | protein_coding | NA |
| ENSRNOG00000018832 | FALSE | TRUE | RGD1564854 | 3 | 18154300 | 18173796 | + | protein_coding | NA |
| ENSRNOG0000002831 | FALSE | TRUE | Wfikkn2 | 10 | 79186647 | 79191657 | - | protein_coding | NA |
| ENSRNOG00000002540 | FALSE | TRUE | Rhoh | 14 | 42337751 | 42386369 | - | protein_coding | NA |
| ENSRNOG00000019770 | FALSE | TRUE | Bnc1 | 1 | 1.36E+08 | 1.36E+08 | - | protein_coding | zf-C2H2 |
| ENSRNOG00000060703 | FALSE | TRUE | Troap | 7 | 1.3E+08 | 1.3E+08 | + | protein_coding | NA |
| ENSRNOG00000063505 | FALSE | TRUE | Scn2b | 8 | 45425629 | 45437765 | + | protein_coding | NA |
| ENSRNOG00000002583 | FALSE | TRUE | Serpinb11 | 13 | 23304456 | 23344604 | + | protein_coding | NA |
| ENSRNOG00000021840 | FALSE | TRUE | Cped1 | 4 | 50516819 | 50788674 | + | protein_coding | NA |
| ENSRNOG00000049681 | FALSE | TRUE | AABR07031491.2 | 18 | 15029041 | 15168044 | + | protein_coding | NA |
| ENSRNOG00000013287 | FALSE | TRUE | Sall2 | 15 | 25021345 | 25038918 | - | protein_coding | zf-C2H2 |
| ENSRNOG00000033579 | FALSE | TRUE | Glt8d2 | 7 | 21029525 | 21081080 | + | protein_coding | NA |
| ENSRNOG00000018551 | FALSE | TRUE | Olr63 | 1 | 1.57E+08 | 1.57E+08 | + | protein_coding | NA |
| ENSRNOG00000013932 | FALSE | TRUE | Rbp2 | 8 | 99079104 | 99104473 | + | protein_coding | NA |
| ENSRNOG00000011758 | FALSE | TRUE | Fkbp7 | 3 | 61608557 | 61623280 | - | protein_coding | NA |
| ENSRNOG00000032261 | FALSE | TRUE | Cyp2d4 | 7 | 1.14E+08 | 1.14E+08 | - | protein_coding | NA |
| ENSRNOG00000007000 | FALSE | TRUE | Grhl2 | 7 | 68400477 | 68530258 | + | protein_coding | CP2 |

| | | | | | | | | | |
|---|---|---|---|---|---|---|---|---|---|
| ENSRNOG00000043093 | FALSE | TRUE | Ap1m2 | 8 | 19838580 | 19856482 | - | protein_coding | NA |
| ENSRNOG00000028982 | FALSE | TRUE | Mrgprb3 | 1 | 98272099 | 98286242 | - | protein_coding | NA |
| ENSRNOG00000010938 | FALSE | TRUE | Slc7a10 | 1 | 87829175 | 87845071 | + | protein_coding | NA |
| ENSRNOG00000009144 | FALSE | TRUE | Lad1 | 13 | 47249605 | 47263967 | + | protein_coding | NA |
| ENSRNOG00000066499 | FALSE | TRUE | C1H10orf95 | 1 | 2.45E+08 | 2.45E+08 | - | protein_coding | NA |
| ENSRNOG00000008668 | FALSE | TRUE | Vsig8 | 13 | 85023177 | 85031364 | + | protein_coding | NA |
| ENSRNOG00000004278 | FALSE | TRUE | Dlx3 | 10 | 80064489 | 80069872 | + | protein_coding | Homeobox |
| ENSRNOG00000021140 | FALSE | TRUE | Kcnk4 | 1 | 2.04E+08 | 2.04E+08 | - | protein_coding | NA |
| ENSRNOG00000052510 | FALSE | TRUE | Wnt10a | 9 | 76349931 | 76362400 | + | protein_coding | NA |
| ENSRNOG00000033153 | FALSE | TRUE | Sfn | 5 | 1.46E+08 | 1.46E+08 | - | protein_coding | NA |
| ENSRNOG00000003715 | FALSE | TRUE | Srpx2 | X | 97106561 | 97132195 | + | protein_coding | NA |
| ENSRNOG00000046112 | FALSE | TRUE | Dedd2 | 1 | 80792000 | 80807714 | - | protein_coding | NA |
| ENSRNOG00000019099 | FALSE | TRUE | AABR07054578.1 | 3 | 1.54E+08 | 1.54E+08 | - | protein_coding | zf-C2H2 |
| ENSRNOG00000008376 | FALSE | TRUE | Slc2a3 | 4 | 1.56E+08 | 1.56E+08 | - | protein_coding | NA |
| ENSRNOG00000051548 | FALSE | TRUE | Lmod1 | 13 | 46754033 | 46794900 | + | protein_coding | NA |
| ENSRNOG00000020058 | FALSE | TRUE | Celsr2 | 2 | 1.96E+08 | 1.96E+08 | - | protein_coding | NA |
| ENSRNOG00000015210 | FALSE | TRUE | Ggt6 | 10 | 57051825 | 57067260 | + | protein_coding | NA |
| ENSRNOG00000011859 | FALSE | TRUE | Eif5a2 | 2 | 1.12E+08 | 1.12E+08 | + | protein_coding | NA |
| ENSRNOG00000066580 | FALSE | TRUE | ENSRNOG00000066580 | 17 | 42522559 | 42529611 | - | lncRNA | NA |
| ENSRNOG00000005955 | FALSE | TRUE | Naalad2 | 8 | 15407043 | 15479714 | - | protein_coding | NA |
| ENSRNOG00000000728 | FALSE | TRUE | Clic2 | 20 | 148907 | 164355 | + | protein_coding | NA |
| ENSRNOG00000004772 | FALSE | TRUE | Cytip | 3 | 42698768 | 42725993 | - | protein_coding | NA |
| ENSRNOG00000028668 | FALSE | TRUE | Slc28a2 | 3 | 1.09E+08 | 1.09E+08 | + | protein_coding | NA |
| ENSRNOG00000005410 | FALSE | TRUE | Cdca7l | 6 | 1.39E+08 | 1.39E+08 | + | protein_coding | NA |
| ENSRNOG00000028630 | FALSE | TRUE | Ksr2 | 12 | 38802004 | 39188375 | - | protein_coding | NA |
| ENSRNOG00000023492 | FALSE | TRUE | Greb1l | 18 | 1392725 | 1628067 | + | protein_coding | NA |
| ENSRNOG00000029148 | FALSE | TRUE | Pdgfd | 8 | 3488423 | 3722395 | + | protein_coding | NA |
| ENSRNOG00000059790 | FALSE | TRUE | Krt78 | 7 | 1.33E+08 | 1.33E+08 | - | protein_coding | NA |
| ENSRNOG00000015594 | FALSE | TRUE | Rftn2 | 9 | 56630460 | 56707810 | - | protein_coding | NA |
| ENSRNOG00000027030 | FALSE | TRUE | Adm | 1 | 1.65E+08 | 1.65E+08 | + | protein_coding | NA |
| ENSRNOG00000038894 | FALSE | TRUE | AABR07072096.1 | 10 | 31185731 | 31220966 | + | protein_coding | NA |
| ENSRNOG00000022537 | FALSE | TRUE | E2f8 | 1 | 98565717 | 98584098 | - | protein_coding | E2F |
| ENSRNOG00000012802 | FALSE | TRUE | Tenm3 | 16 | 41252182 | 43978594 | + | protein_coding | NA |
| ENSRNOG00000016866 | FALSE | TRUE | Fhl2 | 9 | 45388981 | 45431192 | - | protein_coding | NA |

| | | | | | | | | | |
|---|---|---|---|---|---|---|---|---|---|
| ENSRNOG00000022239 | FALSE | TRUE | Trarg1 | 10 | 61521107 | 61541494 | + | protein_coding | NA |
| ENSRNOG00000016494 | FALSE | TRUE | Tmem204 | 10 | 14076519 | 14101920 | - | protein_coding | NA |
| ENSRNOG00000019708 | FALSE | TRUE | Ctsf | 1 | 2.02E+08 | 2.02E+08 | + | protein_coding | NA |
| ENSRNOG00000014372 | FALSE | TRUE | Gjb3 | 5 | 1.4E+08 | 1.4E+08 | - | protein_coding | NA |
| ENSRNOG00000018782 | FALSE | TRUE | Gmnn | 17 | 40301808 | 40310054 | + | protein_coding | NA |
| ENSRNOG00000034102 | FALSE | TRUE | Plk5 | 7 | 9346777 | 9355196 | - | protein_coding | NA |
| ENSRNOG00000063182 | FALSE | TRUE | ENSRNOG00000063182 | 1 | 80789105 | 80791212 | - | lncRNA | NA |
| ENSRNOG00000015503 | FALSE | TRUE | Otud7a | 1 | 1.17E+08 | 1.17E+08 | + | protein_coding | NA |
| ENSRNOG00000014182 | FALSE | TRUE | Tns1 | 9 | 75495814 | 75703225 | - | protein_coding | NA |
| ENSRNOG00000059519 | FALSE | TRUE | Bbox1 | 3 | 96822655 | 96871458 | - | protein_coding | NA |
| ENSRNOG00000015596 | FALSE | TRUE | Efhd1 | 9 | 87938284 | 87984917 | + | protein_coding | NA |
| ENSRNOG00000020908 | FALSE | TRUE | Hrob | 10 | 87206049 | 87222483 | + | protein_coding | NA |
| ENSRNOG00000015800 | FALSE | TRUE | Elmo3 | 19 | 33182036 | 33186410 | + | protein_coding | NA |
| ENSRNOG00000012791 | FALSE | TRUE | Ajuba | 15 | 28019778 | 28030021 | - | protein_coding | NA |
| ENSRNOG00000046361 | FALSE | TRUE | Hoxa7 | 4 | 81314625 | 81317659 | - | protein_coding | Homeobox |
| ENSRNOG00000011501 | FALSE | TRUE | Atp1b3 | 8 | 96910309 | 96941598 | - | protein_coding | NA |
| ENSRNOG00000010608 | FALSE | TRUE | Cep162 | 8 | 88149726 | 88206830 | - | protein_coding | NA |
| ENSRNOG00000055527 | FALSE | TRUE | Arap3 | 18 | 29807536 | 29833555 | - | protein_coding | NA |
| ENSRNOG00000021569 | FALSE | TRUE | Tiam1 | 11 | 29031348 | 29159901 | - | protein_coding | NA |
| ENSRNOG00000045558 | FALSE | TRUE | Cd34 | 13 | 1.06E+08 | 1.06E+08 | + | protein_coding | NA |
| ENSRNOG00000021390 | FALSE | TRUE | Meis3 | 1 | 76861924 | 76872691 | + | protein_coding | Homeobox |
| ENSRNOG00000012835 | FALSE | TRUE | Espl1 | 7 | 1.33E+08 | 1.33E+08 | + | protein_coding | NA |
| ENSRNOG00000004532 | FALSE | TRUE | Dipk1b | 3 | 9456409 | 9464161 | + | protein_coding | NA |
| ENSRNOG00000048161 | FALSE | TRUE | Tlr9 | 8 | 1.07E+08 | 1.07E+08 | + | protein_coding | NA |
| ENSRNOG00000068747 | FALSE | TRUE | ENSRNOG00000068747 | 18 | 27265615 | 27267685 | + | lncRNA | NA |
| ENSRNOG00000025167 | FALSE | TRUE | Sema4b | 1 | 1.34E+08 | 1.34E+08 | + | protein_coding | NA |
| ENSRNOG00000050190 | FALSE | TRUE | Eng | 3 | 15934518 | 15973230 | + | protein_coding | NA |
| ENSRNOG00000023576 | FALSE | TRUE | Ecrg4 | 9 | 45992954 | 46012595 | + | protein_coding | NA |
| ENSRNOG00000004699 | FALSE | TRUE | Fibin | 3 | 96942406 | 96944676 | - | protein_coding | NA |
| ENSRNOG00000064612 | FALSE | TRUE | Gcnt4 | 2 | 28252860 | 28283082 | + | protein_coding | NA |
| ENSRNOG00000053047 | FALSE | TRUE | Top2a | 10 | 83945735 | 83976874 | - | protein_coding | NA |
| ENSRNOG00000010516 | FALSE | TRUE | Plau | 15 | 3456232 | 3462775 | - | protein_coding | NA |
| ENSRNOG00000010158 | FALSE | TRUE | Magel2 | 1 | 1.16E+08 | 1.16E+08 | + | protein_coding | NA |
| ENSRNOG00000010265 | FALSE | TRUE | Ada | 3 | 1.52E+08 | 1.52E+08 | - | protein_coding | NA |

| | | | | | | | | | |
|---|---|---|---|---|---|---|---|---|---|
| ENSRNOG00000001314 | FALSE | TRUE | Fam20c | 12 | 15826871 | 15884543 | - | protein_coding | NA |
| ENSRNOG00000037456 | FALSE | TRUE | P2rx2 | 12 | 46339549 | 46342891 | + | protein_coding | NA |
| ENSRNOG00000000257 | FALSE | TRUE | Smpd3 | 19 | 34162341 | 34245749 | - | protein_coding | NA |
| ENSRNOG00000015380 | FALSE | TRUE | Jup | 10 | 85300440 | 85327057 | - | protein_coding | NA |
| ENSRNOG00000029049 | FALSE | TRUE | Zdhhc14 | 1 | 46069127 | 46330488 | + | protein_coding | NA |
| ENSRNOG00000062526 | FALSE | TRUE | Ankrd63 | 3 | 1.06E+08 | 1.06E+08 | - | protein_coding | NA |
| ENSRNOG00000005670 | FALSE | TRUE | Art4 | 4 | 1.7E+08 | 1.7E+08 | - | protein_coding | NA |
| ENSRNOG00000015741 | FALSE | TRUE | Slc2a13 | 7 | 1.22E+08 | 1.23E+08 | - | protein_coding | NA |
| ENSRNOG00000004794 | FALSE | TRUE | Rtn1 | 6 | 90763216 | 90983436 | - | protein_coding | NA |
| ENSRNOG00000019949 | FALSE | TRUE | Mrps12 | 1 | 84025899 | 84028780 | - | protein_coding | NA |
| ENSRNOG00000016021 | FALSE | TRUE | Lims2 | 18 | 23553937 | 23592137 | + | protein_coding | NA |
| ENSRNOG00000009589 | FALSE | TRUE | Angptl7 | 5 | 1.59E+08 | 1.59E+08 | - | protein_coding | NA |
| ENSRNOG00000024905 | FALSE | TRUE | Drc1 | 6 | 26025005 | 26059414 | - | protein_coding | NA |
| ENSRNOG00000024651 | FALSE | TRUE | Greb1 | 6 | 39440504 | 39581633 | - | protein_coding | NA |
| ENSRNOG00000019301 | FALSE | TRUE | Lipm | 1 | 2.32E+08 | 2.32E+08 | + | protein_coding | NA |
| ENSRNOG00000030151 | FALSE | TRUE | Thsd7a | 4 | 40460814 | 40899583 | - | protein_coding | NA |
| ENSRNOG00000040205 | FALSE | TRUE | Zcchc24 | 16 | 1274548 | 1326696 | - | protein_coding | NA |
| ENSRNOG00000015275 | FALSE | TRUE | Ska1 | 18 | 67794725 | 67805037 | - | protein_coding | NA |
| ENSRNOG00000057501 | FALSE | TRUE | Fam81a | 8 | 70763614 | 70821875 | - | protein_coding | NA |
| ENSRNOG00000066351 | FALSE | TRUE | Sap25 | 12 | 19055763 | 19076089 | - | protein_coding | NA |
| ENSRNOG00000007805 | FALSE | TRUE | Mybl2 | 3 | 1.52E+08 | 1.52E+08 | + | protein_coding | MYB |
| ENSRNOG00000011032 | FALSE | TRUE | Lhfpl2 | 2 | 25281901 | 25427950 | + | protein_coding | NA |
| ENSRNOG00000002146 | FALSE | TRUE | Pkd2 | 14 | 5237135 | 5280825 | - | protein_coding | NA |
| ENSRNOG00000005348 | FALSE | TRUE | Pamr1 | 3 | 88889046 | 88988606 | + | protein_coding | NA |
| ENSRNOG00000000596 | FALSE | TRUE | Fyn | 20 | 42766369 | 42959911 | + | protein_coding | NA |
| ENSRNOG00000010146 | FALSE | TRUE | Ndn | 1 | 1.16E+08 | 1.16E+08 | + | protein_coding | NA |
| ENSRNOG00000061160 | FALSE | TRUE | Htra4 | 16 | 67001605 | 67015074 | + | protein_coding | NA |
| ENSRNOG00000019412 | FALSE | TRUE | Rhbg | 2 | 1.74E+08 | 1.74E+08 | - | protein_coding | NA |
| ENSRNOG00000002730 | FALSE | TRUE | Rgs5 | 13 | 81836304 | 81885518 | + | protein_coding | NA |
| ENSRNOG00000004400 | FALSE | TRUE | Avpr1a | 7 | 58114284 | 58122215 | + | protein_coding | NA |
| ENSRNOG00000006726 | FALSE | TRUE | Zfp9 | 4 | 1.52E+08 | 1.52E+08 | - | protein_coding | zf-C2H2 |
| ENSRNOG00000017766 | FALSE | TRUE | Ca12 | 8 | 67274359 | 67330440 | + | protein_coding | NA |
| ENSRNOG00000025384 | FALSE | TRUE | Slc2a10 | 3 | 1.54E+08 | 1.54E+08 | + | protein_coding | NA |
| ENSRNOG00000033352 | FALSE | TRUE | S100a7a | 2 | 1.76E+08 | 1.76E+08 | + | protein_coding | NA |

| | | | | | | | | | |
|---|---|---|---|---|---|---|---|---|---|
| ENSRNOG00000057616 | FALSE | TRUE | Ptch2 | 5 | 1.31E+08 | 1.31E+08 | + | protein_coding | NA |
| ENSRNOG00000028996 | FALSE | TRUE | Krt1 | 7 | 1.33E+08 | 1.33E+08 | - | protein_coding | NA |
| ENSRNOG00000014436 | FALSE | TRUE | Ppip5k1 | 3 | 1.08E+08 | 1.08E+08 | - | protein_coding | NA |
| ENSRNOG00000018526 | FALSE | TRUE | Dlg4 | 10 | 54739470 | 54767153 | + | protein_coding | NA |
| ENSRNOG00000006845 | FALSE | TRUE | Psapl1 | 14 | 74544646 | 74547206 | - | protein_coding | NA |
| ENSRNOG00000054052 | FALSE | TRUE | Ppp4r4 | 6 | 1.23E+08 | 1.23E+08 | + | protein_coding | NA |
| ENSRNOG00000001169 | FALSE | TRUE | Slc37a1 | 20 | 9378836 | 9433892 | + | protein_coding | NA |
| ENSRNOG00000013927 | FALSE | TRUE | Jag2 | 6 | 1.32E+08 | 1.32E+08 | - | protein_coding | NA |
| ENSRNOG00000003013 | FALSE | TRUE | Rpgr | X | 12566645 | 12747882 | + | protein_coding | NA |
| ENSRNOG00000017012 | FALSE | TRUE | Coq7 | 1 | 1.73E+08 | 1.73E+08 | - | protein_coding | NA |
| ENSRNOG00000017249 | FALSE | TRUE | Zfp366 | 2 | 30578715 | 30642182 | + | protein_coding | zf-C2H2 |
| ENSRNOG00000016776 | FALSE | TRUE | Extl1 | 5 | 1.47E+08 | 1.47E+08 | - | protein_coding | NA |
| ENSRNOG00000009465 | FALSE | TRUE | Sfrp2 | 2 | 1.69E+08 | 1.69E+08 | + | protein_coding | NA |
| ENSRNOG00000002174 | FALSE | TRUE | Cwh43 | 14 | 34754289 | 34800814 | - | protein_coding | NA |
| ENSRNOG00000008837 | FALSE | TRUE | Ass1 | 3 | 14747368 | 14796903 | + | protein_coding | NA |
| ENSRNOG00000011427 | FALSE | TRUE | Hr | 15 | 45626835 | 45646313 | + | protein_coding | NA |
| ENSRNOG00000011826 | FALSE | TRUE | Lzts1 | 16 | 20542809 | 20598203 | + | protein_coding | NA |
| ENSRNOG00000022760 | FALSE | TRUE | Rubcnl | 15 | 50332418 | 50400849 | + | protein_coding | NA |
| ENSRNOG00000061004 | FALSE | TRUE | Asphd2 | 12 | 44248145 | 44257772 | + | protein_coding | NA |
| ENSRNOG00000007879 | FALSE | TRUE | ENSRNOG00000007879 | X | 1.3E+08 | 1.3E+08 | + | protein_coding | NA |
| ENSRNOG00000004712 | FALSE | TRUE | Angptl1 | 13 | 68962991 | 68992570 | + | protein_coding | NA |
| ENSRNOG00000025031 | FALSE | TRUE | RGD1560559 | 11 | 79989077 | 79989961 | - | protein_coding | NA |
| ENSRNOG00000033110 | FALSE | TRUE | Svep1 | 5 | 72811410 | 72988525 | - | protein_coding | NA |
| ENSRNOG00000011680 | FALSE | TRUE | Il16 | 1 | 1.38E+08 | 1.38E+08 | - | protein_coding | NA |
| ENSRNOG00000063113 | FALSE | TRUE | Mup4 | 5 | 74842319 | 75061474 | - | protein_coding | NA |
| ENSRNOG00000056550 | FALSE | TRUE | Epb41l4b | 5 | 71694331 | 71851990 | - | protein_coding | NA |
| ENSRNOG00000058057 | FALSE | TRUE | AABR07019403.1 | 15 | 96074564 | 96237806 | - | protein_coding | NA |
| ENSRNOG00000050453 | FALSE | TRUE | AABR07002848.1 | 1 | 83965986 | 83987778 | + | protein_coding | NA |
| ENSRNOG00000060614 | FALSE | TRUE | Pxdn | 6 | 46580761 | 46658345 | + | protein_coding | NA |
| ENSRNOG00000010161 | FALSE | TRUE | Myo10 | 2 | 76100987 | 76303030 | + | protein_coding | NA |
| ENSRNOG00000002396 | FALSE | TRUE | Serpinb8 | 13 | 23626945 | 23650835 | + | protein_coding | NA |
| ENSRNOG00000042455 | FALSE | TRUE | Tlr12 | 5 | 1.41E+08 | 1.41E+08 | - | protein_coding | NA |
| ENSRNOG00000009149 | FALSE | TRUE | Jam3 | 8 | 25507057 | 25569355 | - | protein_coding | NA |
| ENSRNOG00000055934 | FALSE | TRUE | Dmkn | 1 | 86014188 | 86030006 | + | protein_coding | NA |

| | | | | | | | | | |
|---|---|---|---|---|---|---|---|---|---|
| ENSRNOG00000016479 | FALSE | TRUE | Plekhg4 | 19 | 33249706 | 33266357 | + | protein_coding | NA |
| ENSRNOG00000004245 | FALSE | TRUE | Mgst3 | 13 | 79526541 | 79547411 | - | protein_coding | NA |
| ENSRNOG00000003800 | FALSE | TRUE | Rgs9 | 10 | 94197054 | 94270892 | - | protein_coding | NA |
| ENSRNOG00000061876 | FALSE | TRUE | Aldh4a1 | 5 | 1.52E+08 | 1.52E+08 | + | protein_coding | NA |
| ENSRNOG00000061346 | FALSE | TRUE | B3glct | 12 | 5255740 | 5346810 | - | protein_coding | NA |
| ENSRNOG00000002947 | FALSE | TRUE | Dpt | 13 | 77123115 | 77151639 | + | protein_coding | NA |
| ENSRNOG00000020783 | FALSE | TRUE | Ntf4 | 1 | 95893457 | 95897243 | + | protein_coding | NA |
| ENSRNOG00000002800 | FALSE | TRUE | Gdpd2 | X | 65826574 | 65835361 | + | protein_coding | NA |
| ENSRNOG00000054989 | FALSE | TRUE | Grhl1 | 6 | 41207348 | 41254301 | + | protein_coding | CP2 |
| ENSRNOG00000028247 | FALSE | TRUE | Ankrd13d | 1 | 2.02E+08 | 2.02E+08 | - | protein_coding | NA |
| ENSRNOG00000020119 | FALSE | TRUE | Pcdhac2 | 18 | 28581225 | 28846211 | + | protein_coding | NA |
| ENSRNOG00000012843 | FALSE | TRUE | Aspg | 6 | 1.31E+08 | 1.31E+08 | + | protein_coding | NA |
| ENSRNOG00000058645 | FALSE | TRUE | Tnc | 5 | 77375851 | 77460624 | - | protein_coding | NA |
| ENSRNOG00000007364 | FALSE | TRUE | Rab15 | 6 | 95499587 | 95523942 | - | protein_coding | NA |
| ENSRNOG00000039976 | FALSE | TRUE | Ptk7 | 9 | 14351202 | 14418494 | + | protein_coding | NA |
| ENSRNOG00000019372 | FALSE | TRUE | Pc | 1 | 2.02E+08 | 2.02E+08 | + | protein_coding | NA |
| ENSRNOG00000012051 | FALSE | TRUE | Ncaph | 3 | 1.14E+08 | 1.14E+08 | - | protein_coding | NA |
| ENSRNOG00000017359 | FALSE | TRUE | Itm2c | 9 | 86545921 | 86559742 | + | protein_coding | NA |
| ENSRNOG00000053735 | FALSE | TRUE | Hebp2 | 1 | 13194982 | 13201777 | - | protein_coding | NA |
| ENSRNOG00000057256 | FALSE | TRUE | Slc19a3 | 9 | 84277024 | 84299337 | - | protein_coding | NA |
| ENSRNOG00000001957 | FALSE | TRUE | Sult1e1 | 14 | 20422324 | 20439275 | + | protein_coding | NA |
| ENSRNOG00000010461 | FALSE | TRUE | Gpx8 | 2 | 44676454 | 44680195 | - | protein_coding | NA |
| ENSRNOG00000015093 | FALSE | TRUE | Sparcl1 | 14 | 5632569 | 5663865 | + | protein_coding | NA |
| ENSRNOG00000069330 | FALSE | TRUE | Ackr1 | 13 | 85782398 | 85783983 | - | protein_coding | NA |
| ENSRNOG00000003864 | FALSE | TRUE | Gipc1 | 19 | 24453123 | 24486997 | - | protein_coding | NA |
| ENSRNOG00000002418 | FALSE | TRUE | Tgfb2 | 13 | 98160087 | 98261405 | - | protein_coding | NA |
| ENSRNOG00000002607 | FALSE | TRUE | Sox9 | 10 | 97806485 | 97811994 | + | protein_coding | HMG |
| ENSRNOG00000016997 | FALSE | TRUE | Bnip5 | 20 | 6956201 | 6975091 | - | protein_coding | NA |
| ENSRNOG00000007925 | FALSE | TRUE | Pak6 | 3 | 1.06E+08 | 1.06E+08 | + | protein_coding | NA |
| ENSRNOG00000005944 | FALSE | TRUE | Slurp1 | 7 | 1.07E+08 | 1.07E+08 | - | protein_coding | NA |
| ENSRNOG00000070043 | FALSE | TRUE | Ces2g | 19 | 32946748 | 32954337 | + | protein_coding | NA |
| ENSRNOG00000012815 | FALSE | TRUE | Chst10 | 9 | 41092808 | 41122503 | - | protein_coding | NA |
| ENSRNOG00000022428 | FALSE | TRUE | Flrt1 | 1 | 2.04E+08 | 2.04E+08 | - | protein_coding | NA |
| ENSRNOG00000055530 | FALSE | TRUE | Panx2 | 7 | 1.2E+08 | 1.2E+08 | + | protein_coding | NA |

| | | | | | | | | | |
|---|---|---|---|---|---|---|---|---|---|
| ENSRNOG00000009118 | FALSE | TRUE | Pou2f3 | 8 | 43495527 | 43577795 | - | protein_coding | Pou |
| ENSRNOG00000004275 | FALSE | TRUE | Egfl6 | X | 27884125 | 27942044 | + | protein_coding | NA |
| ENSRNOG00000054506 | FALSE | TRUE | Nccrp1 | 1 | 83835083 | 83838676 | - | protein_coding | NA |
| ENSRNOG00000015476 | FALSE | TRUE | Ubxn11 | 5 | 1.46E+08 | 1.46E+08 | + | protein_coding | NA |
| ENSRNOG00000062881 | FALSE | TRUE | ENSRNOG00000062881 | 11 | 55680830 | 55684302 | - | protein_coding | NA |
| ENSRNOG00000005743 | FALSE | TRUE | Dapl1 | 3 | 43960810 | 43980940 | + | protein_coding | NA |
| ENSRNOG00000022060 | FALSE | TRUE | Magi1 | 4 | 1.26E+08 | 1.27E+08 | - | protein_coding | NA |
| ENSRNOG00000024000 | FALSE | TRUE | Cd22 | 1 | 86117459 | 86132322 | - | protein_coding | NA |
| ENSRNOG00000023679 | FALSE | TRUE | Prdm5 | 4 | 95075768 | 95238301 | + | protein_coding | zf-C2H2 |
| ENSRNOG00000021103 | FALSE | TRUE | Nrxn2 | 1 | 2.04E+08 | 2.04E+08 | + | protein_coding | NA |
| ENSRNOG00000021285 | FALSE | TRUE | Celsr1 | 7 | 1.17E+08 | 1.17E+08 | - | protein_coding | NA |
| ENSRNOG00000016308 | FALSE | TRUE | Il10ra | 8 | 45563137 | 45578061 | - | protein_coding | NA |
| ENSRNOG00000045670 | FALSE | TRUE | Tnfsf12 | 10 | 54403870 | 54413213 | - | protein_coding | NA |
| ENSRNOG00000006998 | FALSE | TRUE | Fhl4 | 7 | 18598103 | 18635184 | + | protein_coding | NA |
| ENSRNOG00000051905 | FALSE | TRUE | Wnt2 | 4 | 46333998 | 46374402 | - | protein_coding | NA |
| ENSRNOG00000065770 | FALSE | TRUE | Rmi2 | 10 | 4830553 | 4837235 | - | protein_coding | NA |
| ENSRNOG00000030101 | FALSE | TRUE | Traip | 8 | 1.09E+08 | 1.09E+08 | + | protein_coding | NA |
| ENSRNOG00000012546 | FALSE | TRUE | Frmpd1 | 5 | 59443076 | 59545080 | + | protein_coding | NA |
| ENSRNOG00000020731 | FALSE | TRUE | Ppfia3 | 1 | 95817110 | 95845798 | - | protein_coding | NA |
| ENSRNOG00000007567 | FALSE | TRUE | Rem1 | 3 | 1.41E+08 | 1.41E+08 | + | protein_coding | NA |
| ENSRNOG00000007906 | FALSE | TRUE | Bub1b | 3 | 1.06E+08 | 1.06E+08 | + | protein_coding | NA |
| ENSRNOG00000066331 | FALSE | TRUE | Il22ra1 | 5 | 1.48E+08 | 1.48E+08 | + | protein_coding | NA |
| ENSRNOG00000066670 | FALSE | TRUE | ENSRNOG00000066670 | X | 1.5E+08 | 1.5E+08 | + | protein_coding | NA |
| ENSRNOG00000027595 | FALSE | TRUE | Tfap2e | 5 | 1.39E+08 | 1.39E+08 | - | protein_coding | AP-2 |
| ENSRNOG00000012278 | FALSE | TRUE | Fgf10 | 2 | 50800992 | 50876866 | + | protein_coding | NA |
| ENSRNOG00000009625 | FALSE | TRUE | Dpysl2 | 15 | 41005551 | 41111829 | - | protein_coding | NA |
| ENSRNOG00000018282 | FALSE | TRUE | Gda | 1 | 2.19E+08 | 2.19E+08 | - | protein_coding | NA |
| ENSRNOG00000021798 | FALSE | TRUE | Pglyrp4 | 2 | 1.76E+08 | 1.76E+08 | + | protein_coding | NA |
| ENSRNOG00000002449 | FALSE | TRUE | Maged2 | X | 19733597 | 19740477 | - | protein_coding | NA |
| ENSRNOG00000030210 | FALSE | TRUE | Fndc1 | 1 | 47281844 | 47364259 | + | protein_coding | NA |
| ENSRNOG00000011568 | FALSE | TRUE | Rspo3 | 1 | 28283914 | 28367061 | + | protein_coding | NA |
| ENSRNOG00000023664 | FALSE | TRUE | Lepr | 5 | 1.16E+08 | 1.16E+08 | + | protein_coding | NA |
| ENSRNOG00000032446 | FALSE | TRUE | Recql4 | 7 | 1.08E+08 | 1.08E+08 | - | protein_coding | NA |
| ENSRNOG00000042729 | FALSE | TRUE | Efhc1 | 9 | 23375840 | 23414283 | + | protein_coding | NA |

| | | | | | | | | | |
|---|---|---|---|---|---|---|---|---|---|
| ENSRNOG00000004444 | FALSE | TRUE | Ikzf1 | 14 | 86255065 | 86342416 | + | protein_coding | zf-C2H2 |
| ENSRNOG00000013324 | FALSE | TRUE | Cdh5 | 19 | 815411 | 854368 | - | protein_coding | NA |
| ENSRNOG00000018951 | FALSE | TRUE | Col4a5 | X | 1.05E+08 | 1.05E+08 | + | protein_coding | NA |
| ENSRNOG00000015614 | FALSE | TRUE | Ppp1r16b | 3 | 1.47E+08 | 1.47E+08 | + | protein_coding | NA |
| ENSRNOG00000026490 | FALSE | TRUE | Dnm3 | 13 | 74359213 | 74838108 | - | protein_coding | NA |
| ENSRNOG00000004198 | FALSE | TRUE | Stxbp6 | 6 | 61920756 | 62158024 | - | protein_coding | NA |
| ENSRNOG00000000858 | FALSE | TRUE | Sapcd1 | 20 | 3793967 | 3795790 | + | protein_coding | NA |
| ENSRNOG00000002149 | FALSE | TRUE | Nkx6-1 | 14 | 7969756 | 7976681 | + | protein_coding | Homeobox |
| ENSRNOG00000011923 | FALSE | TRUE | Mgarp | 2 | 1.35E+08 | 1.35E+08 | - | protein_coding | NA |
| ENSRNOG00000056297 | FALSE | TRUE | Fmnl3 | 7 | 1.3E+08 | 1.31E+08 | - | protein_coding | NA |
| ENSRNOG00000006831 | FALSE | TRUE | Pgr | 8 | 6072673 | 6131344 | + | protein_coding | ESR-like |
| ENSRNOG00000028624 | FALSE | TRUE | Kif26b | 13 | 90283404 | 90682811 | + | protein_coding | NA |
| ENSRNOG00000023708 | FALSE | TRUE | Tmem176a | 4 | 77774843 | 77778620 | + | protein_coding | NA |
| ENSRNOG00000009867 | FALSE | TRUE | Tgfb3 | 6 | 1.06E+08 | 1.06E+08 | - | protein_coding | NA |
| ENSRNOG00000018355 | FALSE | TRUE | Msx2 | 17 | 11097103 | 11102879 | + | protein_coding | Homeobox |
| ENSRNOG00000020837 | FALSE | TRUE | Cd300lg | 10 | 86991921 | 87008733 | + | protein_coding | NA |
| ENSRNOG00000013167 | FALSE | TRUE | Hmgb2l1 | 16 | 32710658 | 32713140 | - | protein_coding | HMG |
| ENSRNOG00000006251 | FALSE | TRUE | Fam83a | 7 | 89522826 | 89547388 | + | protein_coding | NA |
| ENSRNOG00000020776 | FALSE | TRUE | Dhcr7 | 1 | 1.99E+08 | 1.99E+08 | + | protein_coding | NA |
| ENSRNOG00000059947 | FALSE | TRUE | Sdc1 | 6 | 31562739 | 31585264 | + | protein_coding | NA |
| ENSRNOG00000026504 | FALSE | TRUE | Fam114a1 | 14 | 43275701 | 43346174 | - | protein_coding | NA |
| ENSRNOG00000002640 | FALSE | TRUE | Serpinb5 | 13 | 22985557 | 23005756 | + | protein_coding | NA |
| ENSRNOG00000053550 | FALSE | TRUE | Itga1 | 2 | 46653193 | 46812238 | - | protein_coding | NA |
| ENSRNOG00000003929 | FALSE | TRUE | Pcdh19 | X | 96771947 | 96873524 | - | protein_coding | NA |
| ENSRNOG00000032339 | FALSE | TRUE | Rasl12 | 8 | 65917840 | 65932741 | + | protein_coding | NA |
| ENSRNOG00000001478 | FALSE | TRUE | Gtf2ird1 | 12 | 22254221 | 22361040 | + | protein_coding | GTF2I |
| ENSRNOG00000024433 | FALSE | TRUE | Fbxl7 | 2 | 77140826 | 77509223 | - | protein_coding | NA |
| ENSRNOG00000018815 | FALSE | TRUE | Plk1 | 1 | 1.77E+08 | 1.77E+08 | + | protein_coding | NA |
| ENSRNOG00000020792 | FALSE | TRUE | Etv4 | 10 | 86706749 | 86721974 | - | protein_coding | ETS |
| ENSRNOG00000066359 | FALSE | TRUE | ENSRNOG00000066359 | 14 | 98025965 | 98031937 | - | protein_coding | NA |
| ENSRNOG00000006331 | FALSE | TRUE | Elovl5 | 8 | 78790846 | 78857284 | + | protein_coding | NA |
| ENSRNOG00000022268 | FALSE | TRUE | Pnpla3 | 7 | 1.15E+08 | 1.15E+08 | + | protein_coding | NA |
| ENSRNOG00000021656 | FALSE | TRUE | RGD1359334 | 2 | 1.83E+08 | 1.83E+08 | - | protein_coding | NA |
| ENSRNOG00000008871 | FALSE | TRUE | Sdr16c5 | 5 | 17018813 | 17040970 | - | protein_coding | NA |

| | | | | | | | | | |
|---|---|---|---|---|---|---|---|---|---|
| ENSRNOG00000019609 | FALSE | TRUE | B4galt2 | 5 | 1.31E+08 | 1.31E+08 | - | protein_coding | NA |
| ENSRNOG00000004399 | FALSE | TRUE | Dlx4 | 10 | 80085465 | 80090456 | - | protein_coding | Homeobox |
| ENSRNOG00000065509 | FALSE | TRUE | Frmpd3 | X | 1.04E+08 | 1.04E+08 | + | protein_coding | NA |
| ENSRNOG00000005715 | FALSE | TRUE | Lgr4 | 3 | 96447858 | 96548899 | + | protein_coding | NA |
| ENSRNOG00000006731 | FALSE | TRUE | Spc25 | 3 | 53975704 | 53988959 | - | protein_coding | NA |
| ENSRNOG00000064322 | FALSE | TRUE | Hoxa9 | 4 | 81323382 | 81326358 | - | protein_coding | Homeobox |
| ENSRNOG00000004057 | FALSE | TRUE | Ccdc88a | 14 | 1.03E+08 | 1.03E+08 | + | protein_coding | NA |
| ENSRNOG00000049425 | FALSE | TRUE | Fam167b | 5 | 1.42E+08 | 1.42E+08 | - | protein_coding | NA |
| ENSRNOG00000020444 | FALSE | TRUE | Hcn3 | 2 | 1.75E+08 | 1.75E+08 | - | protein_coding | NA |
| ENSRNOG00000027831 | FALSE | TRUE | Map7d3 | X | 1.35E+08 | 1.35E+08 | - | protein_coding | NA |
| ENSRNOG00000009886 | FALSE | TRUE | Fkbp14 | 4 | 83705652 | 83721528 | - | protein_coding | NA |
| ENSRNOG00000007799 | FALSE | TRUE | Fam151a | 5 | 1.22E+08 | 1.22E+08 | + | protein_coding | NA |
| ENSRNOG00000063508 | FALSE | TRUE | ENSRNOG00000063508 | 17 | 45622444 | 45643353 | - | protein_coding | NA |
| ENSRNOG00000022499 | FALSE | TRUE | Sgo1 | 9 | 6672123 | 6687805 | - | protein_coding | NA |
| ENSRNOG00000003897 | FALSE | TRUE | Col1a1 | 10 | 79883622 | 79900624 | + | protein_coding | NA |
| ENSRNOG00000020530 | FALSE | TRUE | Cnfn | 1 | 80951600 | 80953747 | - | protein_coding | NA |
| ENSRNOG00000067254 | FALSE | TRUE | ENSRNOG00000067254 | 17 | 13570612 | 13575873 | - | lncRNA | NA |
| ENSRNOG00000002773 | FALSE | TRUE | Rgs4 | 13 | 81936775 | 81943068 | - | protein_coding | NA |
| ENSRNOG00000004110 | FALSE | TRUE | Trib2 | 6 | 38545975 | 38569370 | - | protein_coding | NA |
| ENSRNOG00000009514 | FALSE | TRUE | Mme | 2 | 1.48E+08 | 1.48E+08 | + | protein_coding | NA |
| ENSRNOG00000014070 | FALSE | TRUE | Krt13 | 10 | 85051889 | 85056084 | - | protein_coding | NA |
| ENSRNOG00000002093 | FALSE | TRUE | Tgfbr3 | 14 | 2489397 | 2663341 | + | protein_coding | NA |
| ENSRNOG00000008234 | FALSE | TRUE | Lrrc61 | 4 | 77503857 | 77524020 | + | protein_coding | NA |
| ENSRNOG00000039470 | FALSE | TRUE | AC131360.1 | 18 | 29195932 | 29207644 | + | protein_coding | NA |
| ENSRNOG00000020138 | FALSE | TRUE | Slc4a3 | 9 | 77037016 | 77049105 | + | protein_coding | NA |
| ENSRNOG00000049410 | FALSE | TRUE | Chrm3 | 17 | 60005202 | 60467278 | + | protein_coding | NA |
| ENSRNOG00000049530 | FALSE | TRUE | AABR07012054.1 | 2 | 1.7E+08 | 1.7E+08 | - | protein_coding | NA |
| ENSRNOG00000014426 | FALSE | TRUE | Lox | 18 | 45967343 | 46041477 | - | protein_coding | NA |
| ENSRNOG00000015465 | FALSE | TRUE | Cep72 | 1 | 29225361 | 29255271 | + | protein_coding | NA |
| ENSRNOG00000029449 | FALSE | TRUE | Ror1 | 5 | 1.15E+08 | 1.15E+08 | + | protein_coding | NA |
| ENSRNOG00000000034 | FALSE | TRUE | Nuak2 | 13 | 43753832 | 43770604 | + | protein_coding | NA |
| ENSRNOG00000007957 | FALSE | TRUE | Tspoap1 | 10 | 72560980 | 72586412 | + | protein_coding | NA |
| ENSRNOG00000022335 | FALSE | TRUE | Rad21l1 | 3 | 1.4E+08 | 1.4E+08 | - | protein_coding | NA |
| ENSRNOG00000013503 | FALSE | TRUE | Cdh24 | 15 | 28091637 | 28101340 | - | protein_coding | NA |

| | | | | | | | | | |
|---|---|---|---|---|---|---|---|---|---|
| ENSRNOG00000018858 | FALSE | TRUE | Myct1 | 1 | 42018137 | 42029410 | + | protein_coding | NA |
| ENSRNOG00000000955 | FALSE | TRUE | Lnx2 | 12 | 8005227 | 8070494 | + | protein_coding | NA |
| ENSRNOG00000023449 | FALSE | TRUE | Atg9b | 4 | 10786114 | 10793651 | + | protein_coding | NA |
| ENSRNOG00000056697 | FALSE | TRUE | Kcnab1 | 2 | 1.49E+08 | 1.5E+08 | + | protein_coding | NA |
| ENSRNOG00000011882 | FALSE | TRUE | Gab2 | 1 | 1.51E+08 | 1.52E+08 | + | protein_coding | NA |
| ENSRNOG00000070523 | FALSE | TRUE | Mxd3 | 17 | 9301399 | 9305157 | + | protein_coding | bHLH |
| ENSRNOG00000009468 | FALSE | TRUE | Il12a | 2 | 1.53E+08 | 1.53E+08 | + | protein_coding | NA |
| ENSRNOG00000014453 | FALSE | TRUE | Anxa5 | 2 | 1.19E+08 | 1.19E+08 | - | protein_coding | NA |
| ENSRNOG00000068754 | FALSE | TRUE | Cd244 | 13 | 84020095 | 84036100 | + | protein_coding | NA |
| ENSRNOG00000069239 | FALSE | TRUE | Slc22a21 | 10 | 38104122 | 38130671 | - | protein_coding | NA |
| ENSRNOG00000000824 | FALSE | TRUE | Dse | 20 | 26118196 | 26196992 | - | protein_coding | NA |
| ENSRNOG00000036674 | FALSE | TRUE | Cd7 | 10 | 1.06E+08 | 1.06E+08 | - | protein_coding | NA |
| ENSRNOG00000055672 | FALSE | TRUE | Gpx2 | 6 | 95493589 | 95496877 | - | protein_coding | NA |
| ENSRNOG00000067843 | FALSE | TRUE | Olr1748 | 20 | 1435460 | 1441923 | + | protein_coding | NA |
| ENSRNOG00000057458 | FALSE | TRUE | Oip5 | 3 | 1.07E+08 | 1.07E+08 | - | protein_coding | NA |
| ENSRNOG00000028426 | FALSE | TRUE | Mcf2l | 16 | 76507133 | 76652733 | - | protein_coding | NA |
| ENSRNOG00000004783 | FALSE | TRUE | Fam171b | 3 | 68930822 | 68986127 | + | protein_coding | NA |
| ENSRNOG00000066973 | FALSE | TRUE | Kctd12 | 15 | 79801191 | 79806282 | - | protein_coding | NA |
| ENSRNOG00000008479 | FALSE | TRUE | Slc8a1 | 6 | 13194662 | 13535628 | + | protein_coding | NA |
| ENSRNOG00000021437 | FALSE | TRUE | AABR07073181.1 | 5 | 1.5E+08 | 1.5E+08 | + | protein_coding | NA |
| ENSRNOG00000069674 | FALSE | TRUE | ENSRNOG00000069674 | 10 | 54884768 | 54921688 | - | lncRNA | NA |
| ENSRNOG00000067655 | FALSE | TRUE | Aldoa | 1 | 1.81E+08 | 1.81E+08 | - | protein_coding | NA |
| ENSRNOG00000047300 | FALSE | TRUE | Bdkrb2 | 6 | 1.24E+08 | 1.25E+08 | + | protein_coding | NA |
| ENSRNOG00000033059 | FALSE | TRUE | Galnt6 | 7 | 1.32E+08 | 1.32E+08 | - | protein_coding | NA |
| ENSRNOG00000003388 | FALSE | TRUE | Cenpf | 13 | 1.01E+08 | 1.01E+08 | - | protein_coding | NA |
| ENSRNOG00000005038 | FALSE | TRUE | Ube2t | 13 | 46403375 | 46414835 | + | protein_coding | NA |
| ENSRNOG00000018785 | FALSE | TRUE | Slc16a13 | 10 | 54926760 | 54937788 | - | protein_coding | NA |
| ENSRNOG00000057957 | FALSE | TRUE | Crybg2 | 5 | 1.46E+08 | 1.46E+08 | + | protein_coding | NA |
| ENSRNOG00000051418 | FALSE | TRUE | RGD1310935 | 11 | 43483519 | 43526916 | + | protein_coding | NA |
| ENSRNOG00000017863 | FALSE | TRUE | Zeb1 | 17 | 51948948 | 52115214 | - | protein_coding | zf-C2H2 |
| ENSRNOG00000020295 | FALSE | TRUE | Plekhn1 | 5 | 1.67E+08 | 1.67E+08 | - | protein_coding | NA |
| ENSRNOG00000024972 | FALSE | TRUE | Cox10 | 10 | 48630676 | 48746667 | - | protein_coding | NA |
| ENSRNOG00000064843 | FALSE | TRUE | ENSRNOG00000064843 | 9 | 23419225 | 23422852 | + | lncRNA | NA |
| ENSRNOG00000023686 | FALSE | TRUE | Upk3b | 12 | 20631525 | 20637724 | - | protein_coding | NA |

| | | | | | | | | | |
|---|---|---|---|---|---|---|---|---|---|
| ENSRNOG00000009288 | FALSE | TRUE | Fgf14 | 15 | 1.01E+08 | 1.02E+08 | - | protein_coding | NA |
| ENSRNOG00000011986 | FALSE | TRUE | Krt12 | 10 | 84370883 | 84378045 | - | protein_coding | NA |
| ENSRNOG00000015578 | FALSE | TRUE | Tnk1 | 10 | 54571117 | 54579940 | - | protein_coding | NA |
| ENSRNOG00000024990 | FALSE | TRUE | Ppp1r1c | 3 | 64616700 | 64724104 | + | protein_coding | NA |
| ENSRNOG00000053728 | FALSE | TRUE | AABR07006724.1 | 1 | 2.28E+08 | 2.3E+08 | - | protein_coding | NA |
| ENSRNOG00000042918 | FALSE | TRUE | RGD1304770 | 20 | 43344601 | 43353061 | - | protein_coding | NA |
| ENSRNOG00000017878 | FALSE | TRUE | Aldh1a7 | 1 | 2.18E+08 | 2.18E+08 | - | protein_coding | NA |
| ENSRNOG00000020583 | FALSE | TRUE | Fcgrt | 1 | 95567175 | 95584084 | - | protein_coding | NA |
| ENSRNOG00000022012 | FALSE | TRUE | Tnfrsf18 | 5 | 1.67E+08 | 1.67E+08 | + | protein_coding | NA |
| ENSRNOG00000068537 | FALSE | TRUE | Hs3st1 | 14 | 71185682 | 71223248 | + | protein_coding | NA |
| ENSRNOG00000012318 | FALSE | TRUE | Aspm | 13 | 51074849 | 51123755 | + | protein_coding | NA |
| ENSRNOG00000045843 | FALSE | TRUE | Rusc2 | 5 | 57629904 | 57675524 | + | protein_coding | NA |
| ENSRNOG00000020669 | FALSE | TRUE | Ovol1 | 1 | 2.03E+08 | 2.03E+08 | - | protein_coding | zf-C2H2 |
| ENSRNOG00000001728 | FALSE | TRUE | Fam43a | 11 | 70128253 | 70131401 | - | protein_coding | NA |
| ENSRNOG00000008091 | FALSE | TRUE | Gins1 | 3 | 1.4E+08 | 1.4E+08 | + | protein_coding | NA |
| ENSRNOG00000029735 | FALSE | TRUE | Pid1 | 9 | 85284335 | 85503044 | - | protein_coding | NA |
| ENSRNOG00000042478 | FALSE | TRUE | Adam22 | 4 | 25715990 | 25964962 | + | protein_coding | NA |
| ENSRNOG00000005970 | FALSE | TRUE | Lrrc1 | 8 | 78239900 | 78406490 | - | protein_coding | NA |
| ENSRNOG00000007866 | FALSE | TRUE | Clec2dl1 | 4 | 1.62E+08 | 1.62E+08 | + | protein_coding | NA |
| ENSRNOG00000007837 | FALSE | TRUE | Acot11 | 5 | 1.22E+08 | 1.22E+08 | - | protein_coding | NA |
| ENSRNOG00000009388 | FALSE | TRUE | Sptssb | 2 | 1.54E+08 | 1.54E+08 | - | lncRNA | NA |
| ENSRNOG00000002810 | FALSE | TRUE | Gfpt2 | 10 | 34115052 | 34161329 | + | protein_coding | NA |
| ENSRNOG00000020953 | FALSE | TRUE | Ms4a7 | 1 | 2.08E+08 | 2.08E+08 | - | protein_coding | NA |
| ENSRNOG00000024711 | FALSE | TRUE | Sdk2 | 10 | 98828348 | 99101759 | - | protein_coding | NA |
| ENSRNOG00000001793 | FALSE | TRUE | Heg1 | 11 | 66957190 | 67095051 | - | protein_coding | NA |
| ENSRNOG00000033791 | FALSE | TRUE | Apc2 | 7 | 9392336 | 9414310 | - | protein_coding | NA |
| ENSRNOG00000004635 | FALSE | TRUE | Kera | 7 | 32397382 | 32404837 | + | protein_coding | NA |
| ENSRNOG00000014714 | FALSE | TRUE | Trpv6 | 4 | 70507348 | 70523017 | - | protein_coding | NA |
| ENSRNOG00000039489 | FALSE | TRUE | Tmem253 | 15 | 24706529 | 24708625 | + | protein_coding | NA |
| ENSRNOG00000018659 | FALSE | TRUE | Csf1 | 2 | 1.95E+08 | 1.95E+08 | - | protein_coding | NA |
| ENSRNOG00000011841 | FALSE | TRUE | Map2 | 9 | 67723371 | 67979809 | + | protein_coding | NA |
| ENSRNOG00000000275 | FALSE | TRUE | Fam13c | 20 | 18065628 | 18187592 | - | protein_coding | NA |
| ENSRNOG00000024429 | FALSE | TRUE | Peg12 | 1 | 1.16E+08 | 1.16E+08 | - | protein_coding | NA |
| ENSRNOG00000015354 | FALSE | TRUE | Aox1 | 9 | 59579649 | 59658770 | + | protein_coding | NA |

| | | | | | | | | | |
|---|---|---|---|---|---|---|---|---|---|
| ENSRNOG00000017123 | FALSE | TRUE | B2m | 3 | 1.09E+08 | 1.09E+08 | + | protein_coding | NA |
| ENSRNOG00000065741 | FALSE | TRUE | Il7r | 2 | 58454217 | 58477757 | - | protein_coding | NA |
| ENSRNOG00000065340 | FALSE | TRUE | ENSRNOG00000065340 | 7 | 1.32E+08 | 1.32E+08 | + | lncRNA | NA |
| ENSRNOG00000025597 | FALSE | TRUE | Rnase6 | 15 | 24354303 | 24357328 | + | protein_coding | NA |
| ENSRNOG00000060538 | FALSE | TRUE | Cdc42se1 | 2 | 1.83E+08 | 1.83E+08 | + | lncRNA | NA |
| ENSRNOG00000016102 | FALSE | TRUE | Ebf3 | 1 | 1.92E+08 | 1.92E+08 | - | protein_coding | COE |
| ENSRNOG00000021713 | FALSE | TRUE | Kif18b | 10 | 87870428 | 87889850 | - | protein_coding | NA |
| ENSRNOG00000016456 | FALSE | TRUE | Il33 | 1 | 2.28E+08 | 2.28E+08 | + | protein_coding | NA |
| ENSRNOG00000012062 | FALSE | TRUE | Npc2 | 6 | 1.04E+08 | 1.04E+08 | - | protein_coding | NA |
| ENSRNOG00000033658 | FALSE | TRUE | Kntc1 | 12 | 32769020 | 32839561 | - | protein_coding | NA |
| ENSRNOG00000063043 | FALSE | TRUE | Cbx4 | 10 | 1.04E+08 | 1.04E+08 | - | protein_coding | NA |
| ENSRNOG00000001618 | FALSE | TRUE | Ripk4 | 11 | 37122565 | 37144799 | - | protein_coding | NA |
| ENSRNOG00000008658 | FALSE | TRUE | Mitf | 4 | 1.3E+08 | 1.31E+08 | + | protein_coding | bHLH |
| ENSRNOG00000016853 | FALSE | TRUE | Dsg1 | 18 | 11674402 | 11703443 | + | protein_coding | NA |
| ENSRNOG00000037707 | FALSE | TRUE | Armcx6 | X | 97929041 | 97931977 | - | protein_coding | NA |
| ENSRNOG00000000562 | FALSE | TRUE | Prf1 | 20 | 29246202 | 29251701 | + | protein_coding | NA |
| ENSRNOG00000018029 | FALSE | TRUE | Doc2g | 1 | 2.01E+08 | 2.01E+08 | + | protein_coding | NA |
| ENSRNOG00000016512 | FALSE | TRUE | Sema3b | 8 | 1.08E+08 | 1.08E+08 | - | protein_coding | NA |
| ENSRNOG00000018113 | FALSE | TRUE | ENSRNOG00000018113 | 17 | 57986511 | 57989997 | - | protein_coding | NA |
| ENSRNOG00000011478 | FALSE | TRUE | Ackr4 | 8 | 1.05E+08 | 1.05E+08 | - | protein_coding | NA |
| ENSRNOG00000007110 | FALSE | TRUE | Ankrd6 | 5 | 47202873 | 47343090 | - | protein_coding | NA |
| ENSRNOG00000011631 | FALSE | TRUE | Fst | 2 | 46123439 | 46130571 | - | protein_coding | NA |
| ENSRNOG00000020774 | FALSE | TRUE | Zfp383 | 1 | 85132520 | 85143679 | + | protein_coding | zf-C2H2 |
| ENSRNOG00000014059 | FALSE | TRUE | Odf2l | 2 | 2.34E+08 | 2.34E+08 | + | protein_coding | NA |
| ENSRNOG00000017156 | FALSE | TRUE | Ankle1 | 16 | 18076492 | 18083729 | + | protein_coding | NA |
| ENSRNOG00000015088 | FALSE | TRUE | Prune2 | 1 | 2.14E+08 | 2.15E+08 | + | protein_coding | NA |
| ENSRNOG00000020485 | FALSE | TRUE | Vav3 | 2 | 1.97E+08 | 1.97E+08 | + | protein_coding | NA |
| ENSRNOG00000001249 | FALSE | TRUE | Col6a1 | 20 | 11905957 | 11924597 | + | protein_coding | NA |
| ENSRNOG00000026607 | FALSE | TRUE | Tnfsf18 | 13 | 73831252 | 73843169 | + | protein_coding | NA |
| ENSRNOG00000010643 | FALSE | TRUE | Kank2 | 8 | 20311676 | 20340900 | - | protein_coding | NA |
| ENSRNOG00000007177 | FALSE | TRUE | Poglut3 | 8 | 53777785 | 53795399 | + | protein_coding | NA |
| ENSRNOG00000025702 | FALSE | TRUE | Kdm2b | 12 | 33574657 | 33701355 | + | protein_coding | NA |
| ENSRNOG00000013774 | FALSE | TRUE | Lmnb1 | 18 | 50175874 | 50214502 | + | protein_coding | NA |
| ENSRNOG00000024874 | FALSE | TRUE | Prtfdc1 | 17 | 83548803 | 83655179 | - | protein_coding | NA |

| | | | | | | | | | |
|---|---|---|---|---|---|---|---|---|---|
| ENSRNOG00000062421 | FALSE | TRUE | RragB | X | 18184992 | 18234639 | - | protein_coding | NA |
| ENSRNOG00000020980 | FALSE | TRUE | AABR07006278.1 | 1 | 2.08E+08 | 2.08E+08 | + | protein_coding | NA |
| ENSRNOG00000018406 | FALSE | TRUE | Wipf1 | 3 | 58314542 | 58372742 | - | protein_coding | NA |
| ENSRNOG00000004928 | FALSE | TRUE | Sntg2 | 6 | 46801377 | 47003372 | - | protein_coding | NA |
| ENSRNOG00000027024 | FALSE | TRUE | Rgs16 | 13 | 65887530 | 65892857 | + | protein_coding | NA |
| ENSRNOG00000067538 | FALSE | TRUE | ENSRNOG00000067538 | 6 | 14081555 | 14149061 | - | lncRNA | NA |
| ENSRNOG00000042496 | FALSE | TRUE | Cyp4f5 | 7 | 11544082 | 11558978 | + | protein_coding | NA |
| ENSRNOG00000005158 | FALSE | TRUE | Slc24a5 | 3 | 1.12E+08 | 1.12E+08 | + | protein_coding | NA |
| ENSRNOG00000025581 | FALSE | TRUE | Vwa2 | 1 | 2.56E+08 | 2.56E+08 | + | protein_coding | NA |
| ENSRNOG00000024349 | FALSE | TRUE | Cbarp | 7 | 9566364 | 9575204 | + | protein_coding | NA |
| ENSRNOG00000056585 | FALSE | TRUE | Fscn1 | 12 | 11597048 | 11610211 | - | protein_coding | NA |
| ENSRNOG00000005678 | FALSE | TRUE | Lamb1 | 6 | 47835525 | 47902585 | + | protein_coding | NA |
| ENSRNOG00000013694 | FALSE | TRUE | Ntng2 | 3 | 12492639 | 12545890 | - | protein_coding | NA |
| ENSRNOG00000005115 | FALSE | TRUE | Asf1b | 19 | 24181028 | 24195549 | - | protein_coding | NA |
| ENSRNOG00000027042 | FALSE | TRUE | Srd5a2 | 6 | 21426215 | 21462112 | + | protein_coding | NA |
| ENSRNOG00000005996 | FALSE | TRUE | Lhx6 | 3 | 19433494 | 19456061 | - | protein_coding | Homeobox |
| ENSRNOG00000033234 | FALSE | TRUE | Ccrl2 | 8 | 1.11E+08 | 1.11E+08 | - | protein_coding | NA |
| ENSRNOG00000008737 | FALSE | TRUE | Tbc1d21 | 8 | 58761563 | 58773711 | - | protein_coding | NA |
| ENSRNOG00000014661 | FALSE | TRUE | Zfp385d | 15 | 5042572 | 5423367 | - | protein_coding | NA |
| ENSRNOG00000026579 | FALSE | TRUE | AC119332.1 | 1 | 2.02E+08 | 2.02E+08 | - | protein_coding | NA |
| ENSRNOG00000017646 | FALSE | TRUE | RGD1309651 | 19 | 48660288 | 48686349 | - | protein_coding | NA |
| ENSRNOG00000001684 | FALSE | TRUE | Ripply3 | 11 | 33648486 | 33656584 | + | protein_coding | NA |
| ENSRNOG00000002660 | FALSE | TRUE | Magee1 | X | 70189187 | 70192810 | + | protein_coding | NA |
| ENSRNOG00000062598 | FALSE | TRUE | ENSRNOG00000062598 | 18 | 61845577 | 61848438 | + | lncRNA | NA |
| ENSRNOG00000069735 | FALSE | TRUE | ENSRNOG00000069735 | 6 | 23600171 | 23614225 | + | lncRNA | NA |
| ENSRNOG00000026091 | FALSE | TRUE | Slc10a4 | 14 | 35197886 | 35203729 | - | protein_coding | NA |
| ENSRNOG00000004752 | FALSE | TRUE | Nalcn | 15 | 1E+08 | 1.01E+08 | - | protein_coding | NA |
| ENSRNOG00000018748 | FALSE | TRUE | Slc16a11 | 10 | 54927725 | 54942915 | + | protein_coding | NA |
| ENSRNOG00000031515 | FALSE | TRUE | Klra2 | 4 | 1.65E+08 | 1.65E+08 | - | protein_coding | NA |
| ENSRNOG00000023116 | FALSE | TRUE | Agmo | 6 | 53986292 | 54317623 | + | protein_coding | NA |
| ENSRNOG00000063706 | FALSE | TRUE | ENSRNOG00000063706 | 2 | 54159664 | 54165214 | - | lncRNA | NA |
| ENSRNOG00000016831 | FALSE | TRUE | Serpinh1 | 1 | 1.54E+08 | 1.54E+08 | - | protein_coding | NA |
| ENSRNOG00000001258 | FALSE | TRUE | Snx8 | 12 | 14251859 | 14300432 | + | protein_coding | NA |
| ENSRNOG00000018003 | FALSE | TRUE | F2rl1 | 2 | 26772278 | 26785226 | - | protein_coding | NA |

| | | | | | | | | | |
|---|---|---|---|---|---|---|---|---|---|
| ENSRNOG00000005659 | FALSE | TRUE | Aurkb | 10 | 53745142 | 53750837 | + | protein_coding | NA |
| ENSRNOG00000020382 | FALSE | TRUE | Ltb4r2 | 15 | 29259240 | 29260316 | + | protein_coding | NA |
| ENSRNOG00000002358 | FALSE | TRUE | Scpep1 | 10 | 73703278 | 73732850 | - | protein_coding | NA |
| ENSRNOG00000024178 | FALSE | TRUE | Cenpt | 19 | 33734685 | 33741142 | - | protein_coding | NA |
| ENSRNOG00000011154 | FALSE | TRUE | Adgrf5 | 9 | 17520009 | 17623968 | - | protein_coding | NA |
| ENSRNOG00000021244 | FALSE | TRUE | Hspa12b | 3 | 1.18E+08 | 1.18E+08 | + | protein_coding | NA |
| ENSRNOG00000060970 | FALSE | TRUE | Tnmd | X | 97057137 | 97072634 | + | protein_coding | NA |
| ENSRNOG00000010905 | FALSE | TRUE | Dlx5 | 4 | 34999139 | 35003407 | - | protein_coding | Homeobox |
| ENSRNOG00000038228 | FALSE | TRUE | Serp2 | 15 | 51879877 | 51904785 | - | protein_coding | NA |
| ENSRNOG00000006723 | FALSE | TRUE | Itga11 | 8 | 63146001 | 63254407 | + | protein_coding | NA |
| ENSRNOG00000006403 | FALSE | TRUE | Nectin1 | 8 | 44101776 | 44189787 | + | protein_coding | NA |
| ENSRNOG00000018674 | FALSE | TRUE | Ntrk3 | 1 | 1.32E+08 | 1.33E+08 | - | protein_coding | NA |
| ENSRNOG00000011548 | FALSE | TRUE | Ebf2 | 15 | 41435509 | 41634403 | + | protein_coding | COE |
| ENSRNOG00000069303 | FALSE | TRUE | Sh3bgrl | X | 74166871 | 74263783 | + | protein_coding | NA |
| ENSRNOG00000060879 | FALSE | TRUE | 1500009L16Rik | 7 | 20015102 | 20057975 | - | protein_coding | NA |
| ENSRNOG00000007307 | FALSE | TRUE | Syde1 | 7 | 11032898 | 11039869 | + | protein_coding | NA |
| ENSRNOG00000020860 | FALSE | TRUE | Tdrkh | 2 | 1.82E+08 | 1.82E+08 | + | protein_coding | NA |
| ENSRNOG00000023262 | FALSE | TRUE | Cenpm | 7 | 1.14E+08 | 1.14E+08 | - | protein_coding | NA |
| ENSRNOG00000001424 | FALSE | TRUE | Cux1 | 12 | 20107311 | 20425866 | + | protein_coding | CUT |
| ENSRNOG00000015986 | FALSE | TRUE | Rassf8 | 4 | 1.79E+08 | 1.79E+08 | + | protein_coding | NA |
| ENSRNOG00000022353 | FALSE | TRUE | Klk13 | 1 | 94165381 | 94177002 | + | protein_coding | NA |
| ENSRNOG00000063970 | FALSE | TRUE | Tprg1 | 11 | 75322470 | 75449907 | - | protein_coding | NA |
| ENSRNOG00000006957 | FALSE | TRUE | Gria4 | 8 | 1562119 | 2034979 | - | protein_coding | NA |
| ENSRNOG00000004049 | FALSE | TRUE | Baiap2 | 10 | 1.05E+08 | 1.05E+08 | + | protein_coding | NA |
| ENSRNOG00000005506 | FALSE | TRUE | Arhgef5 | 4 | 72087247 | 72111254 | + | protein_coding | NA |
| ENSRNOG00000014230 | FALSE | TRUE | Map1a | 3 | 1.08E+08 | 1.08E+08 | + | protein_coding | NA |
| ENSRNOG00000015529 | FALSE | TRUE | Cdca3 | 4 | 1.58E+08 | 1.58E+08 | + | protein_coding | NA |
| ENSRNOG00000000521 | FALSE | TRUE | Cdkn1a | 20 | 7149217 | 7159585 | + | protein_coding | NA |
| ENSRNOG00000009797 | FALSE | TRUE | Aqp3 | 5 | 56239201 | 56244720 | - | protein_coding | NA |
| ENSRNOG00000065225 | FALSE | TRUE | ENSRNOG00000065225 | 10 | 54918137 | 54926637 | + | lncRNA | NA |
| ENSRNOG00000020385 | FALSE | TRUE | Fads3 | 1 | 2.07E+08 | 2.07E+08 | + | protein_coding | NA |
| ENSRNOG00000021497 | FALSE | TRUE | Akt3 | 13 | 88946091 | 89225708 | - | protein_coding | NA |
| ENSRNOG00000002050 | FALSE | TRUE | Igfbp7 | 14 | 30737164 | 30797314 | + | protein_coding | NA |
| ENSRNOG00000005096 | FALSE | TRUE | Bzw2 | 6 | 52827661 | 52888628 | - | protein_coding | NA |

| | | | | | | | | | |
|---|---|---|---|---|---|---|---|---|---|
| ENSRNOG00000008939 | FALSE | TRUE | Nxph1 | 4 | 36997669 | 37311132 | + | protein_coding | NA |
| ENSRNOG00000018929 | FALSE | TRUE | Kif20b | 1 | 2.32E+08 | 2.32E+08 | + | protein_coding | NA |
| ENSRNOG00000014243 | FALSE | TRUE | Pear1 | 2 | 1.73E+08 | 1.73E+08 | - | protein_coding | NA |
| ENSRNOG00000025069 | FALSE | TRUE | Ly9 | 13 | 84079458 | 84100823 | - | protein_coding | NA |
| ENSRNOG00000030568 | FALSE | TRUE | Rgs12 | 14 | 75715934 | 75794596 | - | protein_coding | NA |
| ENSRNOG00000032959 | FALSE | TRUE | Adh7 | 2 | 2.27E+08 | 2.27E+08 | + | protein_coding | NA |
| ENSRNOG00000068431 | FALSE | TRUE | Eppk1 | 7 | 1.08E+08 | 1.08E+08 | - | protein_coding | NA |
| ENSRNOG00000009620 | FALSE | TRUE | Cybrd1 | 3 | 55934874 | 55962108 | + | protein_coding | NA |
| ENSRNOG00000001854 | FALSE | TRUE | Tmtc1 | 4 | 1.81E+08 | 1.81E+08 | - | protein_coding | NA |
| ENSRNOG00000003720 | FALSE | TRUE | Prrx1 | 13 | 75601706 | 75670866 | - | protein_coding | Homeobox |
| ENSRNOG00000024594 | FALSE | TRUE | Fhdc1 | 2 | 1.7E+08 | 1.7E+08 | - | protein_coding | NA |
| ENSRNOG00000016695 | FALSE | TRUE | Mmp2 | 19 | 14154657 | 14182870 | - | protein_coding | NA |
| ENSRNOG00000015859 | FALSE | TRUE | Chdh | 16 | 5194269 | 5225537 | + | protein_coding | NA |
| ENSRNOG00000012562 | FALSE | TRUE | Grin3b | 7 | 9730862 | 9737183 | - | protein_coding | NA |
| ENSRNOG00000028137 | FALSE | TRUE | Mki67 | 1 | 1.9E+08 | 1.91E+08 | - | protein_coding | NA |
| ENSRNOG00000013048 | FALSE | TRUE | Pde7a | 2 | 1.02E+08 | 1.02E+08 | - | protein_coding | NA |
| ENSRNOG00000032539 | FALSE | TRUE | Spag8 | 5 | 57901682 | 57903894 | - | protein_coding | NA |
| ENSRNOG00000046772 | FALSE | TRUE | Fam83c | 3 | 1.44E+08 | 1.44E+08 | - | protein_coding | NA |
| ENSRNOG00000004882 | FALSE | TRUE | Capn6 | X | 1.07E+08 | 1.07E+08 | - | protein_coding | NA |
| ENSRNOG00000007338 | FALSE | TRUE | Fbln2 | 4 | 1.24E+08 | 1.24E+08 | + | protein_coding | NA |
| ENSRNOG00000026866 | FALSE | TRUE | Syn3 | 7 | 17376372 | 17808790 | + | protein_coding | NA |
| ENSRNOG00000011269 | FALSE | TRUE | Sult1c3 | 9 | 7221578 | 7267030 | - | protein_coding | NA |
| ENSRNOG00000043007 | FALSE | TRUE | Rcn3 | 1 | 95564380 | 95573725 | - | protein_coding | NA |
| ENSRNOG00000015423 | FALSE | TRUE | Ccna2 | 2 | 1.19E+08 | 1.19E+08 | - | protein_coding | NA |
| ENSRNOG00000018126 | FALSE | TRUE | Abca1 | 5 | 67681297 | 67801170 | - | protein_coding | NA |
| ENSRNOG00000021213 | FALSE | TRUE | Lix1l | 2 | 1.84E+08 | 1.84E+08 | + | protein_coding | NA |
| ENSRNOG00000059604 | FALSE | TRUE | Loricrin | 2 | 1.78E+08 | 1.78E+08 | - | protein_coding | NA |
| ENSRNOG00000003716 | FALSE | TRUE | Batf3 | 13 | 1.03E+08 | 1.03E+08 | + | protein_coding | TF_bZIP |
| ENSRNOG00000004921 | FALSE | TRUE | Nusap1 | 3 | 1.07E+08 | 1.07E+08 | + | protein_coding | NA |
| ENSRNOG00000016756 | FALSE | TRUE | Ptgir | 1 | 77579596 | 77581979 | + | protein_coding | NA |
| ENSRNOG00000021441 | FALSE | TRUE | Reln | 4 | 12736130 | 13162211 | + | protein_coding | NA |
| ENSRNOG00000007483 | FALSE | TRUE | Ccnf | 10 | 13253380 | 13279101 | - | protein_coding | NA |
| ENSRNOG00000068586 | FALSE | TRUE | Krt6a | 7 | 1.33E+08 | 1.33E+08 | - | protein_coding | NA |
| ENSRNOG00000004795 | FALSE | TRUE | Pof1b | X | 77683128 | 77749688 | - | protein_coding | NA |

| | | | | | | | | | |
|---|---|---|---|---|---|---|---|---|---|
| ENSRNOG00000016879 | FALSE | TRUE | Ldlrad4 | 18 | 61521428 | 61843238 | + | protein_coding | NA |
| ENSRNOG00000012826 | FALSE | TRUE | Creb3l2 | 4 | 65907655 | 66023763 | - | protein_coding | TF_bZIP |
| ENSRNOG00000069830 | FALSE | TRUE | ENSRNOG00000069830 | 3 | 74282149 | 74287593 | - | protein_coding | NA |
| ENSRNOG00000014367 | FALSE | TRUE | Ephb6 | 4 | 70491973 | 70507230 | + | protein_coding | NA |
| ENSRNOG00000054576 | FALSE | TRUE | Gsdmc | 7 | 95594015 | 95611344 | - | protein_coding | NA |
| ENSRNOG00000047491 | FALSE | TRUE | AABR07037520.1 | X | 19563794 | 19567126 | - | protein_coding | NA |
| ENSRNOG00000027724 | FALSE | TRUE | Plekhf1 | 1 | 90904742 | 90915820 | - | protein_coding | NA |
| ENSRNOG00000003813 | FALSE | TRUE | Kcnk10 | 6 | 1.18E+08 | 1.18E+08 | - | protein_coding | NA |
| ENSRNOG00000050539 | FALSE | TRUE | Fbln5 | 6 | 1.21E+08 | 1.21E+08 | - | protein_coding | NA |
| ENSRNOG00000010105 | FALSE | TRUE | S100a11 | 2 | 1.79E+08 | 1.79E+08 | + | protein_coding | NA |
| ENSRNOG00000013306 | FALSE | TRUE | Pcdh20 | 15 | 64425534 | 64431790 | - | protein_coding | NA |
| ENSRNOG00000064585 | FALSE | TRUE | Dcdc2b | 5 | 1.42E+08 | 1.42E+08 | - | protein_coding | NA |
| ENSRNOG00000007666 | FALSE | TRUE | Cacnb4 | 3 | 36910427 | 37168944 | - | protein_coding | NA |
| ENSRNOG00000000567 | FALSE | TRUE | Unc5b | 20 | 28697726 | 28764537 | - | protein_coding | NA |
| ENSRNOG00000067573 | FALSE | TRUE | ENSRNOG00000067573 | 10 | 1.02E+08 | 1.02E+08 | - | lncRNA | NA |
| ENSRNOG00000017231 | FALSE | TRUE | Adam9 | 16 | 67022655 | 67100917 | + | protein_coding | NA |
| ENSRNOG00000018316 | FALSE | TRUE | Grap2 | 7 | 1.12E+08 | 1.12E+08 | + | protein_coding | NA |
| ENSRNOG00000054286 | FALSE | TRUE | Rrm2 | 6 | 41340557 | 41346773 | + | protein_coding | NA |
| ENSRNOG00000046635 | FALSE | TRUE | Cdca5 | 1 | 2.03E+08 | 2.03E+08 | + | protein_coding | NA |
| ENSRNOG00000000001 | FALSE | TRUE | Arsj | 2 | 2.15E+08 | 2.15E+08 | + | protein_coding | NA |
| ENSRNOG00000001192 | FALSE | TRUE | Gltp | 12 | 41907144 | 41927319 | + | protein_coding | NA |
| ENSRNOG00000037211 | FALSE | TRUE | Kif14 | 13 | 47927044 | 47989164 | + | protein_coding | NA |
| ENSRNOG00000010244 | FALSE | TRUE | Sarm1 | 10 | 63369456 | 63392822 | - | protein_coding | NA |
| ENSRNOG00000024365 | FALSE | TRUE | Ect2 | 2 | 1.1E+08 | 1.1E+08 | - | protein_coding | NA |
| ENSRNOG00000007292 | FALSE | TRUE | Spdl1 | 10 | 19269127 | 19294127 | - | protein_coding | NA |
| ENSRNOG00000017753 | FALSE | TRUE | Ercc2 | 1 | 79033326 | 79047102 | + | protein_coding | NA |
| ENSRNOG00000004265 | FALSE | TRUE | Dipk2b | X | 4205490 | 4271574 | + | protein_coding | NA |
| ENSRNOG00000021644 | FALSE | TRUE | Slc15a3 | 1 | 2.07E+08 | 2.08E+08 | + | protein_coding | NA |
| ENSRNOG00000066475 | FALSE | TRUE | ENSRNOG00000066475 | 7 | 1169039 | 1178734 | - | protein_coding | NA |
| ENSRNOG00000050223 | FALSE | TRUE | Rin1 | 1 | 2.02E+08 | 2.02E+08 | + | protein_coding | NA |
| ENSRNOG00000053045 | FALSE | TRUE | Wscd2 | 12 | 43048043 | 43088591 | - | protein_coding | NA |
| ENSRNOG00000053450 | FALSE | TRUE | Myadm | 1 | 65864173 | 65874035 | - | protein_coding | NA |
| ENSRNOG00000056151 | FALSE | TRUE | AABR07007642.1 | 2 | 23117004 | 23361240 | - | protein_coding | NA |
| ENSRNOG00000048411 | FALSE | TRUE | Uhrf1 | 9 | 1134909 | 1154631 | + | protein_coding | NA |

| | | | | | | | | | |
|---|---|---|---|---|---|---|---|---|---|
| ENSRNOG00000007680 | FALSE | TRUE | Fhl5 | 5 | 38907946 | 38956552 | - | protein_coding | NA |
| ENSRNOG00000020716 | FALSE | TRUE | Axl | 1 | 81265088 | 81296265 | - | protein_coding | NA |
| ENSRNOG00000054809 | FALSE | TRUE | AABR07026032.1 | 16 | 56422138 | 56471065 | - | protein_coding | NA |
| ENSRNOG00000032576 | FALSE | TRUE | Nlgn1 | 2 | 1.08E+08 | 1.09E+08 | - | protein_coding | NA |
| ENSRNOG00000014008 | FALSE | TRUE | Mfsd2a | 5 | 1.35E+08 | 1.35E+08 | - | protein_coding | NA |
| ENSRNOG00000019128 | FALSE | TRUE | St8sia4 | 9 | 95993503 | 96084653 | - | protein_coding | NA |
| ENSRNOG00000047473 | FALSE | TRUE | Dchs2 | 2 | 1.68E+08 | 1.69E+08 | + | protein_coding | NA |
| ENSRNOG00000015713 | FALSE | TRUE | Parva | 1 | 1.67E+08 | 1.67E+08 | + | protein_coding | NA |
| ENSRNOG00000008465 | FALSE | TRUE | Tmem176b | 4 | 77766928 | 77774384 | - | protein_coding | NA |
| ENSRNOG00000007454 | FALSE | TRUE | Aloxe3 | 10 | 53831264 | 53854328 | + | protein_coding | NA |
| ENSRNOG00000021793 | FALSE | TRUE | Tlcd5 | 8 | 43479016 | 43486290 | - | protein_coding | NA |
| ENSRNOG00000025110 | FALSE | TRUE | Vwa3a | 1 | 1.75E+08 | 1.75E+08 | + | protein_coding | NA |
| ENSRNOG00000013166 | FALSE | TRUE | Wnt4 | 5 | 1.5E+08 | 1.5E+08 | + | protein_coding | NA |
| ENSRNOG00000067242 | FALSE | TRUE | ENSRNOG00000067242 | X | 1.12E+08 | 1.12E+08 | - | protein_coding | NA |
| ENSRNOG00000019864 | FALSE | TRUE | C1qtnf12 | 5 | 1.67E+08 | 1.67E+08 | + | protein_coding | NA |
| ENSRNOG00000013330 | FALSE | TRUE | Cdhr1 | 16 | 12843437 | 12863396 | - | protein_coding | NA |
| ENSRNOG00000049495 | FALSE | TRUE | Krt71 | 7 | 1.33E+08 | 1.33E+08 | - | protein_coding | NA |
| ENSRNOG00000006753 | FALSE | TRUE | Tgm3 | 3 | 1.17E+08 | 1.17E+08 | + | protein_coding | NA |
| ENSRNOG00000008586 | FALSE | TRUE | Aldh1l2 | 7 | 20254233 | 20305776 | + | protein_coding | NA |
| ENSRNOG00000014357 | FALSE | TRUE | Gja4 | 5 | 1.4E+08 | 1.4E+08 | - | protein_coding | NA |
| ENSRNOG00000057832 | FALSE | TRUE | Rnf125 | 18 | 12253852 | 12275983 | + | protein_coding | NA |
| ENSRNOG00000011544 | FALSE | TRUE | Zfp219 | 15 | 24695837 | 24710030 | - | protein_coding | zf-C2H2 |
| ENSRNOG00000006086 | FALSE | TRUE | Lynx1 | 7 | 1.07E+08 | 1.07E+08 | - | protein_coding | NA |
| ENSRNOG00000020995 | FALSE | TRUE | Fut1 | 1 | 96086635 | 96090616 | + | protein_coding | NA |
| ENSRNOG00000043167 | FALSE | TRUE | Itga9 | 8 | 1.18E+08 | 1.19E+08 | + | protein_coding | NA |
| ENSRNOG00000029055 | FALSE | TRUE | Ttk | 8 | 84754315 | 84792651 | + | protein_coding | NA |
| ENSRNOG00000008103 | FALSE | TRUE | Mdh1 | 14 | 95630306 | 95645925 | - | protein_coding | NA |
| ENSRNOG00000030530 | FALSE | TRUE | Gzmm | 7 | 10011066 | 10016665 | - | protein_coding | NA |
| ENSRNOG00000019322 | FALSE | TRUE | Notch1 | 3 | 9278086 | 9323531 | - | protein_coding | NA |
| ENSRNOG00000003736 | FALSE | TRUE | Col5a2 | 9 | 47448736 | 47598154 | - | protein_coding | NA |
| ENSRNOG00000023972 | FALSE | TRUE | Col4a2 | 16 | 78047602 | 78183839 | - | protein_coding | NA |
| ENSRNOG00000058870 | FALSE | TRUE | Mroh7 | 5 | 1.21E+08 | 1.22E+08 | - | protein_coding | NA |
| ENSRNOG00000031136 | FALSE | TRUE | Ntng1 | 2 | 1.97E+08 | 1.98E+08 | - | protein_coding | NA |
| ENSRNOG00000068021 | FALSE | TRUE | ENSRNOG00000068021 | 6 | 10516336 | 10519170 | + | lncRNA | NA |

| | | | | | | | | | |
|---|---|---|---|---|---|---|---|---|---|
| ENSRNOG00000012405 | FALSE | TRUE | Tcf4 | 18 | 62943782 | 63284425 | + | protein_coding | bHLH |
| ENSRNOG00000023643 | FALSE | TRUE | Mmp17 | 12 | 27074409 | 27099316 | - | protein_coding | NA |
| ENSRNOG00000051770 | FALSE | TRUE | Cyp2w1 | 12 | 15291704 | 15296421 | - | protein_coding | NA |
| ENSRNOG00000002497 | FALSE | TRUE | Anos1 | 10 | 68561954 | 68562801 | + | protein_coding | NA |
| ENSRNOG00000002682 | FALSE | TRUE | Zfp692 | 10 | 42488588 | 42496018 | + | protein_coding | zf-C2H2 |
| ENSRNOG00000016281 | FALSE | TRUE | Col4a1 | 16 | 78183533 | 78294412 | + | protein_coding | NA |
| ENSRNOG00000071194 | FALSE | TRUE | ENSRNOG00000071194 | 1 | 81298301 | 81301536 | - | lncRNA | NA |
| ENSRNOG00000033796 | FALSE | TRUE | Kcnj5 | 8 | 30724925 | 30753518 | - | protein_coding | NA |
| ENSRNOG00000056069 | FALSE | TRUE | Kif11 | 1 | 2.35E+08 | 2.35E+08 | + | protein_coding | NA |
| ENSRNOG00000016057 | FALSE | TRUE | Kcnj13 | 9 | 88063003 | 88071112 | - | protein_coding | NA |
| ENSRNOG00000045949 | FALSE | TRUE | Faah | 5 | 1.29E+08 | 1.29E+08 | - | protein_coding | NA |
| ENSRNOG00000001715 | FALSE | TRUE | Ece2 | 11 | 80263162 | 80278428 | - | protein_coding | NA |
| ENSRNOG00000056228 | FALSE | TRUE | Atp10a | 1 | 1.1E+08 | 1.1E+08 | + | protein_coding | NA |
| ENSRNOG00000055796 | FALSE | TRUE | AC103574.1 | 1 | 65645942 | 65662502 | + | protein_coding | NA |
| ENSRNOG00000059510 | FALSE | TRUE | Amph | 17 | 45739395 | 45983315 | - | protein_coding | NA |
| ENSRNOG00000011150 | FALSE | TRUE | Arsb | 2 | 25002346 | 25162671 | + | protein_coding | NA |
| ENSRNOG00000003039 | FALSE | TRUE | Wnt3a | 10 | 44034194 | 44078324 | - | protein_coding | NA |
| ENSRNOG00000007439 | FALSE | TRUE | Unc13d | 10 | 1.01E+08 | 1.01E+08 | - | protein_coding | NA |
| ENSRNOG00000070245 | FALSE | TRUE | Vstm1 | 1 | 65687375 | 65701188 | + | protein_coding | NA |
| ENSRNOG00000002460 | FALSE | TRUE | Serpinb2 | 13 | 23541400 | 23550408 | + | protein_coding | NA |
| ENSRNOG00000020136 | FALSE | TRUE | Tgm1 | 15 | 29191041 | 29204523 | - | protein_coding | NA |
| ENSRNOG00000016260 | FALSE | TRUE | Polr3g | 2 | 11945217 | 11986125 | - | protein_coding | NA |
| ENSRNOG00000006588 | FALSE | TRUE | Meox2 | 6 | 53856758 | 53917467 | + | protein_coding | Homeobox |
| ENSRNOG00000019100 | FALSE | TRUE | Kif2c | 5 | 1.31E+08 | 1.31E+08 | - | protein_coding | NA |
| ENSRNOG00000003172 | FALSE | TRUE | Serpinf1 | 10 | 60249708 | 60262646 | - | protein_coding | NA |
| ENSRNOG00000020784 | FALSE | TRUE | Kcnk7 | 1 | 2.03E+08 | 2.03E+08 | + | protein_coding | NA |
| ENSRNOG00000004677 | FALSE | TRUE | Zeb2 | 3 | 29218301 | 29345157 | - | protein_coding | zf-C2H2 |
| ENSRNOG00000012772 | FALSE | TRUE | Nqo1 | 19 | 35295573 | 35310557 | - | protein_coding | NA |
| ENSRNOG00000056343 | FALSE | TRUE | Lats2 | 15 | 31825092 | 31877220 | - | protein_coding | NA |
| ENSRNOG00000052564 | FALSE | TRUE | Gpx3 | 10 | 39028570 | 39037035 | + | lncRNA | NA |
| ENSRNOG00000013057 | FALSE | TRUE | Prc1 | 1 | 1.34E+08 | 1.34E+08 | + | protein_coding | NA |
| ENSRNOG00000015201 | FALSE | TRUE | Tmem266 | 8 | 55707122 | 55820692 | + | protein_coding | NA |
| ENSRNOG00000033321 | FALSE | TRUE | Hmgb2l1 | 11 | 47252569 | 47253669 | - | protein_coding | HMG |
| ENSRNOG00000019737 | FALSE | TRUE | Sema4a | 2 | 1.74E+08 | 1.74E+08 | - | protein_coding | NA |

| | | | | | | | | | |
|---|---|---|---|---|---|---|---|---|---|
| ENSRNOG00000063216 | FALSE | TRUE | Ccnb2 | 8 | 71087595 | 71100874 | - | protein_coding | NA |
| ENSRNOG00000009360 | FALSE | TRUE | Sh3bp1 | 7 | 1.1E+08 | 1.1E+08 | + | protein_coding | NA |
| ENSRNOG00000025005 | FALSE | TRUE | Ccdc190 | 13 | 82147176 | 82154863 | + | protein_coding | NA |
| ENSRNOG00000012640 | FALSE | TRUE | Dpp7 | 3 | 8165091 | 8169355 | - | protein_coding | NA |
| ENSRNOG00000018260 | FALSE | TRUE | Hrh2 | 17 | 10368298 | 10407631 | - | protein_coding | NA |
| ENSRNOG00000062844 | FALSE | TRUE | ENSRNOG00000062844 | X | 1.12E+08 | 1.12E+08 | - | lncRNA | NA |
| ENSRNOG00000022337 | FALSE | TRUE | Slitrk6 | 15 | 87563322 | 87570393 | - | protein_coding | NA |
| ENSRNOG00000023216 | FALSE | TRUE | Ccdc17 | 5 | 1.3E+08 | 1.3E+08 | + | protein_coding | NA |
| ENSRNOG00000020006 | FALSE | TRUE | Wdr90 | 10 | 14864670 | 14881292 | - | protein_coding | NA |
| ENSRNOG00000020762 | FALSE | TRUE | Zfp260 | 1 | 85336618 | 85350067 | + | protein_coding | zf-C2H2 |
| ENSRNOG00000011582 | FALSE | TRUE | Rab3d | 8 | 20439294 | 20449185 | - | protein_coding | NA |
| ENSRNOG00000016044 | FALSE | TRUE | Mab21l3 | 2 | 1.89E+08 | 1.89E+08 | - | protein_coding | NA |
| ENSRNOG00000011373 | FALSE | TRUE | Reep4 | 15 | 45619941 | 45623526 | + | protein_coding | NA |
| ENSRNOG00000017550 | FALSE | TRUE | Fbln7 | 3 | 1.16E+08 | 1.16E+08 | + | protein_coding | NA |
| ENSRNOG00000020701 | FALSE | TRUE | Brca1 | 10 | 86418000 | 86477304 | - | protein_coding | NA |
| ENSRNOG00000050233 | FALSE | TRUE | Cchcr1 | 20 | 3205676 | 3218308 | - | protein_coding | NA |
| ENSRNOG00000002711 | FALSE | TRUE | Nuf2 | 13 | 81693598 | 81722766 | - | protein_coding | NA |
| ENSRNOG00000027319 | FALSE | TRUE | Ephx3 | 7 | 11206812 | 11212122 | - | protein_coding | NA |
| ENSRNOG00000026653 | FALSE | TRUE | Hcar2 | 12 | 32726184 | 32728504 | + | protein_coding | NA |
| ENSRNOG00000051704 | FALSE | TRUE | ENSRNOG00000051704 | X | 1.51E+08 | 1.51E+08 | + | protein_coding | NA |
| ENSRNOG00000016377 | FALSE | TRUE | Cep55 | 1 | 2.36E+08 | 2.36E+08 | + | protein_coding | NA |
| ENSRNOG00000020078 | FALSE | TRUE | Vstm4 | 16 | 8120336 | 8200269 | + | protein_coding | NA |
| ENSRNOG00000005747 | FALSE | TRUE | Il27ra | 19 | 24109972 | 24121938 | + | protein_coding | NA |
| ENSRNOG00000013598 | FALSE | TRUE | Melk | 5 | 58540449 | 58600937 | + | protein_coding | NA |
| ENSRNOG00000021243 | FALSE | TRUE | Siglec1 | 3 | 1.18E+08 | 1.18E+08 | - | protein_coding | NA |
| ENSRNOG00000005214 | FALSE | TRUE | Plek | 14 | 91397019 | 91454131 | - | protein_coding | Others |
| ENSRNOG00000014414 | FALSE | TRUE | Qrfpr | 2 | 1.19E+08 | 1.19E+08 | - | protein_coding | NA |
| ENSRNOG00000024705 | FALSE | TRUE | Rarres2 | 4 | 77522535 | 77525556 | - | protein_coding | NA |
| ENSRNOG00000057078 | FALSE | TRUE | Ddit4 | 20 | 27891998 | 27894105 | - | protein_coding | NA |
| ENSRNOG00000062262 | FALSE | TRUE | ENSRNOG00000062262 | 1 | 86038437 | 86041455 | + | protein_coding | NA |
| ENSRNOG00000014064 | FALSE | TRUE | Ctsh | 8 | 90608941 | 90627824 | + | protein_coding | NA |
| ENSRNOG00000003650 | FALSE | TRUE | Nt5c | 10 | 1.01E+08 | 1.01E+08 | - | protein_coding | NA |
| ENSRNOG00000042944 | FALSE | TRUE | Cenpw | 1 | 27525324 | 27530464 | + | protein_coding | NA |
| ENSRNOG00000005378 | FALSE | TRUE | Gna15 | 7 | 8184861 | 8205508 | + | protein_coding | NA |

| | | | | | | | | | |
|---|---|---|---|---|---|---|---|---|---|
| ENSRNOG00000032410 | FALSE | TRUE | Mtus2 | 12 | 6706532 | 7078331 | - | protein_coding | NA |
| ENSRNOG00000000175 | FALSE | TRUE | Mier2 | 7 | 10153649 | 10169378 | + | protein_coding | MYB |
| ENSRNOG00000014343 | FALSE | TRUE | Anln | 8 | 20858228 | 20921538 | - | protein_coding | NA |
| ENSRNOG00000030246 | FALSE | TRUE | Btla | 11 | 55564899 | 55587181 | - | protein_coding | NA |
| ENSRNOG00000000488 | FALSE | TRUE | Hmga1 | 20 | 5611694 | 5618752 | + | protein_coding | HMGA |
| ENSRNOG00000017560 | FALSE | TRUE | Mdk | 3 | 77901158 | 77903130 | - | protein_coding | NA |
| ENSRNOG00000032693 | FALSE | TRUE | ENSRNOG00000032693 | 12 | 15732557 | 15786843 | - | protein_coding | NA |
| ENSRNOG00000053691 | FALSE | TRUE | Lama5 | 3 | 1.67E+08 | 1.67E+08 | - | protein_coding | NA |
| ENSRNOG00000010210 | FALSE | TRUE | Slc7a11 | 2 | 1.34E+08 | 1.35E+08 | - | protein_coding | NA |
| ENSRNOG00000009447 | FALSE | TRUE | Fam221a | 4 | 78337296 | 78358909 | + | protein_coding | NA |
| ENSRNOG00000028627 | FALSE | TRUE | Hmcn1 | 13 | 62615461 | 63084524 | - | protein_coding | NA |
| ENSRNOG00000011300 | FALSE | TRUE | Lama3 | 18 | 3523133 | 3751353 | + | protein_coding | NA |
| ENSRNOG00000048472 | FALSE | TRUE | Comp | 16 | 19047207 | 19055845 | - | protein_coding | NA |
| ENSRNOG00000008450 | FALSE | TRUE | ENSRNOG00000008450 | 19 | 23879383 | 23881937 | + | lncRNA | NA |
| ENSRNOG00000057451 | FALSE | TRUE | Itga5 | 7 | 1.34E+08 | 1.35E+08 | - | protein_coding | NA |
| ENSRNOG00000003812 | FALSE | TRUE | Nlgn3 | X | 66429458 | 66451876 | + | protein_coding | NA |
| ENSRNOG00000069481 | FALSE | TRUE | Abcg3l1 | 14 | 5124353 | 5177184 | - | protein_coding | NA |
| ENSRNOG00000016349 | FALSE | TRUE | Gnmt | 9 | 14254675 | 14258434 | + | protein_coding | NA |
| ENSRNOG00000013704 | FALSE | TRUE | Cps1 | 9 | 68614153 | 68737033 | + | protein_coding | NA |
| ENSRNOG00000016148 | FALSE | TRUE | Gtse1 | 7 | 1.17E+08 | 1.17E+08 | + | protein_coding | NA |
| ENSRNOG00000001645 | FALSE | TRUE | Filip1l | 11 | 42940467 | 43177415 | - | protein_coding | NA |
| ENSRNOG00000009503 | FALSE | TRUE | Depdc1 | 2 | 2.49E+08 | 2.49E+08 | + | protein_coding | NA |
| ENSRNOG00000013656 | FALSE | TRUE | Lpar1 | 5 | 73229625 | 73369895 | - | protein_coding | NA |
| ENSRNOG00000040208 | FALSE | TRUE | Mmp27 | 8 | 4745883 | 4755806 | + | protein_coding | NA |
| ENSRNOG00000059219 | FALSE | TRUE | Myo15a | 10 | 45278737 | 45335340 | + | protein_coding | NA |
| ENSRNOG00000027787 | FALSE | TRUE | Cdc6 | 10 | 83864638 | 83878011 | + | protein_coding | NA |
| ENSRNOG00000004487 | FALSE | TRUE | Nek2l1 | 13 | 1.03E+08 | 1.03E+08 | + | protein_coding | NA |
| ENSRNOG00000039891 | FALSE | TRUE | Adgrg5 | 19 | 10079375 | 10097214 | - | protein_coding | NA |
| ENSRNOG00000067723 | FALSE | TRUE | Pclaf | 8 | 66420587 | 66432994 | + | protein_coding | NA |
| ENSRNOG00000012428 | FALSE | TRUE | Maf | 19 | 43360342 | 43712365 | - | protein_coding | TF_bZIP |
| ENSRNOG00000017273 | FALSE | TRUE | RGD1563917 | 9 | 86383201 | 86405361 | - | protein_coding | NA |
| ENSRNOG00000021693 | FALSE | TRUE | Fam149a | 16 | 46854635 | 46912066 | + | protein_coding | NA |
| ENSRNOG00000021433 | FALSE | TRUE | Arhgef39 | 5 | 57752509 | 57756109 | - | protein_coding | NA |
| ENSRNOG00000000479 | FALSE | TRUE | Kifc1 | 20 | 4999047 | 5017105 | + | protein_coding | NA |

| | | | | | | | | | |
|---|---|---|---|---|---|---|---|---|---|
| ENSRNOG00000009708 | FALSE | TRUE | Tas1r1 | 5 | 1.63E+08 | 1.63E+08 | - | protein_coding | NA |
| ENSRNOG00000069957 | FALSE | TRUE | ENSRNOG00000069957 | 15 | 28350801 | 28356566 | + | lncRNA | NA |
| ENSRNOG00000018413 | FALSE | TRUE | Per3 | 5 | 1.61E+08 | 1.61E+08 | - | protein_coding | NA |
| ENSRNOG00000043478 | FALSE | TRUE | AABR07006142.1 | 2 | 85011149 | 85049805 | + | protein_coding | NA |
| ENSRNOG00000017932 | FALSE | TRUE | St3gal2 | 19 | 38923999 | 38939869 | + | protein_coding | NA |
| ENSRNOG00000059622 | FALSE | TRUE | Pigb | 8 | 73751798 | 73775679 | - | protein_coding | NA |
| ENSRNOG00000016357 | FALSE | TRUE | Golm2 | 3 | 1.09E+08 | 1.09E+08 | + | protein_coding | NA |
| ENSRNOG00000009177 | FALSE | TRUE | Fcer1a | 13 | 85686092 | 85692351 | - | protein_coding | NA |
| ENSRNOG00000020213 | FALSE | TRUE | Tubg1 | 10 | 86052743 | 86059433 | + | protein_coding | NA |
| ENSRNOG00000005930 | FALSE | TRUE | Nnmt | 8 | 48933598 | 48946655 | - | protein_coding | NA |
| ENSRNOG00000030127 | FALSE | TRUE | Eml2 | 1 | 78828080 | 78859341 | + | protein_coding | NA |
| ENSRNOG00000060054 | FALSE | TRUE | Doc2b | 10 | 60628029 | 60653267 | - | protein_coding | NA |
| ENSRNOG00000007657 | FALSE | TRUE | Col27a1 | 5 | 76647429 | 76765174 | + | protein_coding | NA |
| ENSRNOG00000048433 | FALSE | TRUE | Tshz2 | 3 | 1.58E+08 | 1.59E+08 | + | protein_coding | zf-C2H2 |
| ENSRNOG00000000897 | FALSE | TRUE | Rxfp2 | 12 | 4925722 | 4986596 | - | protein_coding | NA |
| ENSRNOG00000049743 | FALSE | TRUE | Gstm3 | 2 | 1.96E+08 | 1.96E+08 | + | protein_coding | NA |
| ENSRNOG00000007960 | FALSE | TRUE | Theg | 7 | 10127424 | 10137531 | + | protein_coding | NA |
| ENSRNOG00000015131 | FALSE | TRUE | Ube2c | 3 | 1.54E+08 | 1.54E+08 | + | protein_coding | NA |
| ENSRNOG00000053687 | FALSE | TRUE | Znf660 | 8 | 1.23E+08 | 1.23E+08 | + | protein_coding | zf-C2H2 |
| ENSRNOG00000014896 | FALSE | TRUE | Adgrg3 | 19 | 9972430 | 10001123 | - | protein_coding | NA |
| ENSRNOG00000011376 | FALSE | TRUE | Bbof1 | 6 | 1.04E+08 | 1.04E+08 | + | protein_coding | NA |
| ENSRNOG00000020505 | FALSE | TRUE | Map4k1 | 1 | 84271069 | 84292493 | + | protein_coding | NA |
| ENSRNOG00000009105 | FALSE | TRUE | Kb15 | 7 | 1.33E+08 | 1.33E+08 | - | protein_coding | NA |
| ENSRNOG00000038483 | FALSE | TRUE | Tnfrsf10b | 15 | 44840386 | 44867467 | + | protein_coding | NA |
| ENSRNOG00000001427 | FALSE | TRUE | Orai2 | 12 | 20497317 | 20520428 | + | protein_coding | NA |
| ENSRNOG00000008040 | FALSE | TRUE | Pimreg | 10 | 56669675 | 56674791 | + | protein_coding | NA |
| ENSRNOG00000016488 | FALSE | TRUE | Pltp | 3 | 1.54E+08 | 1.54E+08 | - | protein_coding | NA |
| ENSRNOG00000047647 | FALSE | TRUE | Il2ra | 17 | 66849974 | 66898697 | - | protein_coding | NA |
| ENSRNOG00000018177 | FALSE | TRUE | Dhrs2 | 15 | 28688940 | 28703644 | + | protein_coding | NA |
| ENSRNOG00000015036 | FALSE | TRUE | Ccn2 | 1 | 20802199 | 20805734 | - | protein_coding | NA |
| ENSRNOG00000047276 | FALSE | TRUE | ENSRNOG00000047276 | 16 | 70838568 | 70840795 | + | protein_coding | NA |
| ENSRNOG00000042411 | FALSE | TRUE | Rps6ka1 | 5 | 1.46E+08 | 1.46E+08 | - | protein_coding | NA |
| ENSRNOG00000047314 | FALSE | TRUE | Tk1 | 10 | 1.03E+08 | 1.03E+08 | - | protein_coding | NA |
| ENSRNOG00000020279 | FALSE | TRUE | Syt11 | 2 | 1.74E+08 | 1.74E+08 | - | protein_coding | NA |

| | | | | | | | | | |
|---|---|---|---|---|---|---|---|---|---|
| ENSRNOG00000068243 | FALSE | TRUE | Cdk5r1 | 10 | 65483941 | 65488456 | + | protein_coding | NA |
| ENSRNOG00000049033 | FALSE | TRUE | Racgap1 | 7 | 1.31E+08 | 1.31E+08 | - | protein_coding | NA |
| ENSRNOG00000013577 | FALSE | TRUE | Kdelr3 | 7 | 1.11E+08 | 1.11E+08 | + | protein_coding | NA |
| ENSRNOG00000025994 | FALSE | TRUE | Krt80 | 7 | 1.32E+08 | 1.32E+08 | - | protein_coding | NA |
| ENSRNOG00000027037 | FALSE | TRUE | Alox12 | 10 | 54958271 | 54970542 | - | protein_coding | NA |
| ENSRNOG00000065081 | FALSE | TRUE | Otx1 | 14 | 96082151 | 96089086 | - | protein_coding | TF_Otx |
| ENSRNOG00000015878 | FALSE | TRUE | Pif1 | 8 | 66111072 | 66120200 | + | protein_coding | NA |
| ENSRNOG00000031849 | FALSE | TRUE | Armh4 | 15 | 22975895 | 23076949 | - | protein_coding | NA |
| ENSRNOG00000018533 | FALSE | TRUE | Iffo1 | 4 | 1.58E+08 | 1.58E+08 | + | protein_coding | NA |
| ENSRNOG00000055408 | FALSE | TRUE | Gcat | 7 | 1.11E+08 | 1.11E+08 | + | protein_coding | NA |
| ENSRNOG00000061484 | FALSE | TRUE | Adamts2 | 10 | 34921049 | 35123821 | + | protein_coding | NA |
| ENSRNOG00000005708 | FALSE | TRUE | Mmp16 | 5 | 31312280 | 31548388 | + | protein_coding | NA |
| ENSRNOG00000056562 | FALSE | TRUE | Olfml1 | 1 | 1.61E+08 | 1.62E+08 | + | protein_coding | NA |
| ENSRNOG00000011193 | FALSE | TRUE | Hmgcll1 | 8 | 76757497 | 76886816 | + | protein_coding | NA |
| ENSRNOG00000006060 | FALSE | TRUE | Matn2 | 7 | 65494834 | 65645113 | + | protein_coding | NA |
| ENSRNOG00000042519 | FALSE | TRUE | Peak1 | 8 | 56609015 | 56819096 | - | protein_coding | NA |
| ENSRNOG00000021234 | FALSE | TRUE | Slc4a11 | 3 | 1.18E+08 | 1.18E+08 | - | protein_coding | NA |
| ENSRNOG00000064154 | FALSE | TRUE | Rassf10 | 1 | 1.67E+08 | 1.67E+08 | + | protein_coding | NA |
| ENSRNOG00000000442 | FALSE | TRUE | Notch4 | 20 | 4160445 | 4184465 | - | protein_coding | NA |
| ENSRNOG00000006075 | FALSE | TRUE | Fez1 | 8 | 36544535 | 36589683 | + | protein_coding | NA |
| ENSRNOG00000024120 | FALSE | TRUE | Rxfp1 | 2 | 1.65E+08 | 1.65E+08 | - | protein_coding | NA |
| ENSRNOG00000067108 | FALSE | TRUE | ENSRNOG00000067108 | 5 | 98268768 | 98270330 | + | protein_coding | NA |
| ENSRNOG00000014215 | FALSE | TRUE | Klf9 | 1 | 2.21E+08 | 2.21E+08 | + | protein_coding | zf-C2H2 |
| ENSRNOG00000031773 | FALSE | TRUE | Cngb1 | 19 | 9726595 | 9791173 | + | protein_coding | NA |
| ENSRNOG00000039969 | FALSE | TRUE | Dsc2 | 18 | 11450390 | 11482392 | - | protein_coding | NA |
| ENSRNOG00000013727 | FALSE | TRUE | Ndc80 | 9 | 1.11E+08 | 1.11E+08 | - | protein_coding | NA |
| ENSRNOG00000020587 | FALSE | TRUE | Efemp2 | 1 | 2.03E+08 | 2.03E+08 | + | protein_coding | NA |
| ENSRNOG00000020202 | FALSE | TRUE | Asrgl1 | 1 | 2.06E+08 | 2.06E+08 | - | protein_coding | NA |
| ENSRNOG00000000251 | FALSE | TRUE | St6galnac1 | 10 | 1.02E+08 | 1.02E+08 | - | protein_coding | NA |
| ENSRNOG00000006784 | FALSE | TRUE | Sema4f | 4 | 1.15E+08 | 1.15E+08 | - | protein_coding | NA |
| ENSRNOG00000066624 | FALSE | TRUE | ENSRNOG00000066624 | 14 | 94563775 | 94574801 | - | lncRNA | NA |
| ENSRNOG00000020653 | FALSE | TRUE | S1pr2 | 8 | 19502627 | 19523574 | - | protein_coding | NA |
| ENSRNOG00000019577 | FALSE | TRUE | Adamts19 | 18 | 52347428 | 52530509 | + | protein_coding | NA |
| ENSRNOG00000039079 | FALSE | TRUE | Ces2h | 19 | 32974242 | 32988830 | + | protein_coding | NA |

| | | | | | | | | | |
|---|---|---|---|---|---|---|---|---|---|
| ENSRNOG00000031431 | FALSE | TRUE | Cdca8 | 5 | 1.37E+08 | 1.37E+08 | - | protein_coding | NA |
| ENSRNOG00000032368 | FALSE | TRUE | Tlr11 | 15 | 23545408 | 23549849 | + | protein_coding | NA |
| ENSRNOG00000061085 | FALSE | TRUE | Fancd2 | 4 | 1.47E+08 | 1.47E+08 | + | protein_coding | NA |
| ENSRNOG00000002984 | FALSE | TRUE | Bmp15 | X | 16169123 | 16174187 | + | protein_coding | NA |
| ENSRNOG00000014819 | FALSE | TRUE | Hap1 | 10 | 85277890 | 85286126 | - | protein_coding | NA |
| ENSRNOG00000064855 | FALSE | TRUE | ENSRNOG00000064855 | 2 | 1.84E+08 | 1.84E+08 | + | lncRNA | NA |
| ENSRNOG00000003365 | FALSE | TRUE | Cadm3 | 13 | 85786483 | 85817749 | - | protein_coding | NA |
| ENSRNOG00000020991 | FALSE | TRUE | Ms4a6a | 1 | 2.08E+08 | 2.08E+08 | - | protein_coding | NA |
| ENSRNOG00000010050 | FALSE | TRUE | Stard9 | 3 | 1.08E+08 | 1.08E+08 | + | protein_coding | NA |
| ENSRNOG00000018087 | FALSE | TRUE | Vim | 17 | 76668647 | 76677187 | + | protein_coding | NA |
| ENSRNOG00000026143 | FALSE | TRUE | Ckap2l | 3 | 1.16E+08 | 1.17E+08 | - | protein_coding | NA |
| ENSRNOG00000007300 | FALSE | TRUE | C1qtnf6 | 7 | 1.1E+08 | 1.1E+08 | - | protein_coding | NA |
| ENSRNOG00000000632 | FALSE | TRUE | Cdk1 | 20 | 19266248 | 19281408 | + | protein_coding | NA |
| ENSRNOG00000015410 | FALSE | TRUE | Aspn | 17 | 15080639 | 15104041 | - | protein_coding | NA |
| ENSRNOG00000016143 | FALSE | TRUE | P3h3 | 4 | 1.58E+08 | 1.58E+08 | - | protein_coding | NA |
| ENSRNOG00000004033 | FALSE | TRUE | Sema6a | 18 | 40050624 | 40171954 | - | protein_coding | NA |
| ENSRNOG00000009830 | FALSE | TRUE | Fam204a | 1 | 2.59E+08 | 2.59E+08 | - | protein_coding | NA |
| ENSRNOG00000007830 | FALSE | TRUE | Apold1 | 4 | 1.68E+08 | 1.68E+08 | + | protein_coding | NA |
| ENSRNOG00000014758 | FALSE | TRUE | Lgi1 | 1 | 2.36E+08 | 2.36E+08 | + | protein_coding | NA |
| ENSRNOG00000016366 | FALSE | TRUE | Colec12 | 18 | 732950 | 920618 | + | protein_coding | NA |
| ENSRNOG00000000704 | FALSE | TRUE | Cmklr1 | 12 | 42974410 | 43028129 | + | protein_coding | NA |
| ENSRNOG00000058539 | FALSE | TRUE | Ccnb1 | 2 | 31912193 | 31921172 | - | protein_coding | NA |
| ENSRNOG00000050819 | FALSE | TRUE | Birc5 | 10 | 1.03E+08 | 1.03E+08 | + | protein_coding | NA |
| ENSRNOG00000006956 | FALSE | TRUE | Adamtsl1 | 5 | 99964486 | 1.01E+08 | + | protein_coding | NA |
| ENSRNOG00000025220 | FALSE | TRUE | Slc6a16 | 1 | 95726140 | 95750266 | + | protein_coding | NA |
| ENSRNOG00000004890 | FALSE | TRUE | Adcy8 | 7 | 96417324 | 96665911 | - | protein_coding | NA |
| ENSRNOG00000017078 | FALSE | TRUE | Selenon | 5 | 1.47E+08 | 1.47E+08 | - | protein_coding | NA |
| ENSRNOG00000063630 | FALSE | TRUE | Dlx6 | 4 | 34984232 | 34991343 | + | protein_coding | Homeobox |
| ENSRNOG00000008247 | FALSE | TRUE | Ikbip | 7 | 25579832 | 25605162 | + | protein_coding | NA |
| ENSRNOG00000006814 | FALSE | TRUE | Klrg2 | 4 | 67363919 | 67374147 | - | protein_coding | NA |
| ENSRNOG00000018005 | FALSE | TRUE | Duoxa1 | 3 | 1.09E+08 | 1.09E+08 | - | protein_coding | NA |
| ENSRNOG00000053055 | FALSE | TRUE | Otop3 | 10 | 1.01E+08 | 1.01E+08 | + | protein_coding | NA |
| ENSRNOG00000024089 | FALSE | TRUE | Fndc3b | 2 | 1.1E+08 | 1.11E+08 | - | protein_coding | NA |
| ENSRNOG00000018863 | FALSE | TRUE | Abcc10 | 9 | 14657264 | 14677178 | + | protein_coding | NA |

| | | | | | | | | | |
|---|---|---|---|---|---|---|---|---|---|
| ENSRNOG00000009552 | FALSE | TRUE | Serinc3 | 3 | 1.52E+08 | 1.52E+08 | - | protein_coding | NA |
| ENSRNOG00000014844 | FALSE | TRUE | Kif21a | 7 | 1.22E+08 | 1.22E+08 | - | protein_coding | NA |
| ENSRNOG00000007728 | FALSE | TRUE | Gsdmd | 7 | 1.08E+08 | 1.08E+08 | + | protein_coding | NA |
| ENSRNOG00000021020 | FALSE | TRUE | Gpha2 | 1 | 2.04E+08 | 2.04E+08 | + | protein_coding | NA |
| ENSRNOG00000025122 | FALSE | TRUE | AABR07005506.1 | 1 | 1.68E+08 | 1.68E+08 | - | protein_coding | NA |
| ENSRNOG00000039902 | FALSE | TRUE | Lbh | 6 | 22568834 | 22593079 | - | protein_coding | NA |
| ENSRNOG00000023683 | FALSE | TRUE | Nog | 10 | 74128712 | 74130339 | - | protein_coding | NA |
| ENSRNOG00000000507 | FALSE | TRUE | Tulp1 | 20 | 6412171 | 6424073 | - | protein_coding | Tub |
| ENSRNOG00000066823 | FALSE | TRUE | ENSRNOG00000066823 | 12 | 38391132 | 38409309 | - | lncRNA | NA |
| ENSRNOG00000008270 | FALSE | TRUE | Cntrob | 10 | 54022852 | 54044849 | - | protein_coding | NA |
| ENSRNOG00000064511 | FALSE | TRUE | Ankfn1 | 10 | 74218915 | 74663295 | - | protein_coding | NA |
| ENSRNOG00000001714 | FALSE | TRUE | Atp13a4 | 11 | 71226161 | 71359933 | + | protein_coding | NA |
| ENSRNOG00000016011 | FALSE | TRUE | Plekhg1 | 1 | 40311221 | 40434063 | + | protein_coding | NA |
| ENSRNOG00000037505 | FALSE | TRUE | Ulk1 | 12 | 45851710 | 45877966 | + | protein_coding | NA |
| ENSRNOG00000039740 | FALSE | TRUE | Cenpk | 2 | 35360132 | 35385706 | + | protein_coding | NA |
| ENSRNOG00000001345 | FALSE | TRUE | Mapkapk5 | 12 | 34994092 | 35013052 | + | protein_coding | NA |
| ENSRNOG00000022937 | FALSE | TRUE | Bpifc | 7 | 17861007 | 17905919 | + | protein_coding | NA |
| ENSRNOG00000062986 | FALSE | TRUE | Ccbe1 | 18 | 59580768 | 59824400 | - | protein_coding | NA |
| ENSRNOG00000028713 | FALSE | TRUE | Acvrl1 | 7 | 1.32E+08 | 1.32E+08 | + | protein_coding | NA |
| ENSRNOG00000057619 | FALSE | TRUE | Hic1 | 10 | 60011528 | 60019475 | - | protein_coding | ZBTB |
| ENSRNOG00000013690 | FALSE | TRUE | Clba1 | 6 | 1.32E+08 | 1.32E+08 | + | protein_coding | NA |
| ENSRNOG00000033143 | FALSE | TRUE | Cep295nl | 10 | 1.04E+08 | 1.04E+08 | - | protein_coding | NA |
| ENSRNOG00000032472 | FALSE | TRUE | Adgrg2 | X | 34297402 | 34422609 | - | protein_coding | NA |
| ENSRNOG00000039471 | FALSE | TRUE | Pcdhb16l | 18 | 29192124 | 29200539 | + | protein_coding | NA |
| ENSRNOG00000022784 | FALSE | TRUE | Fzd10 | 12 | 28098371 | 28101566 | - | protein_coding | NA |
| ENSRNOG00000028703 | FALSE | TRUE | Slc39a6 | 18 | 15864875 | 15885417 | - | protein_coding | NA |
| ENSRNOG00000061073 | FALSE | TRUE | Zfp275 | X | 1.51E+08 | 1.51E+08 | + | protein_coding | zf-C2H2 |
| ENSRNOG00000005154 | FALSE | TRUE | Alkal2 | 6 | 47495782 | 47505234 | + | protein_coding | NA |
| ENSRNOG00000030763 | FALSE | TRUE | Dpp4 | 3 | 46962243 | 47043901 | - | protein_coding | NA |
| ENSRNOG00000011134 | FALSE | TRUE | Lama2 | 1 | 17672536 | 18320530 | + | protein_coding | NA |
| ENSRNOG00000012735 | FALSE | TRUE | Kif24 | 5 | 56561154 | 56628025 | - | protein_coding | NA |
| ENSRNOG00000017538 | FALSE | TRUE | Lrrc27 | 1 | 1.94E+08 | 1.94E+08 | + | protein_coding | NA |
| ENSRNOG00000061450 | FALSE | TRUE | Homer2 | 1 | 1.36E+08 | 1.36E+08 | - | protein_coding | NA |
| ENSRNOG00000029805 | FALSE | TRUE | Zfp780b | 1 | 83160399 | 83183254 | + | protein_coding | zf-C2H2 |

| | | | | | | | | | |
|---|---|---|---|---|---|---|---|---|---|
| ENSRNOG00000003869 | FALSE | TRUE | Sod3 | 14 | 58609958 | 58615990 | - | protein_coding | NA |
| ENSRNOG00000025302 | FALSE | TRUE | Cdca2 | 15 | 41895901 | 41941611 | - | protein_coding | NA |
| ENSRNOG00000069775 | FALSE | TRUE | ENSRNOG00000069775 | 2 | 39508461 | 39526936 | - | lncRNA | NA |
| ENSRNOG00000043267 | FALSE | TRUE | Mrm2 | 12 | 14251941 | 14314118 | + | protein_coding | NA |
| ENSRNOG00000053075 | FALSE | TRUE | B4galnt4 | 1 | 1.96E+08 | 1.96E+08 | + | protein_coding | NA |
| ENSRNOG00000048195 | FALSE | TRUE | Nudt13 | 15 | 4001794 | 4022293 | - | protein_coding | NA |
| ENSRNOG00000069992 | FALSE | TRUE | Sgpp2 | 9 | 79763284 | 79880457 | + | protein_coding | NA |
| ENSRNOG00000015835 | FALSE | TRUE | Cacna2d2 | 8 | 1.08E+08 | 1.08E+08 | + | protein_coding | NA |
| ENSRNOG00000016534 | FALSE | TRUE | Fam171a1 | 17 | 75024575 | 75150255 | - | protein_coding | NA |
| ENSRNOG00000021540 | FALSE | TRUE | Rnf227 | 10 | 54059947 | 54063010 | + | protein_coding | NA |
| ENSRNOG00000061050 | FALSE | TRUE | Mboat2 | 6 | 41471161 | 41593485 | + | protein_coding | NA |
| ENSRNOG00000013970 | FALSE | TRUE | Cdt1 | 19 | 50620713 | 50625659 | + | protein_coding | NA |
| ENSRNOG00000027357 | FALSE | TRUE | Sox17 | 5 | 15016731 | 15022228 | + | protein_coding | HMG |
| ENSRNOG00000013847 | FALSE | TRUE | Nova2 | 1 | 78572279 | 78608021 | + | protein_coding | NA |
| ENSRNOG00000055991 | FALSE | TRUE | Tmprss11d | 14 | 21707336 | 21769396 | + | protein_coding | NA |
| ENSRNOG00000015921 | FALSE | TRUE | Esco2 | 15 | 40034568 | 40055306 | - | protein_coding | NA |
| ENSRNOG00000018944 | FALSE | TRUE | Pank1 | 1 | 2.32E+08 | 2.32E+08 | - | protein_coding | NA |
| ENSRNOG00000050562 | FALSE | TRUE | Zfp449 | X | 1.34E+08 | 1.34E+08 | + | protein_coding | zf-C2H2 |
| ENSRNOG00000033173 | FALSE | TRUE | Fam71e2 | 1 | 69077207 | 69095316 | + | protein_coding | NA |
| ENSRNOG00000059897 | FALSE | TRUE | Gjb5 | 5 | 1.4E+08 | 1.4E+08 | - | protein_coding | NA |
| ENSRNOG00000025742 | FALSE | TRUE | Lmnb2 | 7 | 8789314 | 8808665 | + | protein_coding | NA |
| ENSRNOG00000009957 | FALSE | TRUE | Slc25a13 | 4 | 34179224 | 34361902 | - | protein_coding | NA |
| ENSRNOG00000021053 | FALSE | TRUE | Lsr | 1 | 86186431 | 86201952 | - | protein_coding | NA |
| ENSRNOG00000014872 | FALSE | TRUE | Sec24d | 2 | 2.11E+08 | 2.12E+08 | + | protein_coding | NA |
| ENSRNOG00000068914 | FALSE | TRUE | Nrep | 18 | 25017083 | 25046591 | - | protein_coding | NA |
| ENSRNOG00000030880 | FALSE | TRUE | Hs6st2 | X | 1.31E+08 | 1.31E+08 | - | protein_coding | NA |
| ENSRNOG00000066026 | FALSE | TRUE | ENSRNOG00000066026 | 9 | 86275635 | 86287059 | + | lncRNA | NA |
| ENSRNOG00000001228 | FALSE | TRUE | Pofut2 | 20 | 11367096 | 11377743 | - | protein_coding | NA |
| ENSRNOG00000047098 | FALSE | TRUE | Hbb-b1 | 1 | 1.58E+08 | 1.58E+08 | - | protein_coding | NA |
| ENSRNOG00000049215 | FALSE | TRUE | Cbx2 | 10 | 1.04E+08 | 1.04E+08 | + | protein_coding | NA |
| ENSRNOG00000068617 | FALSE | TRUE | ENSRNOG00000068617 | 13 | 39245354 | 39251400 | - | lncRNA | NA |
| ENSRNOG00000017987 | FALSE | TRUE | Ucma | 17 | 73293978 | 73303611 | - | protein_coding | NA |
| ENSRNOG00000005781 | FALSE | TRUE | Wnt16 | 4 | 50820968 | 50831380 | + | protein_coding | NA |
| ENSRNOG00000033348 | FALSE | TRUE | Duox1 | 3 | 1.09E+08 | 1.09E+08 | + | protein_coding | NA |

| | | | | | | | | | |
|---|---|---|---|---|---|---|---|---|---|
| ENSRNOG00000024492 | FALSE | TRUE | Ap1ar | 2 | 2.16E+08 | 2.16E+08 | - | protein_coding | NA |
| ENSRNOG00000022146 | FALSE | TRUE | Hist1h2af | 17 | 41379404 | 41379796 | - | protein_coding | NA |
| ENSRNOG00000071035 | FALSE | TRUE | ENSRNOG00000071035 | 14 | 2664232 | 2665383 | + | lncRNA | NA |
| ENSRNOG00000057153 | FALSE | TRUE | Pla1a | 11 | 62320493 | 62357779 | + | protein_coding | NA |
| ENSRNOG00000007545 | FALSE | TRUE | Angptl4 | 7 | 14550311 | 14556519 | - | protein_coding | NA |
| ENSRNOG00000000981 | FALSE | TRUE | Scarb1 | 12 | 31296156 | 31362647 | + | protein_coding | NA |
| ENSRNOG00000007827 | FALSE | TRUE | Cox4i2 | 3 | 1.41E+08 | 1.41E+08 | + | protein_coding | NA |
| ENSRNOG00000026407 | FALSE | TRUE | Fam184a | 20 | 32934636 | 33056652 | - | protein_coding | NA |
| ENSRNOG00000023546 | FALSE | TRUE | Hspb1 | 12 | 20794028 | 20795743 | - | protein_coding | NA |
| ENSRNOG00000016013 | FALSE | TRUE | Gprc5b | 1 | 1.73E+08 | 1.73E+08 | - | protein_coding | NA |
| ENSRNOG00000000655 | FALSE | TRUE | Ptprc | 13 | 49596193 | 49708692 | - | protein_coding | NA |
| ENSRNOG00000014835 | FALSE | TRUE | Il1rl1 | 9 | 42697192 | 42727256 | + | protein_coding | NA |
| ENSRNOG00000001806 | FALSE | TRUE | Fetub | 11 | 78082156 | 78095135 | - | protein_coding | NA |
| ENSRNOG00000022957 | FALSE | TRUE | Ctxn3 | 18 | 50930235 | 50940641 | + | protein_coding | NA |
| ENSRNOG00000011332 | FALSE | TRUE | Clspn | 5 | 1.39E+08 | 1.39E+08 | + | protein_coding | NA |
| ENSRNOG00000016967 | FALSE | TRUE | Hfe | 17 | 41413451 | 41421502 | + | protein_coding | NA |
| ENSRNOG00000026053 | FALSE | TRUE | Grem1 | 3 | 1.01E+08 | 1.01E+08 | - | protein_coding | NA |
| ENSRNOG00000038955 | FALSE | TRUE | MGC105567 | 18 | 53870795 | 53892024 | + | protein_coding | NA |
| ENSRNOG00000015991 | FALSE | TRUE | Npr2 | 5 | 57883171 | 57901580 | + | protein_coding | NA |
| ENSRNOG00000027894 | FALSE | TRUE | Iqgap3 | 2 | 1.74E+08 | 1.74E+08 | + | protein_coding | NA |
| ENSRNOG00000020259 | FALSE | TRUE | RGD1305347 | 5 | 1.32E+08 | 1.32E+08 | + | protein_coding | NA |
| ENSRNOG00000001726 | FALSE | TRUE | Tmem44 | 11 | 70181345 | 70216958 | + | protein_coding | NA |
| ENSRNOG00000020588 | FALSE | TRUE | Efna4 | 2 | 1.75E+08 | 1.75E+08 | - | protein_coding | NA |
| ENSRNOG00000042950 | FALSE | TRUE | Slc5a9 | 5 | 1.27E+08 | 1.27E+08 | - | protein_coding | NA |
| ENSRNOG00000029520 | FALSE | TRUE | AC110315.1 | 15 | 50452312 | 50455724 | - | protein_coding | NA |
| ENSRNOG00000053239 | FALSE | TRUE | Clmp | 8 | 41060799 | 41168838 | + | protein_coding | NA |
| ENSRNOG00000010605 | FALSE | TRUE | Gsc | 6 | 1.23E+08 | 1.23E+08 | - | protein_coding | Homeobox |
| ENSRNOG00000000454 | FALSE | TRUE | RT1-DOb | 20 | 4619816 | 4625667 | - | protein_coding | NA |
| ENSRNOG00000001027 | FALSE | TRUE | Cd209f | 12 | 2426421 | 2428961 | - | protein_coding | NA |
| ENSRNOG00000009785 | FALSE | TRUE | Cdkn3 | 15 | 20022664 | 20033945 | + | protein_coding | NA |
| ENSRNOG00000008736 | FALSE | TRUE | Slamf8 | 13 | 85047727 | 85057211 | - | protein_coding | NA |
| ENSRNOG00000050262 | FALSE | TRUE | ENSRNOG00000050262 | 3 | 13033687 | 13037380 | + | protein_coding | NA |
| ENSRNOG00000019190 | FALSE | TRUE | Ankrd22 | 1 | 2.32E+08 | 2.32E+08 | - | protein_coding | NA |
| ENSRNOG00000024998 | FALSE | TRUE | Trps1 | 7 | 81921601 | 82141905 | - | protein_coding | zf-GATA |

| | | | | | | | | | |
|---|---|---|---|---|---|---|---|---|---|
| ENSRNOG00000004332 | FALSE | TRUE | Egfr | 14 | 91177067 | 91344382 | + | protein_coding | NA |
| ENSRNOG00000032178 | FALSE | TRUE | Cenpa | 6 | 25700142 | 25711675 | - | protein_coding | NA |
| ENSRNOG00000056135 | FALSE | TRUE | Tsc22d3 | X | 1.04E+08 | 1.04E+08 | - | protein_coding | TSC22 |
| ENSRNOG00000018092 | FALSE | TRUE | Cd83 | 17 | 20887309 | 20907083 | - | protein_coding | NA |
| ENSRNOG00000019428 | FALSE | TRUE | Higd1a | 8 | 1.22E+08 | 1.22E+08 | - | protein_coding | NA |
| ENSRNOG00000020743 | FALSE | TRUE | Cyp2s1 | 1 | 81310451 | 81325303 | - | protein_coding | NA |
| ENSRNOG00000008880 | FALSE | TRUE | Nrk | X | 1.02E+08 | 1.02E+08 | + | protein_coding | NA |
| ENSRNOG00000004205 | FALSE | TRUE | Pkdcc | 6 | 11455686 | 11465556 | - | protein_coding | NA |
| ENSRNOG00000020465 | FALSE | TRUE | Ripk3 | 15 | 29283145 | 29292121 | - | protein_coding | NA |
| ENSRNOG00000064417 | FALSE | TRUE | ENSRNOG00000064417 | 1 | 1.55E+08 | 1.55E+08 | - | lncRNA | NA |
| ENSRNOG00000016980 | FALSE | TRUE | Qprt | 1 | 1.82E+08 | 1.82E+08 | - | protein_coding | NA |
| ENSRNOG00000003984 | FALSE | TRUE | ENSRNOG00000003984 | X | 1.27E+08 | 1.27E+08 | - | lncRNA | NA |
| ENSRNOG00000018666 | FALSE | TRUE | Gpsm1 | 3 | 9128636 | 9167827 | + | protein_coding | NA |
| ENSRNOG00000007405 | FALSE | TRUE | Krba1 | 4 | 77340959 | 77363613 | + | protein_coding | NA |
| ENSRNOG00000009751 | FALSE | TRUE | Gpx7 | 5 | 1.23E+08 | 1.23E+08 | - | protein_coding | NA |
| ENSRNOG00000005770 | FALSE | TRUE | Sostdc1 | 6 | 53051354 | 53055579 | + | protein_coding | NA |
| ENSRNOG00000031521 | FALSE | TRUE | Cldn24 | 16 | 44424810 | 44425472 | - | protein_coding | NA |
| ENSRNOG00000032350 | FALSE | TRUE | Kcnip4 | 14 | 61381076 | 62530144 | + | protein_coding | NA |
| ENSRNOG00000000081 | FALSE | TRUE | Antxr2 | 14 | 11541772 | 11682094 | + | protein_coding | NA |
| ENSRNOG00000032473 | FALSE | TRUE | Scn10a | 8 | 1.19E+08 | 1.19E+08 | - | protein_coding | NA |
| ENSRNOG00000014505 | FALSE | TRUE | Pmfbp1 | 19 | 37458108 | 37507879 | + | protein_coding | NA |
| ENSRNOG00000033271 | FALSE | TRUE | AABR07048271.1 | 5 | 67671721 | 67680926 | + | protein_coding | NA |
| ENSRNOG00000013867 | FALSE | TRUE | Fgf1 | 18 | 30686581 | 30772357 | - | protein_coding | NA |
| ENSRNOG00000024028 | FALSE | TRUE | Sprr1a | 2 | 1.78E+08 | 1.78E+08 | - | protein_coding | NA |
| ENSRNOG00000068036 | FALSE | TRUE | ENSRNOG00000068036 | 3 | 1.1E+08 | 1.1E+08 | + | protein_coding | NA |
| ENSRNOG00000055996 | FALSE | TRUE | AABR07006032.1 | 1 | 1.97E+08 | 1.97E+08 | + | protein_coding | NA |
| ENSRNOG00000022286 | FALSE | TRUE | AABR07039316.1 | X | 67928231 | 67963076 | - | protein_coding | NA |
| ENSRNOG00000010350 | FALSE | TRUE | Rcan2 | 9 | 16959480 | 17174856 | - | protein_coding | NA |
| ENSRNOG00000014869 | FALSE | TRUE | Wdr31 | 5 | 75898353 | 75917417 | - | protein_coding | NA |
| ENSRNOG00000000137 | FALSE | TRUE | Ly86 | 17 | 27415830 | 27487260 | - | protein_coding | NA |
| ENSRNOG00000059322 | FALSE | TRUE | N4bp2l1 | 12 | 102573 | 127257 | - | protein_coding | NA |
| ENSRNOG00000002657 | FALSE | TRUE | Pla2g4a | 13 | 61877813 | 62022266 | - | protein_coding | NA |
| ENSRNOG00000046535 | FALSE | TRUE | Ppm1m | 8 | 1.07E+08 | 1.07E+08 | - | protein_coding | NA |
| ENSRNOG00000025711 | FALSE | TRUE | Spout1 | 3 | 13451202 | 13493355 | - | protein_coding | NA |

| | | | | | | | | | |
|---|---|---|---|---|---|---|---|---|---|
| ENSRNOG00000016505 | FALSE | TRUE | Fam169a | 2 | 28383784 | 28438910 | + | protein_coding | NA |
| ENSRNOG00000028902 | FALSE | TRUE | Spink14 | 18 | 36339223 | 36342144 | + | protein_coding | NA |
| ENSRNOG00000005655 | FALSE | TRUE | Cpa6 | 5 | 8526741 | 8888485 | + | protein_coding | NA |
| ENSRNOG00000025115 | FALSE | TRUE | Exph5 | 8 | 53698852 | 53773169 | + | protein_coding | NA |
| ENSRNOG00000001254 | FALSE | TRUE | Col6a2 | 20 | 12021767 | 12057564 | + | protein_coding | NA |
| ENSRNOG00000001338 | FALSE | TRUE | Hpd | 12 | 33381231 | 33392766 | + | protein_coding | NA |
| ENSRNOG00000028992 | FALSE | TRUE | Acan | 1 | 1.33E+08 | 1.33E+08 | + | protein_coding | NA |
| ENSRNOG00000002002 | FALSE | TRUE | Tmprss11b | 14 | 21383862 | 21408817 | + | protein_coding | NA |
| ENSRNOG00000049943 | FALSE | TRUE | Sinhcaf | 4 | 1.82E+08 | 1.82E+08 | - | protein_coding | NA |
| ENSRNOG00000008570 | FALSE | TRUE | Cadps | 15 | 12290262 | 12742779 | + | protein_coding | NA |
| ENSRNOG00000001167 | FALSE | TRUE | Ubash3a | 20 | 9292139 | 9329224 | + | protein_coding | NA |
| ENSRNOG00000009334 | FALSE | TRUE | Knstrn | 3 | 1.06E+08 | 1.06E+08 | + | protein_coding | NA |
| ENSRNOG00000068476 | FALSE | TRUE | RGD1560314 | 7 | 12164922 | 12180383 | - | protein_coding | zf-C2H2 |
| ENSRNOG00000020746 | FALSE | TRUE | Lin7b | 1 | 95846243 | 95849977 | - | protein_coding | NA |
| ENSRNOG00000056826 | FALSE | TRUE | Arap2 | 14 | 46718760 | 46917154 | + | protein_coding | NA |
| ENSRNOG00000030253 | FALSE | TRUE | Tafa3 | 2 | 1.92E+08 | 1.92E+08 | - | protein_coding | NA |
| ENSRNOG00000003554 | FALSE | TRUE | Piga | X | 30042343 | 30055804 | - | protein_coding | NA |
| ENSRNOG00000070875 | FALSE | TRUE | Nat8f4 | 4 | 1.18E+08 | 1.18E+08 | - | protein_coding | NA |
| ENSRNOG00000005195 | FALSE | TRUE | Cst3 | 3 | 1.36E+08 | 1.36E+08 | - | protein_coding | NA |
| ENSRNOG00000048924 | FALSE | TRUE | Islr | 8 | 58570777 | 58574130 | - | protein_coding | NA |
| ENSRNOG00000006782 | FALSE | TRUE | RGD1560028 | 4 | 94678370 | 94757034 | + | protein_coding | NA |
| ENSRNOG00000006778 | FALSE | TRUE | Mmp19 | 7 | 1221343 | 1229555 | + | protein_coding | NA |
| ENSRNOG00000049605 | FALSE | TRUE | Zfp296 | 1 | 79228401 | 79231866 | + | protein_coding | zf-C2H2 |
| ENSRNOG00000063259 | FALSE | TRUE | Krtap13-2 | 11 | 28089681 | 28090825 | + | protein_coding | NA |
| ENSRNOG00000021155 | FALSE | TRUE | Ctsk | 2 | 1.83E+08 | 1.83E+08 | + | protein_coding | NA |
| ENSRNOG00000021704 | FALSE | TRUE | Atp8b5p | 5 | 57506466 | 57620056 | + | protein_coding | NA |
| ENSRNOG00000005391 | FALSE | TRUE | Prex2 | 5 | 7942573 | 8253068 | - | protein_coding | NA |
| ENSRNOG00000008161 | FALSE | TRUE | Pdzk1ip1 | 5 | 1.29E+08 | 1.29E+08 | + | protein_coding | NA |
| ENSRNOG00000065112 | FALSE | TRUE | ENSRNOG00000065112 | 1 | 1.67E+08 | 1.67E+08 | + | protein_coding | NA |
| ENSRNOG00000015625 | FALSE | TRUE | MGC116202 | 19 | 33158056 | 33166445 | - | protein_coding | NA |
| ENSRNOG00000064875 | FALSE | TRUE | ENSRNOG00000064875 | 18 | 28314181 | 28314827 | + | protein_coding | NA |
| ENSRNOG00000003878 | FALSE | TRUE | Thsd7b | 13 | 40768570 | 41666501 | + | protein_coding | NA |
| ENSRNOG00000029244 | FALSE | TRUE | Pcdhb10 | 18 | 29117701 | 29122146 | + | protein_coding | NA |
| ENSRNOG00000023628 | FALSE | TRUE | Tmem106a | 10 | 86506936 | 86515892 | + | protein_coding | NA |

| | | | | | | | | | |
|---|---|---|---|---|---|---|---|---|---|
| ENSRNOG00000059741 | FALSE | TRUE | Tmc4 | 1 | 65539721 | 65551677 | + | protein_coding | NA |
| ENSRNOG00000022943 | FALSE | TRUE | Dgka | 7 | 1148735 | 1175110 | - | protein_coding | NA |
| ENSRNOG00000014320 | FALSE | TRUE | Inhba | 17 | 49095920 | 49108982 | - | protein_coding | NA |
| ENSRNOG00000007490 | FALSE | TRUE | Gabrr2 | 5 | 47452150 | 47489809 | + | protein_coding | NA |
| ENSRNOG00000016957 | FALSE | TRUE | Igfbp2 | 9 | 74415546 | 74442937 | + | protein_coding | NA |
| ENSRNOG00000058288 | FALSE | TRUE | Tcf19 | 20 | 3218693 | 3223271 | + | protein_coding | Others |
| ENSRNOG00000027035 | FALSE | TRUE | Sgo2 | 9 | 59548683 | 59573229 | + | protein_coding | NA |
| ENSRNOG00000016623 | FALSE | TRUE | Tmeff2 | 9 | 50436079 | 50733475 | - | protein_coding | NA |
| ENSRNOG00000063983 | FALSE | TRUE | Sox21 | 15 | 95292265 | 95296091 | - | protein_coding | HMG |
| ENSRNOG00000017927 | FALSE | TRUE | Drd4 | 1 | 1.96E+08 | 1.96E+08 | + | protein_coding | NA |
| ENSRNOG00000033688 | FALSE | TRUE | Cep170b | 6 | 1.32E+08 | 1.32E+08 | + | protein_coding | NA |
| ENSRNOG00000060087 | FALSE | TRUE | Adra1b | 10 | 28255025 | 28312919 | - | protein_coding | NA |
| ENSRNOG00000022838 | FALSE | TRUE | Cnksr1 | 5 | 1.46E+08 | 1.46E+08 | - | protein_coding | NA |
| ENSRNOG00000009884 | FALSE | TRUE | Lgals1 | 7 | 1.1E+08 | 1.1E+08 | + | protein_coding | NA |
| ENSRNOG00000046662 | FALSE | TRUE | Ints6l | X | 1.34E+08 | 1.34E+08 | + | protein_coding | NA |
| ENSRNOG00000018397 | FALSE | TRUE | Dnph1 | 9 | 14481066 | 14484022 | - | protein_coding | NA |
| ENSRNOG00000002417 | FALSE | TRUE | Serpinb10 | 13 | 23553430 | 23571182 | + | protein_coding | NA |
| ENSRNOG00000067479 | FALSE | TRUE | Hlf | 10 | 75154507 | 75211935 | - | protein_coding | TF_bZIP |
| ENSRNOG00000016541 | FALSE | TRUE | Enc1 | 2 | 28550464 | 28562713 | + | protein_coding | NA |
| ENSRNOG00000007551 | FALSE | TRUE | Patj | 5 | 1.13E+08 | 1.13E+08 | + | protein_coding | NA |
| ENSRNOG00000017955 | FALSE | TRUE | Nudt8 | 1 | 2.01E+08 | 2.01E+08 | + | protein_coding | NA |
| ENSRNOG00000067545 | FALSE | TRUE | ENSRNOG00000067545 | 7 | 1.33E+08 | 1.33E+08 | - | protein_coding | NA |
| ENSRNOG00000003802 | FALSE | TRUE | Pttg1 | 10 | 27893689 | 27904837 | - | protein_coding | NA |
| ENSRNOG00000012440 | FALSE | TRUE | Msra | 15 | 38351445 | 38676585 | - | protein_coding | NA |
| ENSRNOG00000016612 | FALSE | TRUE | Trim36 | 18 | 38861018 | 38912859 | - | protein_coding | NA |
| ENSRNOG00000017922 | FALSE | TRUE | Tpbgl | 1 | 1.54E+08 | 1.54E+08 | - | protein_coding | NA |
| ENSRNOG00000014928 | FALSE | TRUE | Apba1 | 1 | 2.21E+08 | 2.22E+08 | + | protein_coding | NA |
| ENSRNOG00000059894 | FALSE | TRUE | Hmmr | 10 | 25132977 | 25163060 | - | protein_coding | NA |
| ENSRNOG00000006966 | FALSE | TRUE | Nfia | 5 | 1.12E+08 | 1.13E+08 | + | protein_coding | CTF_NFI |
| ENSRNOG00000070694 | FALSE | TRUE | ENSRNOG00000070694 | 19 | 50320912 | 50322711 | + | protein_coding | zf-C2H2 |
| ENSRNOG00000023312 | FALSE | TRUE | Lonrf2 | 9 | 41034628 | 41070649 | - | protein_coding | NA |
| ENSRNOG00000008165 | FALSE | TRUE | Tpx2 | 3 | 1.41E+08 | 1.41E+08 | + | protein_coding | NA |
| ENSRNOG00000019112 | FALSE | TRUE | Chp2 | 1 | 1.77E+08 | 1.77E+08 | + | protein_coding | NA |
| ENSRNOG00000027433 | FALSE | TRUE | Akr1b8 | 4 | 62997161 | 63010097 | + | protein_coding | NA |

| | | | | | | | | | |
|---|---|---|---|---|---|---|---|---|---|
| ENSRNOG00000000925 | FALSE | TRUE | Psph | 12 | 26883133 | 26905074 | + | protein_coding | NA |
| ENSRNOG00000006952 | FALSE | TRUE | Prex1 | 3 | 1.55E+08 | 1.55E+08 | - | protein_coding | NA |
| ENSRNOG00000033893 | FALSE | TRUE | Cacna1h | 10 | 14390113 | 14448376 | - | protein_coding | NA |
| ENSRNOG00000016798 | FALSE | TRUE | Pla2g2f | 5 | 1.51E+08 | 1.51E+08 | - | protein_coding | NA |
| ENSRNOG00000008113 | FALSE | TRUE | RGD1561149 | 5 | 1.42E+08 | 1.42E+08 | - | protein_coding | NA |
| ENSRNOG00000053607 | FALSE | TRUE | Fam217b | 3 | 1.66E+08 | 1.66E+08 | + | protein_coding | NA |
| ENSRNOG00000042980 | FALSE | TRUE | Adam19 | 10 | 30491405 | 30583105 | + | protein_coding | NA |
| ENSRNOG00000066473 | FALSE | TRUE | ENSRNOG00000066473 | 17 | 42759961 | 42774981 | - | protein_coding | NA |
| ENSRNOG00000017939 | FALSE | TRUE | Chn1 | 3 | 58510536 | 58676490 | - | protein_coding | NA |
| ENSRNOG00000009191 | FALSE | TRUE | Ccm2l | 3 | 1.42E+08 | 1.42E+08 | + | protein_coding | NA |
| ENSRNOG00000013073 | FALSE | TRUE | Spink6 | 18 | 36397910 | 36409819 | + | protein_coding | NA |
| ENSRNOG00000065480 | FALSE | TRUE | Gpr17 | 18 | 23577242 | 23582966 | - | protein_coding | NA |
| ENSRNOG00000060356 | FALSE | TRUE | Kif15 | 8 | 1.23E+08 | 1.23E+08 | + | protein_coding | NA |
| ENSRNOG00000038035 | FALSE | TRUE | Kif4a | X | 65721779 | 65824139 | + | protein_coding | NA |
| ENSRNOG00000022686 | FALSE | TRUE | Zdhhc2 | 16 | 51808468 | 51878060 | - | protein_coding | NA |
| ENSRNOG00000032574 | FALSE | TRUE | Scrib | 7 | 1.08E+08 | 1.08E+08 | - | protein_coding | NA |
| ENSRNOG00000014592 | FALSE | TRUE | Zbtb46 | 3 | 1.68E+08 | 1.69E+08 | - | protein_coding | ZBTB |
| ENSRNOG00000026252 | FALSE | TRUE | E2f7 | 7 | 46151293 | 46192734 | + | protein_coding | E2F |
| ENSRNOG00000001143 | FALSE | TRUE | Cit | 12 | 40605563 | 40763860 | - | protein_coding | NA |
| ENSRNOG00000010888 | FALSE | TRUE | Ankrd33b | 2 | 82283108 | 82358872 | - | protein_coding | NA |
| ENSRNOG00000004657 | FALSE | TRUE | Sec23a | 6 | 76658427 | 76706035 | - | protein_coding | NA |
| ENSRNOG00000008209 | FALSE | TRUE | St3gal1 | 7 | 98845270 | 98913236 | - | protein_coding | NA |
| ENSRNOG00000013754 | FALSE | TRUE | Krt35 | 10 | 85026171 | 85029568 | - | protein_coding | NA |
| ENSRNOG00000024631 | FALSE | TRUE | Chadl | 7 | 1.13E+08 | 1.13E+08 | - | protein_coding | NA |
| ENSRNOG00000020254 | FALSE | TRUE | Per2 | 9 | 92007296 | 92049459 | - | protein_coding | NA |
| ENSRNOG00000003069 | FALSE | TRUE | Cd38 | 14 | 67172063 | 67211986 | - | protein_coding | NA |
| ENSRNOG00000016547 | FALSE | TRUE | Rgs19 | 3 | 1.69E+08 | 1.69E+08 | - | protein_coding | NA |
| ENSRNOG00000010645 | FALSE | TRUE | Lgals3 | 15 | 20607692 | 20632025 | + | protein_coding | NA |
| ENSRNOG00000057404 | FALSE | TRUE | Slc47a1 | 10 | 46034122 | 46087637 | - | protein_coding | NA |
| ENSRNOG00000012333 | FALSE | TRUE | Kbtbd11 | 16 | 74615792 | 74637736 | - | protein_coding | NA |
| ENSRNOG00000016408 | FALSE | TRUE | Kirrel1 | 2 | 1.73E+08 | 1.73E+08 | - | protein_coding | NA |
| ENSRNOG00000031342 | FALSE | TRUE | AABR07063082.1 | 6 | 1714435 | 1715952 | - | protein_coding | NA |
| ENSRNOG00000068587 | FALSE | TRUE | ENSRNOG00000068587 | 17 | 16058491 | 16085870 | - | lncRNA | NA |
| ENSRNOG00000068516 | FALSE | TRUE | Hist1h2ail1 | 17 | 41568471 | 41569109 | - | protein_coding | NA |

| | | | | | | | | | |
|---|---|---|---|---|---|---|---|---|---|
| ENSRNOG00000011334 | FALSE | TRUE | Tmem63c | 6 | 1.07E+08 | 1.07E+08 | + | protein_coding | NA |
| ENSRNOG00000038068 | FALSE | TRUE | Pcdh9 | 15 | 69340645 | 70237538 | - | protein_coding | NA |
| ENSRNOG00000004280 | FALSE | TRUE | Tcn2 | 14 | 78813343 | 78828489 | - | protein_coding | NA |
| ENSRNOG00000032929 | FALSE | TRUE | Incenp | 1 | 2.07E+08 | 2.07E+08 | - | protein_coding | NA |
| ENSRNOG00000003616 | FALSE | TRUE | Grem2 | 13 | 86778500 | 86871615 | - | protein_coding | NA |
| ENSRNOG00000027376 | FALSE | TRUE | Il17rc | 4 | 1.47E+08 | 1.47E+08 | + | protein_coding | NA |
| ENSRNOG00000008245 | FALSE | TRUE | AABR07054614.1 | 3 | 1.56E+08 | 1.56E+08 | - | protein_coding | NA |
| ENSRNOG00000006830 | FALSE | TRUE | Tgm6 | 3 | 1.17E+08 | 1.17E+08 | + | protein_coding | NA |
| ENSRNOG00000007078 | FALSE | TRUE | Ccn4 | 7 | 98645182 | 98677248 | + | protein_coding | NA |
| ENSRNOG00000012499 | FALSE | TRUE | Rarg | 7 | 1.33E+08 | 1.33E+08 | - | protein_coding | THR-like |
| ENSRNOG00000003095 | FALSE | TRUE | Fgfbp1 | 14 | 67104545 | 67107496 | + | protein_coding | NA |
| ENSRNOG00000018267 | FALSE | TRUE | B3gnt7 | 9 | 86956220 | 86960170 | + | protein_coding | NA |
| ENSRNOG00000066436 | FALSE | TRUE | ENSRNOG00000066436 | 4 | 13198317 | 13199409 | + | protein_coding | NA |
| ENSRNOG00000000767 | FALSE | TRUE | AC112568.1 | 20 | 1383424 | 1389406 | - | protein_coding | NA |
| ENSRNOG00000025987 | FALSE | TRUE | ENSRNOG00000025987 | 13 | 47630003 | 47665394 | - | protein_coding | NA |
| ENSRNOG00000033609 | FALSE | TRUE | Irx1 | 1 | 31294615 | 31300444 | + | protein_coding | Homeobox |
| ENSRNOG00000006729 | FALSE | TRUE | Slc24a4 | 6 | 1.21E+08 | 1.21E+08 | + | protein_coding | NA |
| ENSRNOG00000012438 | FALSE | TRUE | Larp6 | 8 | 61184116 | 61205535 | + | protein_coding | NA |
| ENSRNOG00000038202 | FALSE | TRUE | Calml4 | 8 | 63323351 | 63335234 | + | protein_coding | NA |
| ENSRNOG00000029095 | FALSE | TRUE | Trabd | 7 | 1.2E+08 | 1.2E+08 | + | protein_coding | NA |
| ENSRNOG00000020013 | FALSE | TRUE | Psrc1 | 2 | 1.96E+08 | 1.96E+08 | + | protein_coding | NA |
| ENSRNOG00000011644 | FALSE | TRUE | Slc1a7 | 5 | 1.23E+08 | 1.23E+08 | + | protein_coding | NA |
| ENSRNOG00000018839 | FALSE | TRUE | Ntrk2 | 17 | 5559043 | 5869136 | - | protein_coding | NA |
| ENSRNOG00000010866 | FALSE | TRUE | Tmem154 | 2 | 1.7E+08 | 1.7E+08 | + | protein_coding | NA |
| ENSRNOG00000004805 | FALSE | TRUE | Stac2 | 10 | 83032417 | 83048260 | - | protein_coding | NA |
| ENSRNOG00000009198 | FALSE | TRUE | Rab6b | 8 | 1.04E+08 | 1.04E+08 | + | protein_coding | NA |
| ENSRNOG00000070591 | FALSE | TRUE | H2bc18 | 2 | 1.84E+08 | 1.84E+08 | + | protein_coding | NA |
| ENSRNOG00000002624 | FALSE | TRUE | Edaradd | 17 | 85656905 | 85910447 | - | protein_coding | NA |
| ENSRNOG00000018952 | FALSE | TRUE | Sema3g | 16 | 6413589 | 6425221 | + | protein_coding | NA |
| ENSRNOG00000029205 | FALSE | TRUE | Zfp354c | 10 | 35132959 | 35145661 | - | protein_coding | zf-C2H2 |
| ENSRNOG00000052795 | FALSE | TRUE | Itpr3 | 20 | 5136441 | 5202337 | + | protein_coding | NA |
| ENSRNOG00000018715 | FALSE | TRUE | Clec10a | 10 | 54876260 | 54880435 | + | protein_coding | NA |
| ENSRNOG00000021338 | FALSE | TRUE | Tmem132a | 1 | 2.08E+08 | 2.08E+08 | - | protein_coding | NA |
| ENSRNOG00000020879 | FALSE | TRUE | Nags | 10 | 87098330 | 87102465 | + | protein_coding | NA |

| | | | | | | | | | |
|---|---|---|---|---|---|---|---|---|---|
| ENSRNOG00000007740 | FALSE | TRUE | Tacstd2 | 4 | 96707951 | 96709650 | - | protein_coding | NA |
| ENSRNOG00000022136 | FALSE | TRUE | BC016579 | 11 | 55166678 | 55192292 | + | protein_coding | NA |
| ENSRNOG00000015941 | FALSE | TRUE | Fkbp10 | 10 | 85346126 | 85427330 | + | protein_coding | NA |
| ENSRNOG00000028415 | FALSE | TRUE | Cdc20 | 5 | 1.32E+08 | 1.32E+08 | - | protein_coding | NA |
| ENSRNOG00000020614 | FALSE | TRUE | Phlda2 | 1 | 1.99E+08 | 1.99E+08 | - | protein_coding | NA |
| ENSRNOG00000008806 | FALSE | TRUE | Clip4 | 6 | 23616281 | 23677874 | - | protein_coding | NA |
| ENSRNOG00000031598 | FALSE | TRUE | Atp8b4 | 3 | 1.14E+08 | 1.14E+08 | - | protein_coding | NA |
| ENSRNOG00000053875 | FALSE | TRUE | Nacad | 14 | 81465299 | 81473781 | - | protein_coding | NA |
| ENSRNOG00000020349 | FALSE | TRUE | Rab3il1 | 1 | 2.07E+08 | 2.07E+08 | + | protein_coding | NA |
| ENSRNOG00000015520 | FALSE | TRUE | Ticrr | 1 | 1.34E+08 | 1.34E+08 | + | protein_coding | NA |
| ENSRNOG00000020948 | FALSE | TRUE | Pth1r | 8 | 1.11E+08 | 1.11E+08 | - | protein_coding | NA |
| ENSRNOG00000025551 | FALSE | TRUE | Rgs22 | 7 | 67188519 | 67308875 | - | protein_coding | NA |
| ENSRNOG00000028930 | FALSE | TRUE | Dab2 | 2 | 55514700 | 55567476 | + | protein_coding | NA |
| ENSRNOG00000043128 | FALSE | TRUE | Armcx4 | X | 97860629 | 97870912 | + | protein_coding | NA |
| ENSRNOG00000009339 | FALSE | TRUE | Cenpe | 2 | 2.24E+08 | 2.24E+08 | + | protein_coding | NA |
| ENSRNOG00000050152 | FALSE | TRUE | Chsy3 | 18 | 52644461 | 52898098 | + | protein_coding | NA |
| ENSRNOG00000014164 | FALSE | TRUE | Paqr5 | 8 | 62431867 | 62513688 | - | protein_coding | NA |
| ENSRNOG00000055650 | FALSE | TRUE | Pou2f2 | 1 | 80685741 | 80724261 | - | protein_coding | Pou |
| ENSRNOG00000020205 | FALSE | TRUE | Agrn | 5 | 1.67E+08 | 1.67E+08 | - | protein_coding | NA |
| ENSRNOG00000064648 | FALSE | TRUE | ENSRNOG00000064648 | 13 | 1.06E+08 | 1.07E+08 | + | lncRNA | NA |
| ENSRNOG00000013526 | FALSE | TRUE | Rassf4 | 4 | 1.5E+08 | 1.5E+08 | - | protein_coding | NA |
| ENSRNOG00000003284 | FALSE | TRUE | Epn3 | 10 | 79438978 | 79447356 | - | protein_coding | NA |
| ENSRNOG00000019962 | FALSE | TRUE | Sars2 | 1 | 84028986 | 84040725 | + | protein_coding | NA |
| ENSRNOG00000027630 | FALSE | TRUE | Dnai7 | 4 | 1.78E+08 | 1.78E+08 | - | protein_coding | NA |
| ENSRNOG00000022946 | FALSE | TRUE | Slc22a3 | 1 | 48235476 | 48324612 | + | protein_coding | NA |
| ENSRNOG00000033528 | FALSE | TRUE | Tll1 | 16 | 25509146 | 25709543 | + | protein_coding | NA |
| ENSRNOG00000002142 | FALSE | TRUE | Cds1 | 14 | 7820351 | 7882681 | - | protein_coding | NA |
| ENSRNOG00000007370 | FALSE | TRUE | Rnf144a | 6 | 42903174 | 43020012 | - | protein_coding | NA |
| ENSRNOG00000025676 | FALSE | TRUE | Gask1a | 8 | 1.22E+08 | 1.22E+08 | + | protein_coding | NA |
| ENSRNOG00000061182 | FALSE | TRUE | ENSRNOG00000061182 | X | 1.5E+08 | 1.5E+08 | - | protein_coding | NA |
| ENSRNOG00000036942 | FALSE | TRUE | Tnfrsf9 | 5 | 1.61E+08 | 1.61E+08 | + | protein_coding | NA |
| ENSRNOG00000017406 | FALSE | TRUE | Atrnl1 | 1 | 2.57E+08 | 2.57E+08 | + | protein_coding | NA |
| ENSRNOG00000071015 | FALSE | TRUE | Tlcd1 | 10 | 63074469 | 63076501 | + | protein_coding | NA |
| ENSRNOG00000015308 | FALSE | TRUE | Pbk | 15 | 40023002 | 40034014 | + | protein_coding | NA |

| | | | | | | | | | |
|---|---|---|---|---|---|---|---|---|---|
| ENSRNOG00000059326 | FALSE | TRUE | Abca9 | 10 | 95079058 | 95139608 | - | protein_coding | NA |
| ENSRNOG00000010436 | FALSE | TRUE | Zbtb17 | 5 | 1.54E+08 | 1.54E+08 | + | protein_coding | ZBTB |
| ENSRNOG00000061690 | FALSE | TRUE | Sycp2 | 3 | 1.65E+08 | 1.65E+08 | - | protein_coding | NA |
| ENSRNOG00000001452 | FALSE | TRUE | Fzd9 | 12 | 21427084 | 21429398 | + | protein_coding | NA |
| ENSRNOG00000054311 | FALSE | TRUE | ENSRNOG00000054311 | 12 | 36918224 | 36920511 | - | lncRNA | NA |
| ENSRNOG00000054474 | FALSE | TRUE | Mastl | 17 | 85251997 | 85287353 | - | protein_coding | NA |
| ENSRNOG00000002555 | FALSE | TRUE | Serpinb7 | 13 | 23395671 | 23442205 | + | protein_coding | NA |
| ENSRNOG00000018524 | FALSE | TRUE | Ezr | 1 | 46967658 | 47011505 | - | protein_coding | NA |
| ENSRNOG00000004127 | FALSE | TRUE | Cep170 | 13 | 88670358 | 88732226 | - | protein_coding | NA |
| ENSRNOG00000002255 | FALSE | TRUE | Fam162a | 11 | 64680323 | 64711239 | + | protein_coding | NA |
| ENSRNOG00000016697 | FALSE | TRUE | Lrrc56 | 1 | 1.96E+08 | 1.96E+08 | + | protein_coding | NA |
| ENSRNOG00000028187 | FALSE | TRUE | Sp9 | 3 | 58146826 | 58149357 | + | protein_coding | zf-C2H2 |
| ENSRNOG00000055300 | FALSE | TRUE | Ncapd2 | 4 | 1.58E+08 | 1.58E+08 | - | protein_coding | NA |
| ENSRNOG00000045683 | FALSE | TRUE | Mfap4 | 10 | 46167217 | 46170155 | + | protein_coding | NA |
| ENSRNOG00000010011 | FALSE | TRUE | Osbpl3 | 4 | 79350349 | 79523393 | - | protein_coding | NA |
| ENSRNOG00000008445 | FALSE | TRUE | Dact1 | 6 | 89790644 | 89817906 | + | protein_coding | NA |
| ENSRNOG00000032618 | FALSE | TRUE | Mst1r | 8 | 1.09E+08 | 1.09E+08 | + | protein_coding | NA |
| ENSRNOG00000036571 | FALSE | TRUE | Ces2c | 1 | 2.6E+08 | 2.6E+08 | + | protein_coding | NA |
| ENSRNOG00000046482 | FALSE | TRUE | Znf750 | 10 | 1.07E+08 | 1.07E+08 | - | protein_coding | Others |
| ENSRNOG00000066747 | FALSE | TRUE | ENSRNOG00000066747 | 14 | 94560541 | 94568345 | + | lncRNA | NA |
| ENSRNOG00000008182 | FALSE | TRUE | Htra3 | 14 | 75068917 | 75097315 | + | protein_coding | NA |
| ENSRNOG00000010646 | FALSE | TRUE | Tmem229b | 6 | 97775332 | 97819489 | - | protein_coding | NA |
| ENSRNOG00000039048 | FALSE | TRUE | Cd200r1 | 11 | 55887717 | 55921285 | - | protein_coding | NA |
| ENSRNOG00000012991 | FALSE | TRUE | Adgra2 | 16 | 64933315 | 64971483 | - | protein_coding | NA |
| ENSRNOG00000061857 | FALSE | TRUE | Mgst2 | 2 | 1.36E+08 | 1.36E+08 | + | protein_coding | NA |
| ENSRNOG00000014776 | FALSE | TRUE | Adcy7 | 19 | 18740875 | 18776311 | - | protein_coding | NA |
| ENSRNOG00000016758 | FALSE | TRUE | Loxl2 | 15 | 44683880 | 44773067 | + | protein_coding | NA |
| ENSRNOG00000003969 | FALSE | TRUE | Fam20a | 10 | 94642850 | 94697672 | - | protein_coding | NA |
| ENSRNOG00000065278 | FALSE | TRUE | ENSRNOG00000065278 | 2 | 1.89E+08 | 1.89E+08 | + | lncRNA | NA |
| ENSRNOG00000006617 | FALSE | TRUE | Cntnap2 | 4 | 74109472 | 76362027 | + | protein_coding | NA |
| ENSRNOG00000008865 | FALSE | TRUE | Stac | 8 | 1.12E+08 | 1.12E+08 | - | protein_coding | NA |
| ENSRNOG00000070470 | FALSE | TRUE | Krt6a | 7 | 1.33E+08 | 1.33E+08 | - | protein_coding | NA |
| ENSRNOG00000011654 | FALSE | TRUE | Plk4 | 2 | 1.24E+08 | 1.24E+08 | + | protein_coding | NA |
| ENSRNOG00000003018 | FALSE | TRUE | Olfml2b | 13 | 82869433 | 82906607 | + | protein_coding | NA |

| | | | | | | | | | |
|---|---|---|---|---|---|---|---|---|---|
| ENSRNOG00000006841 | FALSE | TRUE | Ano4 | 7 | 23329070 | 23738434 | - | protein_coding | NA |
| ENSRNOG00000024280 | FALSE | TRUE | Ccdc170 | 1 | 40945961 | 41085040 | + | protein_coding | NA |
| ENSRNOG00000004226 | FALSE | TRUE | Irak3 | 7 | 55653962 | 55713121 | - | protein_coding | NA |
| ENSRNOG00000011646 | FALSE | TRUE | Rem2 | 15 | 27933950 | 27938429 | + | protein_coding | NA |
| ENSRNOG00000049075 | FALSE | TRUE | ENSRNOG00000049075 | 2 | 91765056 | 91853773 | - | protein_coding | NA |
| ENSRNOG00000034012 | FALSE | TRUE | Klk12 | 1 | 94223045 | 94227637 | + | protein_coding | NA |
| ENSRNOG00000021745 | FALSE | TRUE | Bhlhe22 | 2 | 1E+08 | 1E+08 | + | protein_coding | bHLH |
| ENSRNOG00000039924 | FALSE | TRUE | Triqk | 5 | 26391552 | 26464421 | + | protein_coding | NA |
| ENSRNOG00000012140 | FALSE | TRUE | Cep89 | 1 | 88058227 | 88100112 | + | protein_coding | NA |
| ENSRNOG00000020777 | FALSE | TRUE | Zfp382 | 1 | 85377484 | 85399667 | + | protein_coding | zf-C2H2 |
| ENSRNOG00000023411 | FALSE | TRUE | Vsig10l | 1 | 93851774 | 93879566 | + | protein_coding | NA |
| ENSRNOG00000030930 | FALSE | TRUE | Samsn1 | 11 | 14483893 | 14534900 | - | protein_coding | NA |
| ENSRNOG00000005673 | FALSE | TRUE | Runx1t1 | 5 | 27187551 | 27335592 | + | protein_coding | NA |
| ENSRNOG00000064907 | FALSE | TRUE | ENSRNOG00000064907 | 3 | 8608999 | 8612279 | + | protein_coding | NA |
| ENSRNOG00000016873 | FALSE | TRUE | Msrb2 | 17 | 81974196 | 82000043 | + | protein_coding | NA |
| ENSRNOG00000007430 | FALSE | TRUE | Slx4ip | 3 | 1.24E+08 | 1.24E+08 | + | protein_coding | NA |
| ENSRNOG00000014863 | FALSE | TRUE | Reck | 5 | 58102981 | 58169502 | + | protein_coding | NA |
| ENSRNOG00000045654 | FALSE | TRUE | ENSRNOG00000045654 | 20 | 3856006 | 3873227 | - | protein_coding | NA |
| ENSRNOG00000010906 | FALSE | TRUE | Ccl5 | 10 | 68322829 | 68327377 | - | protein_coding | NA |
| ENSRNOG00000024650 | FALSE | TRUE | Ckap2 | 16 | 69839630 | 69864913 | + | protein_coding | NA |
| ENSRNOG00000069647 | FALSE | TRUE | ENSRNOG00000069647 | 15 | 908691 | 1007927 | + | protein_coding | NA |
| ENSRNOG00000003713 | FALSE | TRUE | Pbsn | X | 41914346 | 41929591 | - | protein_coding | NA |
| ENSRNOG00000008526 | FALSE | TRUE | Pdzd3 | 8 | 44584390 | 44588860 | - | protein_coding | NA |
| ENSRNOG00000012817 | FALSE | TRUE | Lrrc17 | 4 | 13501436 | 13531845 | - | protein_coding | NA |
| ENSRNOG00000031266 | FALSE | TRUE | Siae | 8 | 37318747 | 37353996 | + | protein_coding | NA |
| ENSRNOG00000029862 | FALSE | TRUE | Spc24 | 8 | 20300319 | 20305310 | - | protein_coding | NA |
| ENSRNOG00000016313 | FALSE | TRUE | Ctnnbip1 | 5 | 1.6E+08 | 1.6E+08 | + | protein_coding | NA |
| ENSRNOG00000002782 | FALSE | TRUE | Epgn | 14 | 17076774 | 17084806 | - | protein_coding | NA |
| ENSRNOG00000015582 | FALSE | TRUE | Me2 | 18 | 67355795 | 67400987 | - | protein_coding | NA |
| ENSRNOG00000012081 | FALSE | TRUE | Txn1 | 5 | 72711933 | 72724629 | - | protein_coding | NA |
| ENSRNOG00000019533 | FALSE | TRUE | Klhl3 | 17 | 6521912 | 6642154 | + | protein_coding | NA |
| ENSRNOG00000005375 | FALSE | TRUE | RGD1564053 | 5 | 7238853 | 7776769 | - | protein_coding | NA |
| ENSRNOG00000031805 | FALSE | TRUE | Krt36 | 10 | 85036594 | 85039959 | - | protein_coding | NA |
| ENSRNOG00000018434 | FALSE | TRUE | Stab1 | 16 | 6330444 | 6360923 | - | protein_coding | NA |

| | | | | | | | | | |
|---|---|---|---|---|---|---|---|---|---|
| ENSRNOG00000010970 | FALSE | TRUE | Krt24 | 10 | 84232164 | 84237299 | - | protein_coding | NA |
| ENSRNOG00000069945 | FALSE | TRUE | Tmem229a | 4 | 53443831 | 53444940 | - | protein_coding | NA |
| ENSRNOG00000050395 | FALSE | TRUE | Ms4a6bl | 1 | 2.08E+08 | 2.08E+08 | + | protein_coding | NA |
| ENSRNOG00000050946 | FALSE | TRUE | Fam110a | 3 | 1.4E+08 | 1.4E+08 | - | protein_coding | NA |
| ENSRNOG00000006236 | FALSE | TRUE | Dsn1 | 3 | 1.46E+08 | 1.46E+08 | - | protein_coding | NA |
| ENSRNOG00000043017 | FALSE | TRUE | Krtap7-1 | 11 | 28740390 | 28741003 | - | protein_coding | NA |
| ENSRNOG00000036829 | FALSE | TRUE | Nckap1l | 7 | 1.35E+08 | 1.35E+08 | + | protein_coding | NA |
| ENSRNOG00000008116 | FALSE | TRUE | Plpp3 | 5 | 1.2E+08 | 1.2E+08 | + | protein_coding | NA |
| ENSRNOG00000026527 | FALSE | TRUE | Snx32 | 1 | 2.03E+08 | 2.03E+08 | - | protein_coding | NA |
| ENSRNOG00000027739 | FALSE | TRUE | Cndp1 | 18 | 77984907 | 78007765 | - | protein_coding | NA |
| ENSRNOG00000012681 | FALSE | TRUE | Lgals9 | 10 | 63907018 | 63930045 | - | protein_coding | NA |
| ENSRNOG00000020482 | FALSE | TRUE | Nfatc4 | 15 | 29305535 | 29314610 | + | protein_coding | RHD |
| ENSRNOG00000065108 | FALSE | TRUE | Obp3 | 5 | 74842316 | 74845757 | - | protein_coding | NA |
| ENSRNOG00000066011 | FALSE | TRUE | 1700066B19Rik | 18 | 27325433 | 27327213 | + | protein_coding | NA |
| ENSRNOG00000016255 | FALSE | TRUE | Podxl2 | 4 | 1.21E+08 | 1.21E+08 | - | protein_coding | NA |
| ENSRNOG00000069978 | FALSE | TRUE | ENSRNOG00000069978 | 10 | 44167681 | 44185997 | + | lncRNA | NA |
| ENSRNOG00000021323 | FALSE | TRUE | Ca13 | 2 | 86928383 | 86959525 | - | protein_coding | NA |
| ENSRNOG00000046949 | FALSE | TRUE | Kcnb1 | 3 | 1.56E+08 | 1.56E+08 | - | protein_coding | NA |
| ENSRNOG00000011321 | FALSE | TRUE | Rftn1 | 9 | 10804611 | 11002084 | + | protein_coding | NA |
| ENSRNOG00000012557 | FALSE | TRUE | Lgals5 | 10 | 63853935 | 63857153 | - | protein_coding | NA |
| ENSRNOG00000016812 | FALSE | TRUE | Adamts16 | 1 | 32430436 | 32560494 | + | protein_coding | NA |
| ENSRNOG00000042286 | FALSE | TRUE | Nsl1 | 13 | 1.03E+08 | 1.03E+08 | + | protein_coding | NA |
| ENSRNOG00000066676 | FALSE | TRUE | ENSRNOG00000066676 | 4 | 11773595 | 11783462 | + | lncRNA | NA |
| ENSRNOG00000039649 | FALSE | TRUE | Lnp1 | 11 | 43654705 | 43678721 | + | protein_coding | NA |
| ENSRNOG00000009028 | FALSE | TRUE | Rnf126 | 7 | 9938229 | 9946738 | + | protein_coding | NA |
| ENSRNOG00000005971 | FALSE | TRUE | Gpr176 | 3 | 1.05E+08 | 1.05E+08 | - | protein_coding | NA |
| ENSRNOG00000028207 | FALSE | TRUE | Colgalt2 | 13 | 64523658 | 64627549 | + | protein_coding | NA |
| ENSRNOG00000000599 | FALSE | TRUE | Lama4 | 20 | 42392268 | 42533347 | + | protein_coding | NA |
| ENSRNOG00000017259 | FALSE | TRUE | Tacc3 | 14 | 77051215 | 77065219 | - | protein_coding | NA |
| ENSRNOG00000065666 | FALSE | TRUE | ENSRNOG00000065666 | 4 | 31319843 | 31342307 | - | protein_coding | NA |
| ENSRNOG00000027733 | FALSE | TRUE | Lair1 | 1 | 70157681 | 70185807 | + | protein_coding | NA |
| ENSRNOG00000009436 | FALSE | TRUE | Hemgn | 5 | 60679633 | 60698669 | - | protein_coding | NA |
| ENSRNOG00000018742 | FALSE | TRUE | Klk11 | 1 | 94228741 | 94233281 | + | protein_coding | NA |
| ENSRNOG00000008135 | FALSE | TRUE | Pla2g4f | 3 | 1.07E+08 | 1.07E+08 | - | protein_coding | NA |

| | | | | | | | | | |
|---|---|---|---|---|---|---|---|---|---|
| ENSRNOG00000045771 | FALSE | TRUE | Chl1 | 4 | 1.36E+08 | 1.37E+08 | + | protein_coding | NA |
| ENSRNOG00000021521 | FALSE | TRUE | Chst5 | 19 | 39860501 | 39881064 | - | protein_coding | NA |
| ENSRNOG00000022417 | FALSE | TRUE | Prickle3 | X | 14837650 | 14848218 | - | protein_coding | NA |
| ENSRNOG00000013641 | FALSE | TRUE | Myo7a | 1 | 1.52E+08 | 1.52E+08 | - | protein_coding | NA |
| ENSRNOG00000032946 | FALSE | TRUE | Pdzd7 | 1 | 2.44E+08 | 2.44E+08 | - | protein_coding | NA |
| ENSRNOG00000050553 | FALSE | TRUE | Atp6v1c2 | 6 | 40080547 | 40120028 | - | protein_coding | NA |
| ENSRNOG00000037845 | FALSE | TRUE | Cysltr1 | X | 71663821 | 71690121 | - | protein_coding | NA |
| ENSRNOG00000012015 | FALSE | TRUE | Lrrc49 | 8 | 61053267 | 61160850 | - | protein_coding | NA |
| ENSRNOG00000069480 | FALSE | TRUE | ENSRNOG00000069480 | 20 | 3310825 | 3421372 | - | protein_coding | NA |
| ENSRNOG00000065817 | FALSE | TRUE | Cep152 | 3 | 1.13E+08 | 1.13E+08 | - | protein_coding | NA |
| ENSRNOG00000016717 | FALSE | TRUE | Gas2 | 1 | 1.01E+08 | 1.02E+08 | + | protein_coding | NA |
| ENSRNOG00000013938 | FALSE | TRUE | Tshz3 | 1 | 89381858 | 89460874 | + | protein_coding | zf-C2H2 |
| ENSRNOG00000003242 | FALSE | TRUE | Gulp1 | 9 | 46622669 | 46899005 | + | protein_coding | NA |
| ENSRNOG00000005227 | FALSE | TRUE | Tfap4 | 10 | 11002911 | 11019386 | + | protein_coding | bHLH |
| ENSRNOG00000065587 | FALSE | TRUE | ENSRNOG00000065587 | 5 | 1.45E+08 | 1.45E+08 | + | lncRNA | NA |
| ENSRNOG00000015794 | FALSE | TRUE | Fam83d | 3 | 1.47E+08 | 1.47E+08 | + | protein_coding | NA |
| ENSRNOG00000001888 | FALSE | TRUE | Arvcf | 11 | 82587881 | 82645805 | - | protein_coding | NA |
| ENSRNOG00000018827 | FALSE | TRUE | Htr7 | 1 | 2.34E+08 | 2.34E+08 | - | protein_coding | NA |
| ENSRNOG00000029308 | FALSE | TRUE | Irag2 | 4 | 1.78E+08 | 1.78E+08 | + | protein_coding | NA |
| ENSRNOG00000067172 | FALSE | TRUE | ENSRNOG00000067172 | 1 | 2.15E+08 | 2.15E+08 | - | lncRNA | NA |
| ENSRNOG00000047940 | FALSE | TRUE | Mmp28 | 10 | 68241138 | 68264866 | - | protein_coding | NA |
| ENSRNOG00000026748 | FALSE | TRUE | Dennd2a | 4 | 68233958 | 68332105 | - | protein_coding | NA |
| ENSRNOG00000033087 | FALSE | TRUE | Cdh23 | 20 | 28240645 | 28622419 | - | protein_coding | NA |
| ENSRNOG00000011228 | FALSE | TRUE | Layn | 8 | 51367091 | 51384330 | - | protein_coding | NA |
| ENSRNOG00000021046 | FALSE | TRUE | Sult2b1 | 1 | 96200156 | 96261295 | - | protein_coding | NA |
| ENSRNOG00000029047 | FALSE | TRUE | Cubn | 17 | 76385060 | 76593231 | - | protein_coding | NA |
| ENSRNOG00000026212 | FALSE | TRUE | Micall1 | 7 | 1.11E+08 | 1.11E+08 | + | protein_coding | NA |
| ENSRNOG00000069198 | FALSE | TRUE | Lrmda | 15 | 1225710 | 2284749 | - | protein_coding | NA |
| ENSRNOG00000015957 | FALSE | TRUE | F13a1 | 17 | 27815702 | 27992700 | + | protein_coding | NA |
| ENSRNOG00000018915 | FALSE | TRUE | Tagap | 1 | 47170725 | 47179792 | - | protein_coding | NA |
| ENSRNOG00000010480 | FALSE | TRUE | Vstm5 | 8 | 12080203 | 12100427 | + | protein_coding | NA |
| ENSRNOG00000013003 | FALSE | TRUE | Srms | 3 | 1.68E+08 | 1.68E+08 | - | protein_coding | NA |
| ENSRNOG00000002152 | FALSE | TRUE | Dcun1d4 | 14 | 34652723 | 34733207 | - | protein_coding | NA |
| ENSRNOG00000002092 | FALSE | TRUE | Hunk | 11 | 29640775 | 29757526 | + | protein_coding | NA |

| | | | | | | | | | |
|---|---|---|---|---|---|---|---|---|---|
| ENSRNOG00000003765 | FALSE | TRUE | Nr0b1 | X | 50756886 | 50761011 | + | protein_coding | Miscellaneous |
| ENSRNOG00000010224 | FALSE | TRUE | Rab30 | 1 | 1.47E+08 | 1.47E+08 | + | protein_coding | NA |
| ENSRNOG00000017409 | FALSE | TRUE | Wnt6 | 9 | 76329882 | 76343523 | + | protein_coding | NA |
| ENSRNOG00000010447 | FALSE | TRUE | Tmod2 | 8 | 76325667 | 76365720 | - | protein_coding | NA |
| ENSRNOG00000020630 | FALSE | TRUE | Il9r | 10 | 15431706 | 15441990 | + | protein_coding | NA |
| ENSRNOG00000016613 | FALSE | TRUE | Hoxc4 | 7 | 1.34E+08 | 1.34E+08 | + | protein_coding | Homeobox |
| ENSRNOG00000060237 | FALSE | TRUE | Inhbb | 13 | 30530860 | 30537832 | - | protein_coding | NA |
| ENSRNOG00000010451 | FALSE | TRUE | Slc34a3 | 3 | 8044296 | 8049970 | - | protein_coding | NA |
| ENSRNOG00000018426 | FALSE | TRUE | NEWGENE_2134 | 1 | 79346136 | 79350375 | - | protein_coding | NA |
| ENSRNOG00000065909 | FALSE | TRUE | ENSRNOG00000065909 | 2 | 1.02E+08 | 1.02E+08 | + | lncRNA | NA |
| ENSRNOG00000064244 | FALSE | TRUE | ENSRNOG00000064244 | 1 | 58455453 | 58480021 | + | protein_coding | zf-C2H2 |
| ENSRNOG00000067844 | FALSE | TRUE | ENSRNOG00000067844 | X | 19563517 | 19572953 | - | protein_coding | NA |
| ENSRNOG00000011160 | FALSE | TRUE | Sv2b | 1 | 1.29E+08 | 1.29E+08 | - | protein_coding | NA |
| ENSRNOG00000034190 | FALSE | TRUE | Ighm | 6 | 1.32E+08 | 1.33E+08 | - | protein_coding | NA |
| ENSRNOG00000033608 | FALSE | TRUE | Cd276 | 8 | 58937751 | 58968380 | - | protein_coding | NA |
| ENSRNOG00000007118 | FALSE | TRUE | Eva1a | 4 | 1.15E+08 | 1.15E+08 | + | protein_coding | NA |
| ENSRNOG00000064486 | FALSE | TRUE | ENSRNOG00000064486 | 2 | 1.79E+08 | 1.79E+08 | + | protein_coding | NA |
| ENSRNOG00000070246 | FALSE | TRUE | ENSRNOG00000070246 | 12 | 15642295 | 15644220 | + | lncRNA | NA |
| ENSRNOG00000054022 | FALSE | TRUE | Eddm3b | 15 | 24345573 | 24346025 | + | protein_coding | NA |
| ENSRNOG00000015285 | FALSE | TRUE | Lrp4 | 3 | 77429798 | 77483593 | + | protein_coding | NA |
| ENSRNOG00000007151 | FALSE | TRUE | Cdk14 | 4 | 28666843 | 29258861 | + | protein_coding | NA |
| ENSRNOG00000064228 | FALSE | TRUE | ENSRNOG00000064228 | 12 | 18601158 | 18651608 | - | protein_coding | NA |
| ENSRNOG00000014838 | FALSE | TRUE | Glipr2 | 5 | 58170425 | 58202272 | + | protein_coding | NA |
| ENSRNOG00000070099 | FALSE | TRUE | Crb3 | 9 | 1896623 | 1903859 | + | protein_coding | NA |
| ENSRNOG00000027491 | FALSE | TRUE | Vldlr | 1 | 2.25E+08 | 2.25E+08 | + | protein_coding | NA |
| ENSRNOG00000004084 | FALSE | TRUE | Lratd1 | 6 | 36913469 | 36922319 | - | protein_coding | NA |
| ENSRNOG00000019620 | FALSE | TRUE | Pmf1 | 2 | 1.74E+08 | 1.74E+08 | - | protein_coding | NA |
| ENSRNOG00000020703 | FALSE | TRUE | Sipa1l3 | 1 | 84618719 | 84703802 | - | protein_coding | NA |
| ENSRNOG00000047951 | FALSE | TRUE | Hoxa5 | 4 | 81302353 | 81306234 | - | protein_coding | Homeobox |
| ENSRNOG00000042407 | FALSE | TRUE | Obp2a | 3 | 8505990 | 8509269 | + | protein_coding | NA |
| ENSRNOG00000003657 | FALSE | TRUE | Pkmyt1 | 10 | 12748237 | 12758995 | + | protein_coding | NA |
| ENSRNOG00000022623 | FALSE | TRUE | Ttll12 | 7 | 1.15E+08 | 1.15E+08 | - | protein_coding | NA |
| ENSRNOG00000023633 | FALSE | TRUE | Crabp1 | 8 | 55151285 | 55159360 | + | protein_coding | NA |

| | | | | | | | | | |
|---|---|---|---|---|---|---|---|---|---|
| ENSRNOG00000017803 | FALSE | TRUE | Apbb1ip | 17 | 84982243 | 85033010 | + | protein_coding | NA |
| ENSRNOG00000021170 | FALSE | TRUE | Plekho1 | 2 | 1.84E+08 | 1.84E+08 | - | protein_coding | NA |
| ENSRNOG00000019271 | FALSE | TRUE | Ulk4 | 8 | 1.21E+08 | 1.21E+08 | - | protein_coding | NA |
| ENSRNOG00000015810 | FALSE | TRUE | Trip13 | 1 | 29357130 | 29402074 | + | protein_coding | NA |
| ENSRNOG00000055962 | FALSE | TRUE | Bgn | X | 1.51E+08 | 1.51E+08 | + | protein_coding | NA |
| ENSRNOG00000064067 | FALSE | TRUE | ENSRNOG00000064067 | 7 | 1.3E+08 | 1.3E+08 | - | lncRNA | NA |
| ENSRNOG00000048723 | FALSE | TRUE | Pros1 | 11 | 230696 | 311286 | + | protein_coding | NA |
| ENSRNOG00000014323 | FALSE | TRUE | Extl2 | 2 | 2.04E+08 | 2.04E+08 | + | protein_coding | NA |
| ENSRNOG00000004479 | FALSE | TRUE | Aurka | 3 | 1.61E+08 | 1.61E+08 | - | protein_coding | NA |
| ENSRNOG00000016550 | FALSE | TRUE | Dclk2 | 2 | 1.72E+08 | 1.72E+08 | - | protein_coding | NA |
| ENSRNOG00000010747 | FALSE | TRUE | Dap | 2 | 82199280 | 82251752 | + | protein_coding | NA |
| ENSRNOG00000004668 | FALSE | TRUE | Slc25a35 | 10 | 53673340 | 53698160 | + | protein_coding | NA |
| ENSRNOG00000031955 | FALSE | TRUE | Calml3 | 17 | 66419882 | 66423175 | + | protein_coding | NA |
| ENSRNOG00000017850 | FALSE | TRUE | Dctpp1 | 1 | 1.82E+08 | 1.82E+08 | - | protein_coding | NA |
| ENSRNOG00000011424 | FALSE | TRUE | Cldn23 | 16 | 56413381 | 56415523 | + | protein_coding | NA |
| ENSRNOG00000000451 | FALSE | TRUE | RT1-Ba | 20 | 4575134 | 4579744 | + | protein_coding | NA |
| ENSRNOG00000019220 | FALSE | TRUE | Rhod | 1 | 2.02E+08 | 2.02E+08 | - | protein_coding | NA |
| ENSRNOG00000024420 | FALSE | TRUE | Eme1 | 10 | 79586729 | 79595435 | - | protein_coding | NA |
| ENSRNOG00000011777 | FALSE | TRUE | Spag5 | 10 | 63198768 | 63216745 | + | protein_coding | NA |
| ENSRNOG0000024918 | FALSE | TRUE | Fam229b | 20 | 42538868 | 42552248 | - | protein_coding | NA |
| ENSRNOG00000014658 | FALSE | TRUE | Zfp423 | 19 | 19110238 | 19407373 | + | protein_coding | zf-C2H2 |
| ENSRNOG00000067366 | FALSE | TRUE | ENSRNOG00000067366 | 15 | 43976207 | 43982932 | - | protein_coding | NA |
| ENSRNOG00000012544 | FALSE | TRUE | Krtap3-1 | 10 | 84527448 | 84528041 | - | protein_coding | NA |
| ENSRNOG00000066201 | FALSE | TRUE | Lgi2 | 14 | 58415965 | 58443407 | + | protein_coding | NA |
| ENSRNOG00000017332 | FALSE | TRUE | Dapk2 | 8 | 66706609 | 66825567 | + | protein_coding | NA |
| ENSRNOG00000047699 | FALSE | TRUE | Snai2 | 11 | 86181909 | 86186200 | - | protein_coding | zf-C2H2 |
| ENSRNOG00000063556 | FALSE | TRUE | ENSRNOG00000063556 | 9 | 59008776 | 59040615 | + | lncRNA | NA |
| ENSRNOG00000021137 | FALSE | TRUE | Kctd15 | 1 | 87258658 | 87273497 | - | protein_coding | NA |
| ENSRNOG00000026466 | FALSE | TRUE | Lrrtm3 | 20 | 24515627 | 24689669 | + | protein_coding | NA |
| ENSRNOG00000001578 | FALSE | TRUE | Hoxd4 | 3 | 59614464 | 59631528 | + | protein_coding | Homeobox |
| ENSRNOG00000031789 | FALSE | TRUE | Rangap1 | 7 | 1.13E+08 | 1.13E+08 | - | protein_coding | NA |
| ENSRNOG00000069880 | FALSE | TRUE | ENSRNOG00000069880 | 8 | 48502706 | 48590177 | + | lncRNA | NA |
| ENSRNOG00000019915 | FALSE | TRUE | Xrcc1 | 1 | 80141207 | 80168701 | + | protein_coding | NA |
| ENSRNOG00000010121 | FALSE | TRUE | Lef1 | 2 | 2.2E+08 | 2.2E+08 | + | protein_coding | HMG |

| | | | | | | | | | |
|---|---|---|---|---|---|---|---|---|---|
| ENSRNOG00000000341 | FALSE | TRUE | Nid2 | 15 | 4458084 | 4513843 | - | protein_coding | NA |
| ENSRNOG00000002331 | FALSE | TRUE | Aldh3a1 | 10 | 45892924 | 45902681 | + | protein_coding | NA |
| ENSRNOG00000009574 | FALSE | TRUE | Crct1 | 2 | 1.79E+08 | 1.79E+08 | - | protein_coding | NA |
| ENSRNOG00000006996 | FALSE | TRUE | Mms22l | 5 | 38197779 | 38313745 | + | protein_coding | NA |
| ENSRNOG00000019005 | FALSE | TRUE | Pde8a | 1 | 1.35E+08 | 1.35E+08 | + | protein_coding | NA |
| ENSRNOG00000042369 | FALSE | TRUE | RGD1564614 | 13 | 51422592 | 51491502 | - | protein_coding | NA |
| ENSRNOG00000064205 | FALSE | TRUE | St8sia6 | 17 | 76745224 | 76884299 | - | protein_coding | NA |
| ENSRNOG00000010240 | FALSE | TRUE | Tent5a | 8 | 86225357 | 86229045 | - | protein_coding | NA |
| ENSRNOG00000010701 | FALSE | TRUE | Depdc1b | 2 | 39891481 | 39963779 | + | protein_coding | NA |
| ENSRNOG00000016888 | FALSE | TRUE | Pask | 9 | 93844278 | 93885111 | - | protein_coding | NA |
| ENSRNOG00000005082 | FALSE | TRUE | Irf6 | 13 | 1.05E+08 | 1.05E+08 | + | protein_coding | IRF |
| ENSRNOG00000015788 | FALSE | TRUE | Adora3 | 2 | 1.93E+08 | 1.93E+08 | + | protein_coding | NA |
| ENSRNOG00000028194 | FALSE | TRUE | Serpinb13 | 13 | 23118584 | 23150760 | + | protein_coding | NA |
| ENSRNOG00000067329 | FALSE | TRUE | ENSRNOG00000067329 | 1 | 20935300 | 20948353 | + | lncRNA | NA |
| ENSRNOG00000017468 | FALSE | TRUE | Trappc6a | 1 | 79158529 | 79165493 | + | protein_coding | NA |
| ENSRNOG00000061813 | FALSE | TRUE | Ly6c | 7 | 1.07E+08 | 1.07E+08 | - | protein_coding | NA |
| ENSRNOG00000009773 | FALSE | TRUE | Elovl4 | 8 | 84702362 | 84729697 | - | protein_coding | NA |
| ENSRNOG00000007335 | FALSE | TRUE | Ccl11 | 10 | 67028328 | 67032926 | + | protein_coding | NA |
| ENSRNOG00000002852 | FALSE | TRUE | Myo19 | 10 | 69753068 | 69782450 | + | protein_coding | NA |
| ENSRNOG00000021555 | FALSE | TRUE | Mis18a | 11 | 29967701 | 29981062 | - | protein_coding | NA |
| ENSRNOG00000050983 | FALSE | TRUE | Hpcal4 | 5 | 1.35E+08 | 1.35E+08 | + | protein_coding | NA |
| ENSRNOG00000009331 | FALSE | TRUE | Hck | 3 | 1.42E+08 | 1.42E+08 | + | protein_coding | NA |
| ENSRNOG00000009912 | FALSE | TRUE | Fgr | 5 | 1.45E+08 | 1.45E+08 | + | protein_coding | NA |
| ENSRNOG00000016252 | FALSE | TRUE | Mblac2 | 2 | 11986027 | 12030950 | + | protein_coding | NA |
| ENSRNOG00000046996 | FALSE | TRUE | Pea15 | 13 | 84654870 | 84667499 | - | protein_coding | NA |
| ENSRNOG00000000156 | FALSE | TRUE | Megf6 | 5 | 1.65E+08 | 1.65E+08 | + | protein_coding | NA |
| ENSRNOG00000033883 | FALSE | TRUE | Stard8 | X | 64124574 | 64196052 | + | protein_coding | NA |
| ENSRNOG00000000288 | FALSE | TRUE | Scarf2 | 11 | 83175963 | 83187348 | - | protein_coding | NA |
| ENSRNOG00000018257 | FALSE | TRUE | Hpx | 1 | 1.6E+08 | 1.6E+08 | - | protein_coding | NA |
| ENSRNOG00000067163 | FALSE | TRUE | ENSRNOG00000067163 | 1 | 2.25E+08 | 2.25E+08 | - | lncRNA | NA |
| ENSRNOG00000000262 | FALSE | TRUE | Zfp821 | 19 | 37680628 | 37698831 | + | protein_coding | NA |
| ENSRNOG00000026928 | FALSE | TRUE | RGD1560402 | 2 | 42754639 | 42755856 | + | protein_coding | NA |
| ENSRNOG00000063920 | FALSE | TRUE | ENSRNOG00000063920 | 17 | 31540786 | 31555653 | + | lncRNA | NA |
| ENSRNOG00000005667 | FALSE | TRUE | Astn1 | 13 | 70537703 | 70855440 | + | protein_coding | NA |

| | | | | | | | | | |
|---|---|---|---|---|---|---|---|---|---|
| ENSRNOG00000009298 | FALSE | TRUE | Fbxo44 | 5 | 1.59E+08 | 1.59E+08 | - | protein_coding | NA |
| ENSRNOG00000023548 | FALSE | TRUE | Sned1 | 9 | 93774119 | 93830694 | + | protein_coding | NA |
| ENSRNOG00000068890 | FALSE | TRUE | ENSRNOG00000068890 | 15 | 43959219 | 43970923 | - | lncRNA | NA |
| ENSRNOG00000018019 | FALSE | TRUE | Hspa12a | 1 | 2.58E+08 | 2.58E+08 | - | protein_coding | NA |
| ENSRNOG00000003183 | FALSE | TRUE | Fmod | 13 | 45493517 | 45504133 | + | protein_coding | NA |
| ENSRNOG00000016384 | FALSE | TRUE | Pik3cb | 8 | 99594644 | 99699663 | - | protein_coding | NA |
| ENSRNOG00000000167 | FALSE | TRUE | Alas2 | X | 19463171 | 19486519 | + | protein_coding | NA |
| ENSRNOG00000048004 | FALSE | TRUE | Garem2 | 6 | 26230048 | 26241762 | - | protein_coding | NA |
| ENSRNOG00000032922 | FALSE | TRUE | Dclk1 | 2 | 1.39E+08 | 1.4E+08 | + | protein_coding | NA |
| ENSRNOG00000046468 | FALSE | TRUE | Ptgfr | 2 | 2.41E+08 | 2.41E+08 | - | protein_coding | NA |
| ENSRNOG00000066242 | FALSE | TRUE | Rtp4 | 11 | 77238216 | 77335437 | - | protein_coding | NA |
| ENSRNOG00000018414 | FALSE | TRUE | Csf1r | 18 | 54546659 | 54590415 | + | protein_coding | NA |
| ENSRNOG00000071076 | FALSE | TRUE | ENSRNOG00000071076 | 6 | 1.23E+08 | 1.23E+08 | + | lncRNA | NA |
| ENSRNOG00000009372 | FALSE | TRUE | Tacr3 | 2 | 2.23E+08 | 2.23E+08 | + | protein_coding | NA |
| ENSRNOG00000021098 | FALSE | TRUE | Rasgrp2 | 1 | 2.04E+08 | 2.04E+08 | + | protein_coding | NA |
| ENSRNOG00000011167 | FALSE | TRUE | Wdhd1 | 15 | 20489291 | 20523096 | - | protein_coding | HMG |
| ENSRNOG00000007907 | FALSE | TRUE | Tmem178a | 6 | 14004630 | 14063549 | - | protein_coding | NA |
| ENSRNOG00000047692 | FALSE | TRUE | Hells | 1 | 2.37E+08 | 2.37E+08 | + | protein_coding | NA |
| ENSRNOG00000004459 | FALSE | TRUE | Sdr9c7 | 7 | 63707071 | 63721480 | + | protein_coding | NA |
| ENSRNOG00000010275 | FALSE | TRUE | Slc17a9 | 3 | 1.68E+08 | 1.68E+08 | + | protein_coding | NA |
| ENSRNOG00000017459 | FALSE | TRUE | C1ql3 | 17 | 76119447 | 76128530 | - | protein_coding | NA |
| ENSRNOG00000042163 | FALSE | TRUE | Btbd19 | 5 | 1.31E+08 | 1.31E+08 | - | protein_coding | NA |
| ENSRNOG00000011971 | FALSE | TRUE | C1s | 4 | 1.57E+08 | 1.57E+08 | - | protein_coding | NA |
| ENSRNOG00000021824 | FALSE | TRUE | Dnajb1 | 19 | 24522717 | 24526458 | - | protein_coding | NA |
| ENSRNOG00000016210 | FALSE | TRUE | Micalcl | 1 | 1.66E+08 | 1.67E+08 | + | protein_coding | NA |
| ENSRNOG00000043331 | FALSE | TRUE | Ankrd65 | 5 | 1.66E+08 | 1.66E+08 | + | protein_coding | NA |
| ENSRNOG00000017693 | FALSE | TRUE | Slc2a5 | 5 | 1.61E+08 | 1.61E+08 | + | protein_coding | NA |
| ENSRNOG00000013408 | FALSE | TRUE | Npas2 | 9 | 41463830 | 41642320 | + | protein_coding | bHLH |
| ENSRNOG00000003304 | FALSE | TRUE | Chad | 10 | 79511931 | 79515940 | + | protein_coding | NA |
| ENSRNOG00000017895 | FALSE | TRUE | Eno1 | 5 | 1.61E+08 | 1.61E+08 | + | protein_coding | NA |
| ENSRNOG00000034184 | FALSE | TRUE | Zfp583 | 1 | 67575854 | 67594476 | + | protein_coding | zf-C2H2 |
| ENSRNOG00000020468 | FALSE | TRUE | Stard4 | 18 | 24817107 | 24830024 | - | protein_coding | NA |
| ENSRNOG00000007346 | FALSE | TRUE | Tamalin | 7 | 1.32E+08 | 1.32E+08 | + | protein_coding | NA |
| ENSRNOG00000011648 | FALSE | TRUE | Aqp1 | 4 | 84482512 | 84494690 | + | protein_coding | NA |

| | | | | | | | | | |
|---|---|---|---|---|---|---|---|---|---|
| ENSRNOG00000030800 | FALSE | TRUE | Igdcc3 | 8 | 65661196 | 65707959 | + | protein_coding | NA |
| ENSRNOG00000056894 | FALSE | TRUE | St6galnac3 | 2 | 2.42E+08 | 2.43E+08 | - | protein_coding | NA |
| ENSRNOG00000061231 | FALSE | TRUE | Selenom | 14 | 78395826 | 78398429 | + | protein_coding | NA |
| ENSRNOG00000070954 | FALSE | TRUE | Clec3a | 19 | 42404628 | 42412008 | + | protein_coding | NA |
| ENSRNOG00000009967 | FALSE | TRUE | Otof | 6 | 25928055 | 26024631 | + | protein_coding | NA |
| ENSRNOG00000057827 | FALSE | TRUE | Lmln2 | 15 | 28151019 | 28158129 | - | protein_coding | NA |
| ENSRNOG00000068560 | FALSE | TRUE | ENSRNOG00000068560 | 11 | 42122492 | 42125698 | - | lncRNA | NA |
| ENSRNOG00000042460 | FALSE | TRUE | Hmga2 | 7 | 55880112 | 55994784 | - | protein_coding | HMGA |
| ENSRNOG00000011791 | FALSE | TRUE | Zfand4 | 4 | 1.49E+08 | 1.49E+08 | + | protein_coding | NA |
| ENSRNOG00000016769 | FALSE | TRUE | Rab38 | 1 | 1.42E+08 | 1.42E+08 | + | protein_coding | NA |
| ENSRNOG00000067167 | FALSE | TRUE | Tmem263 | 7 | 18610149 | 18628534 | - | protein_coding | NA |
| ENSRNOG00000013945 | FALSE | TRUE | Itpkc | 1 | 82500957 | 82522779 | - | protein_coding | NA |
| ENSRNOG00000001001 | FALSE | TRUE | Retn | 12 | 1710881 | 1712620 | + | protein_coding | NA |
| ENSRNOG00000014378 | FALSE | TRUE | Il1r2 | 9 | 42384433 | 42424725 | + | protein_coding | NA |
| ENSRNOG00000042189 | FALSE | TRUE | Rab31 | 9 | 1.05E+08 | 1.05E+08 | - | protein_coding | NA |
| ENSRNOG00000066223 | FALSE | TRUE | Cby3 | 10 | 34677770 | 34682784 | + | protein_coding | NA |
| ENSRNOG00000015253 | FALSE | TRUE | Hsf4 | 19 | 33147755 | 33153479 | + | protein_coding | HSF |
| ENSRNOG00000064826 | FALSE | TRUE | ENSRNOG00000064826 | 2 | 1.74E+08 | 1.74E+08 | - | lncRNA | NA |
| ENSRNOG00000024688 | FALSE | TRUE | Erfe | 9 | 91956977 | 91964846 | + | protein_coding | NA |
| ENSRNOG00000037149 | FALSE | TRUE | Bcl2l15 | 2 | 1.91E+08 | 1.91E+08 | + | protein_coding | NA |
| ENSRNOG00000016728 | FALSE | TRUE | Tiam2 | 1 | 43918174 | 44119493 | + | protein_coding | NA |
| ENSRNOG00000070387 | FALSE | TRUE | Serpinb3 | 13 | 23274484 | 23313682 | - | protein_coding | NA |
| ENSRNOG00000014721 | FALSE | TRUE | Ahrr | 1 | 28993634 | 29088673 | + | protein_coding | bHLH |
| ENSRNOG00000063669 | FALSE | TRUE | Stfa2l2 | 11 | 64498206 | 64500574 | + | protein_coding | NA |
| ENSRNOG00000005542 | FALSE | TRUE | Apob | 6 | 30844368 | 30892497 | + | protein_coding | NA |
| ENSRNOG00000006327 | FALSE | TRUE | Rcc2 | 5 | 1.53E+08 | 1.53E+08 | + | protein_coding | NA |
| ENSRNOG00000069388 | FALSE | TRUE | ENSRNOG00000069388 | 2 | 1.78E+08 | 1.78E+08 | - | protein_coding | NA |
| ENSRNOG00000020330 | FALSE | TRUE | Wdr47 | 2 | 1.96E+08 | 1.96E+08 | + | protein_coding | NA |
| ENSRNOG00000004195 | FALSE | TRUE | Dtl | 13 | 1.03E+08 | 1.03E+08 | - | protein_coding | NA |
| ENSRNOG00000033933 | FALSE | TRUE | Stfa3 | 11 | 64353720 | 64358862 | + | protein_coding | NA |
| ENSRNOG00000008930 | FALSE | TRUE | Tnfsf15 | 5 | 77139878 | 77156228 | - | protein_coding | NA |
| ENSRNOG00000005094 | FALSE | TRUE | C1qtnf7 | 14 | 67468014 | 67580410 | - | protein_coding | NA |
| ENSRNOG00000030689 | FALSE | TRUE | Ms4a6bl | 1 | 2.08E+08 | 2.08E+08 | + | protein_coding | NA |
| ENSRNOG00000064985 | FALSE | TRUE | ENSRNOG00000064985 | 1 | 1.53E+08 | 1.53E+08 | - | lncRNA | NA |

| | | | | | | | | | |
|---|---|---|---|---|---|---|---|---|---|
| ENSRNOG00000004812 | FALSE | TRUE | Sema6d | 3 | 1.12E+08 | 1.12E+08 | + | protein_coding | NA |
| ENSRNOG00000002592 | FALSE | TRUE | Rps6ka6 | X | 76353760 | 76454484 | - | protein_coding | NA |
| ENSRNOG00000003845 | FALSE | TRUE | Wnt3 | 10 | 88680248 | 88724099 | + | protein_coding | NA |
| ENSRNOG00000020687 | FALSE | TRUE | Dpf1 | 1 | 84602336 | 84615950 | + | protein_coding | NA |
| ENSRNOG00000004141 | FALSE | TRUE | Vasn | 10 | 10917605 | 10928357 | - | protein_coding | NA |
| ENSRNOG00000017212 | FALSE | TRUE | Spsb1 | 5 | 1.6E+08 | 1.6E+08 | - | protein_coding | NA |
| ENSRNOG00000020239 | FALSE | TRUE | Capn15 | 10 | 14972800 | 14999508 | - | protein_coding | Others |
| ENSRNOG00000021856 | FALSE | TRUE | Lat2 | 12 | 22104219 | 22118288 | + | protein_coding | NA |
| ENSRNOG00000059984 | FALSE | TRUE | Dll1 | 1 | 56312066 | 56320179 | - | protein_coding | NA |
| ENSRNOG00000026226 | FALSE | TRUE | Hook1 | 5 | 1.11E+08 | 1.11E+08 | + | protein_coding | NA |
| ENSRNOG00000014264 | FALSE | TRUE | Phactr1 | 17 | 21562721 | 22039831 | - | protein_coding | NA |
| ENSRNOG00000017206 | FALSE | TRUE | Igfbp5 | 9 | 74448382 | 74465156 | - | protein_coding | NA |
| ENSRNOG00000014964 | FALSE | TRUE | Hp | 19 | 37539627 | 37544523 | - | protein_coding | NA |
| ENSRNOG00000026128 | FALSE | TRUE | Cpne8 | 7 | 1.22E+08 | 1.22E+08 | - | protein_coding | NA |
| ENSRNOG00000024046 | FALSE | TRUE | Sprr3 | 2 | 1.78E+08 | 1.78E+08 | - | protein_coding | NA |
| ENSRNOG00000032002 | FALSE | TRUE | Hapln1 | 2 | 20631640 | 20693777 | + | protein_coding | NA |
| ENSRNOG00000059050 | FALSE | TRUE | AABR07001416.1 | 7 | 1.33E+08 | 1.33E+08 | + | protein_coding | NA |
| ENSRNOG00000019174 | FALSE | TRUE | Chtf18 | 10 | 14742621 | 14751050 | - | protein_coding | NA |
| ENSRNOG00000008030 | FALSE | TRUE | Shc2 | 7 | 10066705 | 10088926 | + | protein_coding | NA |
| ENSRNOG00000014678 | FALSE | TRUE | Fzd5 | 9 | 66113112 | 66121457 | - | protein_coding | NA |
| ENSRNOG00000053026 | FALSE | TRUE | Shcbp1 | 16 | 84672321 | 84703844 | + | protein_coding | NA |
| ENSRNOG00000039725 | FALSE | TRUE | Ces1a | 19 | 13959482 | 13986370 | + | protein_coding | NA |
| ENSRNOG00000000456 | FALSE | TRUE | Psmb8 | 20 | 4652159 | 4655283 | - | protein_coding | NA |
| ENSRNOG00000032827 | FALSE | TRUE | AABR07058412.1 | 7 | 1.06E+08 | 1.06E+08 | - | protein_coding | NA |
| ENSRNOG00000063393 | FALSE | TRUE | ENSRNOG00000063393 | 6 | 10495268 | 10572247 | + | lncRNA | NA |
| ENSRNOG00000000017 | FALSE | TRUE | Steap1 | 4 | 28276909 | 28287479 | + | protein_coding | NA |
| ENSRNOG00000017981 | FALSE | TRUE | Mcm10 | 17 | 73266095 | 73287364 | + | protein_coding | NA |
| ENSRNOG00000022964 | FALSE | TRUE | Rnf222 | 10 | 53596751 | 53603300 | + | protein_coding | NA |
| ENSRNOG00000065902 | FALSE | TRUE | ENSRNOG00000065902 | 2 | 8106020 | 8283660 | + | lncRNA | NA |
| ENSRNOG00000033564 | FALSE | TRUE | Cfd | 7 | 9813150 | 9815053 | - | protein_coding | NA |
| ENSRNOG00000009522 | FALSE | TRUE | Adra1a | 15 | 40832534 | 40927500 | + | protein_coding | NA |
| ENSRNOG00000043219 | FALSE | TRUE | Fbn2 | 18 | 51499737 | 51703976 | - | protein_coding | NA |
| ENSRNOG00000057173 | FALSE | TRUE | Lipn | 1 | 2.32E+08 | 2.32E+08 | + | protein_coding | NA |
| ENSRNOG00000020434 | FALSE | TRUE | Ascl2 | 1 | 1.98E+08 | 1.98E+08 | - | protein_coding | bHLH |

| | | | | | | | | | |
|---|---|---|---|---|---|---|---|---|---|
| ENSRNOG00000000512 | FALSE | TRUE | Slc26a8 | 20 | 6697723 | 6749478 | - | protein_coding | NA |
| ENSRNOG00000050761 | FALSE | TRUE | Serpina10 | 6 | 1.23E+08 | 1.23E+08 | - | protein_coding | NA |
| ENSRNOG00000030021 | FALSE | TRUE | ENSRNOG00000030021 | 10 | 68380188 | 68384001 | - | protein_coding | NA |
| ENSRNOG00000051216 | FALSE | TRUE | AABR07038983.1 | X | 61138374 | 61144585 | + | lncRNA | NA |
| ENSRNOG00000007573 | FALSE | TRUE | Hoxb9 | 10 | 81237312 | 81240818 | + | protein_coding | Homeobox |
| ENSRNOG00000002014 | FALSE | TRUE | Stap1 | 14 | 21952496 | 21981245 | - | protein_coding | NA |
| ENSRNOG00000063483 | FALSE | TRUE | ENSRNOG00000063483 | 11 | 28074851 | 28075375 | + | protein_coding | NA |
| ENSRNOG00000019776 | FALSE | TRUE | Sh3gl3 | 1 | 1.36E+08 | 1.36E+08 | + | protein_coding | NA |
| ENSRNOG00000028566 | FALSE | TRUE | Pld4 | 6 | 1.32E+08 | 1.32E+08 | + | protein_coding | NA |
| ENSRNOG00000014259 | FALSE | TRUE | Mycl | 5 | 1.35E+08 | 1.35E+08 | + | protein_coding | bHLH |
| ENSRNOG00000031851 | FALSE | TRUE | Ndufa4l2 | 7 | 63356883 | 63359010 | + | protein_coding | NA |
| ENSRNOG00000066609 | FALSE | TRUE | ENSRNOG00000066609 | 4 | 1.49E+08 | 1.5E+08 | + | protein_coding | NA |
| ENSRNOG00000019607 | FALSE | TRUE | Bglap | 2 | 1.74E+08 | 1.74E+08 | - | protein_coding | NA |
| ENSRNOG00000013024 | FALSE | TRUE | Csgalnact1 | 16 | 21235784 | 21330319 | + | protein_coding | NA |
| ENSRNOG00000013389 | FALSE | TRUE | Rgs7bp | 2 | 36139201 | 36231505 | - | protein_coding | NA |
| ENSRNOG00000006367 | FALSE | TRUE | Slc5a8 | 7 | 23272427 | 23314642 | + | protein_coding | NA |
| ENSRNOG00000010457 | FALSE | TRUE | Vash1 | 6 | 1.06E+08 | 1.06E+08 | + | protein_coding | NA |
| ENSRNOG00000028865 | FALSE | TRUE | Kprp | 2 | 1.78E+08 | 1.78E+08 | - | protein_coding | NA |
| ENSRNOG00000007164 | FALSE | TRUE | Cln6 | 8 | 63303029 | 63318360 | + | protein_coding | NA |
| ENSRNOG00000013967 | FALSE | TRUE | Blnk | 1 | 2.4E+08 | 2.4E+08 | - | protein_coding | NA |
| ENSRNOG00000001514 | FALSE | TRUE | Cdca7 | 3 | 57322708 | 57333353 | + | protein_coding | NA |
| ENSRNOG00000004667 | FALSE | TRUE | Gen1 | 6 | 33948206 | 33978401 | - | protein_coding | NA |
| ENSRNOG00000006198 | FALSE | TRUE | Prr11 | 10 | 71901202 | 71921012 | - | protein_coding | NA |
| ENSRNOG00000023511 | FALSE | TRUE | 2310050C09Rik | 2 | 1.78E+08 | 1.78E+08 | - | protein_coding | NA |
| ENSRNOG00000065696 | FALSE | TRUE | ENSRNOG00000065696 | 12 | 27678556 | 27687753 | + | lncRNA | NA |
| ENSRNOG00000039191 | FALSE | TRUE | Yjefn3 | 16 | 19533788 | 19539728 | + | protein_coding | NA |
| ENSRNOG00000063476 | FALSE | TRUE | ENSRNOG00000063476 | 10 | 69780210 | 69790446 | - | protein_coding | NA |
| ENSRNOG00000016539 | FALSE | TRUE | Rab24 | 17 | 9308525 | 9310553 | + | protein_coding | NA |
| ENSRNOG00000011808 | FALSE | TRUE | Ghrhr | 4 | 84500212 | 84532776 | + | protein_coding | NA |
| ENSRNOG00000050059 | FALSE | TRUE | ENSRNOG00000050059 | 12 | 18869048 | 18880075 | + | protein_coding | NA |
| ENSRNOG00000058939 | FALSE | TRUE | Spem2 | 10 | 54537174 | 54539058 | - | protein_coding | NA |
| ENSRNOG00000029886 | FALSE | TRUE | Hba-a2 | 10 | 15307815 | 15338392 | - | protein_coding | NA |
| ENSRNOG00000024237 | FALSE | TRUE | Scel | 15 | 80339951 | 80456372 | + | protein_coding | NA |
| ENSRNOG00000009712 | FALSE | TRUE | Gale | 5 | 1.48E+08 | 1.48E+08 | + | protein_coding | NA |

| | | | | | | | | | |
|---|---|---|---|---|---|---|---|---|---|
| ENSRNOG00000011439 | FALSE | TRUE | Grk5 | 1 | 2.6E+08 | 2.6E+08 | + | protein_coding | NA |
| ENSRNOG00000020275 | FALSE | TRUE | Ccr10 | 10 | 86108551 | 86110988 | - | protein_coding | NA |
| ENSRNOG00000054420 | FALSE | TRUE | Vdr | 7 | 1.29E+08 | 1.29E+08 | - | protein_coding | THR-like |
| ENSRNOG00000046566 | FALSE | TRUE | Tub | 1 | 1.63E+08 | 1.63E+08 | + | protein_coding | Tub |
| ENSRNOG00000025297 | FALSE | TRUE | Gpr171 | 2 | 1.43E+08 | 1.43E+08 | - | protein_coding | NA |
| ENSRNOG00000046309 | FALSE | TRUE | Gpr68 | 6 | 1.2E+08 | 1.2E+08 | - | protein_coding | NA |
| ENSRNOG00000001627 | FALSE | TRUE | Abi3bp | 11 | 43913043 | 44128929 | - | protein_coding | NA |
| ENSRNOG00000069221 | FALSE | TRUE | Kctd16 | 18 | 32166861 | 32491749 | + | protein_coding | NA |
| ENSRNOG00000014751 | FALSE | TRUE | Ret | 4 | 1.51E+08 | 1.51E+08 | - | protein_coding | NA |
| ENSRNOG00000015895 | FALSE | TRUE | B4galt6 | 18 | 11958390 | 12015247 | - | protein_coding | NA |
| ENSRNOG00000023093 | FALSE | TRUE | Mis18bp1 | 6 | 83182499 | 83231383 | - | protein_coding | MYB |
| ENSRNOG00000064404 | FALSE | TRUE | ENSRNOG00000064404 | 7 | 89548622 | 89553136 | + | lncRNA | NA |
| ENSRNOG00000049596 | FALSE | TRUE | Cfap58 | 1 | 2.47E+08 | 2.47E+08 | + | protein_coding | NA |
| ENSRNOG00000068784 | FALSE | TRUE | ENSRNOG00000068784 | 7 | 1.33E+08 | 1.33E+08 | - | protein_coding | NA |
| ENSRNOG00000008115 | FALSE | TRUE | Arhgap11a | 3 | 1.01E+08 | 1.01E+08 | - | protein_coding | NA |
| ENSRNOG00000033496 | FALSE | TRUE | Igdcc4 | 8 | 65621897 | 65657648 | + | protein_coding | NA |
| ENSRNOG00000033706 | FALSE | TRUE | Klk14 | 1 | 94131998 | 94137240 | + | protein_coding | NA |
| ENSRNOG00000063568 | FALSE | TRUE | ENSRNOG00000063568 | 3 | 29390015 | 29400630 | + | lncRNA | NA |
| ENSRNOG00000006639 | FALSE | TRUE | Scn9a | 3 | 51145146 | 51293516 | - | protein_coding | NA |
| ENSRNOG00000048980 | FALSE | TRUE | Gng2 | 15 | 4316059 | 4417183 | + | lncRNA | NA |
| ENSRNOG00000064788 | FALSE | TRUE | ENSRNOG00000064788 | 18 | 28144342 | 28162144 | - | lncRNA | NA |
| ENSRNOG00000051837 | FALSE | TRUE | Nwd2 | 14 | 44482287 | 44670299 | - | protein_coding | NA |
| ENSRNOG00000022921 | FALSE | TRUE | Dact2 | 1 | 55181024 | 55191096 | - | protein_coding | NA |
| ENSRNOG00000053400 | FALSE | TRUE | Cfi | 2 | 2.18E+08 | 2.18E+08 | + | protein_coding | NA |
| ENSRNOG00000039861 | FALSE | TRUE | ENSRNOG00000039861 | 7 | 8345303 | 8347429 | + | protein_coding | NA |
| ENSRNOG00000031643 | FALSE | TRUE | Dchs1 | 1 | 1.6E+08 | 1.6E+08 | - | protein_coding | NA |
| ENSRNOG00000061595 | FALSE | TRUE | Tfec | 4 | 45107641 | 45180236 | - | protein_coding | bHLH |
| ENSRNOG00000016368 | FALSE | TRUE | Ppp1r14c | 1 | 39773403 | 39886956 | + | protein_coding | NA |
| ENSRNOG00000048275 | FALSE | TRUE | RGD1309350 | 1 | 1.96E+08 | 1.96E+08 | + | protein_coding | NA |
| ENSRNOG00000067849 | FALSE | TRUE | ENSRNOG00000067849 | 20 | 20525256 | 20609951 | - | protein_coding | NA |
| ENSRNOG00000010617 | FALSE | TRUE | Scube1 | 7 | 1.15E+08 | 1.15E+08 | - | protein_coding | NA |
| ENSRNOG00000001115 | FALSE | TRUE | Slc29a4 | 12 | 11853540 | 11874834 | - | protein_coding | NA |
| ENSRNOG00000001324 | FALSE | TRUE | Asmt | 12 | 16304719 | 16309568 | - | protein_coding | NA |
| ENSRNOG00000007271 | FALSE | TRUE | Map3k9 | 6 | 1.01E+08 | 1.01E+08 | - | protein_coding | NA |

| | | | | | | | | | |
|---|---|---|---|---|---|---|---|---|---|
| ENSRNOG00000055433 | FALSE | TRUE | Trim46 | 2 | 1.75E+08 | 1.75E+08 | - | protein_coding | NA |
| ENSRNOG00000029810 | FALSE | TRUE | Tspan4 | 1 | 1.97E+08 | 1.97E+08 | + | protein_coding | NA |
| ENSRNOG00000064145 | FALSE | TRUE | ENSRNOG00000064145 | 1 | 1.24E+08 | 1.24E+08 | + | lncRNA | NA |
| ENSRNOG00000013663 | FALSE | TRUE | Tmem86a | 1 | 97595842 | 97600260 | + | protein_coding | NA |
| ENSRNOG00000050968 | FALSE | TRUE | Ptger2 | 15 | 18217285 | 18228714 | + | protein_coding | NA |
| ENSRNOG00000008062 | FALSE | TRUE | Dqx1 | 4 | 1.16E+08 | 1.16E+08 | + | protein_coding | NA |
| ENSRNOG00000002469 | FALSE | TRUE | Trim7 | 10 | 33205491 | 33221468 | + | protein_coding | NA |
| ENSRNOG00000049497 | FALSE | TRUE | Eif6 | 3 | 1.44E+08 | 1.44E+08 | - | protein_coding | NA |
| ENSRNOG00000070872 | FALSE | TRUE | Lce1g | 2 | 1.78E+08 | 1.78E+08 | - | protein_coding | NA |
| ENSRNOG00000006119 | FALSE | TRUE | Slc7a15 | 6 | 31403747 | 31415231 | - | protein_coding | NA |
| ENSRNOG00000024543 | FALSE | TRUE | Mfsd4b | 20 | 43324436 | 43333953 | - | protein_coding | NA |
| ENSRNOG00000032659 | FALSE | TRUE | Plcl1 | 9 | 56901534 | 57238782 | + | protein_coding | NA |
| ENSRNOG00000001288 | FALSE | TRUE | Gpr146 | 12 | 15251813 | 15267206 | - | protein_coding | NA |
| ENSRNOG00000052376 | FALSE | TRUE | Bend6 | 9 | 36081484 | 36135228 | - | protein_coding | NA |
| ENSRNOG00000012720 | FALSE | TRUE | Irx4 | 1 | 30030561 | 30039549 | - | protein_coding | Homeobox |
| ENSRNOG00000002305 | FALSE | TRUE | Slc15a2 | 11 | 64014182 | 64043225 | + | protein_coding | NA |
| ENSRNOG00000050071 | FALSE | TRUE | Cdc45 | 11 | 82211475 | 82214992 | - | protein_coding | NA |
| ENSRNOG00000013445 | FALSE | TRUE | Aaas | 7 | 1.33E+08 | 1.33E+08 | - | protein_coding | NA |
| ENSRNOG00000018938 | FALSE | TRUE | Mkx | 17 | 55077540 | 55156124 | - | protein_coding | Homeobox |
| ENSRNOG00000042182 | FALSE | TRUE | Mroh2a | 9 | 88814197 | 88852607 | + | protein_coding | NA |
| ENSRNOG00000068922 | FALSE | TRUE | Oas1f | 12 | 35697015 | 35706323 | - | protein_coding | NA |
| ENSRNOG00000030676 | FALSE | TRUE | AABR07044373.1 | 20 | 3172330 | 3173886 | - | protein_coding | NA |
| ENSRNOG00000012447 | FALSE | TRUE | Setd6 | 19 | 9347458 | 9350453 | - | protein_coding | NA |
| ENSRNOG00000004476 | FALSE | TRUE | Wif1 | 7 | 56548053 | 56618360 | + | protein_coding | NA |
| ENSRNOG00000019383 | FALSE | TRUE | Tef | 7 | 1.13E+08 | 1.13E+08 | + | protein_coding | TF_bZIP |
| ENSRNOG00000013318 | FALSE | TRUE | Ccdc150 | 9 | 55793551 | 55897224 | + | protein_coding | NA |
| ENSRNOG00000006324 | FALSE | TRUE | ENSRNOG00000006324 | 8 | 5758935 | 5828092 | + | protein_coding | NA |
| ENSRNOG00000063398 | FALSE | TRUE | Nat8 | 4 | 1.18E+08 | 1.18E+08 | - | protein_coding | NA |
| ENSRNOG00000057315 | FALSE | TRUE | Kcnh3 | 7 | 1.3E+08 | 1.3E+08 | + | protein_coding | NA |
| ENSRNOG00000016413 | FALSE | TRUE | Pstpip1 | 8 | 56499590 | 56538580 | + | protein_coding | NA |
| ENSRNOG00000015599 | FALSE | TRUE | Mall | 3 | 1.15E+08 | 1.15E+08 | - | protein_coding | NA |
| ENSRNOG00000019542 | FALSE | TRUE | MGC108823 | 18 | 53748631 | 53772834 | + | protein_coding | NA |
| ENSRNOG00000006932 | FALSE | TRUE | AABR07029272.1 | 10 | 18827239 | 19244808 | - | protein_coding | NA |
| ENSRNOG00000007410 | FALSE | TRUE | Dab1 | 5 | 1.19E+08 | 1.2E+08 | + | protein_coding | NA |

| | | | | | | | | | |
|---|---|---|---|---|---|---|---|---|---|
| ENSRNOG00000053240 | FALSE | TRUE | Soga1 | 3 | 1.46E+08 | 1.46E+08 | - | protein_coding | NA |
| ENSRNOG00000047321 | FALSE | TRUE | Hba-a3 | 10 | 15311634 | 15312481 | - | protein_coding | NA |
| ENSRNOG00000013213 | FALSE | TRUE | Epha4 | 9 | 78815460 | 78958139 | - | protein_coding | NA |
| ENSRNOG00000021401 | FALSE | TRUE | Lpar5 | 4 | 1.58E+08 | 1.58E+08 | + | protein_coding | NA |
| ENSRNOG00000055858 | FALSE | TRUE | Myb | 1 | 15939761 | 15973057 | - | protein_coding | MYB |
| ENSRNOG00000005413 | FALSE | TRUE | Creb3l1 | 3 | 77952540 | 77993456 | - | protein_coding | TF_bZIP |
| ENSRNOG00000031058 | FALSE | TRUE | Was | X | 14405124 | 14413849 | + | protein_coding | NA |
| ENSRNOG00000066247 | FALSE | TRUE | Sugct | 17 | 47376521 | 48234376 | + | protein_coding | NA |
| ENSRNOG00000015658 | FALSE | TRUE | Sorbs1 | 1 | 2.39E+08 | 2.39E+08 | - | protein_coding | NA |
| ENSRNOG00000039476 | FALSE | TRUE | Pcdhb2 | 18 | 29005996 | 29008782 | + | protein_coding | NA |
| ENSRNOG00000008409 | FALSE | TRUE | Myo1f | 7 | 14363350 | 14413911 | + | protein_coding | NA |
| ENSRNOG00000028733 | FALSE | TRUE | Prkar1b | 12 | 15511801 | 15624942 | + | protein_coding | NA |
| ENSRNOG00000033618 | FALSE | TRUE | Col28a1 | 4 | 36069970 | 36223220 | - | protein_coding | NA |
| ENSRNOG00000049615 | FALSE | TRUE | Fam228b | 6 | 27754506 | 27786537 | - | protein_coding | NA |
| ENSRNOG00000012655 | FALSE | TRUE | Adamts6 | 2 | 35421510 | 35633298 | + | protein_coding | NA |
| ENSRNOG00000042607 | FALSE | TRUE | Rhof | 12 | 33431280 | 33446703 | + | protein_coding | NA |
| ENSRNOG00000033335 | FALSE | TRUE | Cenpi | X | 97515972 | 97567657 | + | protein_coding | NA |
| ENSRNOG00000070391 | FALSE | TRUE | ENSRNOG00000070391 | 5 | 57985355 | 57987537 | + | protein_coding | NA |
| ENSRNOG00000005575 | FALSE | TRUE | Abi3 | 10 | 80769822 | 80780816 | - | protein_coding | NA |
| ENSRNOG00000030771 | FALSE | TRUE | Dgkb | 6 | 54641614 | 55397043 | + | protein_coding | NA |
| ENSRNOG00000066309 | FALSE | TRUE | ENSRNOG00000066309 | X | 1239931 | 1292355 | - | lncRNA | NA |
| ENSRNOG00000042105 | FALSE | TRUE | Lce1l | 2 | 1.78E+08 | 1.78E+08 | - | protein_coding | NA |
| ENSRNOG00000064495 | FALSE | TRUE | ENSRNOG00000064495 | 19 | 52912680 | 52915635 | + | lncRNA | NA |
| ENSRNOG00000056258 | FALSE | TRUE | Dsc1 | 18 | 11502003 | 11528494 | - | protein_coding | NA |
| ENSRNOG00000009636 | FALSE | TRUE | Scrn1 | 4 | 83632131 | 83693852 | - | protein_coding | NA |
| ENSRNOG00000006589 | FALSE | TRUE | Mif | 20 | 12790902 | 12799504 | + | protein_coding | NA |
| ENSRNOG00000052803 | FALSE | TRUE | Klri1 | 4 | 1.63E+08 | 1.63E+08 | - | protein_coding | NA |
| ENSRNOG00000042592 | FALSE | TRUE | Rgs10 | 1 | 1.83E+08 | 1.83E+08 | - | protein_coding | NA |
| ENSRNOG00000051691 | FALSE | TRUE | Gjd3 | 10 | 83934135 | 83934971 | - | protein_coding | NA |
| ENSRNOG00000067363 | FALSE | TRUE | Xkrx | X | 97341152 | 97354759 | - | protein_coding | NA |
| ENSRNOG00000016244 | FALSE | TRUE | Mical2 | 1 | 1.66E+08 | 1.66E+08 | + | protein_coding | NA |
| ENSRNOG00000038047 | FALSE | TRUE | Mt1 | 17 | 74786654 | 74787135 | + | protein_coding | NA |
| ENSRNOG00000070046 | FALSE | TRUE | Cdc45 | 11 | 82185473 | 82210619 | + | protein_coding | NA |
| ENSRNOG00000046452 | FALSE | TRUE | Fcgr2b | 13 | 83193163 | 83207778 | - | protein_coding | NA |

| | | | | | | | | | |
|---|---|---|---|---|---|---|---|---|---|
| ENSRNOG00000006278 | FALSE | TRUE | Tspyl5 | 7 | 64884225 | 64885457 | - | protein_coding | NA |
| ENSRNOG00000032134 | FALSE | TRUE | Uqcrc1 | 8 | 1.1E+08 | 1.1E+08 | + | protein_coding | NA |
| ENSRNOG00000065458 | FALSE | TRUE | ENSRNOG00000065458 | 8 | 84075040 | 84104402 | + | lncRNA | NA |
| ENSRNOG00000000201 | FALSE | TRUE | Gsta5 | 8 | 79105661 | 79117472 | + | protein_coding | NA |
| ENSRNOG00000027295 | FALSE | TRUE | Aph1b | 8 | 67429198 | 67450243 | - | protein_coding | NA |
| ENSRNOG00000058506 | FALSE | TRUE | AC095078.1 | 4 | 1.16E+08 | 1.16E+08 | + | lncRNA | NA |
| ENSRNOG00000068660 | FALSE | TRUE | ENSRNOG00000068660 | 7 | 61043675 | 61044761 | - | lncRNA | NA |
| ENSRNOG00000017606 | FALSE | TRUE | P2rx1 | 10 | 57618586 | 57633623 | + | protein_coding | NA |
| ENSRNOG00000007585 | FALSE | TRUE | Hoxb8 | 10 | 81248887 | 81250638 | + | protein_coding | Homeobox |
| ENSRNOG00000019842 | FALSE | TRUE | Artn | 5 | 1.31E+08 | 1.31E+08 | - | protein_coding | NA |
| ENSRNOG00000054902 | FALSE | TRUE | Trpc1 | 8 | 96263329 | 96314276 | - | protein_coding | NA |
| ENSRNOG00000024264 | FALSE | TRUE | Amz1 | 12 | 13891123 | 13909783 | - | protein_coding | NA |
| ENSRNOG00000046643 | FALSE | TRUE | Cyp3a9 | 12 | 16806207 | 16846422 | - | protein_coding | NA |
| ENSRNOG00000008282 | FALSE | TRUE | Elf5 | 3 | 89797880 | 89836973 | + | protein_coding | ETS |
| ENSRNOG00000000918 | FALSE | TRUE | Zbed5 | 12 | 26807600 | 26813664 | - | protein_coding | NA |
| ENSRNOG00000015906 | FALSE | TRUE | Tgif1 | 9 | 1.11E+08 | 1.11E+08 | - | protein_coding | Homeobox |
| ENSRNOG00000024008 | FALSE | TRUE | Cdc25c | 18 | 26335834 | 26356185 | - | protein_coding | NA |
| ENSRNOG00000009390 | FALSE | TRUE | Edn2 | 5 | 1.34E+08 | 1.34E+08 | + | protein_coding | NA |
| ENSRNOG00000014937 | FALSE | TRUE | Lrrc71 | 2 | 1.73E+08 | 1.73E+08 | - | protein_coding | NA |
| ENSRNOG00000063579 | FALSE | TRUE | ENSRNOG00000063579 | 13 | 43631977 | 43650173 | + | lncRNA | NA |
| ENSRNOG00000018566 | FALSE | TRUE | Ctsl | 17 | 764309 | 770548 | + | protein_coding | NA |
| ENSRNOG00000006450 | FALSE | TRUE | Erbb2 | 10 | 83411313 | 83435078 | + | protein_coding | NA |
| ENSRNOG00000004766 | FALSE | TRUE | Glis2 | 10 | 10951371 | 10971578 | - | protein_coding | zf-C2H2 |
| ENSRNOG00000049829 | FALSE | TRUE | AABR07060872.1 | 4 | 1.02E+08 | 1.03E+08 | + | protein_coding | NA |
| ENSRNOG00000022426 | FALSE | TRUE | C130050O18Rik | 12 | 15229743 | 15242139 | - | protein_coding | NA |
| ENSRNOG00000023657 | FALSE | TRUE | Gprin3 | 4 | 88563607 | 88657429 | - | protein_coding | NA |
| ENSRNOG00000060100 | FALSE | TRUE | Knl1 | 3 | 1.06E+08 | 1.06E+08 | + | protein_coding | NA |
| ENSRNOG00000063070 | FALSE | TRUE | Pcdhga7 | 18 | 29493954 | 29667868 | + | protein_coding | NA |
| ENSRNOG00000016312 | FALSE | TRUE | Catsper4 | 5 | 1.46E+08 | 1.46E+08 | - | protein_coding | NA |
| ENSRNOG00000011076 | FALSE | TRUE | Ank2 | 2 | 2.15E+08 | 2.16E+08 | - | protein_coding | NA |
| ENSRNOG00000012358 | FALSE | TRUE | Pcyt1b | X | 58378116 | 58468935 | + | protein_coding | NA |
| ENSRNOG00000058780 | FALSE | TRUE | Igfbp1 | 14 | 82047415 | 82052482 | + | protein_coding | NA |
| ENSRNOG00000042747 | FALSE | TRUE | Fam72a | 13 | 42967578 | 42984208 | + | protein_coding | NA |
| ENSRNOG00000049151 | FALSE | TRUE | Csap1 | 10 | 12914455 | 12916334 | + | protein_coding | NA |

| | | | | | | | | | |
|---|---|---|---|---|---|---|---|---|---|
| ENSRNOG00000022030 | FALSE | TRUE | Aunip | 5 | 1.47E+08 | 1.47E+08 | + | protein_coding | NA |
| ENSRNOG00000059997 | FALSE | TRUE | Brip1 | 10 | 70907371 | 71030324 | - | protein_coding | NA |
| ENSRNOG00000034228 | FALSE | TRUE | Pik3c2g | 4 | 1.72E+08 | 1.73E+08 | + | protein_coding | NA |
| ENSRNOG00000047867 | FALSE | TRUE | Klhdc8b | 8 | 1.09E+08 | 1.09E+08 | - | protein_coding | NA |
| ENSRNOG00000005037 | FALSE | TRUE | Kif18a | 3 | 95764388 | 95824582 | + | protein_coding | NA |
| ENSRNOG00000033123 | FALSE | TRUE | Pcdhb20 | 18 | 29211883 | 29215258 | + | protein_coding | NA |
| ENSRNOG00000004554 | FALSE | TRUE | Dcn | 7 | 32281252 | 32321270 | + | protein_coding | NA |
| ENSRNOG00000003476 | FALSE | TRUE | Slc6a4 | 10 | 61826123 | 61858384 | + | protein_coding | NA |
| ENSRNOG00000009660 | FALSE | TRUE | Enpp6 | 16 | 45169178 | 45294077 | - | protein_coding | NA |
| ENSRNOG00000037449 | FALSE | TRUE | Pole | 12 | 46345420 | 46393939 | - | protein_coding | NA |
| ENSRNOG00000033064 | FALSE | TRUE | Edil3 | 2 | 19890373 | 20405008 | + | protein_coding | NA |
| ENSRNOG00000045678 | FALSE | TRUE | Krtap3-3 | 10 | 84511092 | 84511812 | - | protein_coding | NA |
| ENSRNOG00000014376 | FALSE | TRUE | Proc | 18 | 23764368 | 23775133 | - | protein_coding | NA |
| ENSRNOG00000058983 | FALSE | TRUE | Gata5 | 3 | 1.67E+08 | 1.67E+08 | - | protein_coding | zf-GATA |
| ENSRNOG00000018251 | FALSE | TRUE | Mrc1 | 17 | 77249187 | 77330857 | + | protein_coding | NA |
| ENSRNOG00000013823 | FALSE | TRUE | Cers3 | 1 | 1.2E+08 | 1.2E+08 | + | protein_coding | NA |
| ENSRNOG00000066613 | FALSE | TRUE | RGD1564571 | 12 | 2360071 | 2365560 | + | protein_coding | NA |
| ENSRNOG00000012932 | FALSE | TRUE | Ccdc146 | 4 | 13662766 | 13811608 | + | protein_coding | NA |
| ENSRNOG00000039859 | FALSE | TRUE | Dnmt1 | 8 | 19440611 | 19486659 | - | protein_coding | NA |
| ENSRNOG00000008436 | FALSE | TRUE | Krt85 | 7 | 1.33E+08 | 1.33E+08 | - | protein_coding | NA |
| ENSRNOG00000014576 | FALSE | TRUE | Dock3 | 8 | 1.08E+08 | 1.08E+08 | - | protein_coding | NA |
| ENSRNOG00000064054 | FALSE | TRUE | ENSRNOG00000064054 | 7 | 1.09E+08 | 1.09E+08 | - | lncRNA | NA |
| ENSRNOG00000039251 | FALSE | TRUE | Spink9 | 18 | 36426989 | 36430271 | + | protein_coding | NA |
| ENSRNOG00000043332 | FALSE | TRUE | Foxd1 | 2 | 29702558 | 29704978 | + | protein_coding | Fork_head |
| ENSRNOG00000066240 | FALSE | TRUE | ENSRNOG00000066240 | 15 | 24454978 | 24463251 | - | lncRNA | NA |
| ENSRNOG00000047233 | FALSE | TRUE | Lyg1 | 9 | 40171123 | 40182869 | - | protein_coding | NA |
| ENSRNOG00000020533 | FALSE | TRUE | Htra1 | 1 | 1.85E+08 | 1.86E+08 | + | protein_coding | NA |
| ENSRNOG00000027139 | FALSE | TRUE | Krt20 | 10 | 84384802 | 84394107 | - | protein_coding | NA |
| ENSRNOG00000016678 | FALSE | TRUE | Angptl2 | 3 | 16517420 | 16548178 | + | protein_coding | NA |
| ENSRNOG00000014336 | FALSE | TRUE | Mcm5 | 19 | 13483066 | 13504389 | + | protein_coding | NA |
| ENSRNOG00000067704 | FALSE | TRUE | ENSRNOG00000067704 | 5 | 1.6E+08 | 1.6E+08 | - | lncRNA | NA |
| ENSRNOG00000016752 | FALSE | TRUE | Crispld2 | 19 | 48053287 | 48110465 | + | protein_coding | NA |
| ENSRNOG00000013128 | FALSE | TRUE | Tmem179 | 6 | 1.32E+08 | 1.32E+08 | - | protein_coding | NA |
| ENSRNOG00000029508 | FALSE | TRUE | Slpil2 | 3 | 1.53E+08 | 1.53E+08 | - | protein_coding | NA |

| | | | | | | | | | |
|---|---|---|---|---|---|---|---|---|---|
| ENSRNOG00000019183 | FALSE | TRUE | Alox15 | 10 | 55060412 | 55068874 | - | protein_coding | NA |
| ENSRNOG00000017198 | FALSE | TRUE | Hif3a | 1 | 77722471 | 77749758 | - | protein_coding | NA |
| ENSRNOG00000013820 | FALSE | TRUE | Tnfrsf14 | 5 | 1.65E+08 | 1.65E+08 | - | protein_coding | NA |
| ENSRNOG00000010386 | FALSE | TRUE | H2ax | 8 | 44671786 | 44673239 | + | protein_coding | NA |
| ENSRNOG00000031397 | FALSE | TRUE | Stk32b | 14 | 73078061 | 73336458 | + | protein_coding | NA |
| ENSRNOG00000051302 | FALSE | TRUE | Krt34 | 10 | 84970017 | 84974040 | - | protein_coding | NA |
| ENSRNOG00000020028 | FALSE | TRUE | Pcdhb22 | 18 | 29223149 | 29225536 | + | protein_coding | NA |
| ENSRNOG00000013898 | FALSE | TRUE | Cdca4 | 6 | 1.32E+08 | 1.32E+08 | - | protein_coding | NA |
| ENSRNOG00000049179 | FALSE | TRUE | Pidd1 | 1 | 1.97E+08 | 1.97E+08 | - | protein_coding | NA |
| ENSRNOG00000001295 | FALSE | TRUE | S100b | 20 | 12372881 | 12394743 | - | protein_coding | NA |
| ENSRNOG00000016526 | FALSE | TRUE | Dsg2 | 18 | 11846183 | 11904156 | + | protein_coding | NA |
| ENSRNOG00000056209 | FALSE | TRUE | Exo1 | 13 | 87809810 | 87834654 | + | protein_coding | NA |
| ENSRNOG00000065419 | FALSE | TRUE | ENSRNOG00000065419 | 11 | 28068788 | 28069300 | - | protein_coding | NA |
| ENSRNOG00000039260 | FALSE | TRUE | RGD1563060 | 18 | 35948402 | 35969502 | - | protein_coding | NA |
| ENSRNOG00000042133 | FALSE | TRUE | AABR07006275.1 | 1 | 2.08E+08 | 2.08E+08 | + | protein_coding | NA |
| ENSRNOG00000004242 | FALSE | TRUE | Pole2 | 6 | 87674702 | 87699305 | - | protein_coding | NA |
| ENSRNOG00000067018 | FALSE | TRUE | ENSRNOG00000067018 | 20 | 3864200 | 3870816 | - | protein_coding | NA |
| ENSRNOG00000000825 | FALSE | TRUE | Calhm5 | 20 | 26066242 | 26072272 | - | protein_coding | NA |
| ENSRNOG00000070355 | FALSE | TRUE | Tedc1 | 6 | 1.32E+08 | 1.32E+08 | + | protein_coding | NA |
| ENSRNOG00000020277 | FALSE | TRUE | Cntnap1 | 10 | 86111643 | 86125611 | + | protein_coding | NA |
| ENSRNOG00000063822 | FALSE | TRUE | ENSRNOG00000063822 | 11 | 15764222 | 15837935 | - | lncRNA | NA |
| ENSRNOG00000005180 | FALSE | TRUE | Vstm2a | 14 | 90660004 | 90685656 | + | protein_coding | NA |
| ENSRNOG00000026309 | FALSE | TRUE | Lrrc23 | 4 | 1.58E+08 | 1.58E+08 | - | protein_coding | NA |
| ENSRNOG00000069818 | FALSE | TRUE | ENSRNOG00000069818 | 7 | 8327569 | 8331685 | - | lncRNA | NA |
| ENSRNOG00000070938 | FALSE | TRUE | ENSRNOG00000070938 | 18 | 27154164 | 27159936 | + | lncRNA | NA |
| ENSRNOG00000054438 | FALSE | TRUE | AU018091 | 1 | 66053268 | 66067039 | + | protein_coding | NA |
| ENSRNOG00000009577 | FALSE | TRUE | Ndst4 | 2 | 2.14E+08 | 2.14E+08 | + | protein_coding | NA |
| ENSRNOG00000009528 | FALSE | TRUE | Sdcbp2 | 3 | 1.4E+08 | 1.4E+08 | + | protein_coding | NA |
| ENSRNOG00000024093 | FALSE | TRUE | Dmrta1 | 5 | 1.04E+08 | 1.04E+08 | + | protein_coding | DM |
| ENSRNOG00000061897 | FALSE | TRUE | AABR07033579.3 | 11 | 28294843 | 28295058 | + | protein_coding | NA |
| ENSRNOG00000071113 | FALSE | TRUE | ENSRNOG00000071113 | 7 | 89437557 | 89446328 | - | protein_coding | NA |
| ENSRNOG00000055583 | FALSE | TRUE | Ttyh3 | 12 | 13997045 | 14025459 | + | protein_coding | NA |
| ENSRNOG00000019306 | FALSE | TRUE | Syt12 | 1 | 2.02E+08 | 2.02E+08 | - | protein_coding | NA |
| ENSRNOG00000034087 | FALSE | TRUE | Krt42 | 10 | 85185181 | 85192595 | - | protein_coding | NA |

| | | | | | | | | | |
|---|---|---|---|---|---|---|---|---|---|
| ENSRNOG00000024825 | FALSE | TRUE | Fam163a | 13 | 68262872 | 68341049 | - | protein_coding | NA |
| ENSRNOG00000003947 | FALSE | TRUE | Ntn1 | 10 | 52899934 | 53085326 | - | protein_coding | NA |
| ENSRNOG00000015366 | FALSE | TRUE | Neurl3 | 9 | 38494489 | 38502649 | - | protein_coding | NA |
| ENSRNOG00000024065 | FALSE | TRUE | Zfp575 | 1 | 80175016 | 80177123 | - | protein_coding | zf-C2H2 |
| ENSRNOG00000015397 | FALSE | TRUE | Cpne7 | 19 | 51166034 | 51182677 | + | protein_coding | NA |
| ENSRNOG00000039207 | FALSE | TRUE | Ccdc71l | 6 | 48960191 | 48960841 | + | protein_coding | NA |
| ENSRNOG00000026336 | FALSE | TRUE | Tmem255b | 16 | 76079845 | 76106795 | - | protein_coding | NA |
| ENSRNOG00000033835 | FALSE | TRUE | Dnm1 | 3 | 15604784 | 15648538 | - | protein_coding | NA |
| ENSRNOG00000009329 | FALSE | TRUE | Nr1d1 | 10 | 83728318 | 83735705 | - | protein_coding | THR-like |
| ENSRNOG00000024082 | FALSE | TRUE | Gldn | 8 | 54679119 | 54723196 | + | protein_coding | NA |
| ENSRNOG00000059479 | FALSE | TRUE | Adcy1 | 14 | 81911099 | 82028969 | + | protein_coding | NA |
| ENSRNOG00000008367 | FALSE | TRUE | Krt86 | 7 | 1.33E+08 | 1.33E+08 | + | protein_coding | NA |
| ENSRNOG00000002653 | FALSE | TRUE | Kcnk2 | 13 | 1.01E+08 | 1.01E+08 | - | protein_coding | NA |
| ENSRNOG00000037661 | FALSE | TRUE | ENSRNOG00000037661 | X | 1E+08 | 1E+08 | - | protein_coding | NA |
| ENSRNOG00000002599 | FALSE | TRUE | Grap | 10 | 46332909 | 46352056 | + | protein_coding | NA |
| ENSRNOG00000026310 | FALSE | TRUE | Marchf1 | 16 | 23258799 | 24114277 | - | protein_coding | NA |
| ENSRNOG00000017130 | FALSE | TRUE | Rhd | 5 | 1.47E+08 | 1.47E+08 | + | protein_coding | NA |
| ENSRNOG00000027722 | FALSE | TRUE | H1f10 | 4 | 1.2E+08 | 1.2E+08 | - | protein_coding | NA |
| ENSRNOG00000009005 | FALSE | TRUE | Slco2a1 | 8 | 1.04E+08 | 1.04E+08 | + | protein_coding | NA |
| ENSRNOG00000018830 | FALSE | TRUE | Aff3 | 9 | 40404375 | 40857247 | - | protein_coding | AF-4 |
| ENSRNOG00000009571 | FALSE | TRUE | Wipf3 | 4 | 83550468 | 83629386 | + | protein_coding | NA |
| ENSRNOG00000019004 | FALSE | TRUE | Mt4 | 19 | 10863813 | 10865909 | - | protein_coding | NA |
| ENSRNOG00000006921 | FALSE | TRUE | Rbl1 | 3 | 1.46E+08 | 1.46E+08 | - | protein_coding | NA |
| ENSRNOG00000025889 | FALSE | TRUE | Gnas | 3 | 1.63E+08 | 1.63E+08 | + | protein_coding | NA |
| ENSRNOG00000019243 | FALSE | TRUE | Glb1l | 9 | 76695173 | 76705510 | - | protein_coding | NA |
| ENSRNOG00000039300 | FALSE | TRUE | Ahcyl2 | 4 | 58381218 | 58531060 | + | protein_coding | NA |
| ENSRNOG00000003254 | FALSE | TRUE | Il23a | 7 | 721809 | 723923 | - | protein_coding | NA |
| ENSRNOG00000012280 | FALSE | TRUE | Ptx3 | 2 | 1.5E+08 | 1.5E+08 | + | protein_coding | NA |
| ENSRNOG00000004217 | FALSE | TRUE | Stk10 | 10 | 17117878 | 17212061 | + | protein_coding | NA |
| ENSRNOG00000030344 | FALSE | TRUE | Krt31 | 10 | 84979352 | 84982832 | - | protein_coding | NA |
| ENSRNOG00000037113 | FALSE | TRUE | Slfn2 | 10 | 67953171 | 67959458 | + | protein_coding | NA |
| ENSRNOG00000005751 | FALSE | TRUE | Il36rn | 3 | 7044406 | 7051016 | + | protein_coding | NA |
| ENSRNOG00000019995 | FALSE | TRUE | Dnajc18 | 18 | 27269355 | 27298344 | - | protein_coding | NA |
| ENSRNOG00000001518 | FALSE | TRUE | Itga6 | 3 | 56617268 | 56689428 | + | protein_coding | NA |

| | | | | | | | | | |
|---|---|---|---|---|---|---|---|---|---|
| ENSRNOG00000014837 | FALSE | TRUE | Emilin2 | 9 | 1.11E+08 | 1.11E+08 | - | protein_coding | NA |
| ENSRNOG00000056153 | FALSE | TRUE | Tent5b | 5 | 1.46E+08 | 1.46E+08 | + | protein_coding | NA |
| ENSRNOG00000007012 | FALSE | TRUE | Ly75 | 3 | 44765122 | 44852374 | - | protein_coding | NA |
| ENSRNOG00000064527 | FALSE | TRUE | ENSRNOG00000064527 | 3 | 4086582 | 4113617 | + | protein_coding | NA |
| ENSRNOG00000049052 | FALSE | TRUE | Sgk3 | 5 | 9346040 | 9415476 | - | protein_coding | NA |
| ENSRNOG00000067835 | FALSE | TRUE | ENSRNOG00000067835 | 12 | 35662951 | 35668551 | + | lncRNA | NA |
| ENSRNOG00000026432 | FALSE | TRUE | Syt7 | 1 | 2.07E+08 | 2.07E+08 | + | protein_coding | NA |
| ENSRNOG00000047712 | FALSE | TRUE | Dcx | X | 1.07E+08 | 1.08E+08 | - | protein_coding | NA |
| ENSRNOG00000004786 | FALSE | TRUE | Cyp4f1 | 7 | 12010852 | 12022046 | - | protein_coding | NA |
| ENSRNOG00000004680 | FALSE | TRUE | Kif5c | 3 | 34032105 | 34182413 | + | protein_coding | NA |
| ENSRNOG00000004810 | FALSE | TRUE | Plcb1 | 3 | 1.22E+08 | 1.23E+08 | + | protein_coding | NA |
| ENSRNOG00000069878 | FALSE | TRUE | ENSRNOG00000069878 | 13 | 85977035 | 85994718 | - | protein_coding | NA |
| ENSRNOG00000012762 | FALSE | TRUE | Zfp64 | 3 | 1.58E+08 | 1.58E+08 | - | protein_coding | zf-C2H2 |
| ENSRNOG00000006661 | FALSE | TRUE | Rad51c | 10 | 72205032 | 72231248 | - | protein_coding | NA |
| ENSRNOG00000020865 | FALSE | TRUE | Ano1 | 1 | 2E+08 | 2E+08 | - | protein_coding | NA |
| ENSRNOG00000066870 | FALSE | TRUE | ENSRNOG00000066870 | 3 | 1.1E+08 | 1.1E+08 | + | protein_coding | NA |
| ENSRNOG00000001347 | FALSE | TRUE | Adam1a | 12 | 35017315 | 35020311 | + | protein_coding | NA |
| ENSRNOG00000008932 | FALSE | TRUE | Ncapd3 | 8 | 25437123 | 25506373 | + | protein_coding | NA |
| ENSRNOG00000013097 | FALSE | TRUE | ENSRNOG00000013097 | 6 | 1.32E+08 | 1.32E+08 | + | protein_coding | NA |
| ENSRNOG00000009760 | FALSE | TRUE | Palm | 7 | 9886643 | 9912555 | - | protein_coding | NA |
| ENSRNOG00000068592 | FALSE | TRUE | Btn3a2 | 20 | 4398254 | 4405681 | + | protein_coding | NA |
| ENSRNOG00000002171 | FALSE | TRUE | Phldb2 | 11 | 54859135 | 55078467 | + | protein_coding | NA |
| ENSRNOG00000043471 | FALSE | TRUE | Krtap8-1 | 11 | 28718125 | 28718310 | - | protein_coding | NA |
| ENSRNOG00000045819 | FALSE | TRUE | H2aj | 4 | 1.7E+08 | 1.7E+08 | + | protein_coding | NA |
| ENSRNOG00000008960 | FALSE | TRUE | Fgf22 | 7 | 9948071 | 9950486 | - | protein_coding | NA |
| ENSRNOG00000054603 | FALSE | TRUE | Pacsin1 | 20 | 5719857 | 5765313 | + | protein_coding | NA |
| ENSRNOG00000007041 | FALSE | TRUE | Abcg2 | 4 | 87745319 | 87802409 | + | protein_coding | NA |
| ENSRNOG00000049383 | FALSE | TRUE | Krtap3-3 | 10 | 84517977 | 84518988 | - | protein_coding | NA |
| ENSRNOG00000010758 | FALSE | TRUE | Lpar2 | 16 | 19595655 | 19599998 | - | protein_coding | NA |
| ENSRNOG00000014686 | FALSE | TRUE | Kcnd3 | 2 | 1.93E+08 | 1.93E+08 | + | protein_coding | NA |
| ENSRNOG00000017912 | FALSE | TRUE | Atp2a3 | 10 | 57582128 | 57612748 | + | protein_coding | NA |
| ENSRNOG00000055790 | FALSE | TRUE | Nup210l | 2 | 1.76E+08 | 1.76E+08 | + | protein_coding | NA |
| ENSRNOG00000021272 | FALSE | TRUE | Mcm8 | 3 | 1.2E+08 | 1.2E+08 | + | protein_coding | NA |
| ENSRNOG00000049951 | FALSE | TRUE | NEWGENE_1310680 | 1 | 2.02E+08 | 2.02E+08 | - | protein_coding | NA |

| | | | | | | | | | |
|---|---|---|---|---|---|---|---|---|---|
| ENSRNOG00000015329 | FALSE | TRUE | Kpna2 | 10 | 91933951 | 91946150 | - | protein_coding | NA |
| ENSRNOG00000004535 | FALSE | TRUE | Kcng3 | 6 | 11051134 | 11099264 | + | protein_coding | NA |
| ENSRNOG00000013312 | FALSE | TRUE | Kcnt2 | 13 | 51664686 | 52056987 | + | protein_coding | NA |
| ENSRNOG00000026861 | FALSE | TRUE | Zscan18 | 1 | 73332889 | 73345897 | - | protein_coding | NA |
| ENSRNOG00000032708 | FALSE | TRUE | RT1-Bb | 20 | 4596559 | 4607597 | - | protein_coding | NA |
| ENSRNOG00000023881 | FALSE | TRUE | Skap1 | 10 | 81425525 | 81732650 | + | protein_coding | NA |
| ENSRNOG00000018615 | FALSE | TRUE | Cenph | 2 | 31894667 | 31910154 | - | protein_coding | NA |
| ENSRNOG00000042814 | FALSE | TRUE | Henmt1 | 2 | 1.97E+08 | 1.97E+08 | + | protein_coding | NA |
| ENSRNOG00000042360 | FALSE | TRUE | Treml4 | 9 | 12721815 | 12743253 | - | protein_coding | NA |
| ENSRNOG00000048636 | FALSE | TRUE | Il2rb | 7 | 1.1E+08 | 1.1E+08 | - | protein_coding | NA |
| ENSRNOG00000019432 | FALSE | TRUE | Lypd5 | 1 | 79935240 | 79941039 | + | protein_coding | NA |
| ENSRNOG00000007610 | FALSE | TRUE | Gdf11 | 7 | 1311732 | 1320725 | - | protein_coding | NA |
| ENSRNOG00000015061 | FALSE | TRUE | Igfals | 10 | 13898395 | 13902677 | + | protein_coding | NA |
| ENSRNOG00000048277 | FALSE | TRUE | AABR07035787.1 | 12 | 19392625 | 19406095 | + | protein_coding | NA |
| ENSRNOG00000020244 | FALSE | TRUE | Perm1 | 5 | 1.67E+08 | 1.67E+08 | + | protein_coding | NA |
| ENSRNOG00000033414 | FALSE | TRUE | Klk1c3 | 1 | 94671618 | 94675417 | + | protein_coding | NA |
| ENSRNOG00000049331 | FALSE | TRUE | RGD1559588 | 12 | 17398915 | 17404795 | + | protein_coding | NA |
| ENSRNOG00000007178 | FALSE | TRUE | Cd8a | 4 | 1.03E+08 | 1.03E+08 | + | protein_coding | NA |
| ENSRNOG00000028896 | FALSE | TRUE | A2m | 4 | 1.55E+08 | 1.55E+08 | + | protein_coding | NA |
| ENSRNOG00000036682 | FALSE | TRUE | Pycr1 | 10 | 1.06E+08 | 1.06E+08 | - | protein_coding | NA |
| ENSRNOG00000006305 | FALSE | TRUE | Slc38a2 | 7 | 1.28E+08 | 1.28E+08 | - | protein_coding | NA |
| ENSRNOG00000019342 | FALSE | TRUE | Sult1a1 | 1 | 1.81E+08 | 1.81E+08 | - | protein_coding | NA |
| ENSRNOG00000056836 | FALSE | TRUE | Cav1 | 4 | 45634918 | 45673705 | + | protein_coding | NA |
| ENSRNOG00000017506 | FALSE | TRUE | Cltb | 17 | 10001513 | 10019169 | + | protein_coding | NA |
| ENSRNOG00000016286 | FALSE | TRUE | Iyd | 1 | 40004041 | 40019319 | + | protein_coding | NA |
| ENSRNOG00000005261 | FALSE | TRUE | Fbxl5 | 14 | 67267801 | 67347383 | + | protein_coding | NA |
| ENSRNOG00000008507 | FALSE | TRUE | Cradd | 7 | 29798586 | 29952907 | - | protein_coding | NA |
| ENSRNOG00000030091 | FALSE | TRUE | Dusp14 | 10 | 68935330 | 68965329 | - | protein_coding | NA |
| ENSRNOG00000000640 | FALSE | TRUE | Egr2 | 20 | 21051277 | 21055562 | - | protein_coding | zf-C2H2 |
| ENSRNOG00000015585 | FALSE | TRUE | Suv39h2 | 17 | 74756306 | 74775332 | + | protein_coding | NA |
| ENSRNOG00000050215 | FALSE | TRUE | AABR07044375.1 | 20 | 3179438 | 3184250 | - | protein_coding | NA |
| ENSRNOG00000004276 | FALSE | TRUE | Itga3 | 10 | 79990161 | 80022118 | - | protein_coding | NA |
| ENSRNOG00000007989 | FALSE | TRUE | Chst1 | 3 | 78548525 | 78574883 | + | protein_coding | NA |
| ENSRNOG00000060934 | FALSE | TRUE | Ptx4 | 10 | 14140304 | 14144846 | + | protein_coding | NA |

| | | | | | | | | | |
|---|---|---|---|---|---|---|---|---|---|
| ENSRNOG00000036684 | FALSE | TRUE | Pcyt2 | 10 | 1.06E+08 | 1.06E+08 | - | protein_coding | NA |
| ENSRNOG00000024837 | FALSE | TRUE | Tpte2 | 16 | 69658875 | 69752949 | - | protein_coding | NA |
| ENSRNOG00000005347 | FALSE | TRUE | Fjx1 | 3 | 88774220 | 88776321 | - | protein_coding | NA |
| ENSRNOG00000063403 | FALSE | TRUE | ENSRNOG00000063403 | 2 | 1.97E+08 | 1.97E+08 | - | lncRNA | NA |
| ENSRNOG00000052407 | FALSE | TRUE | Btk | X | 97722802 | 97761853 | - | protein_coding | NA |
| ENSRNOG00000069426 | FALSE | TRUE | ENSRNOG00000069426 | 20 | 26763850 | 26764944 | + | lncRNA | NA |
| ENSRNOG00000024277 | FALSE | TRUE | Card11 | 12 | 13621087 | 13758112 | + | protein_coding | NA |
| ENSRNOG00000047367 | FALSE | TRUE | Card14 | 10 | 1.05E+08 | 1.05E+08 | + | protein_coding | NA |
| ENSRNOG00000007139 | FALSE | TRUE | Ttpa | 5 | 33497137 | 33518073 | + | protein_coding | NA |
| ENSRNOG00000009505 | FALSE | TRUE | Cd164l2 | 5 | 1.45E+08 | 1.45E+08 | + | protein_coding | NA |
| ENSRNOG00000057865 | FALSE | TRUE | Prss16 | 17 | 42514306 | 42521884 | + | protein_coding | NA |
| ENSRNOG00000021962 | FALSE | TRUE | Fzd2 | 10 | 87561326 | 87565334 | + | protein_coding | NA |
| ENSRNOG00000014961 | FALSE | TRUE | Pdpn | 5 | 1.56E+08 | 1.56E+08 | - | protein_coding | NA |
| ENSRNOG00000063200 | FALSE | TRUE | ENSRNOG00000063200 | 15 | 24407829 | 24420580 | - | lncRNA | NA |
| ENSRNOG00000052064 | FALSE | TRUE | Parvg | 7 | 1.15E+08 | 1.15E+08 | + | protein_coding | NA |
| ENSRNOG00000012543 | FALSE | TRUE | Mcm3 | 9 | 23219169 | 23237314 | - | protein_coding | NA |
| ENSRNOG00000002471 | FALSE | TRUE | Polq | 11 | 63673816 | 63775878 | - | protein_coding | NA |
| ENSRNOG00000007310 | FALSE | TRUE | Klrb1a | 4 | 1.62E+08 | 1.62E+08 | - | protein_coding | NA |
| ENSRNOG00000013463 | FALSE | TRUE | Kcnj8 | 4 | 1.76E+08 | 1.76E+08 | - | protein_coding | NA |
| ENSRNOG00000004682 | FALSE | TRUE | Parpbp | 7 | 22511427 | 22573695 | - | protein_coding | NA |
| ENSRNOG00000056983 | FALSE | TRUE | Trex2 | X | 1.51E+08 | 1.51E+08 | - | protein_coding | NA |
| ENSRNOG00000052572 | FALSE | TRUE | Aff2 | X | 1.48E+08 | 1.48E+08 | + | protein_coding | AF-4 |
| ENSRNOG00000014828 | FALSE | TRUE | Avpi1 | 1 | 2.41E+08 | 2.41E+08 | - | protein_coding | NA |
| ENSRNOG00000011526 | FALSE | TRUE | Pcsk6 | 1 | 1.19E+08 | 1.2E+08 | + | protein_coding | NA |
| ENSRNOG00000002602 | FALSE | TRUE | Serpinb12 | 13 | 23052448 | 23083691 | + | protein_coding | NA |
| ENSRNOG00000042825 | FALSE | TRUE | Cd300le | 10 | 1E+08 | 1E+08 | - | protein_coding | NA |
| ENSRNOG00000016793 | FALSE | TRUE | Tshb | 2 | 1.9E+08 | 1.9E+08 | - | protein_coding | NA |
| ENSRNOG00000002911 | FALSE | TRUE | Alb | 14 | 17607381 | 17622836 | - | protein_coding | NA |
| ENSRNOG00000038286 | FALSE | TRUE | Slamf6 | 13 | 84383742 | 84405300 | + | protein_coding | NA |
| ENSRNOG00000010718 | FALSE | TRUE | Gpr153 | 5 | 1.63E+08 | 1.63E+08 | + | protein_coding | NA |
| ENSRNOG00000059303 | FALSE | TRUE | ENSRNOG00000059303 | 1 | 1.99E+08 | 1.99E+08 | + | protein_coding | NA |
| ENSRNOG00000047247 | FALSE | TRUE | Ptprs | 9 | 1245410 | 1306947 | - | protein_coding | NA |
| ENSRNOG00000017063 | FALSE | TRUE | Fcna | 3 | 8445907 | 8449105 | - | protein_coding | NA |
| ENSRNOG00000003703 | FALSE | TRUE | Mcm6 | 13 | 39826763 | 39851960 | - | protein_coding | NA |

| | | | | | | | | | |
|---|---|---|---|---|---|---|---|---|---|
| ENSRNOG00000055111 | FALSE | TRUE | ENSRNOG00000055111 | 1 | 20099733 | 20101248 | - | protein_coding | NA |
| ENSRNOG00000017223 | FALSE | TRUE | Plg | 1 | 48325185 | 48367786 | - | protein_coding | NA |
| ENSRNOG00000020517 | FALSE | TRUE | Prrg2 | 1 | 95534290 | 95543425 | - | protein_coding | NA |
| ENSRNOG00000067434 | FALSE | TRUE | Hist1h2an | 17 | 41534691 | 41539869 | + | protein_coding | NA |
| ENSRNOG00000068643 | FALSE | TRUE | ENSRNOG00000068643 | 2 | 1.48E+08 | 1.48E+08 | + | lncRNA | NA |
| ENSRNOG00000010663 | FALSE | TRUE | Col6a5 | 8 | 1.06E+08 | 1.07E+08 | - | protein_coding | NA |
| ENSRNOG00000015505 | FALSE | TRUE | Mfap5 | 4 | 1.56E+08 | 1.56E+08 | + | protein_coding | NA |
| ENSRNOG00000020165 | FALSE | TRUE | Ahsp | 1 | 1.83E+08 | 1.83E+08 | + | protein_coding | NA |
| ENSRNOG00000036828 | FALSE | TRUE | Pde1b | 7 | 1.35E+08 | 1.35E+08 | + | protein_coding | NA |
| ENSRNOG00000005792 | FALSE | TRUE | Ankef1 | 3 | 1.24E+08 | 1.24E+08 | + | protein_coding | NA |
| ENSRNOG00000026594 | FALSE | TRUE | Tmem61 | 5 | 1.21E+08 | 1.21E+08 | - | protein_coding | NA |
| ENSRNOG00000033581 | FALSE | TRUE | AABR07044388.2 | 20 | 4026463 | 4085578 | - | protein_coding | NA |
| ENSRNOG00000009014 | FALSE | TRUE | Slc35f2 | 8 | 54159970 | 54203612 | + | protein_coding | NA |
| ENSRNOG00000006019 | FALSE | TRUE | G0s2 | 13 | 1.05E+08 | 1.05E+08 | - | protein_coding | NA |
| ENSRNOG00000065054 | FALSE | TRUE | Hist2h3c2 | 17 | 41378175 | 41378719 | - | protein_coding | NA |
| ENSRNOG00000014041 | FALSE | TRUE | Cep78 | 1 | 2.13E+08 | 2.13E+08 | - | protein_coding | NA |
| ENSRNOG00000021403 | FALSE | TRUE | ENSRNOG00000021403 | 6 | 31363548 | 31366108 | + | lncRNA | NA |
| ENSRNOG00000066647 | FALSE | TRUE | ENSRNOG00000066647 | 1 | 1.24E+08 | 1.24E+08 | + | lncRNA | NA |
| ENSRNOG00000010694 | FALSE | TRUE | Tpbg | 8 | 86793749 | 86797741 | + | protein_coding | NA |
| ENSRNOG00000042317 | FALSE | TRUE | Cd209f | 12 | 2484523 | 2487371 | + | protein_coding | NA |
| ENSRNOG00000064635 | FALSE | TRUE | Ly49si2 | 4 | 1.64E+08 | 1.64E+08 | - | protein_coding | NA |
| ENSRNOG00000036673 | FALSE | TRUE | Sectm1b | 10 | 1.06E+08 | 1.06E+08 | - | protein_coding | NA |
| ENSRNOG00000014703 | FALSE | TRUE | Tonsl | 7 | 1.08E+08 | 1.08E+08 | - | protein_coding | NA |
| ENSRNOG00000000900 | FALSE | TRUE | Tpst1 | 12 | 26544536 | 26605704 | - | protein_coding | NA |
| ENSRNOG00000014783 | FALSE | TRUE | Pgghg | 1 | 1.96E+08 | 1.96E+08 | + | protein_coding | NA |
| ENSRNOG00000038572 | FALSE | TRUE | Ncapg | 14 | 65404163 | 65432902 | - | protein_coding | NA |
| ENSRNOG00000056580 | FALSE | TRUE | En1 | 13 | 31751545 | 31755943 | + | protein_coding | Homeobox |
| ENSRNOG00000016467 | FALSE | TRUE | Kctd1 | 18 | 6122390 | 6317393 | - | protein_coding | NA |
| ENSRNOG00000060137 | FALSE | TRUE | AABR07037489.1 | X | 21447361 | 21471498 | - | protein_coding | NA |
| ENSRNOG00000020891 | FALSE | TRUE | Kif9 | 8 | 1.1E+08 | 1.11E+08 | + | protein_coding | NA |
| ENSRNOG00000030715 | FALSE | TRUE | Cfh | 13 | 51511828 | 51613838 | - | protein_coding | NA |
| ENSRNOG00000050024 | FALSE | TRUE | Ms4a4a | 1 | 2.08E+08 | 2.08E+08 | + | protein_coding | NA |
| ENSRNOG00000050706 | FALSE | TRUE | Col25a1 | 2 | 2.19E+08 | 2.19E+08 | + | protein_coding | NA |
| ENSRNOG00000069541 | FALSE | TRUE | ENSRNOG00000069541 | 5 | 1.24E+08 | 1.24E+08 | + | lncRNA | NA |

| | | | | | | | | | |
|---|---|---|---|---|---|---|---|---|---|
| ENSRNOG00000046484 | FALSE | TRUE | ENSRNOG00000046484 | X | 1.32E+08 | 1.32E+08 | + | protein_coding | NA |
| ENSRNOG00000048919 | FALSE | TRUE | Iho1 | 8 | 1.09E+08 | 1.09E+08 | - | protein_coding | NA |
| ENSRNOG00000005906 | FALSE | TRUE | RGD1565355 | 4 | 17354466 | 17513903 | + | protein_coding | NA |
| ENSRNOG00000012458 | FALSE | TRUE | Cyp2e1 | 1 | 1.96E+08 | 1.96E+08 | + | protein_coding | NA |
| ENSRNOG00000013729 | FALSE | TRUE | RGD1306271 | 4 | 66760162 | 66968436 | - | protein_coding | Others |
| ENSRNOG00000058308 | FALSE | TRUE | Asic2 | 10 | 65870594 | 66940424 | - | protein_coding | NA |
| ENSRNOG00000018706 | FALSE | TRUE | Il15ra | 17 | 66802334 | 66832278 | - | protein_coding | NA |
| ENSRNOG00000001501 | FALSE | TRUE | Snrpa | 1 | 82481770 | 82490538 | - | protein_coding | NA |
| ENSRNOG00000002636 | FALSE | TRUE | Abat | 10 | 6999819 | 7092835 | - | protein_coding | NA |
| ENSRNOG00000001399 | FALSE | TRUE | Tbx5 | 12 | 36688014 | 36734885 | - | protein_coding | T-box |
| ENSRNOG00000020952 | FALSE | TRUE | Cgn | 2 | 1.82E+08 | 1.82E+08 | - | protein_coding | NA |
| ENSRNOG00000003681 | FALSE | TRUE | Lct | 13 | 39781929 | 39824456 | - | protein_coding | NA |
| ENSRNOG00000008855 | FALSE | TRUE | Gjb2 | 15 | 31260357 | 31278177 | - | protein_coding | NA |
| ENSRNOG00000015346 | FALSE | TRUE | Obsl1 | 9 | 76974253 | 76993560 | - | protein_coding | NA |
| ENSRNOG00000060063 | FALSE | TRUE | ENSRNOG00000060063 | X | 1.52E+08 | 1.52E+08 | - | protein_coding | NA |
| ENSRNOG00000013426 | FALSE | TRUE | Mrgprf | 1 | 2E+08 | 2E+08 | + | protein_coding | NA |
| ENSRNOG00000004152 | FALSE | TRUE | Lrp12 | 7 | 70941068 | 71012441 | - | protein_coding | NA |
| ENSRNOG00000005492 | FALSE | TRUE | Hpcal1 | 6 | 40478208 | 40584687 | + | protein_coding | NA |
| ENSRNOG00000028958 | FALSE | TRUE | AABR07024795.1 | 16 | 15370293 | 15371300 | + | protein_coding | NA |
| ENSRNOG00000024566 | FALSE | TRUE | Sh3d21 | 5 | 1.38E+08 | 1.38E+08 | - | protein_coding | NA |
| ENSRNOG00000070575 | FALSE | TRUE | Tafa1 | 4 | 1.29E+08 | 1.29E+08 | + | protein_coding | NA |
| ENSRNOG00000004660 | FALSE | TRUE | Fzd6 | 7 | 70055068 | 70086776 | + | protein_coding | NA |
| ENSRNOG00000010463 | FALSE | TRUE | Cpa4 | 4 | 59188284 | 59212255 | + | protein_coding | NA |
| ENSRNOG00000019356 | FALSE | TRUE | Lrfn4 | 1 | 2.02E+08 | 2.02E+08 | - | protein_coding | NA |
| ENSRNOG00000020355 | FALSE | TRUE | Twist2 | 9 | 92374574 | 92419222 | + | protein_coding | bHLH |
| ENSRNOG00000011253 | FALSE | TRUE | Trmt9b | 16 | 55743018 | 55792144 | - | protein_coding | NA |
| ENSRNOG00000015101 | FALSE | TRUE | Mak | 17 | 23693878 | 23730001 | + | protein_coding | NA |
| ENSRNOG00000070375 | FALSE | TRUE | ENSRNOG00000070375 | 4 | 1.55E+08 | 1.55E+08 | + | protein_coding | NA |
| ENSRNOG00000002244 | FALSE | TRUE | Pdgfra | 14 | 33005839 | 33054335 | - | protein_coding | NA |
| ENSRNOG00000012208 | FALSE | TRUE | Itgb7 | 7 | 1.33E+08 | 1.33E+08 | - | protein_coding | NA |
| ENSRNOG00000068605 | FALSE | TRUE | ENSRNOG00000068605 | 1 | 7387083 | 7395497 | + | protein_coding | NA |
| ENSRNOG00000011649 | FALSE | TRUE | Epb42 | 3 | 1.08E+08 | 1.08E+08 | - | protein_coding | NA |
| ENSRNOG00000033090 | FALSE | TRUE | Ltbp1 | 6 | 20029629 | 20425349 | - | protein_coding | NA |
| ENSRNOG00000067595 | FALSE | TRUE | ENSRNOG00000067595 | 8 | 41755488 | 41902911 | + | lncRNA | NA |

| | | | | | | | | | |
|---|---|---|---|---|---|---|---|---|---|
| ENSRNOG00000007391 | FALSE | TRUE | Hes7 | 10 | 53825574 | 53828097 | + | protein_coding | bHLH |
| ENSRNOG00000025979 | FALSE | TRUE | Gramd2a | 8 | 60080338 | 60115842 | + | protein_coding | NA |
| ENSRNOG00000012337 | FALSE | TRUE | Pde1c | 4 | 85300858 | 85863219 | - | protein_coding | NA |
| ENSRNOG00000055078 | FALSE | TRUE | Cyp4b1 | 5 | 1.29E+08 | 1.29E+08 | - | protein_coding | NA |
| ENSRNOG00000055197 | FALSE | TRUE | AABR07027872.1 | 17 | 45680483 | 45686011 | - | protein_coding | NA |
| ENSRNOG00000003348 | FALSE | TRUE | Rasd1 | 10 | 44766455 | 44768186 | - | protein_coding | NA |
| ENSRNOG00000033923 | FALSE | TRUE | Aox2 | 9 | 59828084 | 59916146 | + | protein_coding | NA |
| ENSRNOG00000053232 | FALSE | TRUE | Ror2 | 17 | 11972830 | 12148152 | + | protein_coding | NA |
| ENSRNOG00000021056 | FALSE | TRUE | Kcnj14 | 1 | 96293435 | 96495459 | - | protein_coding | NA |
| ENSRNOG00000068145 | FALSE | TRUE | ENSRNOG00000068145 | 1 | 2.59E+08 | 2.59E+08 | + | lncRNA | NA |
| ENSRNOG00000005807 | FALSE | TRUE | Ptpn7 | 13 | 46571712 | 46583308 | + | protein_coding | NA |
| ENSRNOG00000071041 | FALSE | TRUE | ENSRNOG00000071041 | 11 | 28310977 | 28311204 | + | protein_coding | NA |
| ENSRNOG00000014683 | FALSE | TRUE | Il1rl2 | 9 | 42591934 | 42636667 | + | protein_coding | NA |
| ENSRNOG00000014786 | FALSE | TRUE | Ccne1 | 1 | 90781949 | 90791101 | - | protein_coding | NA |
| ENSRNOG00000005003 | FALSE | TRUE | Ptprn2 | 6 | 1.37E+08 | 1.38E+08 | + | protein_coding | NA |
| ENSRNOG00000016405 | FALSE | TRUE | Pcsk4 | 7 | 9381361 | 9389585 | + | protein_coding | NA |
| ENSRNOG00000025823 | FALSE | TRUE | Rbm46 | 2 | 1.68E+08 | 1.68E+08 | - | protein_coding | NA |
| ENSRNOG00000032884 | FALSE | TRUE | Scn11a | 8 | 1.19E+08 | 1.2E+08 | - | protein_coding | NA |
| ENSRNOG00000047960 | FALSE | TRUE | Rab9b | X | 1E+08 | 1E+08 | - | protein_coding | NA |
| ENSRNOG00000049922 | FALSE | TRUE | AC118963.1 | 5 | 1.46E+08 | 1.46E+08 | - | protein_coding | NA |
| ENSRNOG00000068603 | FALSE | TRUE | ENSRNOG00000068603 | 12 | 19323608 | 19326012 | - | lncRNA | NA |
| ENSRNOG00000024640 | FALSE | TRUE | Oacyl | 18 | 59212354 | 59263722 | + | protein_coding | NA |
| ENSRNOG00000008713 | FALSE | TRUE | Slc41a2 | 7 | 20383757 | 20491166 | + | protein_coding | NA |
| ENSRNOG00000034177 | FALSE | TRUE | Efna5 | 9 | 1.02E+08 | 1.03E+08 | - | protein_coding | NA |
| ENSRNOG00000008678 | FALSE | TRUE | Antxr1 | 4 | 1.2E+08 | 1.2E+08 | - | protein_coding | NA |
| ENSRNOG00000042533 | FALSE | TRUE | Accsl | 3 | 79826464 | 79841163 | - | protein_coding | NA |
| ENSRNOG00000017874 | FALSE | TRUE | Cd53 | 2 | 1.94E+08 | 1.94E+08 | - | protein_coding | NA |
| ENSRNOG00000008902 | FALSE | TRUE | Pon1 | 4 | 33294722 | 33321360 | - | protein_coding | NA |
| ENSRNOG00000003457 | FALSE | TRUE | Bicdl2 | 10 | 12691610 | 12700049 | + | protein_coding | NA |
| ENSRNOG00000014626 | FALSE | TRUE | Katnb1 | 19 | 9921219 | 9940650 | - | protein_coding | NA |
| ENSRNOG00000020793 | FALSE | TRUE | Ruvbl2 | 1 | 95901701 | 95915342 | - | protein_coding | NA |
| ENSRNOG00000014975 | FALSE | TRUE | Zfp483 | 5 | 73763179 | 73776223 | + | protein_coding | zf-C2H2 |
| ENSRNOG00000025498 | FALSE | TRUE | Crb2 | 3 | 21542221 | 21563294 | + | protein_coding | NA |
| ENSRNOG00000070864 | FALSE | TRUE | ENSRNOG00000070864 | 10 | 42506142 | 42512230 | - | lncRNA | NA |

| | | | | | | | | | |
|---|---|---|---|---|---|---|---|---|---|
| ENSRNOG00000069232 | FALSE | TRUE | ENSRNOG00000069232 | 9 | 1.05E+08 | 1.05E+08 | - | lncRNA | NA |
| ENSRNOG00000032133 | FALSE | TRUE | Rnase2 | 15 | 24449611 | 24450479 | - | protein_coding | NA |
| ENSRNOG00000015616 | FALSE | TRUE | Rgs14 | 17 | 9249019 | 9263104 | - | protein_coding | NA |
| ENSRNOG00000069665 | FALSE | TRUE | ENSRNOG00000069665 | 5 | 1.15E+08 | 1.15E+08 | - | lncRNA | NA |
| ENSRNOG00000001174 | FALSE | TRUE | Pde9a | 20 | 9469848 | 9562948 | + | protein_coding | NA |
| ENSRNOG00000007351 | FALSE | TRUE | Ggh | 5 | 33529880 | 33552787 | + | protein_coding | NA |
| ENSRNOG00000025731 | FALSE | TRUE | Ptma | 13 | 83921044 | 83922040 | - | protein_coding | NA |
| ENSRNOG00000018905 | FALSE | TRUE | Odad2 | 17 | 55218991 | 55409399 | - | protein_coding | NA |
| ENSRNOG00000019626 | FALSE | TRUE | Slc27a5 | 1 | 73616564 | 73627172 | - | protein_coding | NA |
| ENSRNOG00000012061 | FALSE | TRUE | Prkcb | 1 | 1.77E+08 | 1.77E+08 | + | protein_coding | NA |
| ENSRNOG00000005726 | FALSE | TRUE | Pclo | 4 | 19695315 | 20049885 | - | protein_coding | NA |
| ENSRNOG00000038948 | FALSE | TRUE | Stab2 | 7 | 21249391 | 21434042 | - | protein_coding | NA |
| ENSRNOG00000011016 | FALSE | TRUE | Slc7a2 | 16 | 51417493 | 51470784 | - | protein_coding | NA |
| ENSRNOG00000001628 | FALSE | TRUE | Pcp4 | 11 | 35800713 | 35861725 | + | protein_coding | NA |
| ENSRNOG00000064199 | FALSE | TRUE | Stfa2 | 11 | 64544466 | 64549448 | + | protein_coding | NA |
| ENSRNOG00000028377 | FALSE | TRUE | Mpl | 5 | 1.32E+08 | 1.32E+08 | - | protein_coding | NA |
| ENSRNOG00000023214 | FALSE | TRUE | Il20rb | 8 | 1.01E+08 | 1.01E+08 | - | protein_coding | NA |
| ENSRNOG00000023151 | FALSE | TRUE | ENSRNOG00000023151 | 1 | 2.06E+08 | 2.07E+08 | + | protein_coding | NA |
| ENSRNOG00000014960 | FALSE | TRUE | Bard1 | 9 | 72623155 | 72694265 | - | protein_coding | NA |
| ENSRNOG00000047924 | FALSE | TRUE | Zbtb7c | 18 | 69441616 | 69765297 | + | protein_coding | ZBTB |
| ENSRNOG00000064030 | FALSE | TRUE | ENSRNOG00000064030 | 18 | 59826321 | 59834159 | - | lncRNA | NA |
| ENSRNOG00000015421 | FALSE | TRUE | Slc27a3 | 2 | 1.76E+08 | 1.76E+08 | - | protein_coding | NA |
| ENSRNOG00000068695 | FALSE | TRUE | Diras3 | 6 | 1.31E+08 | 1.31E+08 | - | protein_coding | NA |
| ENSRNOG00000065844 | FALSE | TRUE | ENSRNOG00000065844 | 10 | 1.06E+08 | 1.06E+08 | - | protein_coding | NA |
| ENSRNOG00000011296 | FALSE | TRUE | Cenpn | 19 | 44968308 | 44994012 | + | protein_coding | NA |
| ENSRNOG00000049614 | FALSE | TRUE | AABR07028488.1 | 17 | 68252128 | 68352207 | - | protein_coding | NA |
| ENSRNOG00000002693 | FALSE | TRUE | Nme1 | 10 | 78906083 | 78916408 | - | protein_coding | NA |
| ENSRNOG00000018405 | FALSE | TRUE | Apoc4 | 1 | 79335745 | 79339981 | - | protein_coding | NA |
| ENSRNOG00000022822 | FALSE | TRUE | Fam169b | 1 | 1.22E+08 | 1.22E+08 | + | protein_coding | NA |
| ENSRNOG00000008807 | FALSE | TRUE | Rp1 | 5 | 15121133 | 15579363 | + | protein_coding | NA |
| ENSRNOG00000004298 | FALSE | TRUE | Dpys | 7 | 70835789 | 70929231 | - | protein_coding | NA |
| ENSRNOG00000005985 | FALSE | TRUE | Kcnma1 | 15 | 302214 | 1001198 | + | protein_coding | NA |
| ENSRNOG00000011504 | FALSE | TRUE | Akap2 | 5 | 72181718 | 72648524 | + | protein_coding | NA |
| ENSRNOG00000033722 | FALSE | TRUE | Rnf207 | 5 | 1.63E+08 | 1.63E+08 | - | protein_coding | NA |

| | | | | | | | | | |
|---|---|---|---|---|---|---|---|---|---|
| ENSRNOG00000042224 | FALSE | TRUE | Cyp2j10 | 5 | 1.11E+08 | 1.11E+08 | - | protein_coding | NA |
| ENSRNOG00000004645 | FALSE | TRUE | Galnt5 | 3 | 42573622 | 42619881 | + | protein_coding | NA |
| ENSRNOG00000012142 | FALSE | TRUE | Glyat | 1 | 2.1E+08 | 2.1E+08 | + | protein_coding | NA |
| ENSRNOG00000016583 | FALSE | TRUE | RGD1561517 | 3 | 1.43E+08 | 1.43E+08 | + | protein_coding | NA |
| ENSRNOG00000002042 | FALSE | TRUE | Gfi1 | 14 | 2042434 | 2051814 | + | protein_coding | zf-C2H2 |
| ENSRNOG00000003597 | FALSE | TRUE | Tuba4a | 9 | 76709614 | 76713918 | - | protein_coding | NA |
| ENSRNOG00000018796 | FALSE | TRUE | Herpud1 | 19 | 10598417 | 10618424 | - | protein_coding | NA |
| ENSRNOG00000016810 | FALSE | TRUE | Stmn1 | 5 | 1.47E+08 | 1.47E+08 | + | protein_coding | NA |
| ENSRNOG00000029939 | FALSE | TRUE | Gypc | 18 | 24160739 | 24193204 | - | protein_coding | NA |
| ENSRNOG00000028077 | FALSE | TRUE | Cfap44 | 11 | 56201049 | 56283986 | - | protein_coding | NA |
| ENSRNOG00000011796 | FALSE | TRUE | C1r | 4 | 1.57E+08 | 1.57E+08 | + | protein_coding | NA |
| ENSRNOG00000018953 | FALSE | TRUE | Cblc | 1 | 79439977 | 79456755 | - | protein_coding | NA |
| ENSRNOG00000024330 | FALSE | TRUE | Ngp | 8 | 1.1E+08 | 1.1E+08 | + | protein_coding | NA |
| ENSRNOG00000042899 | FALSE | TRUE | Fcgbpl1 | 1 | 83424788 | 83462799 | + | protein_coding | NA |
| ENSRNOG00000056001 | FALSE | TRUE | Sftpd | 16 | 17046483 | 17059927 | - | protein_coding | NA |
| ENSRNOG00000064814 | FALSE | TRUE | ENSRNOG00000064814 | 1 | 94432638 | 94433770 | + | protein_coding | NA |
| ENSRNOG00000066767 | FALSE | TRUE | ENSRNOG00000066767 | 2 | 1.47E+08 | 1.47E+08 | - | protein_coding | NA |
| ENSRNOG00000016116 | FALSE | TRUE | Hoxc12 | 7 | 1.34E+08 | 1.34E+08 | + | protein_coding | Homeobox |
| ENSRNOG00000001570 | FALSE | TRUE | 2310061N02Rik | 11 | 28023949 | 28024476 | - | protein_coding | NA |
| ENSRNOG00000023143 | FALSE | TRUE | Nlrp1a | 10 | 55778560 | 55825180 | - | protein_coding | NA |
| ENSRNOG00000007262 | FALSE | TRUE | Ccdc134 | 7 | 1.14E+08 | 1.14E+08 | + | protein_coding | NA |
| ENSRNOG00000014156 | FALSE | TRUE | Fut7 | 3 | 8239384 | 8242260 | + | protein_coding | NA |
| ENSRNOG00000047656 | FALSE | TRUE | Gaa | 10 | 1.05E+08 | 1.05E+08 | + | protein_coding | NA |
| ENSRNOG00000016037 | FALSE | TRUE | Mafb | 3 | 1.49E+08 | 1.49E+08 | - | protein_coding | TF_bZIP |
| ENSRNOG00000067013 | FALSE | TRUE | ENSRNOG00000067013 | 11 | 29261318 | 29380097 | - | lncRNA | NA |
| ENSRNOG00000028661 | FALSE | TRUE | Ctcfl | 3 | 1.62E+08 | 1.62E+08 | - | protein_coding | zf-C2H2 |
| ENSRNOG00000015982 | FALSE | TRUE | Wnt11 | 1 | 1.53E+08 | 1.53E+08 | + | protein_coding | NA |
| ENSRNOG00000021151 | FALSE | TRUE | Ppp1r14b | 1 | 2.04E+08 | 2.04E+08 | + | protein_coding | NA |
| ENSRNOG00000016469 | FALSE | TRUE | Fxyd2 | 8 | 45712903 | 45720203 | + | protein_coding | NA |
| ENSRNOG00000036719 | FALSE | TRUE | Samd10 | 3 | 1.69E+08 | 1.69E+08 | - | protein_coding | NA |
| ENSRNOG00000031440 | FALSE | TRUE | Nova1 | 6 | 63783489 | 63906289 | - | protein_coding | NA |
| ENSRNOG00000037509 | FALSE | TRUE | Chek2 | 12 | 45788827 | 45821286 | - | protein_coding | NA |
| ENSRNOG00000057760 | FALSE | TRUE | Stil | 5 | 1.29E+08 | 1.29E+08 | + | protein_coding | NA |
| ENSRNOG00000065957 | FALSE | TRUE | ENSRNOG00000065957 | 1 | 2.59E+08 | 2.59E+08 | - | lncRNA | NA |

| | | | | | | | | | |
|---|---|---|---|---|---|---|---|---|---|
| ENSRNOG00000011718 | FALSE | TRUE | C1rl | 4 | 1.57E+08 | 1.57E+08 | + | protein_coding | NA |
| ENSRNOG00000046377 | FALSE | TRUE | Btnl9 | 10 | 33470536 | 33489025 | - | protein_coding | NA |
| ENSRNOG00000025619 | FALSE | TRUE | Ap1g2 | 15 | 28571568 | 28589004 | - | protein_coding | NA |
| ENSRNOG00000005437 | FALSE | TRUE | Rida | 7 | 65691435 | 65705716 | - | protein_coding | NA |
| ENSRNOG00000005478 | FALSE | TRUE | Fkbp9 | 4 | 86106282 | 86156721 | + | protein_coding | NA |
| ENSRNOG00000012566 | FALSE | TRUE | Kcnv2 | 1 | 2.25E+08 | 2.25E+08 | + | protein_coding | NA |
| ENSRNOG00000019141 | FALSE | TRUE | Ch25h | 1 | 2.32E+08 | 2.32E+08 | - | protein_coding | NA |
| ENSRNOG00000002322 | FALSE | TRUE | RGD1310587 | 13 | 96422302 | 96432068 | - | protein_coding | NA |
| ENSRNOG00000005248 | FALSE | TRUE | Slc1a4 | 14 | 94529084 | 94560418 | - | protein_coding | NA |
| ENSRNOG00000052858 | FALSE | TRUE | AABR07025896.1 | 16 | 51348173 | 51348550 | + | protein_coding | NA |
| ENSRNOG00000009314 | FALSE | TRUE | Ivl | 2 | 1.78E+08 | 1.78E+08 | - | protein_coding | NA |
| ENSRNOG00000023348 | FALSE | TRUE | Tbc1d2 | 5 | 60884124 | 60931352 | - | protein_coding | NA |
| ENSRNOG00000067496 | FALSE | TRUE | ENSRNOG00000067496 | 2 | 1.78E+08 | 1.78E+08 | - | protein_coding | NA |
| ENSRNOG00000062875 | FALSE | TRUE | ENSRNOG00000062875 | 2 | 76303754 | 76305619 | - | lncRNA | NA |
| ENSRNOG00000018366 | FALSE | TRUE | Cracdl | 9 | 39834833 | 39951086 | - | protein_coding | NA |
| ENSRNOG00000006979 | FALSE | TRUE | Hpca | 5 | 1.41E+08 | 1.41E+08 | - | protein_coding | NA |
| ENSRNOG00000037753 | FALSE | TRUE | Slc10a2 | 16 | 84374862 | 84409475 | + | protein_coding | NA |
| ENSRNOG00000005580 | FALSE | TRUE | Itgb4 | 10 | 1.01E+08 | 1.01E+08 | + | protein_coding | NA |
| ENSRNOG00000055281 | FALSE | TRUE | Dcbld2 | 11 | 42125787 | 42179697 | - | protein_coding | NA |
| ENSRNOG00000007722 | FALSE | TRUE | Tlcd3a | 10 | 61058042 | 61065283 | + | protein_coding | NA |
| ENSRNOG00000007970 | FALSE | TRUE | Plxnc1 | 7 | 29390048 | 29543779 | - | protein_coding | NA |
| ENSRNOG00000024689 | FALSE | TRUE | Hopx | 14 | 31075148 | 31082909 | + | protein_coding | Homeobox |
| ENSRNOG00000071138 | FALSE | TRUE | ENSRNOG00000071138 | 10 | 85882413 | 85884081 | - | lncRNA | NA |
| ENSRNOG00000028679 | FALSE | TRUE | Hoxc13 | 7 | 1.34E+08 | 1.34E+08 | + | protein_coding | Homeobox |
| ENSRNOG00000030058 | FALSE | TRUE | ENSRNOG00000030058 | 6 | 7539156 | 7541159 | + | lncRNA | NA |
| ENSRNOG00000022391 | FALSE | TRUE | Zfp711 | X | 77646558 | 77678045 | + | protein_coding | zf-C2H2 |
| ENSRNOG00000018584 | FALSE | TRUE | Ptma | 9 | 87176230 | 87180333 | + | protein_coding | NA |
| ENSRNOG00000048264 | FALSE | TRUE | H4f3 | 17 | 41529667 | 41552334 | - | protein_coding | NA |
| ENSRNOG00000066437 | FALSE | TRUE | Ntm | 8 | 27377773 | 28366595 | - | protein_coding | NA |
| ENSRNOG00000001942 | FALSE | TRUE | Smr3b | 14 | 19783816 | 19788492 | - | protein_coding | NA |
| ENSRNOG00000053017 | FALSE | TRUE | AC131411.2 | 14 | 9644582 | 9681205 | - | protein_coding | NA |
| ENSRNOG00000007682 | FALSE | TRUE | Gria3 | X | 1.2E+08 | 1.21E+08 | + | protein_coding | NA |
| ENSRNOG00000008133 | FALSE | TRUE | Mfng | 7 | 1.1E+08 | 1.1E+08 | - | protein_coding | NA |
| ENSRNOG00000016838 | FALSE | TRUE | Pla2g5 | 5 | 1.51E+08 | 1.51E+08 | - | protein_coding | NA |

| | | | | | | | | | |
|---|---|---|---|---|---|---|---|---|---|
| ENSRNOG00000066314 | FALSE | TRUE | Bean1 | 19 | 739551 | 787537 | - | protein_coding | NA |
| ENSRNOG00000004006 | FALSE | TRUE | Dnajb9 | 6 | 61269913 | 61275063 | - | protein_coding | NA |
| ENSRNOG00000018991 | FALSE | TRUE | Gsn | 3 | 18585172 | 18638402 | + | protein_coding | NA |
| ENSRNOG00000028636 | FALSE | TRUE | Xkr5 | 16 | 70875093 | 70892967 | + | protein_coding | NA |
| ENSRNOG00000002886 | FALSE | TRUE | Myh10 | 10 | 53394389 | 53525165 | + | protein_coding | NA |
| ENSRNOG00000016606 | FALSE | TRUE | Snorc | 9 | 88140419 | 88145891 | + | protein_coding | NA |
| ENSRNOG00000016050 | FALSE | TRUE | Fgfr1 | 16 | 66494042 | 66547350 | - | protein_coding | NA |
| ENSRNOG00000018516 | FALSE | TRUE | Impa2 | 18 | 60834246 | 60865641 | + | protein_coding | NA |
| ENSRNOG00000002597 | FALSE | TRUE | Hsd17b6 | 7 | 422480 | 442425 | - | protein_coding | NA |
| ENSRNOG00000070264 | FALSE | TRUE | ENSRNOG00000070264 | 7 | 10167309 | 10169477 | + | protein_coding | NA |
| ENSRNOG00000027540 | FALSE | TRUE | Fam102b | 2 | 1.97E+08 | 1.97E+08 | - | protein_coding | NA |
| ENSRNOG00000004232 | FALSE | TRUE | G2e3 | 6 | 68764185 | 68793924 | + | protein_coding | NA |
| ENSRNOG00000022261 | FALSE | TRUE | Cenpu | 16 | 45714294 | 45738823 | - | protein_coding | NA |
| ENSRNOG00000010721 | FALSE | TRUE | Dlgap5 | 15 | 20633966 | 20664518 | - | protein_coding | NA |
| ENSRNOG00000014104 | FALSE | TRUE | Myo5b | 18 | 68038759 | 68338745 | + | protein_coding | NA |
| ENSRNOG00000019328 | FALSE | TRUE | Phgdh | 2 | 1.86E+08 | 1.86E+08 | - | protein_coding | NA |
| ENSRNOG00000069891 | FALSE | TRUE | Spem3 | 10 | 54532099 | 54536460 | - | protein_coding | NA |
| ENSRNOG00000036911 | FALSE | TRUE | Bicd1 | 4 | 1.82E+08 | 1.83E+08 | + | protein_coding | NA |
| ENSRNOG00000006048 | FALSE | TRUE | Ezh2 | 4 | 76624399 | 76687362 | - | protein_coding | NA |
| ENSRNOG00000066130 | FALSE | TRUE | ENSRNOG00000066130 | 7 | 9951428 | 9953419 | + | lncRNA | NA |
| ENSRNOG00000019022 | FALSE | TRUE | Fam89a | 19 | 52710019 | 52722631 | - | protein_coding | NA |
| ENSRNOG00000001260 | FALSE | TRUE | Nudt1 | 12 | 14302694 | 14305826 | - | protein_coding | NA |
| ENSRNOG00000064329 | FALSE | TRUE | ENSRNOG00000064329 | 2 | 5188469 | 5189793 | - | lncRNA | NA |
| ENSRNOG00000046479 | FALSE | TRUE | Chaf1a | 9 | 874051 | 900654 | + | protein_coding | NA |
| ENSRNOG00000049491 | FALSE | TRUE | RT1-DMb | 20 | 4693103 | 4700340 | - | protein_coding | NA |
| ENSRNOG00000069550 | FALSE | TRUE | ENSRNOG00000069550 | 10 | 51650386 | 51659400 | + | lncRNA | NA |
| ENSRNOG00000068034 | FALSE | TRUE | ENSRNOG00000068034 | 17 | 42760219 | 42793359 | - | protein_coding | NA |
| ENSRNOG00000002387 | FALSE | TRUE | ENSRNOG00000002387 | X | 1E+08 | 1E+08 | + | protein_coding | NA |
| ENSRNOG00000049028 | FALSE | TRUE | C5ar2 | 1 | 76930383 | 76959517 | - | protein_coding | NA |
| ENSRNOG00000013902 | FALSE | TRUE | P2ry12 | 2 | 1.43E+08 | 1.44E+08 | - | protein_coding | NA |
| ENSRNOG00000058003 | FALSE | TRUE | Spon1 | 1 | 1.68E+08 | 1.68E+08 | + | protein_coding | NA |
| ENSRNOG00000038197 | FALSE | TRUE | Cd226 | 18 | 82450568 | 82543051 | + | protein_coding | NA |
| ENSRNOG00000021903 | FALSE | TRUE | Atad5 | 10 | 65070689 | 65117845 | + | protein_coding | NA |
| ENSRNOG00000003587 | FALSE | TRUE | Vegfd | X | 30074163 | 30108295 | - | protein_coding | NA |

| | | | | | | | | | |
|---|---|---|---|---|---|---|---|---|---|
| ENSRNOG00000015602 | FALSE | TRUE | Cdh2 | 18 | 7776704 | 7990167 | - | protein_coding | NA |
| ENSRNOG00000065113 | FALSE | TRUE | ENSRNOG00000065113 | 19 | 10075536 | 10077345 | - | lncRNA | NA |
| ENSRNOG00000014953 | FALSE | TRUE | Fbxo39 | 10 | 56932297 | 56934906 | + | protein_coding | NA |
| ENSRNOG00000018602 | FALSE | TRUE | Camta1 | 5 | 1.62E+08 | 1.62E+08 | - | protein_coding | CG-1 |
| ENSRNOG00000019614 | FALSE | TRUE | Ptpn22 | 2 | 1.91E+08 | 1.91E+08 | + | protein_coding | NA |
| ENSRNOG00000050647 | FALSE | TRUE | ENSRNOG00000050647 | 20 | 3856006 | 3873240 | + | protein_coding | NA |
| ENSRNOG00000006646 | FALSE | TRUE | Vopp1 | 4 | 87363477 | 87450375 | + | protein_coding | NA |
| ENSRNOG00000068632 | FALSE | TRUE | ENSRNOG00000068632 | 19 | 57293755 | 57317355 | + | protein_coding | zf-C2H2 |
| ENSRNOG00000069865 | FALSE | TRUE | Zfp37 | 5 | 75598732 | 75638942 | - | protein_coding | zf-C2H2 |
| ENSRNOG00000021250 | FALSE | TRUE | Rnf24 | 3 | 1.19E+08 | 1.19E+08 | - | protein_coding | NA |
| ENSRNOG00000058497 | FALSE | TRUE | Fdxr | 10 | 1.01E+08 | 1.01E+08 | - | protein_coding | NA |
| ENSRNOG00000001869 | FALSE | TRUE | Rab44 | 20 | 7164720 | 7198468 | + | protein_coding | NA |
| ENSRNOG00000021578 | FALSE | TRUE | Wfikkn1 | 10 | 14896378 | 14899863 | - | protein_coding | NA |
| ENSRNOG00000010410 | FALSE | TRUE | Serpina3c | 6 | 1.23E+08 | 1.23E+08 | - | protein_coding | NA |
| ENSRNOG00000064310 | FALSE | TRUE | ENSRNOG00000064310 | X | 20724926 | 20726597 | + | lncRNA | NA |
| ENSRNOG00000066886 | FALSE | TRUE | ENSRNOG00000066886 | 4 | 1.7E+08 | 1.7E+08 | - | protein_coding | NA |
| ENSRNOG00000001261 | FALSE | TRUE | Ftcd | 20 | 12055208 | 12068735 | - | protein_coding | NA |
| ENSRNOG00000001134 | FALSE | TRUE | Rfc5 | 12 | 39207484 | 39217312 | + | protein_coding | NA |
| ENSRNOG00000067285 | FALSE | TRUE | ENSRNOG00000067285 | 20 | 1605801 | 1606455 | - | protein_coding | NA |
| ENSRNOG00000017392 | FALSE | TRUE | Fgf2 | 2 | 1.2E+08 | 1.2E+08 | + | protein_coding | NA |
| ENSRNOG00000025108 | FALSE | TRUE | Ankrd35 | 2 | 1.84E+08 | 1.84E+08 | + | protein_coding | NA |
| ENSRNOG00000069525 | FALSE | TRUE | ENSRNOG00000069525 | 6 | 24850005 | 24856949 | + | lncRNA | NA |
| ENSRNOG00000063866 | FALSE | TRUE | ENSRNOG00000063866 | 12 | 18792843 | 18799561 | - | protein_coding | NA |
| ENSRNOG00000020621 | FALSE | TRUE | Dcst1 | 2 | 1.75E+08 | 1.75E+08 | - | protein_coding | NA |
| ENSRNOG00000064401 | FALSE | TRUE | ENSRNOG00000064401 | 7 | 1.13E+08 | 1.13E+08 | - | lncRNA | NA |
| ENSRNOG00000069950 | FALSE | TRUE | ENSRNOG00000069950 | 1 | 2.44E+08 | 2.44E+08 | + | lncRNA | NA |
| ENSRNOG00000015248 | FALSE | TRUE | Abca12 | 9 | 72823350 | 72996049 | - | protein_coding | NA |
| ENSRNOG00000061238 | FALSE | TRUE | Wnt10b | 7 | 1.3E+08 | 1.3E+08 | - | protein_coding | NA |
| ENSRNOG00000010362 | FALSE | TRUE | Anxa2 | 8 | 70105253 | 70141658 | + | protein_coding | NA |
| ENSRNOG00000004216 | FALSE | TRUE | Lrr1 | 6 | 87643217 | 87651894 | + | protein_coding | NA |
| ENSRNOG00000001425 | FALSE | TRUE | Sh2b2 | 12 | 20429043 | 20456844 | + | protein_coding | NA |
| ENSRNOG00000065330 | FALSE | TRUE | Mrgpre | 1 | 1.99E+08 | 1.99E+08 | - | protein_coding | NA |
| ENSRNOG00000022899 | FALSE | TRUE | Cdk15 | 9 | 60711715 | 60802777 | + | protein_coding | NA |
| ENSRNOG00000021750 | FALSE | TRUE | Id1 | 3 | 1.41E+08 | 1.41E+08 | + | protein_coding | bHLH |

| | | | | | | | | | |
|---|---|---|---|---|---|---|---|---|---|
| ENSRNOG00000000368 | FALSE | TRUE | Grik2 | 20 | 52133851 | 52838375 | - | protein_coding | NA |
| ENSRNOG00000002487 | FALSE | TRUE | Mbnl3 | X | 1.31E+08 | 1.31E+08 | - | protein_coding | NA |
| ENSRNOG00000025360 | FALSE | TRUE | Exd1 | 3 | 1.07E+08 | 1.07E+08 | - | protein_coding | NA |
| ENSRNOG00000038225 | FALSE | TRUE | Fbxo15 | 18 | 78319534 | 78390765 | + | protein_coding | NA |

## References

11 Luo, S. Enlightenment on the treatment of impotence with *Cinnamomum cassia* (Rou Gui) based on Zhang Jingyue's theory. *Proceedings of the fifth academic conference of the diagnostic specialty of the Guizhou association of integrated traditional Chinese and western medicine*. **2014**, 2014: 73-75.

12 Chen, X.Q.; Jiang, R. Research progress on *Epimedium brevicornu* in the treatment of erectile dysfunction. *Chin J Androl*. **2015**, 29: 70-72.

13 Wang, Q.X. Preliminary investigation on the kidney-yang-activating activity screening and mechanism of *Cistanche deserticola* in anti-fatigue and reproductive-promoting effects. Master's Thesis, *Beijing University of Chinese Medicine*. Beijing, China, **2020**.

37 Chen, X.Q.; Chen, Z.W.; Zhao, J.Y. Application of *Corii Colla Asini* in male diseases. *Chin J Libr Inf Sci Trad Chin Med*. **2016**, 40: 62-64.

38 Chinese Pharmacopoeia Commission. Pharmacopoeia of the People's Republic of China 2025. Beijing: People's Medical Publishing House.

39 Lu, W. *Ligusticum chuanxiong* hort. for the treatment of impotence: A case report. *Shizhen Journal of TCM*. **2000**, 11: 337.

40 Gu, C.J.; Wu, X.M.; Wang. H. Experience in the treatment of diabetic erectile dysfunction with *Ligusticum Wallichii*, Radix *Paeoniae Alba* and *Centipede* —— Three prescription by professor TONG Xiaolin. *Jilin J Chin Med*. **2020,** 40: 1417-1419.

41 Zhang, K.K.; Zhang, G.H.; Yang. H.J. Theoretical analysis of professor Zhang Guohai's application of Danggui Runzao Decoction in treating internal medicine diseases. *TCM Res*. **2024**, 37: 13-16.

42 Wang, J.S. Treatment of 40 cases of impotence with modified Dihuang Yinzi. *Sichuan Trad Chin Med*. **1996**, 14: 26.

43 Liu, L.M. The mechanism of earthworm protein in improving erectile dysfunction in diabetic rats based on PI3K/Akt signaling pathway. Master's Thesis, *Gansu University of Chinese Medicine*. Lanzhou, China, **2024**.

44 Wang, H.; Zhang, J.W.; Gao, Q.H.; Liu, J.S.; Deng, Y.J.; Guo, J.; Zhao, F.; Guo, J. Exploration of the therapeutic effect of Fuzi on impotence based on the theory that "Prolonged Injury to the Five Zang-Organs Will Eventually Involve the Kidney". *Global TCM*. **2021**, 14: 1646-1649.

45 Wan, H.Q.; Li. Z.J., Xu, G.H.; Zheng, L.Y.; Lin, J.B. Mechanism of action of the "Reinforcing Yang and Nourishing Essence" effects of *Hippocampus* (Sea Horse) based on network pharmacology: An exploration of the material basis, potential targets, and molecular mechanisms. *Tradit Chin Med J*. **2020**, 19: 32-37.

46 Wang, H.W.; Wang, H.J.; Zhu, H.Y. Treatment of impotence with Heshouwu Powder. *China Naturopathy*. **1998**, 4: 55-56.

47 Zhu, S.K. Chuanjiao is efficacious for treating male sexual disorders. **Zhejiang J Tradit Chin Med**. **1996**, 2: 69.

48 Zhou, K.; Ye, M.Y.; Zhao, F.; Ma, K.; Lyu, B.D.; Xu, Z.B. Effect of the *Astragalus* dose in Buyang Huanwu decoction on erectile dysfunction in rats with cavernous nerve injury. *Acta Lab Anim Sci Sin*. **2020**, 28: 788-795.

49 Xiao, Y.; Guang, X.M.; Liao, G.H. Tribulus terrestris and gross saponin of *Tribulus terrestris* in treating erectile dysfunction an update. *Chin J Hum Sex*. **2023**, 32: 109-112.

50 Liu, Y.N. Efficacy of modified Chaihu Plus Longgu Muli Decoction combined with Viagra in the treatment of impotence. *Chin J Urban Rural Enterprise Hyg*. **2021**, 3: 193-194.

51 Cui, Y.G. Clinical efficacy of self-prepared Lurong Yishen Capsules on impotence with kidney-yang deficiency: An observational study. *Chin Community Dr*. **2020**, 36: 102-103.

52 Medicinal king verses for clinical application. Clove (Dingxiang): The king of relieving hiccup. *Chin Med Mod Distance Educ China*. **2013,** 20: 148.

53 Wang, T.C.; Long, Y.; Zhu, Yong. Research progress on the clinical application of *Achyranthis Bidentatae* Radix in andrology. *Chin J Androl*. **2025**, 39: 133-136.

54 Chen, L.L.; Jia, W.G.; Su, H.; Wang, Y.Q. Mechanism and clinical studies on ginseng for treatment of erectile dysfunction. *Drugs & Clinic*. **2010**, 25: 116-120.

55 Yue, Z.X.; He, J.A.; Yi, Q.; Zhang, P.H., Zhong. Z.D. Effect of *Dioscorea opposite* and *Crataegus pinnatifida* Tea on the expression of NF-κB in the penis of diabetic impotence model rats. *Xinjiang J Tradit Chin Med*. **2023**, 41: 40-43.

56 Dogwood and prepared Rehmannia for treating impotence. *Hunan J Tradit Chin Med*. **2019**, 35: 137.

57 Gao, J.; Zhang, J.M. Application review on clinical application of Xianmao to infertility treatment. *J Tradit Chin Med Lit*. **2019**, 5: 65-72.

58 Zhang, F.; Zhang, J.Y.; Yang, A.L.; Yang, F.; Yu, X.J. Based on network pharmacology and molecular docking to explore the molecular mechanism of "*Curculiginis rhizome* and *epimrdii* herba" in the treatment of erectile dysfunction. *Chin J Androl*. **2022**, 36: 51-58.

59 Yang, C.X. *Fennel* (Xiaohuixiang) and prepared *Ginger* (Paojiang) for treating impotence. *Home Med*. **2013**, 11: 54.

60 Yang, M.H.; Wang, J.Y.; Zhang, S.W.; Yang, S.W.; Dai, D.Y.; Li, Z.X. Experimental study on strengthening yang action of traditional Chinese medicine actinolite. *J Pharm Res*. **2010**, 19: 17-18.

61 Zhang, G.Z.; Huo, Q.L.; Wang, J.S.; Zhao, Q.; Li, H.S. Mechanism research of *Epimedium-Morinda* in treating erectile dysfunction based on network pharmacological method. *World Chin Med*. **2021**, 16: 2275-2281.

62 Xu, J.W. Clinical observation of Yuanzhi Decoction combined with tadalafil in the treatment of disquieted heart spirit impotence. Master's Thesis, *Heilongjiang University of Chinese Medicine*. Harbin, China, **2023**.
